# Comprehensive analysis of the immunogenic cell death-related signature for predicting prognosis and immunotherapy efficiency in patients with lung adenocarcinoma

**DOI:** 10.1186/s12920-023-01604-w

**Published:** 2023-08-08

**Authors:** Yingshu Cui, Yi Li, Shan Long, Yuanyuan Xu, Xinxin Liu, Zhijia Sun, Yuanyuan Sun, Jia Hu, Xiaosong Li

**Affiliations:** 1 Department of Oncology, The Fifth Medical Center, Chinese PLA General Hospital, Beijing, China; 2 Medical School of Chinese PLA, Beijing, China; 3 School of Medicine, Nankai University, Tianjin, China; 4 Department of Oncology, The Affiliated Hospital of Southwest Medical University, Sichuan, China; 5 Department of General Surgery, Peking University First Hospital, Beijing, China; 6 Department of Radiation Oncology, Air Force General Hospital, Beijing, China

**Keywords:** Lung adenocarcinoma, Immunogenic cell death, Tumor microenvironment, Immunotherapy efficacy, Prognosis

## Abstract

**Background:**

Although immunotherapy has been considered as a potent strategy for lung adenocarcinoma (LUAD), only a small part of patients was served as potentially clinical benefiters. Immunogenic cell death (ICD), a type of regulated cell death (RCD), which enable to reshape the tumor immune microenvironment and contribute to the immunotherapy efficiency. Developing a novel ICD-based signature may be a potential strategy to differentiate prognosis of patients with LUAD and predict efficacy of immunotherapy.

**Methods:**

In this study, 34 ICD-related genes (ICDRGs) were identified and analyzed in LUAD samples from the Cancer Genome Atlas (TCGA). 572 patients with LUAD were divided into two distinct clusters according to ICDRGs expression levels. Patients were subsequently classified into two distinct gene subtypes based on differentially expressed genes (DEGs) analyzed between two ICD-related clusters. We further developed and validated a novel ICD-related score (ICDRS) followed by comprehensive investigation about the landscape of the prognosis, immune-based features, immunotherapautic responses and sensitivity of target drugs in patients with LUAD.

**Results:**

After confirming transcriptomic aberrations and appraising prognostic value of ICDRGs, two ICD-associated subtypes were initially determined by consensus clustering in accordance with differentially expressional levels of ICDRGs. It was shown that patients in the ICD high-subtype possessed the superior clinical prognosis, abundant immune cell infiltration and higher involvement in immune-related signaling compared with the ICD low-subtype. A signature of ICD-related score (ICDRS) was further established and validated, which was served as an independent prognostic indicator for LUAD patients. These comprehensive results revealed that the high-score patients represented better clinical prognosis, higher immune infiltration-related characteristics, stronger expression of immune checkpoints, and better response to immune checkpoint inhibitor therapy and multiple targeted drugs. To further verify our analysis, we selected TLR4 as the representative of ICDRGs and evaluated its expression on the lung normal cells and cancer cells in vitro. Then, relative animal experiments were performed in vivo, with results of that the stimulation of TLR4 suppressed the growth of lung cancer.

**Conclusions:**

In conclusion, our comprehensive analysis of ICDRGs in LUAD demonstrated their function in serving as a biomarker of predicting prognosis and clinical effects of immunotherapy and targeted drugs, which is meaningful to improve our understanding of ICDRGs and brought inspirations about evaluating prognosis and developing effective therapeutic strategies to patients with LUAD.

**Supplementary Information:**

The online version contains supplementary material available at 10.1186/s12920-023-01604-w.

## Introduction

Lung adenocarcinoma (LUAD) is the most common lung cancer subtype, whose overall survival is fewer than 5 years [1]. Despite the innovation of surgery, chemotherapy and targeted therapies brings inspirational therapeutic progress to LUAD patients, the clinical prognosis still remains a gap from our expectation and remarkably varies between different LUAD patients [2, 3]. In the past decades, PD-1/PD-L1-oriented immune checkpoint inhibitors (ICIs) or other immune regulators have provided a potential hope for LUAD patients [4]. However, the overall response rate of ICI is unsatisfying, only few LUAD patients could obtain significantly clinical effects under ICI treatments [5]. Currently, a range of biomarkers have been tried to predict the efficacy of ICI treatment, including TMB, PD-L1, CTLA-4 and the status of immune cells, which still remain to be further confirmed through clinical trials [6]. Moreover, solely indicator cannot precisely and comprehensively predict the efficacy of immunotherapy and stratify appropriate population [5]. Gene signatures, drawing increasing attraction, are served as various biological function patterns with involvement of the expression data of multiple related genes, and can be utilized to predict the prognosis and progression in various types of malignancies [7, 8]. Therefore, a signature which can both predict the prognosis and the response of immune checkpoint inhibitors or chemotherapies in LUAD patients is worthy exploring.

Immunogenic cell death, a type of regulated cell death (RCD), is capable of activating an adaptive immune response and reshape the tumor immune microenvironment. Specifically, some dying tumor cells release multiple danger signals or damage-associated molecular patterns (DAMPs), including high mobility group protein B1 (HMGB1), calreticulin and ATP, which dominantly represent immunogenic features and may contribute to the immunotherapy [6, 9]. Actually, commonly applied therapies, involving personalized chemotherapeutics, radiation therapy and targeted anticancer agents, initiate ICD and subsequently participate in immune responses of killing tumor cells to enhance treatment efficacy [10–12]. Therefore, it is convincing that the confirmation of ICD-based biomarkers is beneficial to stratify patients with different responses to immunotherapy.

In this study, we initially evaluated the expression profiles of ICD-related genes (ICDRGs) and obtained their prognostic value for the LUAD patients. 572 patients with LUAD were divided into two distinct clusters according to ICDRGs expression levels. Patients were subsequently classified into two distinct gene subtypes based on differentially expressed genes (DEGs) analyzed by the comparisons of the two ICD-related clusters. We further developed and validated a novel ICD-related score (ICDRS) followed by comprehensive investigation about the landscape of the prognosis, immune-based features, immunotherapautic responses and sensitivity of target drugs in patients with LUAD. Based on them, our results revealed that the ICDRS is potentially considered as an efficient and valuable biomarker of clinical prognosis and immunotherapeutic efficacy among patients with LUAD.

## Methods

All methods were carried out in accordance with relevant guidelines and regulations.

### Data collection

**Fig. 1:**
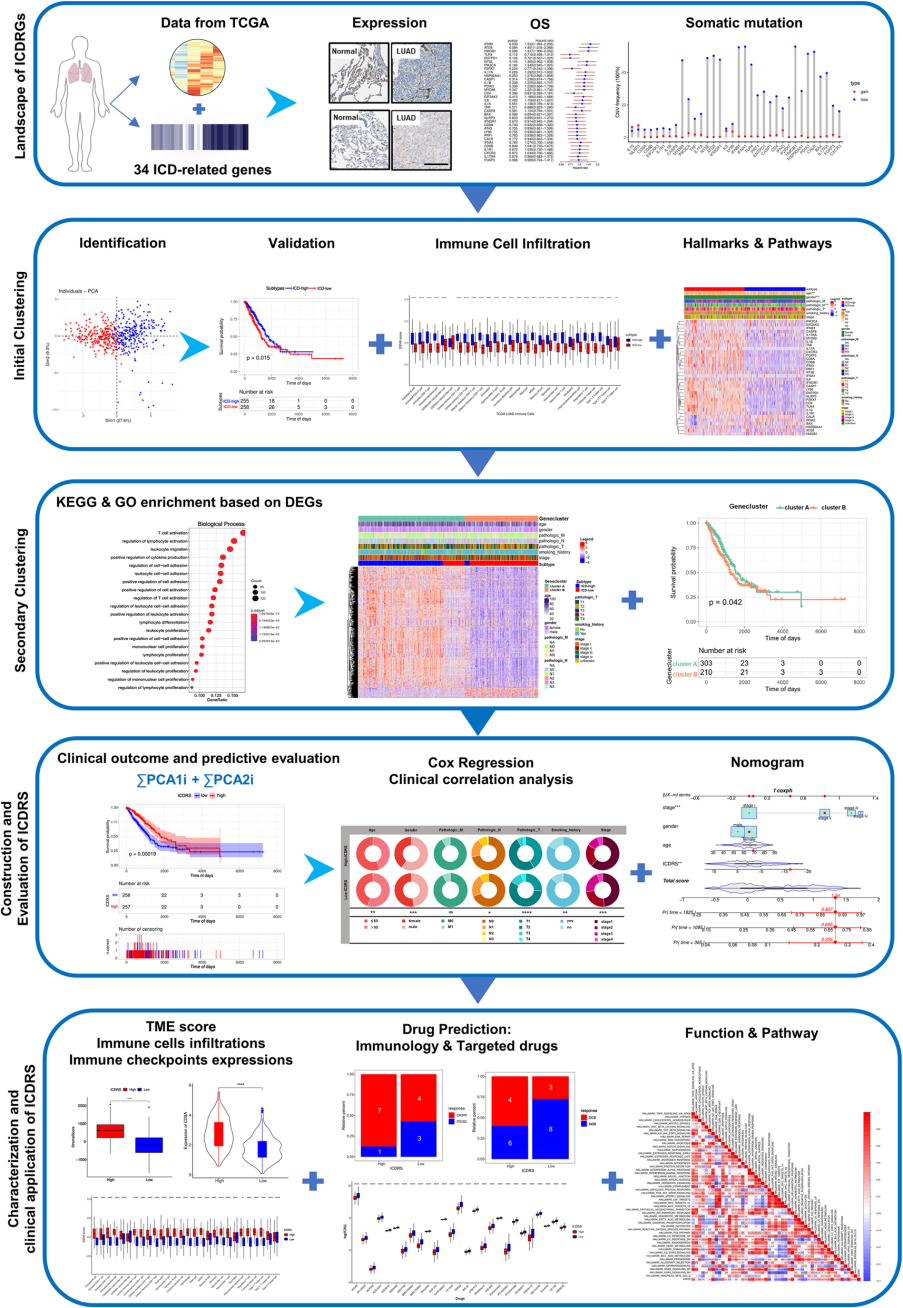
The workflow of identification of the immunogenic cell death-based signature for patients with lung adenocarcinoma

Figure 1 displays our study’s design. The training dataset with RNA-seq transcriptome information and matching clinical data of 513 LUAD and 59 normal samples were downloaded from TCGA-LUAD (https://portal.gdc.cancer.gov/projects/TCGA-LUAD) [13]. The clinical features of the patients are shown in Table S1 in detail. For the following validation, two datasets were retrieved from the Gene Expression Omnibus (GEO; accession number: GSE72094 and GSE26939; https://www.ncbi.nlm.nih.gov/gds) [14]. Besides, a series of ICD-related genes were retrieved from the previous literature and are listed in Supplementary Table S1 [15].The human Gene Annotation Format (Homo_sapiens.GRCh38.99.gtf.gz) were received from Ensembl (http://www.ensembl.org/info/data/ftp/index.html). The correlation of gene expression with immune infiltration level in diverse cancer types was downloaded from IMmune Estimation Resource (TIMER; https://cistrome.shinyapps.io/timer/). Data cleaning was conducted by R software [16]. The immunology cells related genes was retrieved from the previous literature [17]

### Further comparative analysis on ICD related genes (ICDRGs) between LUAD and normal samples

Firstly, the expression of ICD related genes (ICDRGs) was compared between tumor and normal samples from TCGA by Wilcoxon rank-sum test. Subsequently, a univariate Cox analysis was performed about the relation between ICDRGs and overall survival (OS), visualizing as a forest plot. Among them, genes with a significant relativity to prognosis were selected for further analysis. Based on the median expressional value of the genes, LUAD patients in TCGA dataset were divided into high- and low- subgroups. Kaplan–Meier analysis was further used to verify the prognostic value of key ICDRGs with the R package “survival” (v3.2–7) and “survminer” (v0.4.8). The overlapping ICDRGs selected by both differential expression and univariate Cox analysis of survival were further identified. The expression and localization of proteins translated by these selected ICDRGs were represented through immunohistochemical staining images originated from The Human Protein Atlas (HPA) database (https://www.proteinatl as.org/] [18].

### Gene Mutation and Chromosome Distribution Analysis

Somatic mutation information of TCGA-LUAD was summarized from the TCGA data portal (http://tcga-data.nci.nih.gov/tcga/) as the mutation annotation format (MAF) analyzed by the R package “maftools” (v1.0–2) to represent the corresponding gene mutation patterns and frequencies in different groups [19]. The “ggplot2” package was used in R software to describe copy number variation (CNV) downloaded from LUAD. Circos plot was performed with the “RCircos” (v1.2.1) package in R software to obtain visualization about the location of ICD genes in chromosomes [20].

### Consensus clustering analysis of ICDRGs

To determine distinct ICD-related patterns mediated by ICDRGs, a total of 34 ICDRGs was analyzed. An unsupervised clustering was used for sample clustering using the R package “ConsensusClusterPlus” (v1.50.0) [21]. Patients were divided into two ICD related subtypes, high- and low- subtypes, according to the clustering of ICDRGs signature for further analysis. Principal component analysis (PCA) using R packages “factoextra” (v1.0.7) and “FactoMineR” (v2.4) was then performed to determine different subtypes using principal components 1 and 2. Stability evidence was subsequently performed in unsupervised analysis to confirm cluster count and membership. This process was repeated 1,000 times to ensure the stability of clustering. Kaplan‐Meier survival curves were performed in each cluster, and log-rank tests were utilized to compare the overall survival (OS) between subgroups, respectively [22].

### Estimating of functional analysis and tumor immune microenvironment

To investigate the relative functional enrichment between ICD high- and low- subtypes of LUAD, single-sample Gene Set Enrichment Analysis (ssGSEA) algorithm could estimate the degree of substantial variations in the series of genes expressed between the ICD high- and low- subtypes and analyzed biological functions of ICD with an enrichment of 50 cancer hallmark pathways in the MSigDB Collection (MSigDB v7.5.1), the results of which were presented in the form of heatmap. Wilcoxon rank-sum test was utilized to ﻿compare the content of pathways in LUAD ﻿between the ICD high- and low- subtypes.

In order to analyze the distribution of immune cells in distinct ICD-related subtypes, several currently recognized methodologies were utilized to identify the immune infiltration status among samples from TCGA database. Firstly, the Estimation of Stromal and Immune cells in Malignant Tumor tissues using Expression data (ESTIMATE) (v1.0.13) algorithm was applied to compute StromalScore, ImmuneScore and ESTIMATEScore of each sample in LUAD with regard to corresponding gene expression contents of stromal and immune cells via the R package “estimate” [23]. Then, for further calculating the specific proportions of immune cells in each subtype, a set of metagenes, covering nonoverlapping sets of genes representative of 28 specific immune cell subpopulations, was achieved and performed non-parametric and unsupervised Gene Set Variation Analysis (GSVA) analysis with “GSVA” R package [17]. The results were standardized with Function “scale()” in the form of boxplots. Besides, R package “CIBERSORT” (v1.03) that estimates the relative abundance of 22 immune-related cell types, and TIMER algorithms (https://cistrome.shinyapps.io/timer/), a method to quantifying gene expression associations were also conducted, which were shown in supplement figures [24, 25]. Moreover, the expression levels of 45 immune checkpoints retrieved from a research were screened to evaluate the differences between two ICD high- and low- subtypes [26].

### Identification of Differentially Expressed Genes Between ICD-related Subtypes in LUAD

The R package “limma” (v3.42.2) was performed to enrich DEGs between distinct subtypes. Genes with adjusted *P* value < 0.05 and |logFC|> 0.585 were considered statistically significant. Kyoto Encyclopedia of Genes and Genomes (KEGG) pathway (https://www.genome.jp/kegg/) and Gene Ontology (GO) analyses (http://geneontology.org/) were performed to evaluate the biological pathways associated with the DEGs using the R package “clusterProfiler” [27]. Further function analysis of biological processes (BP), molecular functions (MF) and cellular components (CC)) were represented based on GO analyses using R software, ggplot2 package. Pathways with adjusted *P* value < 0.05 and false-discovery rate (FDR) q value < 0.2 were defined as statistically significant.

In terms of genetical aspect, unsupervised clustering and PCA were performed to cluster samples in accordance with the mentioned DEGs. Briefly, DEGs with positive relation was named as ICD gene cluster A, while those with negative relation were defined as ICD gene cluster B, respectively. Corresponding Kaplan–Meier survival analyses were also conducted to evaluate the prognostic values of two gene clusters.

### Construction of ICD-Related Score

To quantify the characteristics of ICD in TCGA-LUAD patients, an algorithm containing ICDRG profiles was constructed and defined as ICD-Related Score (ICDRS). The TCGA-LUAD cohort (*n* = 572) served as the training set. Firstly, univariate Cox proportional hazards regression analysis was performed using the R package “limma” (v3.42.2) on the DEGs between LUAD and normal samples. The significant standard was set as FDR < 0.01. Subsequently, genes with a significant difference in OS were selected for an unsupervised clustering to divided patients into different groups for further analysis, as well as PCA analysis for filtering the main constituents of these genes. With the basement of these analysis, a signature was ultimately constructed. A method with the same core of the gene expression rank index was applied in each patient: ICDRS = ∑PCA1i + ∑PCA2i (i is the expression of prognostic DEGs after screening). The median value of scores was set as the cut-off value of differentiating the high- and low- ICDRS groups. The KM curve was used for estimating this comparison according to prognosis, and log-rank test calculation results with a *P* value < 0.05 were considered statistically significant. Besides, the time-dependent receiver operating characteristic (ROC) curve analysis (containing one-, two-, and three-year survival) was performed to identify the specificity and sensitivity of ICDRS signature utilizing R package “survivalROC”, the effect of which was calculated by the area under the curve (AUC). The datasets of GSE72094 and GSE26939 from GEO were also analyzed to identify the prognostic value of this score. In addition, associations between ICDRS and basically clinical features, including age, gender, TNM stage, clinical stage and smoking history were further analyzed, and Sankey diagrams were performed to visualized the correlation among ICDRS, ICD subtypes and gene clusters.

### Clinical correlation and stratification analyses of the prognostic ICDRS

To evaluate the independence of ICDRS asides from other available clinicopathological features, the samples in TCGA and GEO cohorts (GSE72094 and GSE26939) were conducted to univariate and multivariate analyses. Thereafter, stratified analyses were performed to verify the credibility of ICDRS prediction in distinct subgroups stratified according to age, gender, T stage, N stage, and smoking history. KM analysis was shown utilizing the “survminer” and “survival” packages in R.

### Pathways enrichment and functional analysis of ICDRS

The relationship between the ICDRS and cancer hallmarks was explored and generated a correlation heatmap. The R package “Limma” was used to identify DEGs between two distinct ICDRS groups, with a standard of |log2foldchange|> 0.5 and adjusted *p*-value < 0.05. Then the corresponding expression of DEGs in all samples was selected for GO and KEGG functional annotations analyses with R package “clusterProfiler”. Gene Set Enrichment Analysis (GSEA) was performed to identify hallmark pathways strongly related to the gene signature.

### Characterization of Immune Landscape Between high and low ICDRS groups

To explore the differences of tumor immune microenvironment between high and low ICDRS groups, immune-discriminated analyses were conducted on the basis of gene sets of 28 reported immune cell types of tumor microenvironment with the help of the R package “GSVA”[17]. Differential infiltration analysis was performed and represented by a violin plot. Thereafter, stromal score representative of the stroma sufficiency, immune score symbol of the infiltration of immune cells and estimate score proving tumor purity) derived from ESTIMATE algorithm [23].﻿ Additionally, the correlation of the ICDRS and the expression of two immune checkpoint molecules (CD8A and PD-L1) was also checked.

### Mutation and Prediction of Immunotherapeutic and Chemotherapeutic response

The MAF retrieved from the TCGA database was disposed with the “maftools” R package to identify the somatic mutations of LUAD patients between two score groups. The Tumor Immune Dysfunction and Exclusion (TIDE) algorithm (http://tide.dfci.harvard.edu/), was conducted to predict the clinical efficiency of immunotherapy referring to the gene expression data of TCGA-LUAD. Spearman correlation analysis was carried out between ICDRS and the TIDE scores [26, 28, 29]. GSE126044 and GSE136961 datasets were also used to test the ICDRS for immunotherapy response’s prediction abilities.

To explore differences in the therapeutic effects of targeted drugs in the two groups, the semi-inhibitory concentration (IC50) values of targeted drugs was calculated using R package “pRRophetic” (v 0.5) [30]. IC50 is representative for a concentration of the compound capable of restraining a specifically biological or biochemical processes, which means that the lower the IC50, the more sensitive to the specific compound. A total of 135 drugs were calculated, and the sensitivity of the drugs was analyzed by unpaired t-tests. *P* < 0.05 was considered as the threshold for significance.

### Cell culture

A type of human lung carcinoma A549, human lung fibroblasts HLF and Lewis lung carcinoma (LLC) were purchased from the Type Culture Collection of the Chinese Academy of Sciences. A549 and HLF were cultured in DMEM medium containing 10% fetal bovine serum (FBS) while LLC cells were cultured in RPMI 1640 medium, both of which were added with penicillin (100 IU/ml) and streptomycin (100 μ g/ml). All cells were cultured in a cell incubator at 37˚C atmosphere with 5% CO_2_.

### Western blot analysis

Cells were washed with PBS and lysed in RIPA buffer (Solarbio, Beijing, China) about 30 min. Thereafter, the mixture was collected and centrifuged for 15 min at 12,000 rpm, and the supernatant was measured by BCA protein assay kit (Biosharp, China) to quantitate the protein content of samples. The cell lysates mixed with protein loading buffer and loaded in SDS–polyacrylamide gels then transferred to nitrocellulose filter membrane (ThermoFisher, USA). The membrane was first blocked with 5% non-fat dry milk for 1 h, then incubated with TLR4 primary antibody (MA5-16,216, Invitrogen) (1:500 dilutions) overnight and anti-β-actin (ab8266, Abcam) (1:2000 dilutions) 4 h as a loading control. The blot was then cultured with specific secondary antibodies (1:10,000 dilutions). All proteins were visualized with a western blotting substrate.

### Quantitative Real-Time PCR (qRT-PCR) assay

Total RNA was extracted from collected lung tumor tissues using Trizol (Invitrogen) and reverse transcribed into cDNA by using HifairTM II 1st Strand cDNA Synthesis SuperMix Kit (11123ES60, YEASEN, China) according to the manufacturer’s protocol. Quantitative PCR reactions were performed by a BIO-RAD CFX96™ (Bio-rad, USA) with the supplement of SYBR Green (11201ES08*, YEASEN, Shanghai, China) and the amplification of the desired products was observed and recorded using CFX96TM Real-Time PCR Detection System. Reactions were performed in triplicate. The fold difference in transcripts was calculated using the ΔΔCt method with GAPDH as a control [31]. All the above primers were synthesized by Biomed Company (Beijing, China). The gene sequences used were as follows:

- TLR4-F: 5’-ATGGCATGGCTTACACCACC-3’;
- TLR4-R: 5’-GAGGCCAATTTTGTCTCCACA-3’;
- GAPDH-F: 5’-AGGTCGGTGTGAACGGATTTG-3’;
- GAPDH-R: 5’-TGTAGACCATGTAGTTGAGGTCA-3’.

### Assays for CCK8 Cell Proliferation

The A549 cells were seeded onto 96-well plates (3 × 10^3^ cells per well plate) and treated with DMSO or LPS (10 μg/mL) for 5 days. The medium was replaced every day during the course of the experiment. CCK8 was used to monitor cell proliferation following the manufacturer’s recommendations (Dojindo biochemical techniques).

### Tumor xenografts animal experiments

Animal studies were approved by the Research Ethics Committee of the Chinese PLA General hospital. For the LLC cell subcutaneous xenograft model, healthy C57BL/6 J mice (male, 6–8 weeks old, and weighing 18–22 g) were purchased from Beijing SPF Biotechnology Co., Ltd (Beijing, China, animal license #: SCXK Beijing 2019–0010), and performed in line with guidelines formulated by NIH Guide about laboratory animals. All animals were resident in an appropriate place, including 12 light/12 dark cycle, temperature 25˚C, and humidity 40–60%.

Briefly, under anesthesia situation, mice were implanted subcutaneously with LLC cells (4 × 10^5^) dissolved in 0.1 ml of phosphate-buffered saline (PBS) into the right dorsal of each mouse to establish tumor-bearing mouse models. When the tumor volume reached about 100 mm^3^, C57BL/6 J mice were randomly divided into 2 groups (*n* = 5 for each group): the control group and LPS group. LPS (5 mg/kg; Escherichia coliserotype O111:B4, Sigma, USA) was diluted and dissolved using saline intratumorally injected into mice on that day, seven and fourteen days later of dividing group. Mice in control group were injected intratumorally with phosphate-buffered saline (PBS) as control. The mice’s tumor volume was measured every week. Not until being had mice been anesthetized with persistent isoflurane on day 21, mice were executed with euthanasia and their tumor tissues were collected. Mean tumor masses in size were compared using unpaired t tests between LPS-treated mice and controls.

An additional 5 mice in each group were performed for the survival study. After finishing the 3-week experimental period under LPS treatment as mentioned above, all mice were monitored until death during the survival study.

### Hematoxylin and Eosin (HE) staining and immunohistochemistry

On day 21, mice were anesthetized to obtain tumor samples, part of which were isolated and fixed in 4% paraformaldehyde buffer for HE staining. The other sections of the tumor tissues were fixed by formalin and embedded in paraffin for immunohistochemical staining. During the process, primary antibodies were incubated overnight at 4 °C [anti-Ki-67 (1:200, Abcam, ab16667)], with the following of specific secondary antibodies. Images were obtained at 200 × magnification using an Olympus BX43 microscope. The immunoreactive score (IRS) system was used to evaluate the expressional intensity of genes. The percentage of positive cells lowed than 10% was scored as 0. 10–50%, 51–80% and 81–100% of those was scored as 2, 3 and 4, respectively. The staining intensity was scored as follows: no color reaction: 0; mild reaction: 1; moderate reaction: 2; and intense reaction: 3. Final IRS scores of immunohistochemistry = (scores of staining intensity) × (scores of percentage of positive cells) [31]. Slides were assessed by 2 pathologists. Values were expressed as mean ± SD.

## Results

### Landscape of ICD genes and OS between normal and malignant LUAD samples

The ICDRGs were proposed by massive literature, which has been previously summed up by Abhishek et al. (13). To fully understand the expression pattern of ICDRGs involved in tumorigenesis, 578 patients (59 normal samples and 519 malignant samples) from TCGA-LUAD were introduced in our study for future studies. A summary of information on the 578 LUAD patients was displayed in Table S1.

**Fig. 2:**
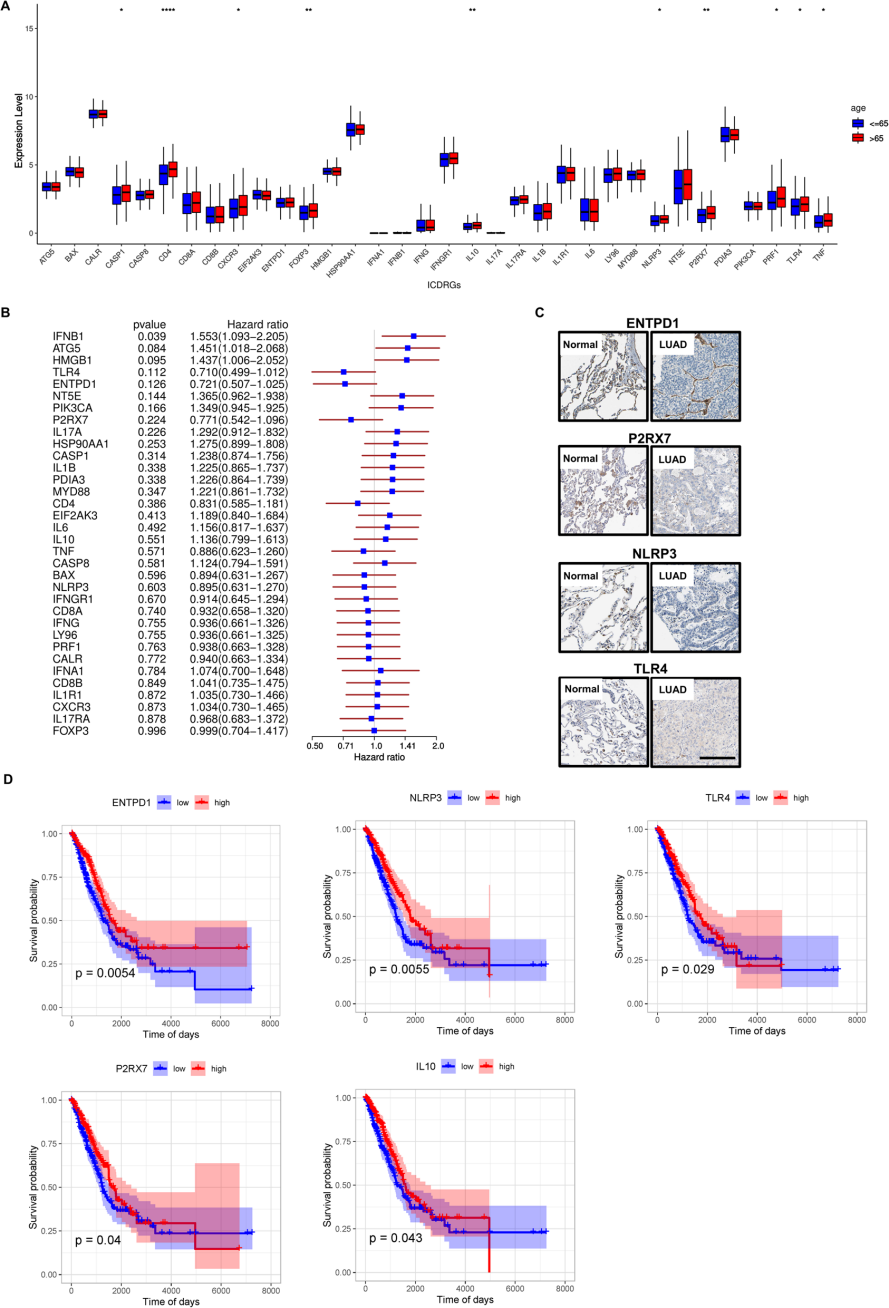
Expressional and prognosis analysis of ICDRGs in normal and lung adenocarcinoma tissues. **A** Expression of ICDRGs in normal and lung adenocarcinoma tissues. The statistical difference was compared by the Wilcoxon test; (**B**) Forest plot presenting the results of the Univariate COX regression analysis between gene expression and OS for the 34 ICDRGs; (**C**) Protein expression level and localization of ICDRGs with significant prognosis value in normal bronchus tissue and lung adenocarcinoma specimens measured by IHC staining based on Human Protein Atlas (bars = 200 μm); (**D**) Kaplan–Meier curves of ICDRGs with significant prognosis value in overall survival of LUAD patients. (*P < 0.05, **P < 0.01, ***P < 0.001, ****P < 0.0001)

We first analyzed the expression patterns of ICD genes in normal and LUAD samples. The results suggested that 29 out of 34 ICD-related genes showed significant differences between normal and malignant samples, where certain genes, for example CALR and PDIA3, displayed higher expression, while others (such as IFNGR1, P2RX7, NLRP3 and TLR4) displayed lower expression in tumor samples (Fig. 2A). Furthermore, the heterogeneous expression of the ICDRGs between normal and cancer tissues indicated that the ICDRG expression differences played a crucial part in the development and occurrence of LUAD.

Based on the TCGA dataset, univariate Cox regression was performed to study the predictive value of key ICDRGs about survival. Hence, of note, ENTPD1, NLRP3, TLR4, P2RX7 and IL10 can be predicted as protective genes of LUAD with HR (hazard ratio) < 1 (*P* < 0.05) (Fig. 2B). Then Kaplan–Meier survival curve analysis of the five genes was performed among two expression groups (high and low). We found that they all were significantly associated with OS in LUAD (Fig. 2D). Interestingly, except for IL-10, the other four could not only predicted an excellent prognosis, but also had relatively low expression in tumor tissues. Moreover, to verify the protein expression of the four genes, including ENTPD1, NLRP3, TLR4, P2RX7, the HPA database was applied for inspection of the expression of the proteins deprived from them in LUAD tumor tissues and normal tissues. Concurrent with what we found in TCGA, all four proteins were significantly downregulated in LUAD tissues compared with normal tissues, and mainly expressed on the cell membrane and cytoplasm in the tumor cells, indicating that the model proteins expression levels shared the similar tendency to that of the corresponding model genes (Fig. 2C).

### Genetic and transcriptional alterations of ICDRGs in LUAD

**Fig. 3:**
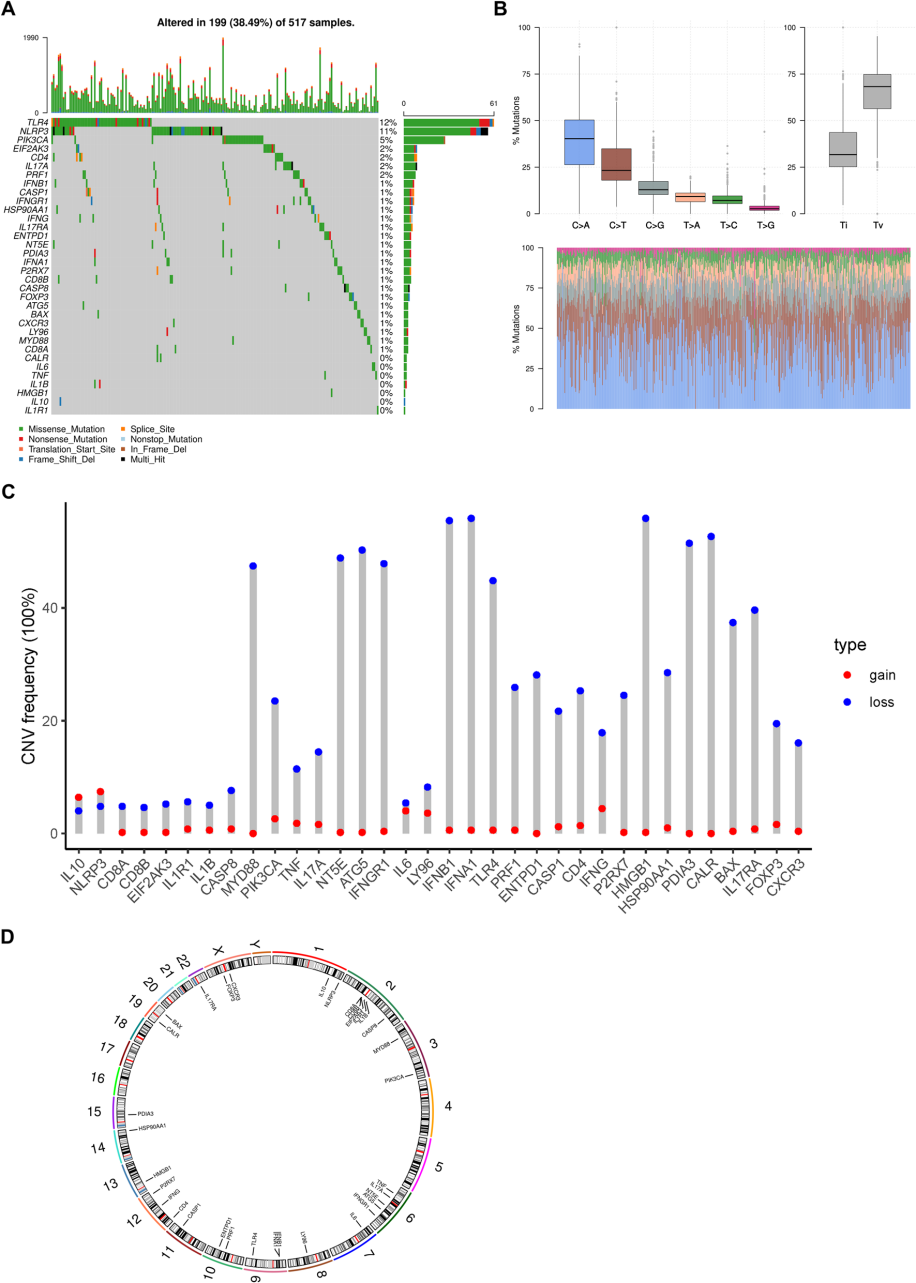
Genetic and transcriptional alterations of ICDRGs in LUAD. **A** The waterfall plot of somatic mutation features in 517 patients with LUAD from TCGA-LUAD cohort. The column represents the patients; (**B**) The boxplot showed variant types of LUAD samples from TCGA-LUAD cohort. The different colors below the figure represent the proportion of variant types. The right stacked barplot demonstrated the proportion of each variant type. Only molecules having mutations, namely mutant frequency > 0%, were illustrated in the waterfall plot; (**C**) The CNV frequency of ICDRGs in TCGA cohort. The height of the column represents the alteration frequency. The blue dot represents deletion frequency. The red dot represents amplification frequency; (**D**) The location of CNV alteration of ICDRGs on 23 chromosomes

Summary analysis of the incidence of somatic mutations in these 34 ICDRGs in the LUAD cohort was shown (Fig. 3A). Of the 517 LUAD samples, 199 (38.49%) had mutations in the ICDRGs. Among them, TLR4 had the highest mutation frequency (12%), followed by NLRP3(11%) and PIK3CA (5%) (Fig. 3A). The mutation type with C to A had the highest proportion in LUAD samples (Fig. 3B).

Next, we calculated and demonstrated somatic copy number alterations in these ICDRGs, resulting discovering that most ICDRGs with high CNA frequency trended to be co-deletion rather than co-amplification (Fig. 3C). It was also showed the locations of the CNV alterations in the ICDRGs on their respective chromosomes (Fig. 3D). Furthermore, we performed a comparation between the mRNA expression levels of LUAD and normal tissues and found a similarly expressional tendency of most ICDRGs as the incidence of CNV alteration. ICDRGs with CNV loss, such as HMGB1, IFNGR1, TLR4 and PIK3CA, were expressed at lower levels in LUAD samples compared to those in normal samples, suggesting that CNV might regulate the mRNA expression of ICDRGs.

### The Initial Clustering: Identification of two ICD subtypes in LUAD Based on the Expression Pattern of ICDRGs

**Fig. 4:**
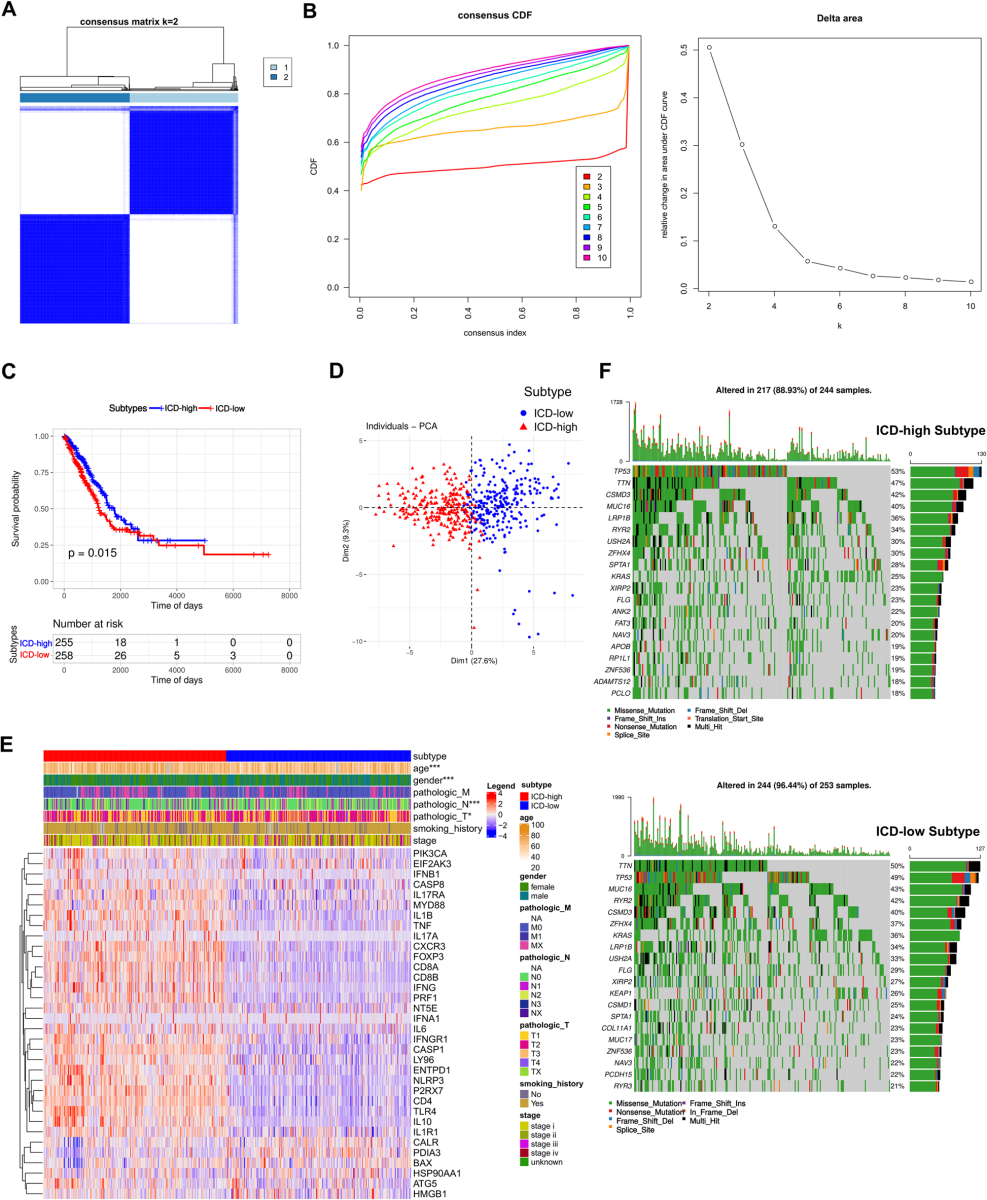
Identification of ICD-associated subtypes by consensus clustering and comparison of somatic mutations between different ICD subtypes. **A** The consensus matrixes for all LUAD samples displayed the clustering stability with 1,000 iterations. All samples were clustered into an appropriate number of subtypes (k = 2); (**B**) Delta area curve of consensus clustering indicates the relative change in area under the cumulative distribution function (CDF) curve for k = 2 to 10; (**C**) Kaplan–Meier curves showed the overall survival difference between ICD-high and low subtypes (p = 0.015); (**D**) PCA analysis showing a remarkable difference in transcriptomes between the two subtypes; (**E**) Differences in clinicopathologic features and expression levels of ICDRGs between the two distinct subtypes. Red represents high expression and blue represents low expression; (**F**) Oncoprint visualization of the top 20 most frequently mutated genes in ICD high- (up part) and low- (down part) subtypes. (*P < 0.05, **P < 0.01, ***P < 0.001, ****P < 0.0001)

For further study on the expression features of ICDRGs in LUAD, patients with LUAD were classified based on different ICDRG expression patterns using consensus clustering algorithm. The outcome showed that k = 2 seemed to be a suitable method for dividing the cohort into cluster 1 (*n* = 244) and 2 (*n* = 253) (Fig. 4A, B). Noticeable differences in the ICD transcription profiles between the two clusters can be concluded by PCA analysis. Generally, clusters 1 indicated high I expression levels of ICDRGs named as ICD-high subtype while clusters 2 presented low expression levels defined as ICD-low subtype (Fig. 4D, E). Thus, we defined cluster 1 as ICD-high subtype, and cluster 2 as ICD-low subtype. The survival analysis represented that a longer OS in patients with ICD-high subtype (*p* = 0.015; Fig. 4C). Furthermore, significant differences in ICDRG expression and clinicopathological characteristics were revealed (Fig. 4E). As shown in Fig. 4F, ICD-high subtype was preferentially related to female, older age, and lower N stage compared to those in ICD-low subtype. These indicate that ICDRGs may affect tumor development by some of the potential mechanisms.

In addition, we estimated the differences in somatic variation between two subtypes. The top 20 highest mutation frequency of driver genes were selected to plot as a waterfall diagram. Results were revealed that patients in ICD-low subtypes had significantly higher frequencies of most genes including TNN, MUC16 and RYR2 compared to those in patients in ICD-high subtypes. However, the opposite results were observed in terms of the mutation frequencies of TP53, LRP1B and CSMD3 (Fig. 4F).

### The immune cell infiltration characteristics and biological behaviors in distinct ICD subtypes

**Fig. 5:**
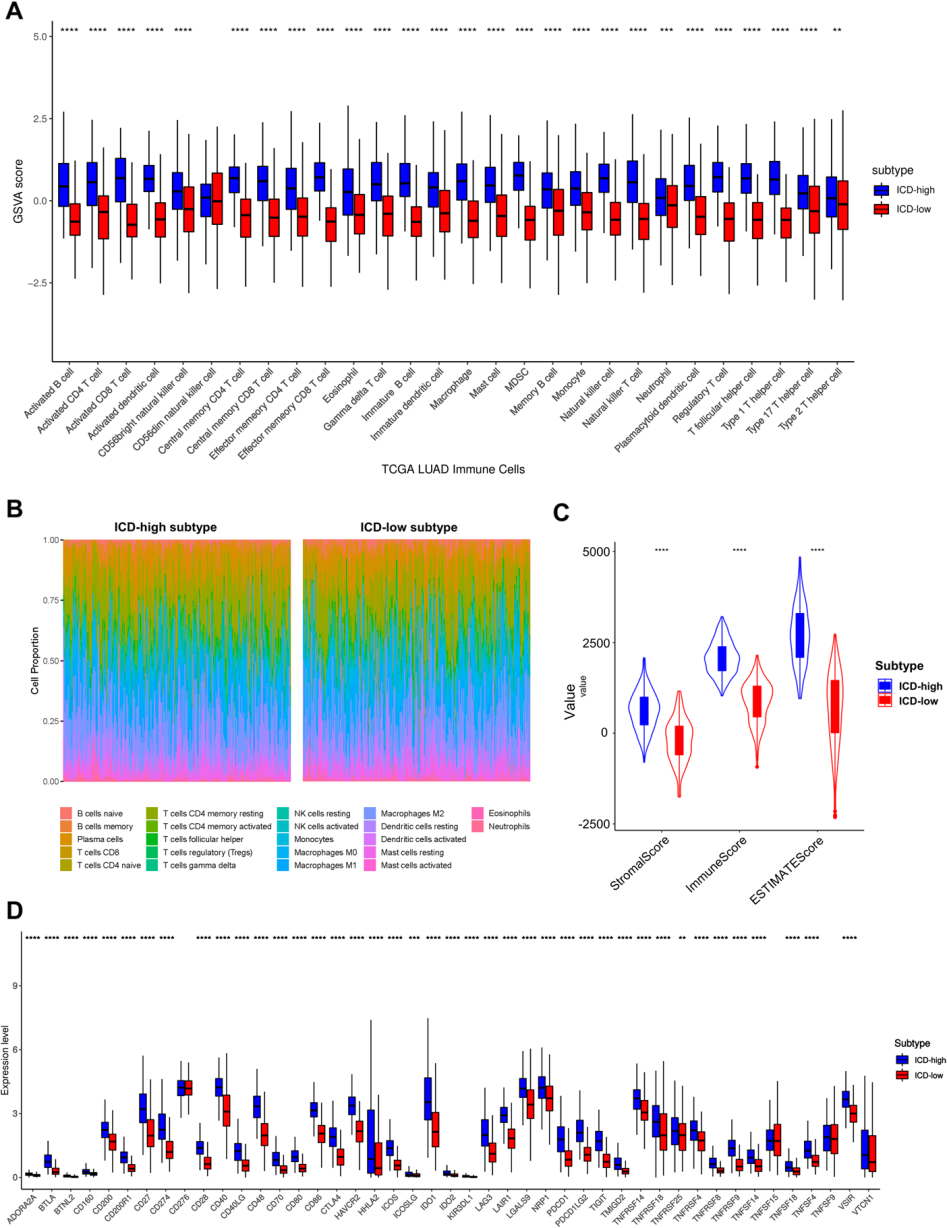
Immune landscape of ICD-high and ICD-low subtypes. **A** The distribution of 28 immune cell subsets infiltration between two subtypes using the ESTIMATE algorithm; (**B**) Relative proportion of immune infiltration in ICD-high and ICD-low subtypes using the CIBERSORT algorithm.; (**C**) Violin plots show the median, and quartile estimations for each immune score; (**D**) Box plots present differential expression of multiple immune checkpoints between ICD-high and ICD-low subtypes. (*P < 0.05, **P < 0.01, ***P < 0.001, ****P < 0.0001)

Increasing evidence demonstrates that ICD exert remarkable effects on specific antitumor immune responses. The analysis of the immune cell infiltration showed that the ICD-high subtype was featured with high activated B cells, activated CD4 T cells, activated CD8 T cells, natural killer T cells and follicular helper T cells, but several immunosuppressive cells, such as eosinophil, myeloid-derived suppressor cells (MDSCs), macrophages, mast cells and regulatory T cells (Tregs) were highly involvement in it (Fig. 5A). These results were somewhat interesting. The CIBERSORT algorithm and TIMER were additionally utilized to evaluate different percentages of immune cells. In detail, ICD-high subtype displayed remarkable high percentages of CD8 T cell, activated CD4 T cell memory, activated CD4 T cell memory and macrophage M1 (Fig. 5B and S3A, B). For the TME score, as shown in Fig. 5C, samples in ICD-high subtype also exhibited significantly higher estimate scores, stromal scores and immune scores, compared with those of ICD-low subtype.

Subsequently, we explored the potential correlation between distinct ICD subtypes and immunotherapy responses, which to some extents could refer to the expressional level of immune checkpoints. The expression levels of PD-1, PD-L1, CTLA4, LAG3 and TIGIT in ICD-high subtype were significantly higher than those in ICD-low subtype according to the Wilcoxon test (*P* < 0.05), suggesting that the ICD-high subtype was more likely to strongly response to immunotherapies (Fig. 5D).

**Fig. 6:**
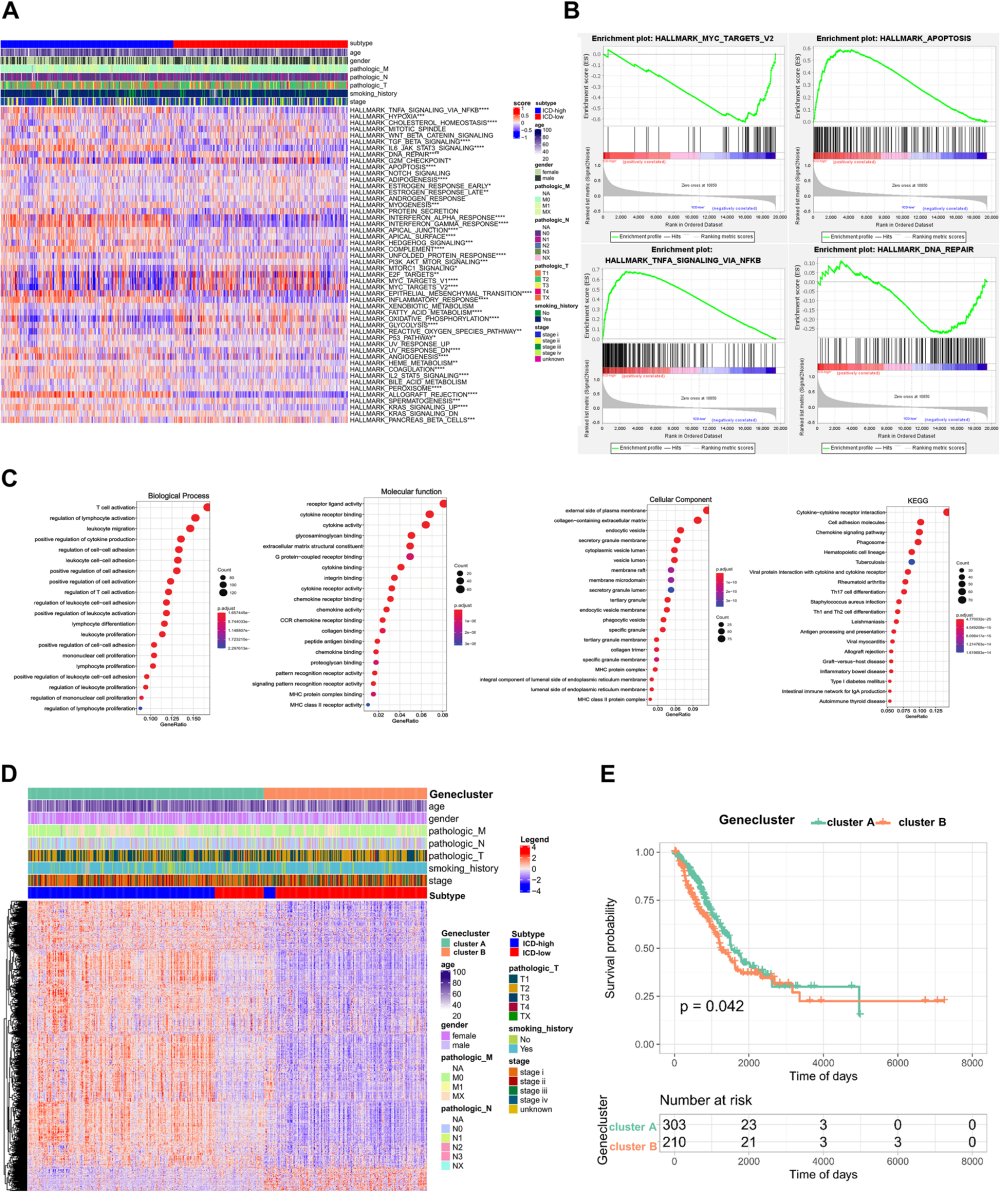
Biological analysis of two ICD-related subtypes and identification of two gene clusters based on DEGs derived from two ICD-related subtypes. **A** The heatmap demonstrates the enrichment of biological pathways. Red and blue represent activated and inhibited pathways, respectively; **B** GSEA analysis determines the underlying signal pathways between ICD-high and ICD-low subtypes; (**C**) Dots plot presents the GO and KEGG signaling pathway enrichment analysis based on DEGs between ICD-high and ICD-low subtypes. The size of the dot represents gene count, and the color of the dot represents – log 10 (p. adjust-value); (**D**) Differences in clinicopathologic features and DEG expression between the two distinct gene clusters. Red represents high expression and blue represents low expression; (**E**) Kaplan–Meier curves estimate the survival differences between two ICD subtype-related DEG clusters. (*p < 0.05, **p < 0.01 and ***p < 0.001)

To investigate the differences between ICD-high and low subtypes in biological processes, ssGSEA algorithm was performed with the hallmark gene set (c2.cp.kegg. v7.2) derived from the MSigDB database. The results showed that 41 out of 50 hallmarks were significantly different between two subtypes, including the TGF-β signaling pathway, apoptosis signaling pathway, epithelial mesenchymal transition signaling pathway and hypoxia signaling pathway. (Fig. 6A, B).

### The secondary clustering: identification of two gene clusters based on differentially expressed genes (DEGs)

As the ICD high subtype indicated with advantageous clinical outcomes while ICD low subtype presented the opposite results, we further identified 879 DEGs related to ICD phenotype and performed functional enrichment analysis (Table S3) in order to explore respective biological characteristics of each ICD pattern. It was presented that the most significant terms enriched by GO enrichment analysis were the biological process (BP) of T cell activation and regulation of lymphocyte activation, cellular component (CC) of external side of plasma membrane and collagen-containing extracellular matrix, and molecular function (MF) of receptor ligand activity and cytokine receptor binding (Fig. 6C). KEGG analysis suggested that they participated mostly in the pathway of cytokine-cytokine receptor interaction and cell adhesion molecules, suggesting that ICD poses significant effects in TME via influencing many immune-related biological processes (Fig. 6C).

To further confirm our analysis, an unsupervised cluster analysis based on the 879 DEGs was performed. Subsequently, patients were re-stratified into two ICD subtype-related DEG clusters (gene clusters A and B). and patients in gene cluster A (*p* < 0.05) (Fig. 6E). In consistent with previous analysis, most patients in gene cluster A belonged to ICD-high subtype with higher DEG expression and more favorable prognosis, while those in gene cluster B with relatively poor prognosis belonged to ICD-low subtype (Fig. 6D).

### Construction and validation of the prognostic ICD-Related Score (ICDRS)

**Fig. 7:**
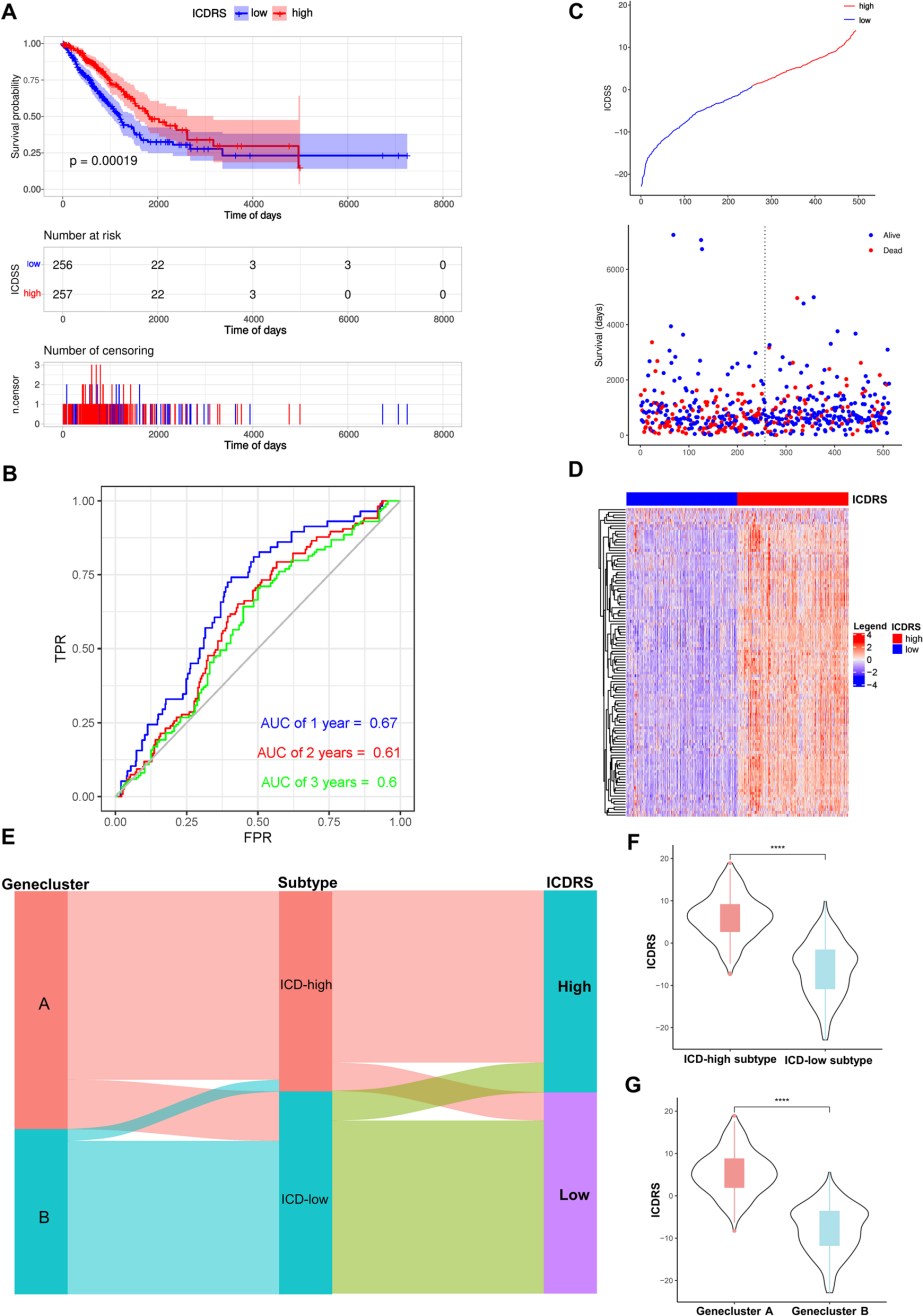
Identification of the ICD-related Score (ICDRS) and investigation of its prognostic value in lung adenocarcinoma (LUAD). **A**, **B** Kaplan–Meier curves and time-dependent receiver operating characteristic (ROC) curves of the prognostic ICDRS model in TCGA. The association between the ICDRS and the survival of patients was investigated using Cox regression and log-rank methods; (**C**) ICDRS scores distribution and survival status of LUAD patients in high-and low- ICDRS groups; (**D**) The heat map depicted the expression of DEGs in high and low ICDRS groups. Heat map colors indicate relative DEGs expression. Levels; (**E**) The Sankey diagram showed the distribution of patients with ICD-related subtypes, gene clusters and ICDRS; (**F**) Differences in ICDRS between 2 ICD-related subtypes with the Wilcoxon test; (**G**) Differences in ICDRS between 2 gene clusters with the Wilcoxon test. (*P < 0.05, **P < 0.01, ***P < 0.001, ****P < 0.0001)

Given the heterogeneity and complexity of individual ICD patterns, we used PCA to quantify the ICD patterns by analyzing DEGs between LUAD and normal samples, and defined the results as ICDRS. DEGs in detail were shown in Table S4. Patients with a ICDRS lower than the median score were stratified into the low score group (*n* = 256), whiles those with a ICDRS higher than the median score were classified into the high score group (*n* = 257). The distribution diagram of ICDRS revealed different survival time in two groups, that is to say, the better patients scored in the ICDRS, the longer they survived (Fig. 7A, C and D). With the basement of the ROC analysis, AUC of one-year, two-year and three-year OS is 0.67, 0.6 and 0.61, respectively, calculated with ICDRS (Fig. 7B). These results could strongly present the promising prognostic value of ICDRS.

To explore the prognostic presentation of the ICDRS, ICDRS was validated across two external cohorts (GSE72094 and GSE26939) (Figures S1A-H). Likewise, patients were also classified into low- or high-score groups. The ICDRS, prognosis and PCA analysis all indicated distinct directions of the low- and high-score groups, respectively (Figures S1B, F). Overall survival analysis also provided similar evidence that patients in high ICDRS group were more likely to possess better prognosis (log-rank; *p* < 0.001; Figure S1A, E). Analysis of the 1-, 2-, and 3-year prognostic prediction revealed that the ICDRS was capable of prognosis prediction for LUAD patients due to relatively high AUC values (Figure S1C, G).

The correlation of ICDRS, ICD subtypes and gene clusters was displayed by the Sankey plot. The high ICDRS group accounts for a higher proportion of the patients in ICD-high subtype or gene cluster A, indicating patients in high ICDRS group with a good outcome and demonstrating the consistency of the predictive effectiveness (Fig. 7E). Moreover, patients in ICD-high subtype or gene cluster A showed a higher ICDRS. Besides, the value of ICDRS was computed in different groups based on twice clustering. The median value of ICDRS in gene cluster A was significantly higher than that in gene clusters B (*p* < 0.0001) (Fig. 7F, G). Therefore, this quantified ICD-related signature could be served as an indicator to predict prognosis of patients with LUAD.

### Clinical correlation analysis and a clinical nomogram establishment of ICDRS

**Fig. 8:**
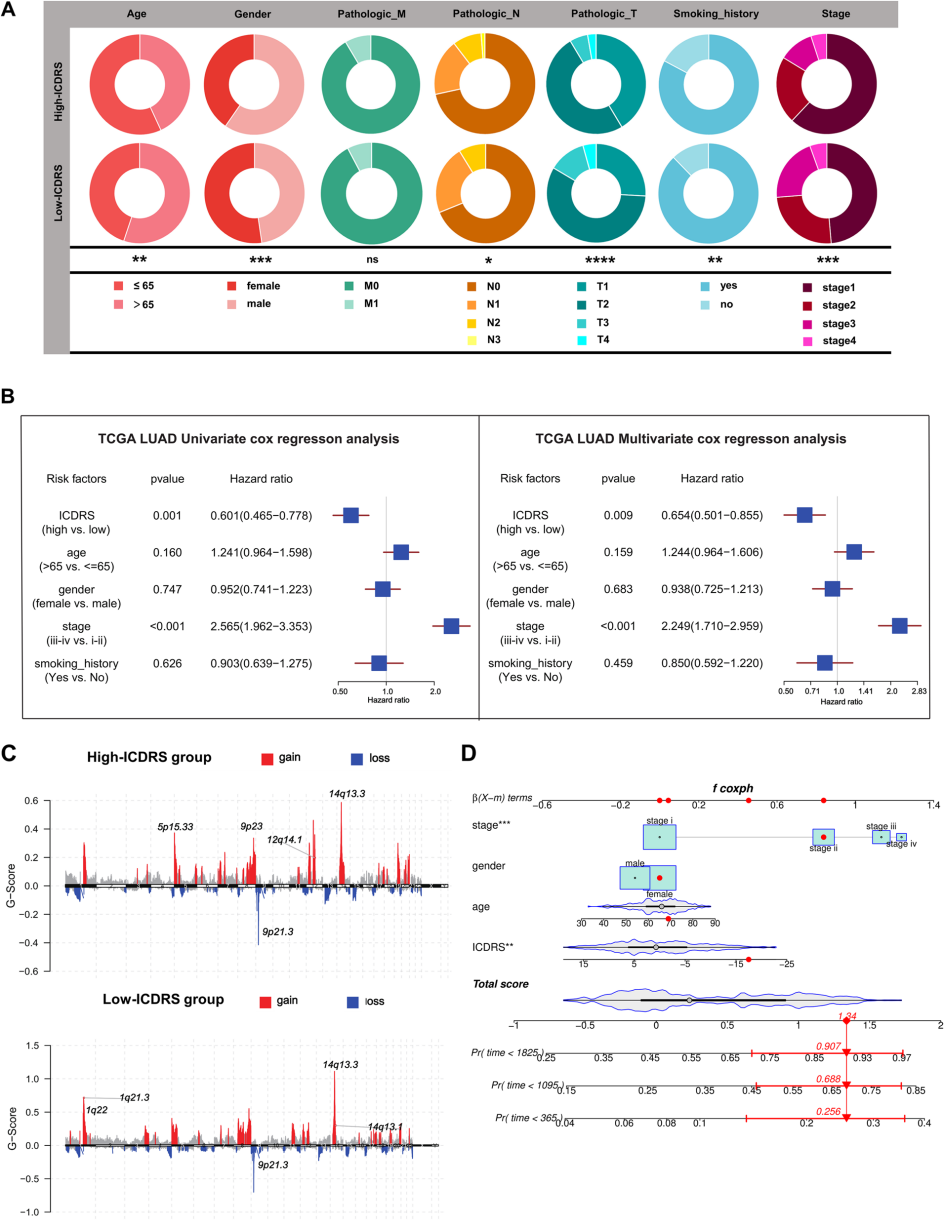
Clinical correlation of ICDRS and genomic alteration. **A** The pie chart showed variations of clinicopathologic characteristics of lung adenocarcinoma between the high-and low-ICDRS groups by Fisher’s exact test; (**B**) A univariate (left part) and multivariate (right part) Cox model showed the independent prognostic values of age, gender, pathologic stage, smoking history and the ICDRS shown by a forest plot. The length of the horizontal line represents the 95% CI for each group. The vertical dotted line represents HR = 1. HR < 1.0 indicates that an elevated ICDRS is a favorable prognostic biomarker; (**C**) CNV overview in high- and low-ICDRS groups, including the logistic score and mutation frequency corresponding to different CNVs; (**D**) Nomogram for both clinic-pathological factors and ICDRS to predict the 1-, 3- and 5-year RFS of LUAD patients. (*P < 0.05, **P < 0.01, ***P < 0.001, ****P < 0.0001)

To explore the effects of the ICDRS on clinical characteristics, we analyzed the correlation between ICDRS and various clinical characteristics (age at diagnosis, sex, Tumor size, lymph node status, TNM stage, smoking habits). We observed that ICDRS was significantly lower in male patients with larger tumor size, lymph node metastasis, advanced TNM stage, smoking habit, and age below 65(*p* < 0.0001; Fig. 8A). Since low ICDRS was significantly associated with advanced lung cancers and in LUAD, we attempted to verify the independence of ICDRS for prognostic prediction in LUAD patients. In addition to ICDRS, several clinical features including age, gender, TNM stage and smoking history were also enlisted as covariates for univariate and multivariate Cox regression analyses. The results ultimately represented that TNM stage and ICDRS were indeed served as independent factors predicting the prognosis of LUAD patients in the TCGA cohort (Fig. 8B), GSE26939 (Fig. S2L) and GSE72094 (Figure S2M).

Thereafter, analysis for further stratification were conducted to estimate whether the ICDRS blocked its predictive capacity in different subgroups, particularly age (≤ 65 and > 65 years), sex (female and male), T stage (T1 and T2-T4), and smoking history (yes and no). As shown in Figure S2A-J, significantly higher OS in patients with high-ICDRS group than those in patients with low-ICDRS group for age (*p* < 0.05), sex (*p* = 0.028 in women and *p* < 0.01 in men), T stage (*p* < 0.05) and smoking history (*p* = 0.0036 for yes and *p* < 0.01 for no).

Furthermore, the distribution variations of the somatic mutations were analyzed between two ICDRS groups in the TCGA-LUAD cohort. Somatic mutation analysis revealed that patients with lower ICDRS had higher mutation frequency of TTN (43% vs. 54%), MUC16 (38% vs. 45%), CSMD3 (37% vs. 44%) and RYR2 (34% vs. 42%) compared with those with higher ICDRS (Fig. S3F; Table S5). Additionally, the copy number alterations (CNAs) landscapes of both the high- and low-score groups were also shown (Fig. 8C; Table S6). Patients in low ICDRS group had higher amplification of LCE3C (1q21.3) and BAZIA (14q13.1), and those in low ICDRS group had higher levels of TERT (15p15.33) and SHMT2 (12q14.1) amplification.

To increase the clinical applicability and generalizability of the ICD-related signature, a prognostic nomogram which simultaneously depends on the score status and common clinical characteristics was established. It could use an algorithm and realize quantitative analysis, which could effectively predict the possible survival time of LUAD patients. Each common clinical characteristic, including age, gender, clinical stage and ICDRS, was applied to calculate the individual sample’s score, respectively, based on which can estimate one-, three- and five-year survival probabilities (Fig. 8D). Furthermore, ROC analysis was also used to verify the nomogram’s predictive accuracy. As shown in Figure S2K, the predicted AUC values of the OS nomogram were better than those of other predictors, suggesting that the nomogram had outstanding performance in predicting survival of LUAD patients. Therefore, there is reason to believe that the nomogram with accuracy and reliability could predict the survival of patients with LUAD.

### Biological pathways and functional enrichment analysis of two ICDRS groups

**Fig. 9:**
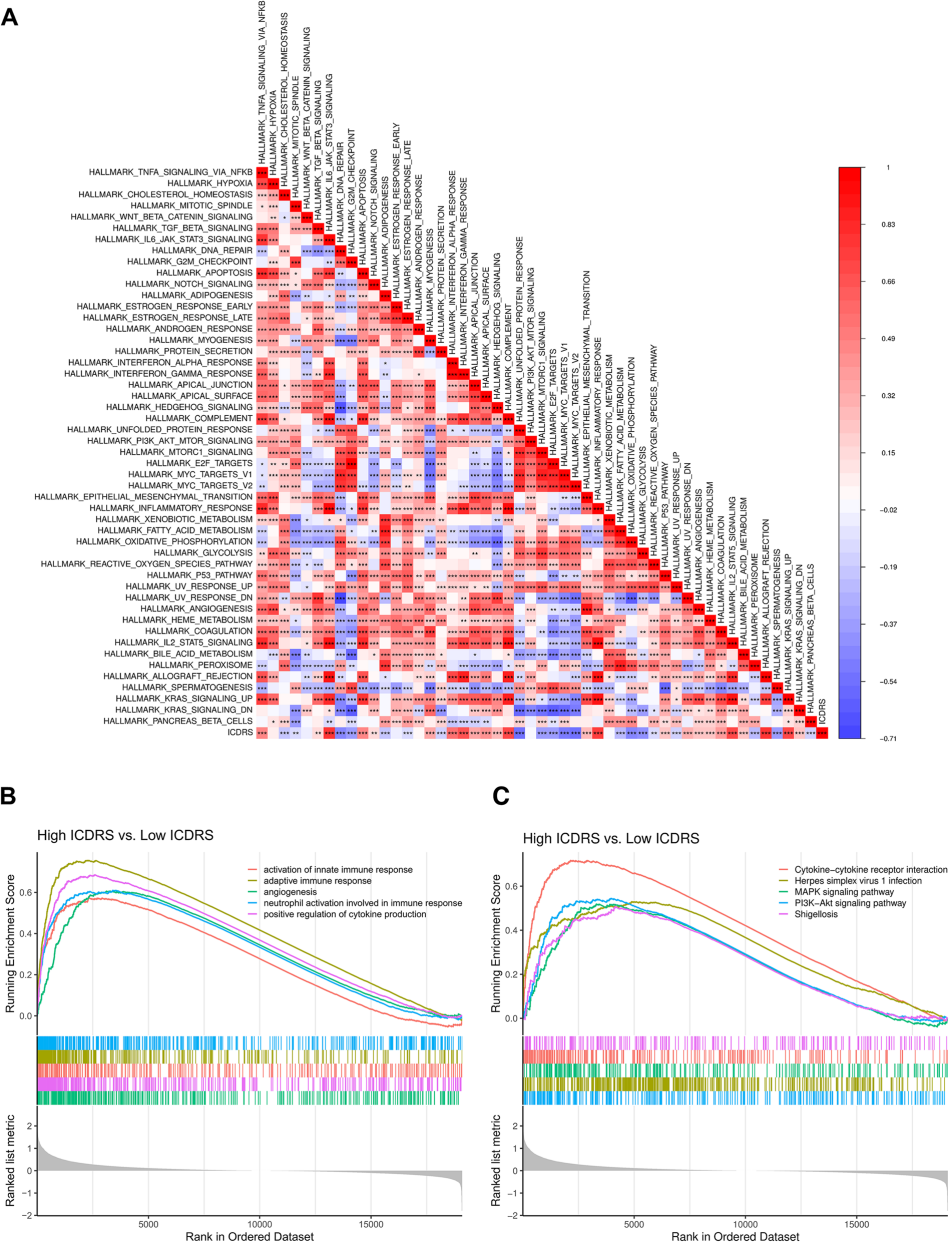
The correlation between the ICDRS and Hallmark pathway activity. **A** The correlation heat map visualized the universal landscape of hallmarks related to ICDRS. The correlation coefficient decreased in size from red to blue. (B,C) GO- (**B**) and KEGG- (**C**) related GSEA showed immune-associated pathways were significantly enriched in the high ICDRS groups. (*P < 0.05, **P < 0.01, ***P < 0.001, ****P < 0.0001)

To investigate the underlying mechanisms that contribute to the different results stratified by ICDRS, we performed KEGG pathway, GSEA, and GO analysis. The relationship between ICDRS and enriched hallmark pathways was demonstrated that abundant pro-oncogenic pathways, such as MYC signal pathway, as well as cell cycle processes pathways, such as DNA repairment and G2M checkpoint pathways were negatively linked to ICDRS, while some tumor suppression pathways, including TNF-alpha signaling were positively associated with ICDRS (Fig. 9A). Meanwhile, the GSEA showed that the gene sets involved in activation of adaptive immune response, innate immune response and positive regulation of cytokine production were gathered together in high-ICDRS patients (Fig. 9B). The GO analysis further revealed that many biological functions in low-risk patients primarily correlated with immune-related biological processes and inflammatory reactions, including cytokine–cytokine (Fig. 9C). The immunological and inflammatory features of the ICDRS were clearly proven, and the potential mechanism of the ICDRS for evaluating the prognosis of patients with LUAD was strongly validated using these results.

### Evaluation of TME and immune characterization of ICDRS

**Fig. 10:**
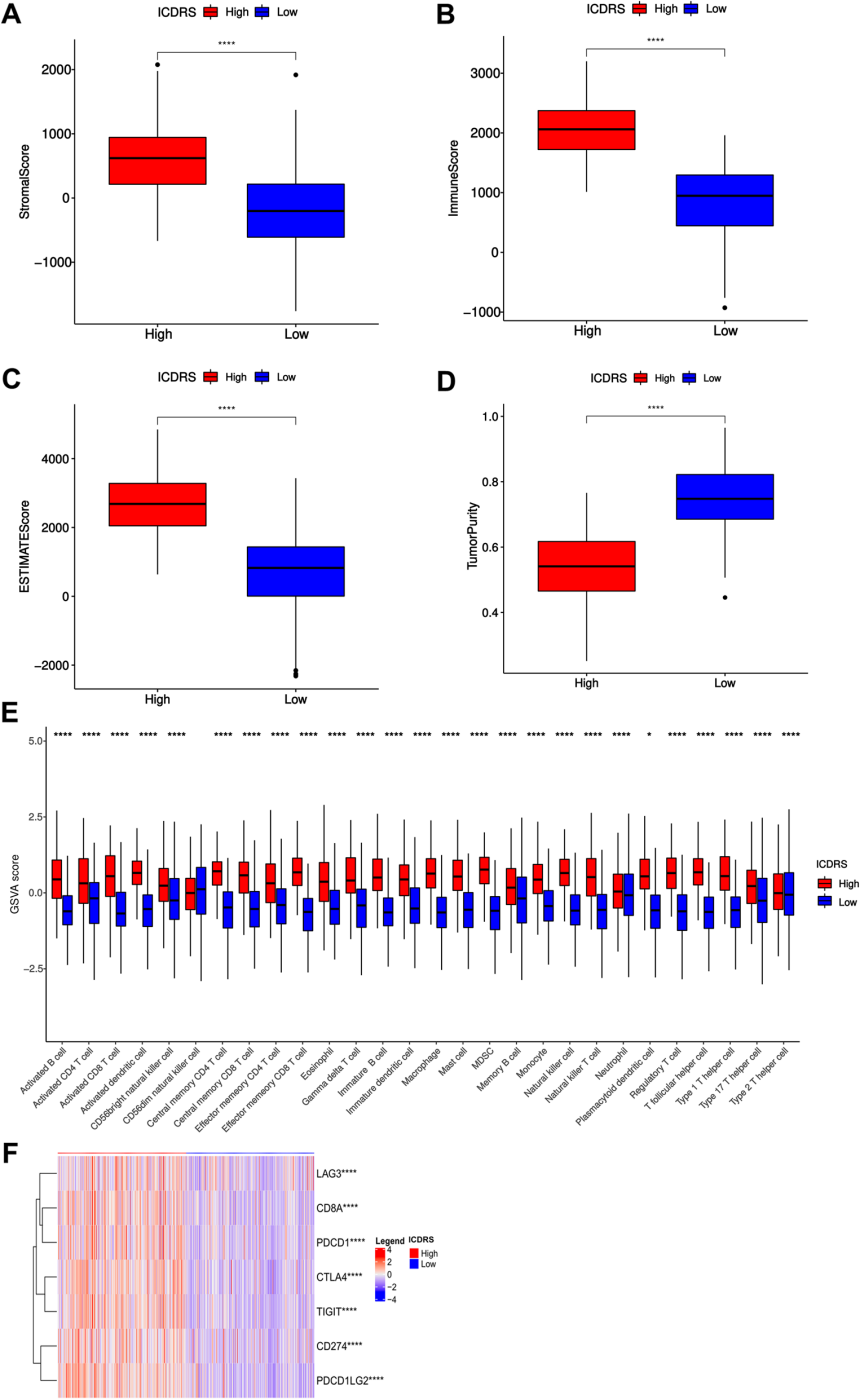
Immunogenic features of different ICDRS groups. **A**-**D** Stromal score(A), Immune score (**B**), ESTIMATE score (**C**) and Tumor purity (**D**) in high and low ICDRS groups. The line in the box represents the median value, and the black dots represented outliers; (**E**) Comparisons of the abundances of 28 immune cells in two ICDRS groups by GSVA; (**F**) The heatmap representing the differential distribution of immune checkpoints in high- and low-ICDRS groups. (*P < 0.05, **P < 0.01, ***P < 0.001, ****P < 0.0001)

Due to the close correlation between ICD gene clusters and immune-related biological pathways, further investigation was performed to analyze the relation between the tumor-infiltrating immune cells and ICDRS. Firstly, we quantified the overall infiltrating immune cells based on TCGA cohort based through ESTIMATE algorithm. As shown, the high ICDRS group represented high Stromal Score, Immune Score and ESTIMATE Score but low tumor purity, suggesting a considerably increasing immune cell infiltration in high ICDRS group (Fig. 10A-D).

A clustering analysis was also performed on the specific difference in 28 types of tumor-infiltrating immune cells between the two groups. The column diagram remarkably revealed a higher infiltrating proportion of antitumor immune cells including activated CD8 T cell and CD4 T cell (*P* < 0.05) in high ICDRS group (Fig. 10E), suggesting a more positive immune response in high ICDRS group. CIBERSORT and TIMER algorithms for the evaluation of the infiltration sufficiency of immune cells also demonstrated the similar results (Fig. S3A, B). Taken together, these results proved that the ICDRS had a tight correlation with tumor immune microenvironment.

To determine the predictive value of the ICDRS in the response of immune checkpoint inhibitor treatment in LUAD, the expression of immune checkpoint was compared in two groups. A significantly high level of them was observed in the high ICDRS group including LAG3, CD274 (PD-L1), CTLA4, PDCD1, TIGIT, PDCD1LG2, demonstrating that the high ICDRS group might benefit from immunotherapy (*P* < 0.05) (Fig. 10F). Spearman correlation analysis indicated that the ICDRS was positively associated with the CD8A and PD-L1 (*p* < 0.001; Fig. 10E, F), suggesting that patients in high ICDRS group might exert a better response to ICI administration. Our findings revealed substantial variations in intrinsic tumor immunogenicity and anticipated immunotherapy response between the low- and high-ICDRS groups.

### Drug susceptibility and mutation analysis

**Fig. 11:**
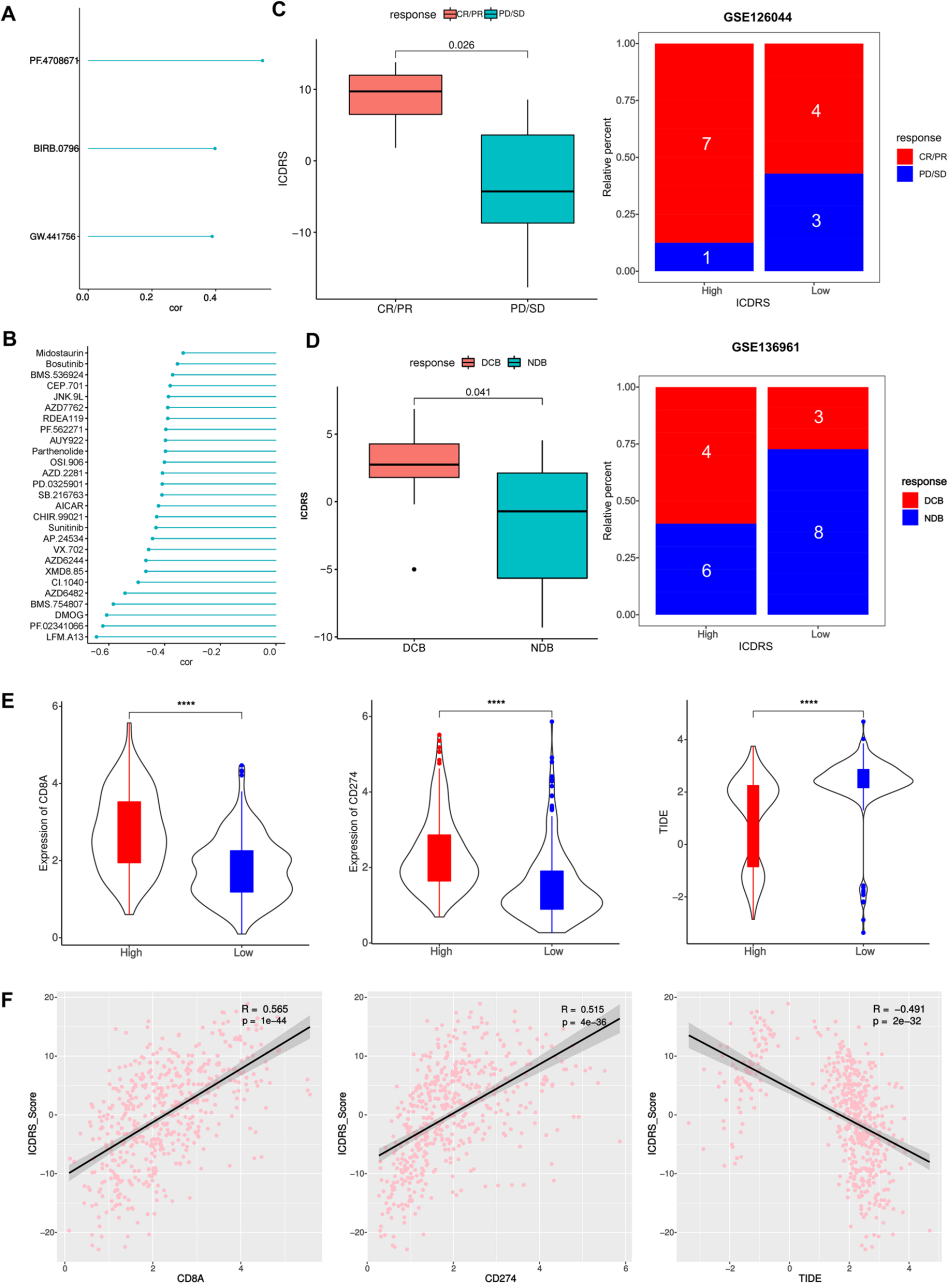
The estimation of two ICDRS groups in immunotherapy and chemotherapy response. **A**, **B** Association between the ICDRS and drug sensitivity, including chemotherapeutics and small molecular drugs, including three drugs with higher IC50 in high-ICDRS group (**A**) and the top 30 of drugs with lower IC50 in high ICDRS (**B**); (**C**) Boxplot and Bar graph illustrated the treatment response [complete response (CR)/partial response (PR) and stable disease (SD)/progressive disease (PD)] to immunotherapy in high and low ICDRS groups in GSE126044 cohorts; (**D**) Boxplot and Bar graph illustrated the treatment response [durable clinical benefit (DCB) and non-durable benefit (NDB)] to immunotherapy in high and low ICDRS groups in GSE136961 cohort; (**E**) Expression differences in CD8A and PD-1, and TIDE score between two ICDRS groups; (**F**) Correlation between the ICDRS and the corresponding immune checkpoints. The Spearman correlation coefficients (R) and corresponding *P* values are shown. (*P < 0.05, **P < 0.01, ***P < 0.001, ****P < 0.0001)

To further estimate the value of ICDRS to predict effects of drugs commonly applied in clinical, the sensitivity of targeted inhibitors was computed between the groups using “pRRophetic” package. Results showed significant differences in the estimated IC50 value of 138 kinds of drug molecules between the low- and high-score groups. It was of 27 out of top significant 30 drugs that patients with higher ICDRS had significantly lower IC50 value compared with those with lower ICDRS, including those of LFM-A13 (BTK inhibitor), PF-02341066 (Crizotinib) (c-Met inhibitor), DMOG (Dimethyloxallyl Glycine) (HIF-PH inhibitor), AZD6482 (PI3Kβ inhibitor), CI-1040 (PD 184352) (MEK Inhibitor), XMD8-85 (ERK inhibitor) and so on, indicating that patients with high ICDRS could be more sensitive to these targeted drugs, which explained the better prognosis of patients in the high ICDRS group (Fig. 11B). The sensitivity of low ICDRS group to the remaining three drugs was higher than that of high ICDRS group (Fig. 11A). In view of these data, the high-ICDRS group might be more sensitive to common chemotherapeutic agents and molecular-targeted drugs. These results suggested that the ICDRS can, to a certain extent, predict drug sensitivity in patients with LUAD. The specific IC50 of every drug was shown in Table S7.

TIDE score, the more accurate predictor for immune checkpoint blockade (ICB) therapies, was introduced into our analysis [28]. Interestingly, patients with LUAD from the high-ICDRS group had a lower TIDE score compared with low-ICDRS patients (Fig. 11E). A greater TIDE score suggests a higher probability of tumor immune escape and lower likelihood of benefitting from anti-PD-1/CTLA4 therapy, illustrating that high-ICDRS patients are candidates for ICB therapy [28]. Currently, the most vital bottlenecks retaining clinical application of immunotherapy is the lack of effectively predictive biomarkers. Because of the marked correlation between ICD subtypes and immune microenvironment as the above studies were shown, we further investigated the predictive ability of ICDRS for immunotherapeutic responses. As shown, the value of ICDRS had a positive correlation with the PD-1 inhibitor therapy responses in the GSE126044 and GSE136961 cohorts. In GSE126044 cohort, the high ICDRS patients possess a lower proportion of SD (stable disease)/PD (progressive disease), and a high proportion of complete response (CR)/partial response (PR) patients in high ICDRS group (87.5%) was significantly higher than that in low ICDRS group (57.1%) (Fig. 11C). In GSE136961 cohort, ICDRS in durable clinical benefit (DCB) group was significantly higher than that in non-durable benefit (NDB) group (*p* < 0.001) (Fig. 11D), and the proportion of DCB was higher in the high ICDRS group (78.6%) than in the low ICDRS group (61.5%; Fig. 11D).

Accumulative evidence shows that patients with a high CD8A or CD274 may benefit from immunotherapy due to their higher numbers of neoantigens. Our analysis of the mutation data from the TCGA LUAD cohort showed a higher CD8A or CD274 in the high score group than that in the low score group (Fig. 11E), suggesting the potential benefits from immunotherapy in the high score group. Spearman correlation analysis demonstrated that the ICDRS was positively associated with the CD8A or CD274 but negatively associated with the TIDE (Fig. 11F). These results suggested that the ICDRS was able to identify high score patients who may benefit from ICB.

### LPS as a known stimulator for TLR4 suppressed tumor growth in vivo.

**Fig. 12:**
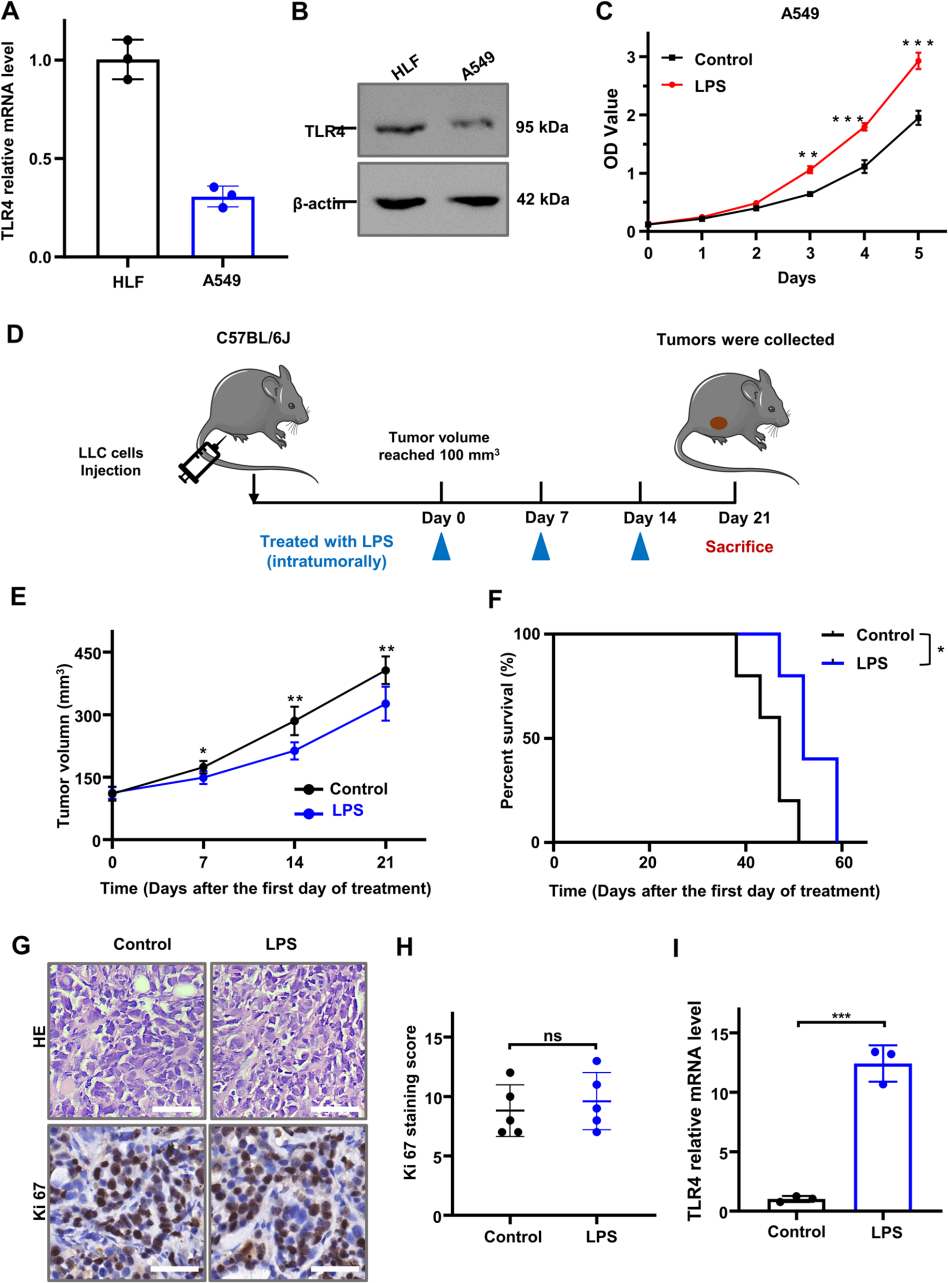
The expression level of TLR4 in vivo and Intratumorally injected with LPS inhibited tumor growth in mouse xenograft model. **A**, **B** The mRNA and protein expressional level of TLR4 in HLF and A549 cells were shown. **C** A549 cells were treated with DMSO (Cnt) or LPS (10 μg/mL) for 5 days and subjected to CCK8 assay. All data represent mean ± SD from three independent experiments; *P* values were calculated in comparison with cells treated with DMSO (Control) unless indicated. **D** The schedule of LPS treated mice with LLC xenograft tumors; (**E**–**H**) Effect of LPS on tumor volume, survival, HE and Ki67 staining of tumor tissues (Scale bar = 50 µm); (**I**) RT-PCR of RNA extracted from tumor tissues treated with or without LPS was carried out to detect the TLR4 mRNA level. GAPDH was used as the loading control. (*P < 0.05, **P < 0.01, ***P < 0.001)

In the previous study, 5 of the 34 ICD-related genes were considerably linked to the prognosis of LUAD patients, including ENTPD1, NLRP3, TLR4, P2RX7 and IL10, which was shown as Fig. 2. Actually, we have investigated published articles on them, but disappointingly, except for TLR4, other four genes are either tumor promoters rather than tumor suppressors both in vivo and in vitro contradicting our present analysis results, or exert different functions in different tumor types [32–34]. Therefore, it is indicated that single gene evaluation on tumor occurrence has limitations and incompleteness, necessary to biological validation. Because of no clear conclusion confirming the specific role in lung adenocarcinoma, TLR4 attracts our attention, which enables to stimulate NLRP3 inflammasomes via enhancing the activation of nuclear factor-κB (NF-κB), releasing more pro-inflammatory cytokines like IL-1β and IL-18 [35, 36]. Furthermore, TLR4 activation has been identified to trigger caspase-1/GSDMD dependent proptosis in tubular cells [37]. And new evidence suggests that TLR4 can be expressed not only on immune cells such as macrophages and dendritic cells, but also on cancer cells. Activating TLR4 on the surface of tumor cells can promote the proliferation and survival of cancer cells, while activating it on immune cells in the tumor microenvironment plays an opposite role. Therefore, in order to verify the function of TLR4, we first chose to compare the TLR4 expression in human lung adenocarcinoma A549 cells and normal human lung fibroblasts HLF cells under in vitro. It was found that the mRNA and protein level of TLR4 in A549 cells was significantly lower than that in HLF normal lung fibrosis cells, which to some extent mean that TLR4 could inhibit tumors (Fig. 12A and B).

Next, LPS, a known TLR4 receptor agonist, was added to treat A549 cells in vitro. Surprisingly, it was found that A549 cells grew faster after LPS treatment, as shown in the Fig. 12C, contrary to our analysis. Regarding different functions of TLR4 receptors on the surface of immune or cancer cells, to further examine the effect of TLR4 activation in vivo, we selected LPS, a known stimulator for TLR4, to treat mice inoculated with LLC cells (Fig. 12D). As shown in Fig. 12E and F, there was a significant decrease in the growth rate of the flank tumors in mice treated with LPS, especially seven, fourteen and twenty-one days later since the first treatment. Treated with LPS led to some elongation of the overall survival with a statistical significance (Log-rank test; *P* = 0.011) (Fig. 12F). In addition, HE and IHC analysis of xenograft tumors demonstrated that LPS treatment made no significant difference in Ki67 staining (a marker of cell proliferation), which might be the reason of that LPS might play two opposite roles when expressed on cancer cells versus immune cells, eventually representing relative moderate effects in tumor suppression (Fig. 12G and H). The mRNA level of TLR4 in tumor tissue treated with LPS was significantly improved compared with that treated with saline, which could be used as a positive indicate of LPS efficiency used for treatment (Fig. 12I). Taken together, the activation of TLR4 could suppress lung adenocarcinoma in vivo to some extent, identical to our previous analysis.

## Discussion

The prognostic evaluation of LUAD has always been a hot topic for concerned scholars. Novel and effective strategies to ameliorate the outcome of lung cancers including immune checkpoint inhibitors, are extensively applied in routine clinical preformation [38]. However, how to specify patients with potentially favorable responses to it remains a challenge.

The emission and interactions with innate immune receptors of active cytokines after inducing ICD exert the crucial function in the initiation of anticancer immunity [39]. It is becoming increasingly clear that ICDRGs have potential ability to predict the response to ICIs treatments in LUAD [40–42]. Therefore, it is deserved to identify ICD-related subtypes or signatures to investigate the promising role of a combined prognosis and immune status classifier for LUAD. Studies have proved the ICD-based gene signature capable of independently predicting prognostic of HNSCC patients [43]. Similarly, three molecular subtypes were identified by the expression of ICD-associated damage-associated molecular pattern (DAMP) in triple-negative breast cancer (TNBC), suggesting that higher gene expression of ICD-associated DAMPs may decide tumor immunogenicity, thereby associating with the good prognosis of TNBC patients [44], inspiring potential benefits from ICD for patients bearing tumor under the interventions of immunotherapy. However, there is still vacancy in ICD-related signature suitable for LUAD patients.

In the current study, the expression of 34 ICDRGs in LUAD tumor tissues and their associations with OS were systematically investigated. Subsequently, two ICD subgroups by consensus clustering were identified based on 34 ICDRGs. After analyzing some related characteristics, we identified two ICD-related gene clusters based on DEGs between the two ICD-related subtypes and showed similar results with the present clustering, demonstrating stability and validity. Subsequently, a robust and effective prognostic scoring system was constructed and validated to quantify the ICD pattern of each LUAD patient and demonstrate its predictive ability, defined as ICDRS, which stratified the LUAD patients into high- and low-ICDRS groups to perform personalized prognostic analysis and proper therapy of LUAD. Moreover, the hybrid nomogram incorporating clinicopathological characteristics and the novel ICD-related prognostic signature was stable and accurate, thus may be applied in clinical management of LUAD patients. Taken together, ICDRS is negatively associated with tumor progression, potentially serving as a biomarker for prognosis classifications of patients with LUAD.

We discovered that the specific features of the TME were noticeably different between the two ICD subtypes or disparate ICDRS groups. Effector T cells, memory T cells and T cell differentiation were previously shown to exert crucial effects on immune-originated defense of LUAD, especially gamma delta T cells, which effectively recognize and attack LUAD cells, ultimately restrain tumor progression via multiple mechanisms [45, 46]. In line with previous study, ICD-high subtype and high ICDRS, both possessed higher infiltration of activated memory CD4 + , CD8 + T cells and gamma delta T cells, which might result from better clinical outcomes. There are several latest researches suggesting B cells-originated involvement in the immune response and that B cell enrichment was considered as the most convincing prognostic indicator and positively associated with the response to PD-1 blockade in soft-tissue sarcomas [47–51]. In our study, we observed the sufficiency of activated B cell, memory B cell and naive B cells in ICD-high subtype and high ICDRS group, which assuredly had better overall survival, in line with the findings of previous studies [50].

Previous studies indicated that patients with higher sensitivity to immunotherapy were at a low risk, identical with what our investigation showed [6]. Through GSEA, our data presented that a high ICDRS was significantly related to immune-activation signature. Subsequent immune analyses also revealed that patients with a higher ICDRS were in concomitant of a relatively high immune status and a higher immune score. Further immune analyses indicated that patients with a better prognosis were in a relatively high immune status (CD8A^high^, PD-1^high^) and possessed a higher immune score, ESTIMATE score and ICDRS. Actually, patients with high ICDRS in two independent cohorts from GEO possessed stronger sensitivity to anti-PD-1 immunotherapy. ICDRS could predicted the immunotherapy outcome of LUAD patients. Our results may meet the need of precision immunotherapy designing for LUAD. Besides, TIDE analysis showed that the TIDE score in high ICDRS group was lower than that in low ICDRS group in TCGA datasets. Moreover, a wide range of chemotherapeutic agents were reported to show striking differences in sensitivity between high and low ICDRS groups. Briefly, high ICDRS group were more sensitive to these targeted drugs. In conclusion, these results indicated that patients in high-ICDRS group might be more strongly responsive to immunotherapies and targeted therapies, and ICDRS could be served as a promising biomarker for predicting the therapeutic efficiency in LUAD, which further represented our score system's stability and independence and underlined its application for therapeutic prediction of patients with LUAD.

Recently, a wide range of studies have reported that gene mutations may be responsible for the response of immune therapy, particularly TMB, capable of predicting response to ICI treatment [52, 53]. However, it is still uncertain whether TMB could serve as a biomarker across all tumors because some studies have also concluded opposite results [54, 55]. According to McGranahan et al., NSCLC patients with high TMB still possessed poor response to immunotherapy, suggesting the limitation of TMB-guided immunotherapy [56]. In our results, the level of ICDRS was negatively linked to the expression of TMB, which also failed to prove the efficacy of TMB-guided immunotherapy, remaining to be further explored.

TLR4 is the best characterized member of transmembrane proteins that play a key role in innate immune and inflammatory responses, which recognizes pathogen-associated molecular patterns and produces various pro-inflammatory cytokines to clear invading pathogens, attracted our attention [57]. LPS is a kind of TLR4 stimulator, which improve the expression and activation of TLR4 and initiates a cascade of downstream events. However, many researches have strongly verified that LPS significantly increased tumor invasion in vitro, which seems opposite to our analyzed resulted. Therefore, in the present study, a murine lung tumor model was performed to evaluate the effects of TLR4 in vivo. Our results have clearly suggested that the activation of TLR4 noticeably retained mice from aggressive tumor progression and improved the survival time of the tumor-bearing mice. Actually, despite the specific function of TLR4 in different cancers investigated by many studies, there are contradictions existing among those researches [58]. Researches were shown that direct injection of LPS into glioblastoma and colorectal cancer led to tumor regression and knocking down TLR4 increased tumor malignancy in a lung metastatic model deprived from breast cancer [58–60]. Opposite results were obtained in these studies that intraperitoneal injection of LPS enhanced the proliferation of cancer cells and retarded apoptosis in metastatic colonic cells, and LPS-mediated chronic inflammation promoted tobacco carcinogen-induced lung cancer [61, 62]. We also conducted in vitro experiments to verify the effect of LPS as a TLR4 agonist. We found that adding a certain dose of LPS promoted cancer cells growth in vitro, as shown in Fig. 12C. These controversies may result, at least partially, from the pattern or dosage of LPS administration, but more importantly from deficiency in discrimination of TLR4 expressing on the surface of immune cells or cancer cells. There existed an assumption that TLR4 may play two entirely antithetical roles when solely expressed on cancer cells or immune cells. Given our results, it was revealed that even in vivo model, the activation of TLR4 played a key role in preventing lung cancer progression, in consistent with our bioinformation analysis deprived from TCGA and GEO profiles.

This study had several limitations, which are as follows: Firstly, despite additional confirmation of animal experiments, all analyses were conducted solely on data from public databases, indicating that potential selection bias may inescapably influence the results. Extensive range of prospective and comprehensive experimental studies and clinical trials are needed to further identify our results. Furthermore, before applying to the immunotherapies, some important clinical therapies for patients such as surgery, neoadjuvant chemotherapy and chemoradiotherapy, may make differences in the prognosis of the immune response. However, information about these treatment histories is usually unknown in most datasets. Therefore, more independent and integrated immunotherapy cohorts are required to validate the accuracy and rationality of the ICDRS.

Taken together, this study provided more evidence that ICD exerts noticeable effects in regulating immune infiltration status in the tumor microenvironment, clinicopathological features, and prognosis in LUAD patients. We also established an innovative and clinically applicative ICDRS signature as an independent prognostic factor of LUAD, which could serve as a promising signature for further mechanism research about ICD and a potential approach for filtering sensitive responders of targeted therapy and immunotherapy in the future. These findings emphasize the crucial roles of ICD and bring new inspirations of tactical and individualized immunotherapy for patients with LUAD.

## Nomenclature

LUAD: Lung adenocarcinoma; ICD: Immunogenic Cell Death; ICDRS: ICD-related Score; AUC: area under the ROC curve; CDF: cumulative distribute ion function; CM: consensus matrix; CMap: Connectivity Map; CNV: copy number variation; DEG: differentially expressed gene; ESTIMATE: Estimation of STromal and Immune cells in MAlignant Tumor tissues using Expression data; FC: fold change; GO: Gene Ontology; GSVA: Gene set variation analysis; HCC: hepatocellular carcinoma; ICB: immune-checkpoint blockade; ICGC: International Cancer Genome Consortium; ICI: immune checkpoint inhibitor; IC50: half-maximal inhibitory concentration; KEGG: Kyoto encyclopedia of genes and genomes; K-M: Kaplan-Meier; MDSC: myeloid-derived suppressor cell; TMB: tumor mutation burden; OS: overall survival; ROC: receiver operating characteristic; ssGSEA: single sample Gene Set Enrichment Analysis; TAM: tumor-associated macrophages; TCGA: The Cancer Genome Atlas; TME: tumor microenvironment; ICI: immune checkpoint inhibitor; GEO: Gene-Expression Omnibus; TCGA: The Cancer Genome Atlas; OS: overall survival; GO: Geno Ontology; KEGG: Kyoto Encyclopedia of Genes and Genomes; GSVA: unsupervised gene set variation analysis; GSEA: gene set enrichment analysis; TIDE: tumor immune dysfunction and exclusion; MAF: mutation annotation format.

### Supplementary Information

**Additional file 1: Figure S1.** Identification of ICDRS prognostic value in lung adenocarcinoma (LUAD) in GEO cohorts. **Figure S2.** The stratification analysis of ICDRS and independent prognosis analysis of ICDRS and clinicopathological variables in LUAD. **Figure S3.** The immune cells infiltrations in two high- and low-ICDRS groups and the correlation between TMB and ICDRS. **Table S1.** Clinical information of 572 lung cancer patients in TCGA-LUAD. **Table S2.** Clinical information of 398 lung cancer patients in GEO profiles. **Table S3.** 879 DEGs identified by secondary clustering. **Table S4.** 113 DEGs related to survival time in TCGA profiles. **Table S5.** 326 genes with differential mutation frequency between high- and low-score groups. **Table S6.** 18430 genes with differential CNAs between high- and low-score groups. **Table S7.** Differential sensitivity of 138 drugs in different score groups.

## Data Availability

Publicly available datasets were analyzed in the study. The RNA-seq data of TCGA_LUAD and GEO were separately from https://portal.gdc.cancer.gov/ and https://www.ncbi.nlm.nih.gov/gds.
