# Supplementary material for: Comprehensive analysis of the immunogenic cell death-related signature for predicting prognosis and immunotherapy efficiency in patients with lung adenocarcinoma
Source: BMC Med Genomics. 2023 Aug 8;16:184. doi: 10.1186/s12920-023-01604-w (PMC10410984; doi:10.1186/s12920-023-01604-w)
Supplement: Supplementary file 1 — Additional file 1: Figure S1. Identification of ICDRS prognostic value in lung adenocarcinoma (LUAD) in GEO cohorts. Figure S2. The stratification analysis of ICDRS and independent prognosis analysis of ICDRS and clinicopathological variables in LUAD. Figure S3. The immune cells infiltrations in two high- and low-ICDRS groups and the correlation between TMB and ICDRS. Table S1. Clinical information of 572 lung cancer patients in TCGA-LUAD. Table S2. Clinical information of 398 lung cancer patients in GEO profiles. Table S3. 879 DEGs identified by secondary clustering. Table S4. 113 DEGs related to survival time in TCGA profiles. Table S5. 326 genes with differential mutation frequency between high- and low-score groups. Table S6. 18430 genes with differential CNAs between high- and low-score groups. Table S7. Differential sensitivity of 138 drugs in different score groups. [file 12920_2023_1604_MOESM1_ESM.pdf]

Supplementary Material

Article Title

Yingshu Cui[1,2†], Yi Li[2†], Shan Long[3†], Yuanyuan Xu[4], Xinxin Liu[5], Zhijia Sun[6], Yuanyuan Sun[1], Jia Hu[1*], Xiaosong Li[1*]

[1]Department of Oncology, the Fifth Medical Center, Chinese PLA General Hospital, Beijing, China.
[2]Medical School of Chinese PLA, Beijing, China.
[3]School of Medicine, Nankai University, Tianjin, China.
[4]Department of Oncology, the Affiliated Hospital of Southwest Medical University, Sichuan, China
[5]Department of General Surgery, Peking University First Hospital, Beijing, China.
[6]Department of Radiation Oncology, Air Force General Hospital, Beijing, China.
† These authors contributed equally to this work and share first authorship

***Correspondence:**
Xiaosong Li
lixiaosong301@163.com

Jia Hu
hujiain301@163.com

Figure S1

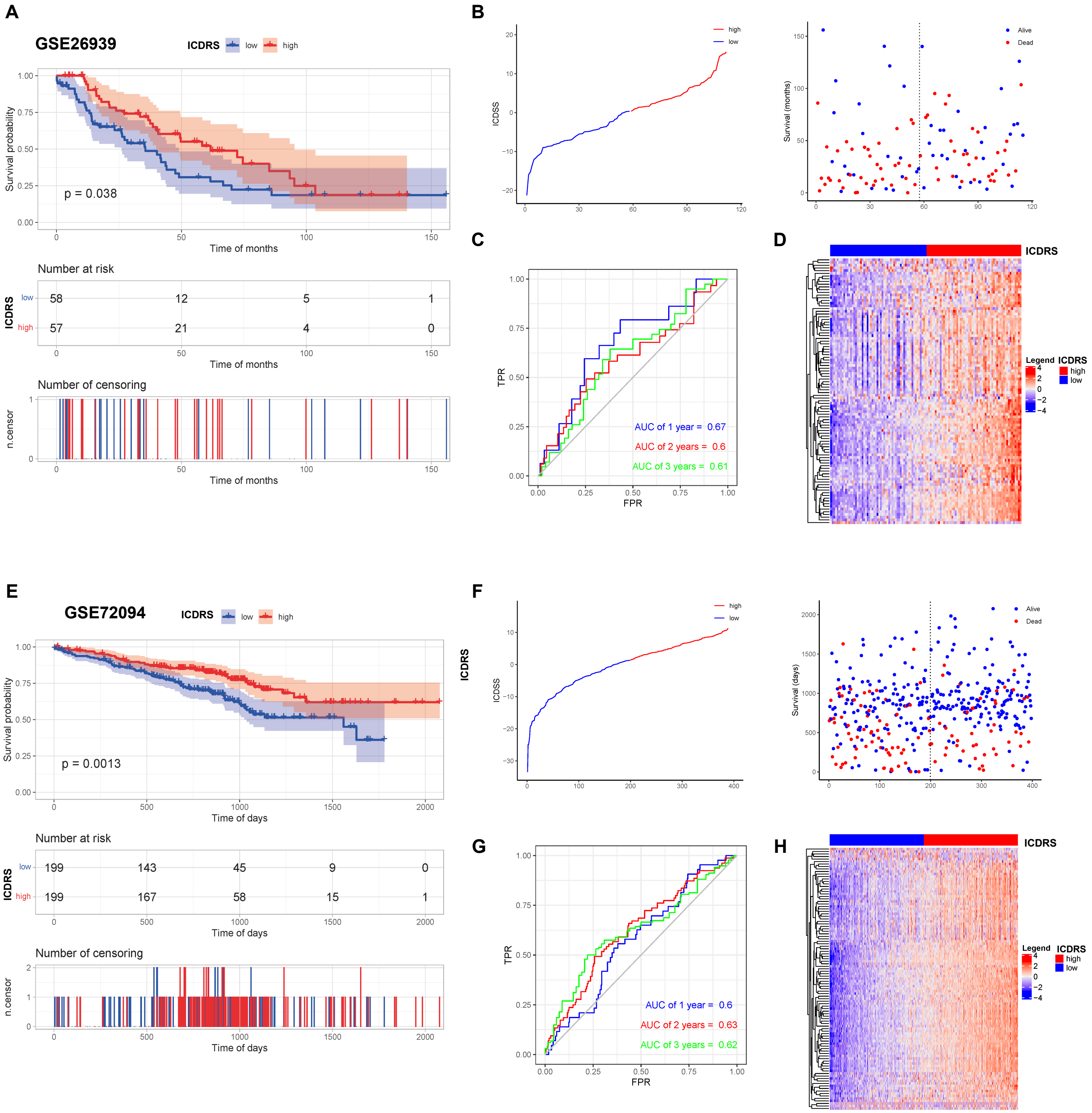

Figure S1. Identification of ICDRS prognostic value in lung adenocarcinoma (LUAD) in GEO cohorts.
(A, E) Kaplan–Meier curves of the prognostic ICDRS model in GSE26939 (A) and GSE72094 (E). The association between the ICDRS and the survival of patients was investigated using Cox regression and log-rank methods;
(B, F) ICDRS scores distribution and survival status of LUAD patients in high-and low- ICDRS groups.
(C, G) Time-dependent receiver operating characteristic (ROC) curves of the prognostic ICDRS model in GSE26939 (C) and GSE72094 (G). The association between the ICDRS and the survival of patients was investigated using Cox regression and log-rank methods;
(D, H) The heat map depicted the expression of DEGs in high and low ICDRS groups. Heat map colors indicate relative DEGs expression levels.

Figure S2

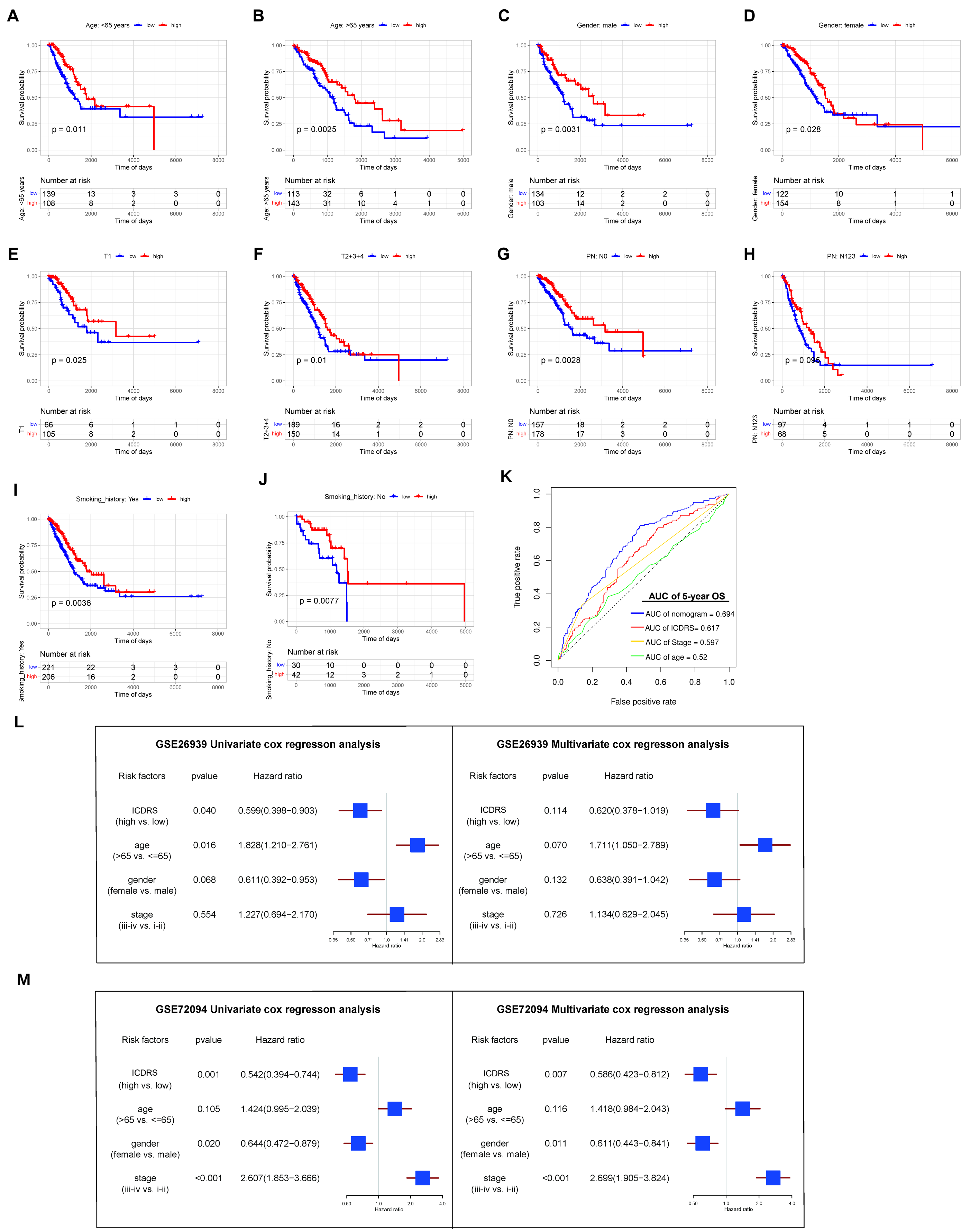

Figure S2. The stratification analysis of ICDRS and independent prognosis analysis of ICDRS and clinicopathological variables in LUAD. (A-J) The stratification analysis of ICDRS was shown based on some clinical characteristics, including age (age ≤ 65 and age > 65 years old) (A, B), Gender (male and female) (C-D), T stage (1 or 2-4) (E-F), N stage (0 or 1-3) (G-H), smoking history (yes and no) (I-J) .
(K) ROC curves of the nomograms compared with those of other clinical variables with regard to five-year survival in TCGA.
(L, M) Univariate and multivariate analyses showed the prognostic value of the ICDRS in the corresponding GEO cohorts.

Figure S3

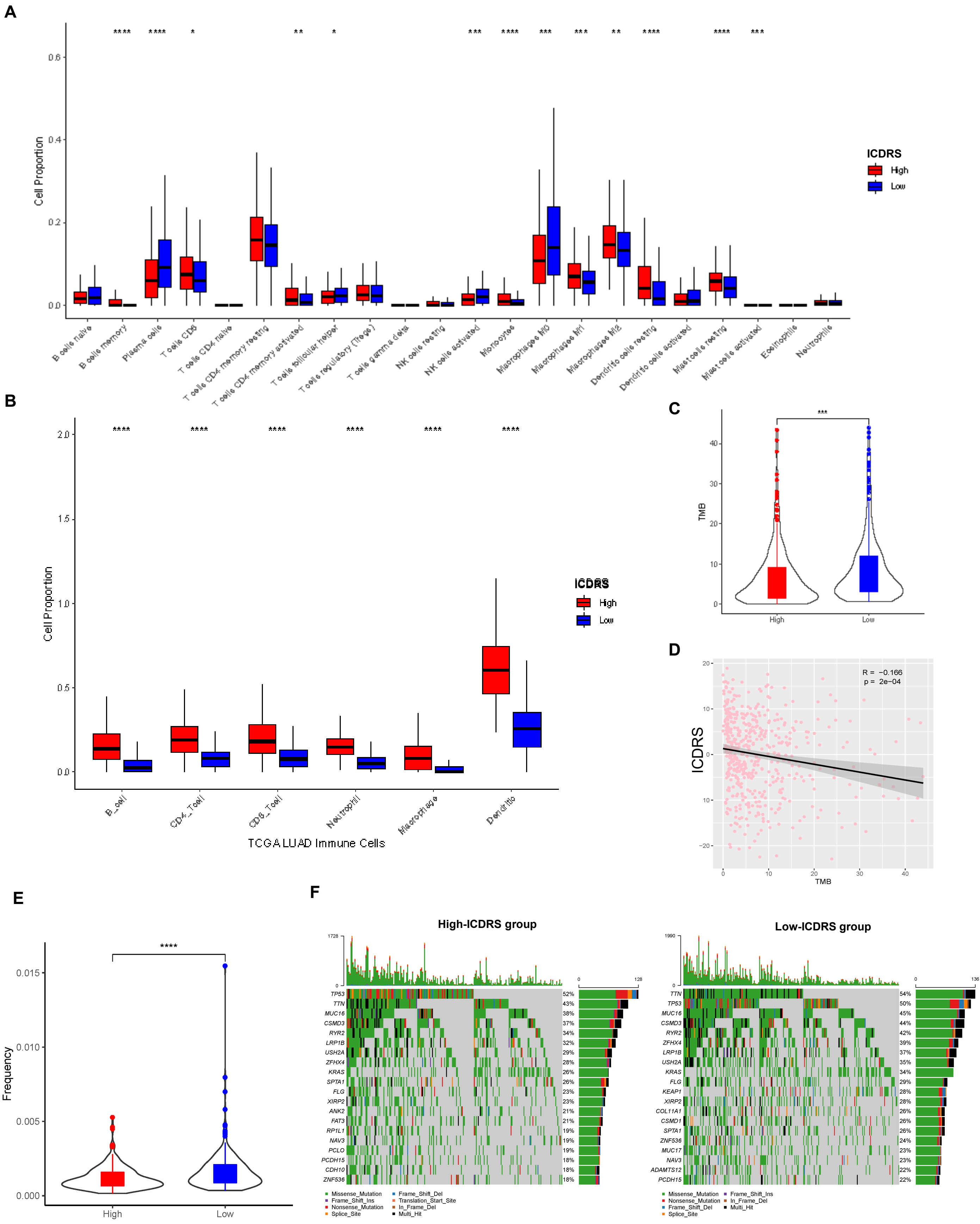

Figure S3. The immune cells infiltrations in two high- and low-ICDRS groups and the correlation between TMB and ICDRS.
(A, B) Comparisons of the abundances of 22 immune cells in two groups by CIBERSORT and TIMER.
(C) Expression differences in TMB between two ICDRS groups.
(D) Correlation between the ICDRS and TMB. The Spearman correlation coefficients (R) and corresponding P values are shown.
(E) Violin plots showing the frequency of CNV in high- and low-ICDRS groups.
(F) Oncoprint visualization of the top 20 most frequently mutated genes in high- (left part) and low- (right part) ICDRS groups.
(*P < 0.05, **P < 0.01, ***P < 0.001, ****P < 0.0001).

Weston Blot

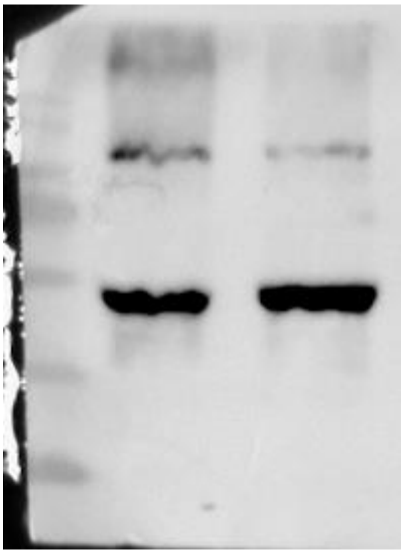

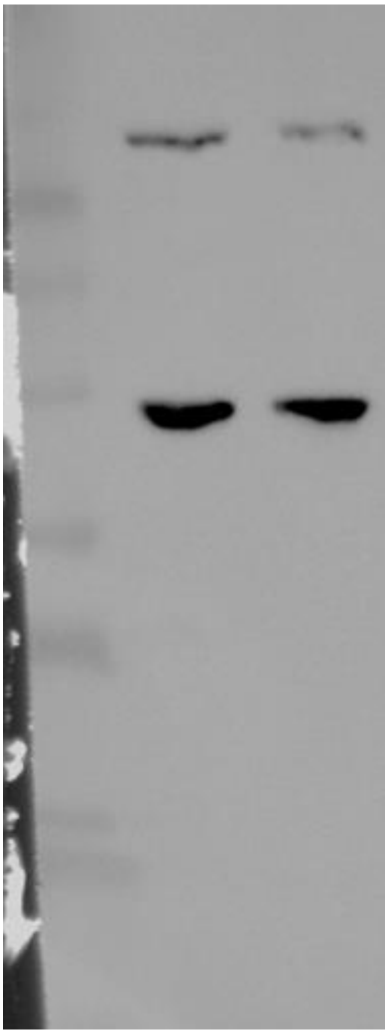

Supplementary tables

Table S1: Clinical information of 572 lung cancer patients in TCGA-LUAD

Table S2: Clinical information of 398 lung cancer patients in GEO profiles

Table S3: 879 DEGs identified by secondary clustering

Table S4: 113 DEGs related to survival time in TCGA profiles

Table S5: 326 genes with differential mutation frequency between high- and low-score groups

Table S6: 18430 genes with differential CNAs between high- and low-score groups

Table S7: Differential sensitivity of 138 drugs in different score groups

Table
Table 1:

Table S1: Clinical information of 572 lung cancer patients in TCGA-LUAD

Table S1		
	TCGA-Tumor (513)	TCGA-Normal (59)
Age (years)		
>65	256	30
<=65	247	29
Unknown	10	
Gender		
male	237	25
female	276	34
Pathologic_M		
M0	342	40
M1	24	2
MX	142	16
Unknown	5	1
pathologic_N		
N0	335	30
N1	94	12
N2	69	13
N3	2	0
NX	12	4
Unknown	1	0
pathologic_T		
T1	171	19
T2	275	37
T3	46	2
T4	18	1
TX	3	0
stage		
stage i	280	30
stage ii	120	13
stage iii	80	13
stage iv	25	2
unknown	8	1
smoking_history		
Yes	427	46
No	76	7
Unknown	10	6
cluster		
cluster1	255	\
cluster2	258	\
DEG.cluster		
cluster1	303	\
cluster2	210	\
OS		
Alive	328	\
Dead	185	\
DFI		
Alive	214	\
Dead	95	\
Unknown	204	\
PFI		
Alive	297	\
Dead	216	\

Table S2: Clinical information of 398 lung cancer patients in GEO profiles

Table S2		
	GSE72094 (398)	GSE26939 (115)
age		
>65	280	56
<=65	118	59
gender		
M	176	49
F	222	50
Unknown	0	16
stage		
1	254	55
2	67	16
3	57	14
4	15	2
Unknown	5	28
OS		
Alive	285	49
Dead	113	66

Table S3: 879 DEGs identified by secondary clustering

Table S3						
	logFC	AveExpr	t	P.Value	adj.P.Val	B
CXCL9	2.080682	4.268292	15.59332	3.87E-45	2.93E-43	91.80236
CXCL10	1.912477	4.244148	16.27444	2.66E-48	2.32E-46	99.04039
CYBB	1.750394	4.340621	22.21136	4.79E-77	4.44E-74	164.8509
CCL19	1.733064	4.123674	12.97041	1.82E-33	7.98E-32	65.10777
HLA-DQA	1.717355	4.886626	18.69776	7.50E-60	1.46E-57	125.4791
CXCL13	1.686071	3.735493	12.26856	1.63E-30	6.22E-29	58.36481
LYZ	1.669293	7.357212	13.08406	5.96E-34	2.66E-32	66.21776
S100P	-1.6372	5.296826	-6.63065	8.50E-11	9.93E-10	13.54082
PTPRC	1.598053	3.200748	24.76909	1.27E-89	8.26E-86	193.6414
HLA-DRA	1.53529	9.804319	16.73205	1.89E-50	1.89E-48	103.9582
CCL5	1.528332	4.652152	16.03687	3.41E-47	2.88E-45	96.50409
UBD	1.524253	2.969474	13.17724	2.37E-34	1.06E-32	67.13133
C1QB	1.524125	6.759967	15.7488	7.40E-46	5.72E-44	93.44528
PLEK	1.523962	3.449924	24.5873	9.91E-89	4.82E-85	191.6011
HLA-DPA1	1.511359	6.145966	16.37978	8.54E-49	7.67E-47	100.1688
GZMA	1.503842	3.292631	16.94477	1.87E-51	1.99E-49	106.2578
HLA-DOA	1.501765	3.778316	17.40965	1.16E-53	1.43E-51	111.3109
FCGR3A	1.488677	4.838201	18.10074	5.69E-57	9.32E-55	118.885
HLA-DRB5	1.488106	7.26412	11.69416	3.65E-28	1.26E-26	52.99923
CD53	1.480902	4.868492	24.12902	1.76E-86	4.90E-83	186.4517
C1QC	1.474067	6.802222	15.82191	3.40E-46	2.66E-44	94.21984
CD2	1.472524	3.460015	21.18083	5.62E-72	3.42E-69	153.2436
JCHAIN	1.462368	7.609254	9.934228	2.20E-21	5.56E-20	37.54382
OR2I1P	1.459619	2.421813	13.31227	6.21E-35	2.85E-33	68.46088
FGL2	1.457257	3.220176	19.26028	1.40E-62	3.45E-60	131.728
MRC1	1.454549	3.613814	13.57167	4.66E-36	2.26E-34	71.03336
CALHM6	1.446487	2.640006	17.85675	8.45E-56	1.27E-53	116.2031
HLA-DQA	1.441119	4.415128	9.513603	7.19E-20	1.69E-18	34.09635
GBP1	1.43059	3.748602	17.54746	2.55E-54	3.31E-52	112.8157
AKR1C2	-1.42647	2.61742	-5.82794	9.94E-09	8.81E-08	8.899137
C1QA	1.419847	6.85902	14.88594	6.63E-42	4.28E-40	84.40289
GBP5	1.414926	2.104426	18.96231	3.92E-61	8.78E-59	128.414
CD163	1.413531	3.73393	15.85074	2.50E-46	1.98E-44	94.52565
HLA-DPB1	1.410431	6.892855	15.5712	4.89E-45	3.68E-43	91.56902
SLAMF7	1.40818	3.05962	17.07934	4.30E-52	4.76E-50	107.7168
CCL18	1.405542	5.427409	10.47505	2.14E-23	5.94E-22	42.12283
ITGB2	1.400235	4.607099	17.17269	1.55E-52	1.77E-50	108.7308
IL7R	1.399922	3.032037	17.97743	2.23E-56	3.47E-54	117.5286
VSIG4	1.399092	4.05243	14.57214	1.73E-40	1.03E-38	81.16324
HLA-DRB1	1.383291	8.907989	13.35293	4.15E-35	1.94E-33	68.86252
LCP1	1.378155	5.145051	19.40751	2.69E-63	6.98E-61	133.3685
NKG7	1.376685	3.240426	14.94816	3.46E-42	2.26E-40	85.04849
FPR3	1.375496	3.49886	20.11223	9.77E-67	3.28E-64	141.2445
F13A1	1.372757	3.159834	13.62782	2.65E-36	1.30E-34	71.59326
HLA-DMB	1.372666	4.156967	18.47533	8.92E-59	1.61E-56	123.0174
GBP4	1.360625	3.13996	18.40805	1.88E-58	3.37E-56	122.2739
SFTPC	1.356297	5.12264	4.423424	1.19E-05	6.22E-05	2.064357
MNDA	1.355515	3.042303	20.06778	1.61E-66	5.32E-64	140.7467
CXCL11	1.353489	1.85451	14.15934	1.21E-38	6.59E-37	76.94539
LAPTM5	1.353208	6.328219	20.62191	3.11E-69	1.38E-66	146.9609
HLA-DQB	1.348194	5.575974	12.03747	1.46E-29	5.40E-28	56.18879
GPX2	-1.34432	3.478127	-5.22393	2.55E-07	1.83E-06	5.753076
C3AR1	1.335275	3.302276	20.54285	7.60E-69	3.22E-66	146.0732
PLA2G2D	1.334018	1.732604	13.82838	3.50E-37	1.76E-35	73.6019
MARCO	1.333524	3.928777	9.228656	7.20E-19	1.58E-17	31.8202
EVI2B	1.331982	3.559719	22.21944	4.37E-77	4.31E-74	164.942

AIM2	1.330725	1.989091	12.92	2.99E-33	1.30E-31	64.61704
SASH3	1.325363	3.285546	23.38786	7.74E-83	1.37E-79	178.1101
CD3D	1.324869	3.245003	17.48725	4.94E-54	6.25E-52	112.1579
SLAMF8	1.319671	3.181744	20.1454	6.72E-67	2.30E-64	141.6161
CD48	1.319474	2.69584	18.77249	3.26E-60	6.62E-58	126.3075
MS4A6A	1.319315	3.141426	20.71513	1.09E-69	5.43E-67	148.0078
CD3E	1.31165	2.988885	18.95773	4.13E-61	9.03E-59	128.3631
IL4I1	1.311024	2.647117	16.03283	3.56E-47	2.99E-45	96.46111
CD52	1.310156	5.037059	14.56392	1.88E-40	1.12E-38	81.07877
ALOX5AP	1.302358	4.245688	15.05145	1.17E-42	7.82E-41	86.12248
AIF1	1.301035	4.411143	19.24484	1.66E-62	4.00E-60	131.5561
CCL13	1.30025	3.324394	10.80691	1.16E-24	3.47E-23	45.01015
CD79A	1.299843	3.75632	10.23657	1.68E-22	4.49E-21	40.08393
FYB1	1.298985	2.54489	20.82887	3.01E-70	1.63E-67	149.2858
IDO1	1.296449	3.084188	10.68323	3.46E-24	1.01E-22	43.92741
FCER1G	1.293629	5.522604	17.71658	3.96E-55	5.47E-53	114.6663
CD74	1.292753	9.842507	13.57333	4.58E-36	2.22E-34	71.04987
SELL	1.291661	2.81001	16.22583	4.48E-48	3.90E-46	98.52044
CSF1R	1.291538	3.834898	18.52785	4.97E-59	9.23E-57	123.5982
GZMK	1.283427	1.860372	17.76818	2.24E-55	3.20E-53	115.2316
CCL4	1.281799	2.470837	19.27534	1.18E-62	2.95E-60	131.8958
CD8A	1.273686	2.275762	17.19799	1.18E-52	1.36E-50	109.0058
IL2RG	1.273196	3.911391	16.92617	2.29E-51	2.39E-49	106.0565
IGSF6	1.267925	2.81883	19.94737	6.25E-66	2.00E-63	139.3988
MPEG1	1.26723	3.317753	19.46802	1.36E-63	3.74E-61	134.0433
ADAMDEC	1.263006	2.035751	12.55783	1.01E-31	4.07E-30	61.12002
GPR183	1.259605	3.424726	16.33939	1.32E-48	1.16E-46	99.73583
CCR5	1.259588	2.073431	25.44947	5.95E-93	5.79E-89	201.2641
TNFSF13B	1.251367	2.691701	21.37115	6.52E-73	4.38E-70	155.3855
TFF1	-1.25043	2.23272	-5.03052	6.78E-07	4.51E-06	4.810865
ITGAL	1.246816	2.617897	20.44253	2.36E-68	9.57E-66	144.9475
IGLL5	1.246654	4.937542	8.453734	2.95E-16	5.54E-15	25.88546
CD4	1.243277	4.445362	19.6078	2.84E-64	8.14E-62	135.6031
IL10RA	1.241099	2.731869	24.25043	4.46E-87	1.74E-83	187.8167
MSMB	-1.24098	3.171485	-4.8936	1.33E-06	8.36E-06	4.163301
HAVCR2	1.239237	2.748198	22.42813	4.10E-78	4.70E-75	167.2943
OLR1	1.239201	3.155889	12.87538	4.63E-33	1.99E-31	64.18344
SLCO2B1	1.23727	3.068407	18.06152	8.78E-57	1.39E-54	118.4533
PIK3AP1	1.235056	2.593669	22.2183	4.43E-77	4.31E-74	164.9292
RGS1	1.233966	4.226818	12.43162	3.42E-31	1.34E-29	59.91367
HCK	1.227705	3.340758	17.04164	6.49E-52	7.11E-50	107.3077
GZMB	1.220661	2.641078	12.43153	3.42E-31	1.34E-29	59.91287
LILRB4	1.218716	2.347967	17.82937	1.14E-55	1.69E-53	115.9027
EVI2A	1.218264	2.745614	19.74547	6.05E-65	1.79E-62	137.141
NCKAP1L	1.216558	2.348398	24.00124	7.47E-86	1.82E-82	185.0146
BCL2A1	1.21641	3.065919	16.91376	2.62E-51	2.73E-49	105.9222
CSF2RB	1.215047	2.445561	22.49417	1.94E-78	2.52E-75	168.0388
GPNMB	1.214833	5.239667	12.75866	1.45E-32	6.04E-31	63.05287
PTGDS	1.213356	4.27094	10.46105	2.42E-23	6.69E-22	42.0023
HLA-DQB	1.210167	4.36178	8.733188	3.52E-17	6.98E-16	27.98146
IL2RB	1.209868	2.447282	19.24919	1.58E-62	3.86E-60	131.6045
TNC	1.207939	4.313612	8.576199	1.17E-16	2.25E-15	26.79772
FGA	-1.2075	2.785224	-4.72543	2.97E-06	1.74E-05	3.39025
IRF8	1.205622	2.439073	20.99002	4.86E-71	2.79E-68	151.0974
SAMSN1	1.203888	2.430624	21.35021	8.26E-73	5.36E-70	155.1498
FOLR2	1.197381	3.546525	12.84706	6.10E-33	2.61E-31	63.90861
MSR1	1.197378	2.803767	15.10788	6.50E-43	4.40E-41	86.71047
CD14	1.197338	5.417627	14.72403	3.58E-41	2.21E-39	82.72786

SPI1	1.195769	4.09787	16.03729	3.39E-47	2.87E-45	96.50862
AOAH	1.195494	2.001196	22.67292	2.56E-79	3.56E-76	170.0537
CHIT1	1.193696	2.439153	8.505972	1.99E-16	3.77E-15	26.27338
FGL1	-1.19048	1.840332	-6.27568	7.42E-10	7.61E-09	11.42493
TYROBP	1.190424	6.062933	13.45121	1.56E-35	7.37E-34	69.83585
GIMAP4	1.182543	3.729515	21.93718	1.07E-75	8.34E-73	161.7609
LTB	1.18238	3.2966	12.58792	7.58E-32	3.08E-30	61.40861
CLEC10A	1.180382	1.902919	18.18512	2.23E-57	3.85E-55	119.8143
CD69	1.174644	2.071921	17.033	7.13E-52	7.72E-50	107.214
LCP2	1.173731	2.609126	25.47617	4.40E-93	5.79E-89	201.5628
PRF1	1.173157	2.561256	15.45594	1.66E-44	1.21E-42	90.35534
GZMH	1.17076	2.181042	14.21401	6.91E-39	3.84E-37	77.50095
MS4A4A	1.16836	3.071689	16.57799	1.00E-49	9.44E-48	102.298
CYTIP	1.16714	2.702305	19.08184	1.03E-61	2.42E-59	129.7424
PTAFR	1.165042	2.874761	17.08176	4.19E-52	4.66E-50	107.7431
FDCSP	1.164456	2.131272	8.501734	2.06E-16	3.89E-15	26.24184
CD86	1.164105	2.589335	23.02487	4.74E-81	7.69E-78	174.0204
SRGN	1.163634	6.616098	14.10416	2.12E-38	1.15E-36	76.38546
FERMT3	1.16159	3.577144	18.39831	2.10E-58	3.72E-56	122.1664
MMP9	1.161551	4.486639	9.097672	2.04E-18	4.37E-17	30.79046
CD37	1.156377	3.039674	17.67516	6.25E-55	8.40E-53	114.2126
CD27	1.15413	2.682521	13.90892	1.55E-37	7.90E-36	74.41234
CD84	1.149204	1.874764	20.85391	2.26E-70	1.26E-67	149.5672
MMP12	1.143735	3.219452	7.266756	1.38E-12	1.93E-11	17.57473
DOK2	1.14102	2.829968	17.21343	9.94E-53	1.16E-50	109.1737
MZB1	1.14071	3.677988	8.831341	1.65E-17	3.32E-16	28.72962
SELPLG	1.138018	3.806727	17.59783	1.46E-54	1.93E-52	113.3664
PLAAT4	1.137748	5.558517	10.21891	1.96E-22	5.20E-21	39.93416
ITGAM	1.135985	2.511304	16.44153	4.38E-49	4.01E-47	100.8313
LCK	1.134312	2.293085	18.68166	8.97E-60	1.73E-57	125.3007
MS4A1	1.133241	1.479787	11.72558	2.73E-28	9.44E-27	53.28888
CST7	1.128068	3.314185	13.54128	6.31E-36	3.02E-34	70.73075
SAA1	1.121353	3.065889	6.5881	1.11E-10	1.27E-09	13.28201
PSMB9	1.121223	4.173915	13.34282	4.59E-35	2.12E-33	68.76262
TAGAP	1.120565	2.017263	23.81043	6.47E-85	1.40E-81	182.8678
PDCD1LG2	1.119241	1.646589	21.52848	1.10E-73	7.64E-71	157.1569
RNASE6	1.118348	3.853544	17.76928	2.22E-55	3.20E-53	115.2437
LAIR1	1.118328	2.393616	20.63471	2.69E-69	1.28E-66	147.1046
CLEC4A	1.117564	2.248002	19.41984	2.34E-63	6.25E-61	133.506
HLA-B	1.116844	9.291114	14.28539	3.32E-39	1.87E-37	78.22783
CORO1A	1.116471	3.879562	16.70261	2.60E-50	2.56E-48	103.6406
CCR1	1.115013	2.846274	17.44479	7.87E-54	9.77E-52	111.6944
SLAMF6	1.112688	1.923643	18.6124	1.94E-59	3.71E-57	124.5337
BIRC3	1.111945	3.420557	11.80325	1.32E-28	4.66E-27	54.00707
SAMD9L	1.109206	2.708837	15.21652	2.08E-43	1.43E-41	87.84482
RAC2	1.109155	4.65351	16.87697	3.91E-51	3.98E-49	105.524
LST1	1.107363	2.834536	15.58811	4.09E-45	3.09E-43	91.74735
TREM2	1.107209	4.085652	11.4482	3.54E-27	1.17E-25	50.7468
CD274	1.106026	1.934564	14.10133	2.19E-38	1.18E-36	76.35678
SIT1	1.105616	1.840407	18.16599	2.76E-57	4.68E-55	119.6035
MMP7	1.104427	4.276826	6.564408	1.28E-10	1.46E-09	13.1385
MAFB	1.104283	3.664387	17.79466	1.68E-55	2.45E-53	115.522
RARRES1	1.097861	3.439493	10.47697	2.11E-23	5.86E-22	42.13939
ARHGAP3	1.095297	2.997385	20.32478	8.90E-68	3.47E-65	143.6266
P2RY13	1.094856	1.660693	20.74425	7.82E-70	4.01E-67	148.3349
CPS1	-1.09446	1.553277	-5.16201	3.50E-07	2.44E-06	5.447956
SIGLEC10	1.094353	1.927936	17.92642	3.91E-56	5.91E-54	116.9682
ALOX5	1.092834	3.589328	14.32841	2.14E-39	1.21E-37	78.66666

APOL3	1.091504	2.651553	19.10926	7.58E-62	1.80E-59	130.0473
CCL3	1.090919	2.40452	14.53725	2.48E-40	1.46E-38	80.80483
VCAM1	1.090212	2.657998	14.19617	8.29E-39	4.58E-37	77.31958
NCF2	1.090129	3.7267	15.46093	1.57E-44	1.15E-42	90.40781
MS4A7	1.083711	2.888885	15.17281	3.29E-43	2.25E-41	87.38812
IL2RA	1.082521	1.853052	17.05301	5.73E-52	6.31E-50	107.4312
LY86	1.076543	2.866533	14.94507	3.58E-42	2.33E-40	85.01635
CD40	1.075109	3.662702	14.71725	3.84E-41	2.37E-39	82.65783
AKR1C1	-1.07031	2.574929	-5.07353	5.47E-07	3.68E-06	5.017606
CLEC7A	1.063832	2.586509	15.04981	1.19E-42	7.92E-41	86.10539
GIMAP7	1.06104	3.296514	16.90951	2.74E-51	2.84E-49	105.8761
CPVL	1.059719	3.264474	12.67136	3.38E-32	1.39E-30	62.21068
GPR34	1.059365	2.185413	15.05452	1.14E-42	7.62E-41	86.15446
SAMHD1	1.059307	4.574968	18.77938	3.02E-60	6.26E-58	126.3838
WAS	1.058453	2.869679	19.03433	1.75E-61	4.02E-59	129.2143
PLAU	1.05721	5.206675	8.08777	4.43E-15	7.70E-14	23.21889
HLA-F	1.056288	4.496575	13.0374	9.43E-34	4.17E-32	65.76138
HK3	1.056161	2.206772	13.90639	1.59E-37	8.08E-36	74.38683
CRLF1	-1.05526	3.306117	-4.95582	9.81E-07	6.32E-06	4.455541
CYTH4	1.05347	2.170492	24.16495	1.17E-86	3.80E-83	186.8557
DOCK2	1.052356	1.813741	22.17203	7.48E-77	6.33E-74	164.4077
FPR1	1.050424	2.369444	14.72616	3.50E-41	2.17E-39	82.74982
LSP1	1.050054	3.708193	14.40405	9.81E-40	5.62E-38	79.43957
APOC1	1.048452	5.812261	9.343065	2.87E-19	6.53E-18	32.72822
CMKLR1	1.047617	2.059809	18.51782	5.56E-59	1.01E-56	123.4872
HCLS1	1.046666	3.669274	16.33423	1.40E-48	1.22E-46	99.68061
CASP1	1.046183	2.838865	18.9597	4.04E-61	8.93E-59	128.385
SH2D1A	1.044955	1.34094	20.63145	2.80E-69	1.30E-66	147.068
CXCR3	1.043698	1.939047	15.50763	9.59E-45	7.11E-43	90.8993
SPN	1.043378	1.91703	18.7671	3.46E-60	6.95E-58	126.2476
TLR4	1.043359	2.060056	18.17226	2.58E-57	4.40E-55	119.6726
TNFAIP8L2	1.042696	2.548299	18.08941	6.45E-57	1.05E-54	118.7603
CHI3L1	1.041878	4.711992	7.279122	1.27E-12	1.78E-11	17.65615
SLA	1.040753	2.288812	19.45809	1.52E-63	4.12E-61	133.9325
LPXN	1.039348	3.332696	22.46225	2.79E-78	3.39E-75	167.679
DPT	1.038983	3.333208	9.737215	1.14E-20	2.76E-19	35.91634
CPA3	1.038892	3.253898	8.587852	1.07E-16	2.06E-15	26.88503
CIITA	1.038889	2.124086	15.99108	5.57E-47	4.64E-45	96.01665
WIPF1	1.032452	3.066804	21.25999	2.29E-72	1.44E-69	154.1343
HSD11B1	1.032174	2.217747	15.46749	1.47E-44	1.08E-42	90.47683
CCL8	1.030082	1.767552	11.48482	2.53E-27	8.39E-26	51.08034
BTK	1.029098	1.896982	22.09582	1.77E-76	1.44E-73	163.5487
PTPN7	1.026677	1.846341	22.23708	3.58E-77	3.87E-74	165.1409
CCR2	1.026569	1.502823	21.01829	3.53E-71	2.08E-68	151.4153
HCST	1.02598	3.143907	14.75474	2.60E-41	1.63E-39	83.04498
HLA-DOB	1.025661	2.257729	14.60295	1.26E-40	7.54E-39	81.48011
IKZF1	1.023632	1.718218	20.5881	4.56E-69	1.97E-66	146.5812
GMFG	1.021718	3.790002	16.93134	2.16E-51	2.28E-49	106.1124
CD5	1.020972	1.974489	16.66171	4.05E-50	3.86E-48	103.1997
FCGR2A	1.020371	3.559933	15.3824	3.61E-44	2.61E-42	89.58268
SIRPA	1.014403	3.457193	16.40235	6.69E-49	6.04E-47	100.4108
CXCR4	1.013088	5.131431	14.00695	5.72E-38	2.96E-36	75.4015
GNLY	1.011893	2.055832	10.15691	3.33E-22	8.74E-21	39.40974
NCF4	1.011387	3.33706	15.35539	4.80E-44	3.41E-42	89.29922
ARHGAP9	1.011003	1.943908	20.67286	1.75E-69	8.53E-67	147.533
CXCR6	1.010364	1.59763	19.38332	3.53E-63	8.92E-61	133.0989
TGFBI	1.009959	4.759469	9.577809	4.25E-20	1.01E-18	34.61592
KLRB1	1.00855	1.99341	16.50135	2.30E-49	2.14E-47	101.4738

APBB1IP	1.007911	2.42516	18.76127	3.70E-60	7.35E-58	126.183
ITGAX	1.00754	2.708284	15.33615	5.88E-44	4.15E-42	89.0975
S100B	1.007153	2.401613	9.256186	5.78E-19	1.28E-17	32.03797
TFF3	-1.00562	4.401732	-4.3664	1.53E-05	7.83E-05	1.823109
GIMAP6	1.005268	2.594902	19.00704	2.38E-61	5.39E-59	128.911
BIN2	1.004031	2.100861	20.5089	1.12E-68	4.62E-66	145.6922
TNFAIP3	1.003935	3.516265	14.78551	1.89E-41	1.20E-39	83.36304
FGR	0.999509	2.650266	15.89943	1.48E-46	1.19E-44	95.04246
CTSS	0.999268	5.834019	15.37845	3.76E-44	2.72E-42	89.54123
CCL2	0.998767	4.211952	11.22702	2.67E-26	8.56E-25	48.74558
IRF4	0.998089	1.542309	14.03404	4.34E-38	2.28E-36	75.67539
HLA-DMA	0.997391	5.781987	11.46113	3.15E-27	1.04E-25	50.86455
SLC7A7	0.996798	2.842985	17.77215	2.15E-55	3.12E-53	115.2752
CCR7	0.995318	1.862533	14.19963	8.00E-39	4.44E-37	77.35472
PTPN22	0.995033	1.717156	17.36651	1.86E-53	2.25E-51	110.8406
TLR8	0.992812	1.290537	19.97538	4.56E-66	1.48E-63	139.7123
DCN	0.990733	4.645267	10.8628	7.06E-25	2.14E-23	45.50207
BPIFA1	-0.99036	4.057553	-3.18806	0.00152	0.00487	-2.50016
SIGLEC1	0.990349	2.100902	13.94237	1.10E-37	5.67E-36	74.74948
P2RY10	0.986312	1.375727	18.77805	3.06E-60	6.28E-58	126.3691
GIMAP2	0.984355	2.61148	17.47236	5.82E-54	7.31E-52	111.9953
TRIM22	0.982448	3.689812	14.06842	3.06E-38	1.63E-36	76.02336
PTGER4	0.98187	2.052745	17.56666	2.06E-54	2.70E-52	113.0255
FCMR	0.981055	2.425005	14.04153	4.02E-38	2.12E-36	75.75118
CTSK	0.980991	4.971453	10.49097	1.87E-23	5.20E-22	42.26002
PIM2	0.979666	4.524796	12.26603	1.67E-30	6.35E-29	58.34087
DOCK8	0.978704	2.088041	18.91573	6.59E-61	1.41E-58	127.8967
PLXNC1	0.977396	2.171888	19.57791	3.97E-64	1.12E-61	135.2694
FGG	-0.97687	3.967962	-3.652	0.000287	0.001107	-0.95055
AKR1C3	-0.9768	3.633276	-5.37265	1.18E-07	8.93E-07	6.49931
C1orf162	0.975221	2.874496	16.37311	9.18E-49	8.20E-47	100.0972
CD79B	0.97412	1.959787	12.03488	1.50E-29	5.52E-28	56.16459
BHLHE41	0.97217	2.472437	13.47653	1.21E-35	5.75E-34	70.08711
MILR1	0.968061	2.168811	15.91472	1.26E-46	1.02E-44	95.20489
FAS	0.96798	2.802951	14.39807	1.04E-39	5.94E-38	79.37835
SCIMP	0.967618	1.449734	22.19943	5.48E-77	4.85E-74	164.7165
PILRA	0.966984	2.530782	16.12369	1.34E-47	1.16E-45	97.42961
RCSD1	0.965596	2.03469	20.43169	2.67E-68	1.06E-65	144.8257
C7	0.965486	3.368293	6.845254	2.19E-11	2.71E-10	14.86752
C1S	0.964581	5.615658	12.47148	2.33E-31	9.24E-30	60.29397
B2M	0.96333	9.517319	16.60116	7.80E-50	7.38E-48	102.5474
ANKRD22	0.962923	3.126816	9.124317	1.65E-18	3.55E-17	30.99907
GBP2	0.961462	3.90284	14.62566	9.94E-41	5.99E-39	81.71382
PLA2G7	0.96024	2.920089	12.69348	2.73E-32	1.13E-30	62.42376
CD300LF	0.960159	2.13405	14.2953	3.00E-39	1.69E-37	78.32891
JAML	0.958558	1.801625	17.35538	2.10E-53	2.52E-51	110.7192
SNX10	0.958305	3.123981	14.02504	4.76E-38	2.49E-36	75.58435
CCL4L2	0.957998	1.959795	13.28255	8.35E-35	3.80E-33	68.16766
TAP1	0.957802	5.061859	12.20587	2.96E-30	1.12E-28	57.77222
APOE	0.95724	6.659357	7.871399	2.11E-14	3.47E-13	21.68523
ARHGEF6	0.955725	2.2076	19.8309	2.32E-65	7.05E-63	138.096
HAPLN3	0.9557	2.489522	15.08454	8.30E-43	5.60E-41	86.46723
SPP1	0.954748	6.911877	5.471354	7.00E-08	5.48E-07	7.004891
APOBEC3C	0.952784	2.047779	17.10044	3.42E-52	3.87E-50	107.9458
LILRB2	0.952676	1.793413	18.31582	5.24E-58	9.21E-56	121.2556
RASAL3	0.950713	1.983507	17.09393	3.67E-52	4.13E-50	107.8752
MYO1F	0.948	2.236502	19.90025	1.06E-65	3.28E-63	138.8717
TESC	-0.94704	3.994578	-4.99081	8.25E-07	5.41E-06	4.621381

CD247	0.945918	1.521457	20.62368	3.05E-69	1.38E-66	146.9807
RUNX3	0.945114	2.306292	16.0379	3.37E-47	2.87E-45	96.51516
TNFAIP2	0.944958	4.466236	9.748865	1.03E-20	2.52E-19	36.01196
CCDC80	0.944195	3.22527	10.5932	7.64E-24	2.18E-22	43.14421
MYO1G	0.941609	2.250789	10.26567	1.31E-22	3.51E-21	40.33107
BARX1	-0.93992	1.580307	-5.42596	8.91E-08	6.87E-07	6.771353
LRRC25	0.937064	2.224457	16.69085	2.95E-50	2.89E-48	103.5138
SNX20	0.935475	1.550408	23.77052	1.02E-84	1.98E-81	182.4186
FGB	-0.93449	2.174997	-3.53233	0.000449	0.001653	-1.36943
CTSW	0.932638	2.575025	10.66146	4.20E-24	1.22E-22	43.73764
NFAM1	0.931753	1.794166	18.06265	8.67E-57	1.39E-54	118.4658
TBC1D10C	0.931278	1.720145	16.72309	2.08E-50	2.07E-48	103.8615
IRF1	0.930402	3.509197	15.55456	5.83E-45	4.35E-43	91.39364
SLC15A3	0.928752	2.942373	14.98231	2.42E-42	1.60E-40	85.40326
CTSE	0.928211	5.037707	4.243141	2.62E-05	0.000127	1.311538
CXCL12	0.927991	2.607042	12.01778	1.76E-29	6.45E-28	56.00449
G0S2	0.926991	4.342593	7.563907	1.83E-13	2.80E-12	19.56208
DPYD	0.92651	3.179137	11.81786	1.15E-28	4.08E-27	54.14243
CD96	0.923155	1.555989	15.22759	1.85E-43	1.28E-41	87.96062
SFRP2	0.923065	5.223938	5.857199	8.43E-09	7.54E-08	9.059231
LAT2	0.922722	2.298401	18.51821	5.54E-59	1.01E-56	123.4915
WARS1	0.922332	5.336486	11.50277	2.15E-27	7.14E-26	51.24414
TNFRSF17	0.922217	1.79861	10.2957	1.01E-22	2.72E-21	40.58664
LUM	0.921481	7.194163	9.683514	1.78E-20	4.26E-19	35.4766
EREG	0.921118	1.33302	6.341933	4.99E-10	5.24E-09	11.81232
CD6	0.919298	1.726967	17.39515	1.36E-53	1.66E-51	111.1528
C16orf54	0.919149	1.722112	15.20548	2.33E-43	1.60E-41	87.72944
TRPV2	0.918559	2.911064	16.7191	2.17E-50	2.15E-48	103.8185
CD300A	0.918166	2.624985	15.75092	7.24E-46	5.62E-44	93.46782
MMP2	0.916224	5.828421	9.474906	9.85E-20	2.29E-18	33.78438
STAT1	0.916067	5.808098	12.86277	5.23E-33	2.25E-31	64.061
MAP4K1	0.915835	1.9992	15.83979	2.81E-46	2.21E-44	94.4095
PDCD1	0.915822	1.362157	15.80865	3.91E-46	3.05E-44	94.07934
CCL21	0.915545	4.978752	7.209708	2.03E-12	2.78E-11	17.20058
FCGR1A	0.915531	1.617673	17.5188	3.49E-54	4.48E-52	112.5024
LAX1	0.914123	1.393348	14.18095	9.69E-39	5.32E-37	77.16491
IL1B	0.913191	1.632422	13.5741	4.54E-36	2.21E-34	71.05755
OSCAR	0.909835	2.446036	12.14131	5.48E-30	2.06E-28	57.16374
TLR2	0.909703	3.345276	10.35949	5.84E-23	1.59E-21	41.13102
ABI3	0.907635	2.700456	18.10167	5.63E-57	9.30E-55	118.8952
STX11	0.906081	2.040876	17.49381	4.60E-54	5.85E-52	112.2295
CTLA4	0.90548	1.464777	16.6692	3.73E-50	3.58E-48	103.2803
PPP1R16B	0.905447	1.725875	15.54043	6.78E-45	5.04E-43	91.24467
MFAP4	0.90485	4.81609	7.375986	6.62E-13	9.51E-12	18.2978
LTF	0.904551	3.462805	5.102686	4.73E-07	3.21E-06	5.158673
FCGR2B	0.90239	1.589267	17.44933	7.49E-54	9.35E-52	111.7439
ETS1	0.902203	3.859779	14.44179	6.65E-40	3.87E-38	79.82586
KCTD12	0.902133	4.182036	11.95199	3.28E-29	1.19E-27	55.38982
TIGIT	0.901931	1.248027	19.78304	3.97E-65	1.19E-62	137.5609
GLIPR2	0.9011	3.425433	13.32084	5.71E-35	2.63E-33	68.54555
SIRPG	0.900694	1.308496	18.07922	7.22E-57	1.16E-54	118.6481
FXYD4	-0.90057	1.017386	-7.43668	4.38E-13	6.42E-12	18.70334
SLC1A3	0.900002	1.630773	15.90398	1.41E-46	1.14E-44	95.09074
ADA2	0.898878	3.87972	11.99903	2.10E-29	7.67E-28	55.8291
ODC1	-0.89857	5.680461	-7.19413	2.25E-12	3.07E-11	17.09881
CD3G	0.898269	1.35558	18.94675	4.66E-61	1.01E-58	128.2412
CSF1	0.897674	3.533094	12.00707	1.95E-29	7.13E-28	55.90424
TGM2	0.897655	5.711646	11.02192	1.70E-25	5.25E-24	46.91102

DAPP1	0.897355	2.230451	14.52427	2.84E-40	1.67E-38	80.67153
ZAP70	0.895431	1.42534	15.30605	8.08E-44	5.64E-42	88.782
CCL17	0.893545	1.935034	7.682948	7.97E-14	1.26E-12	20.37608
CD44	0.890964	4.913702	11.93229	3.94E-29	1.43E-27	55.20609
INPP5D	0.890943	2.368102	15.56604	5.16E-45	3.87E-43	91.51467
FN1	0.889703	7.053414	8.331695	7.36E-16	1.35E-14	24.98624
CRYBG1	0.88897	2.827985	10.69805	3.04E-24	8.91E-23	44.05673
SERPINB9	0.888948	2.875621	14.67875	5.73E-41	3.51E-39	82.26069
GLIPR1	0.888645	2.159208	17.36844	1.82E-53	2.22E-51	110.8616
P2RX7	0.888612	1.417483	20.23102	2.56E-67	9.60E-65	142.5755
PLEKHO2	0.88858	3.699621	19.4693	1.34E-63	3.74E-61	134.0575
ACAP1	0.88825	1.739649	16.95858	1.61E-51	1.72E-49	106.4075
TFEC	0.885893	1.113297	20.20566	3.41E-67	1.23E-64	142.2913
CLEC2B	0.885413	2.57545	12.82833	7.33E-33	3.12E-31	63.72706
RASSF2	0.885385	2.720877	14.66641	6.51E-41	3.98E-39	82.13354
EPSTI1	0.883084	2.585646	13.22619	1.46E-34	6.60E-33	67.61256
LILRB1	0.882461	1.542048	20.2231	2.80E-67	1.03E-64	142.4867
AREG	0.880159	3.889742	5.232942	2.44E-07	1.75E-06	5.797777
CCR4	0.878391	1.25679	15.37112	4.07E-44	2.92E-42	89.46433
LIPA	0.876039	4.825506	13.20178	1.86E-34	8.37E-33	67.37243
PLXDC2	0.875406	2.820226	12.84358	6.31E-33	2.70E-31	63.8749
C5AR1	0.868517	3.056478	12.30095	1.20E-30	4.59E-29	58.67156
IL21R	0.868322	1.191294	21.56695	7.10E-74	5.12E-71	157.5902
IFI16	0.868115	4.36358	10.55981	1.02E-23	2.89E-22	42.85485
CSF2RA	0.867987	2.316486	12.96191	1.98E-33	8.65E-32	65.02498
CX3CL1	0.867979	3.525566	7.072307	5.03E-12	6.65E-11	16.30931
COTL1	0.867804	4.438254	12.79841	9.81E-33	4.15E-31	63.43724
ZNF683	0.86735	1.140738	12.81746	8.15E-33	3.47E-31	63.62171
COL6A3	0.86718	4.934043	9.069888	2.55E-18	5.40E-17	30.5734
ITM2A	0.867153	3.06692	11.98804	2.33E-29	8.50E-28	55.72635
CYP1B1	0.86609	3.431046	9.258568	5.67E-19	1.26E-17	32.05683
CILP	0.865465	1.663388	8.165879	2.50E-15	4.44E-14	23.78044
GPR84	0.863944	1.208737	17.08392	4.09E-52	4.58E-50	107.7666
LAG3	0.86323	1.684114	11.69095	3.76E-28	1.29E-26	52.96961
PGC	-0.86222	4.499759	-2.75726	0.006037	0.016329	-3.75775
SERPING1	0.861929	6.220506	11.71608	2.98E-28	1.03E-26	53.20124
ITGA4	0.861467	1.684645	15.65107	2.09E-45	1.61E-43	92.41193
TNFRSF1B	0.861041	4.051431	14.77518	2.10E-41	1.32E-39	83.25624
GPR174	0.860412	0.870796	17.6282	1.05E-54	1.40E-52	113.6987
INHA	-0.85998	1.154165	-6.07073	2.48E-09	2.39E-08	10.24869
SCGB1A1	0.858656	4.584629	2.87797	0.00417	0.011789	-3.42315
CD83	0.858257	3.38759	12.73291	1.86E-32	7.75E-31	62.80412
TMEM59L	-0.85745	1.919684	-5.37499	1.17E-07	8.85E-07	6.511223
PREX1	0.855501	2.87505	15.60735	3.33E-45	2.55E-43	91.95036
C15orf48	0.854652	4.597268	5.838107	9.38E-09	8.34E-08	8.954686
GPR65	0.854014	1.232144	23.00607	5.86E-81	8.78E-78	173.8085
PIK3CG	0.852642	1.234079	18.05233	9.73E-57	1.53E-54	118.3522
DOCK11	0.851433	2.139757	14.75288	2.65E-41	1.66E-39	83.02579
LGALS2	0.850379	1.988743	10.60044	7.17E-24	2.05E-22	43.20702
CCND2	0.849711	2.600253	12.13206	5.98E-30	2.24E-28	57.07677
IL16	0.849577	1.470412	20.15101	6.31E-67	2.20E-64	141.679
PIGR	0.849545	5.847955	3.620559	0.000323	0.001228	-1.06188
VAV1	0.849413	2.621018	14.63073	9.43E-41	5.70E-39	81.76602
ARHGAP2	0.847667	2.132599	20.15389	6.11E-67	2.16E-64	141.7112
PDPN	0.847482	2.855208	9.51766	6.95E-20	1.63E-18	34.1291
EMP3	0.845963	4.383877	11.71182	3.10E-28	1.07E-26	53.16198
KLHL6	0.845449	1.372624	16.67135	3.65E-50	3.52E-48	103.3036
CH25H	0.843836	2.483967	7.653816	9.78E-14	1.53E-12	20.17594

CST6	0.84292	3.233134	5.636861	2.86E-08	2.37E-07	7.870974
EGR2	0.840468	2.096769	12.91356	3.18E-33	1.38E-31	64.55433
AQP9	0.83862	1.994889	10.22137	1.92E-22	5.10E-21	39.95502
NLRC5	0.837667	2.313625	14.1652	1.14E-38	6.23E-37	77.00487
AC005747	0.837197	2.021024	14.84373	1.03E-41	6.58E-40	83.96552
UBE2L6	0.837053	5.398335	12.48756	2.00E-31	7.93E-30	60.44758
SPOCK2	0.836138	3.271379	9.545845	5.52E-20	1.30E-18	34.35695
TNFSF10	0.834684	5.000925	9.008787	4.12E-18	8.58E-17	30.09773
ABI3BP	0.834575	1.62669	11.91206	4.77E-29	1.72E-27	55.01761
POU2AF1	0.834345	1.856582	9.478516	9.57E-20	2.23E-18	33.81345
COL3A1	0.833901	7.776641	6.766805	3.61E-11	4.37E-10	14.37845
CARD16	0.833649	2.341688	15.33993	5.65E-44	4.00E-42	89.13713
IL32	0.833648	4.46872	9.354398	2.62E-19	5.98E-18	32.8186
TMEM273	0.833556	1.801987	17.94275	3.27E-56	5.01E-54	117.1475
PCSK2	-0.83308	0.800605	-5.56799	4.17E-08	3.37E-07	7.50778
IKZF3	0.832331	1.914374	13.68469	1.49E-36	7.43E-35	72.16144
SUSD3	0.831628	2.238639	14.53252	2.61E-40	1.53E-38	80.75624
DOCK10	0.830913	1.683717	15.49471	1.10E-44	8.12E-43	90.76321
PSTPIP1	0.82989	1.460011	19.03668	1.71E-61	3.96E-59	129.2403
C1R	0.82983	5.520378	12.04199	1.40E-29	5.18E-28	56.23117
RASD1	-0.8289	4.730895	-5.14655	3.79E-07	2.61E-06	5.372255
CD8B	0.828223	1.400296	12.80536	9.17E-33	3.88E-31	63.50451
DAB2	0.8263	3.179462	13.59862	3.55E-36	1.73E-34	71.30196
CALCA	-0.82567	1.313985	-3.41394	0.000691	0.002424	-1.77079
ST8SIA4	0.825459	2.369532	11.75739	2.03E-28	7.05E-27	53.58267
FMO2	0.825051	2.304216	9.691737	1.66E-20	3.99E-19	35.54382
ERAP2	0.824664	2.641363	7.41085	5.22E-13	7.57E-12	18.53042
NPL	0.824314	2.577738	13.67385	1.67E-36	8.26E-35	72.05302
SULF1	0.824248	3.803744	7.142459	3.17E-12	4.26E-11	16.7626
AXL	0.823965	3.415639	11.83206	1.01E-28	3.58E-27	54.2741
S100A8	0.822377	3.848586	5.454189	7.67E-08	5.96E-07	6.916379
IL12RB1	0.82207	1.254176	21.70768	1.44E-74	1.08E-71	159.1752
BTN3A3	0.821355	2.813317	16.67395	3.54E-50	3.45E-48	103.3315
TRAF3IP3	0.821034	1.164894	19.6975	1.04E-64	3.02E-62	136.605
TRAF1	0.821007	2.12124	16.52277	1.82E-49	1.71E-47	101.704
CD180	0.820216	1.374637	18.71664	6.08E-60	1.20E-57	125.6883
MXRA5	0.819401	3.449967	7.84555	2.53E-14	4.14E-13	21.50419
HLA-E	0.818951	8.084293	14.23068	5.82E-39	3.25E-37	77.67058
STAB1	0.818685	2.80377	12.45614	2.70E-31	1.07E-29	60.14753
CD300C	0.818423	1.547789	15.59696	3.72E-45	2.83E-43	91.84072
THEMIS2	0.816708	3.379997	13.94124	1.12E-37	5.72E-36	74.73812
PTGS1	0.81575	2.126968	14.08578	2.56E-38	1.37E-36	76.19916
CXorf21	0.815484	1.58128	17.73056	3.40E-55	4.73E-53	114.8194
ICOS	0.814535	1.005953	20.79072	4.63E-70	2.44E-67	148.857
TLR7	0.814244	1.134501	19.41055	2.60E-63	6.84E-61	133.4024
ADAM19	0.814079	2.184695	12.67532	3.25E-32	1.34E-30	62.24885
AC136428	0.813383	1.296046	8.774399	2.56E-17	5.11E-16	28.29484
FCN1	0.812933	1.563189	11.55252	1.36E-27	4.53E-26	51.69866
PLEKHO1	0.812142	2.994325	15.05138	1.18E-42	7.82E-41	86.12175
APOL6	0.811004	3.498063	14.50106	3.61E-40	2.10E-38	80.43333
RASSF4	0.810786	2.844294	14.01122	5.48E-38	2.85E-36	75.44464
IFI44	0.809151	3.948993	7.768607	4.37E-14	7.03E-13	20.96805
CHRDL1	0.808401	2.086248	7.584401	1.58E-13	2.45E-12	19.70149
ROS1	0.807158	2.848828	6.292339	6.72E-10	6.93E-09	11.52201
HLA-C	0.806323	8.904743	10.55231	1.09E-23	3.07E-22	42.78991
IFIT3	0.805683	4.039232	9.930995	2.26E-21	5.70E-20	37.51693
GBP3	0.8054	3.33043	8.402163	4.35E-16	8.08E-15	25.50426
CLEC5A	0.805363	1.692782	10.81209	1.11E-24	3.32E-23	45.05567

LHFPL2	0.804109	3.041927	13.56341	5.06E-36	2.44E-34	70.95102
SIGLEC9	0.802434	1.49339	17.69282	5.15E-55	7.01E-53	114.406
CD209	0.801748	1.474295	13.81999	3.81E-37	1.91E-35	73.51765
OSM	0.801611	1.674605	10.56313	9.94E-24	2.81E-22	42.88357
IFI44L	0.7978	2.069306	7.920298	1.48E-14	2.48E-13	22.029
TMEM119	0.797671	3.180634	9.853711	4.32E-21	1.08E-19	36.876
RRAD	0.79753	3.342188	6.148386	1.58E-09	1.56E-08	10.69041
PARVG	0.797257	1.670041	16.88217	3.69E-51	3.80E-49	105.5802
VNN2	0.796641	1.510582	11.93983	3.67E-29	1.33E-27	55.27636
GPR68	0.796483	2.265978	10.76351	1.70E-24	5.05E-23	44.62936
MOXD1	0.796478	2.963262	9.757783	9.60E-21	2.35E-19	36.0852
GPR171	0.796046	1.595998	9.477317	9.66E-20	2.25E-18	33.80379
SOD2	0.795884	4.47188	11.91736	4.54E-29	1.64E-27	55.06697
PRKCB	0.794451	1.364252	16.46714	3.32E-49	3.08E-47	101.1063
ITK	0.794232	1.028921	17.6257	1.08E-54	1.43E-52	113.6712
TPSAB1	0.793921	2.939104	7.192868	2.27E-12	3.10E-11	17.09059
CLMP	0.793583	2.024474	9.936143	2.16E-21	5.48E-20	37.55975
SEPTIN1	0.793485	1.923788	14.19802	8.13E-39	4.50E-37	77.33837
CD7	0.792338	2.285159	9.749545	1.03E-20	2.51E-19	36.01754
DSE	0.791455	1.615357	14.97625	2.58E-42	1.70E-40	85.34022
TMC8	0.790974	2.589669	12.499	1.79E-31	7.13E-30	60.55695
GIMAP8	0.790146	2.150787	14.32616	2.19E-39	1.23E-37	78.64373
EBI3	0.789558	1.531667	14.44018	6.76E-40	3.92E-38	79.80938
COLEC12	0.789096	2.481687	8.992658	4.68E-18	9.73E-17	29.97256
SP140	0.788007	1.207757	17.01373	8.80E-52	9.47E-50	107.0051
SLC37A2	0.787866	1.740641	13.71529	1.10E-36	5.47E-35	72.4676
BTN3A1	0.787347	3.428248	15.21433	2.12E-43	1.46E-41	87.82192
JAK3	0.78592	2.313815	14.77883	2.02E-41	1.28E-39	83.294
SLC7A2	-0.78569	2.860924	-5.0402	6.46E-07	4.31E-06	4.857249
DUSP2	0.783689	2.381721	11.57883	1.06E-27	3.57E-26	51.93955
ICAM1	0.783475	6.070214	7.24279	1.62E-12	2.26E-11	17.41725
CXCL14	0.782888	4.223997	3.631975	0.00031	0.001182	-1.02156
PTGIS	0.781874	2.245067	9.363451	2.44E-19	5.56E-18	32.89084
SIGLEC14	0.781809	1.558177	13.34802	4.35E-35	2.02E-33	68.81398
FAM78A	0.78179	1.581459	19.39897	2.96E-63	7.58E-61	133.2734
NCF1	0.781437	1.202678	17.83959	1.02E-55	1.52E-53	116.0149
ACP5	0.781358	5.084886	9.8255	5.46E-21	1.35E-19	36.64289
SLAMF1	0.779388	1.084885	20.24868	2.10E-67	8.02E-65	142.7734
CFH	0.778747	3.558726	8.692003	4.83E-17	9.53E-16	27.66937
GAL3ST4	0.776323	2.079862	13.97781	7.69E-38	3.97E-36	75.10712
TLR1	0.775512	1.738008	16.16947	8.22E-48	7.12E-46	97.91828
CD19	0.775009	1.219464	10.62221	5.93E-24	1.70E-22	43.3961
SYT7	-0.7749	3.092199	-6.54323	1.46E-10	1.65E-09	13.01058
CD1C	0.774781	1.863248	8.128262	3.30E-15	5.78E-14	23.50948
IL15RA	0.774108	2.669491	14.838	1.09E-41	6.96E-40	83.90616
MARCHF1	0.773769	1.404787	18.20646	1.76E-57	3.07E-55	120.0495
APOL4	0.773542	2.004365	12.26637	1.67E-30	6.34E-29	58.34401
SLC16A14	-0.77223	2.184928	-5.95429	4.85E-09	4.50E-08	9.595482
THBS2	0.771936	4.578566	6.52639	1.62E-10	1.82E-09	12.90913
IL18BP	0.771867	2.602501	14.02418	4.80E-38	2.51E-36	75.57572
SLA2	0.771105	1.318175	17.93447	3.58E-56	5.45E-54	117.0565
ADAM8	0.770344	3.60942	8.522738	1.76E-16	3.34E-15	26.39826
EPB41L2	0.769205	2.300904	14.06688	3.11E-38	1.65E-36	76.00781
OAS2	0.769026	3.52872	8.919087	8.33E-18	1.71E-16	29.40366
BATF2	0.766845	2.48973	10.65444	4.46E-24	1.29E-22	43.67651
ISLR	0.766457	4.509144	7.607657	1.35E-13	2.10E-12	19.86006
ITGB7	0.764749	1.275778	18.89181	8.61E-61	1.82E-58	127.6312
HLA-G	0.764699	2.23099	7.695948	7.28E-14	1.15E-12	20.46558

MAF	0.763026	2.717491	14.40254	9.96E-40	5.69E-38	79.42407
SH2B3	0.76259	2.845941	16.80791	8.28E-51	8.35E-49	104.7774
TNFRSF9	0.762172	1.016038	15.98424	5.99E-47	4.95E-45	95.94393
GYPC	0.761117	2.636068	13.73003	9.46E-37	4.73E-35	72.61524
RAB8B	0.760972	2.870953	14.69619	4.78E-41	2.94E-39	82.44058
PPP1R18	0.760474	4.078173	12.03254	1.53E-29	5.64E-28	56.14267
EMILIN2	0.759563	2.429984	14.15425	1.27E-38	6.93E-37	76.89364
ZEB2	0.75951	1.573296	15.07313	9.36E-43	6.29E-41	86.34828
COL6A2	0.758821	6.009093	7.585108	1.58E-13	2.44E-12	19.70631
CYP27A1	0.758551	4.19811	8.342648	6.78E-16	1.25E-14	25.06654
PLA2G10	-0.75806	1.798316	-6.4157	3.19E-10	3.45E-09	12.24776
ADH1B	0.757503	2.209062	5.605708	3.39E-08	2.78E-07	7.706206
GZMM	0.756527	1.739264	9.485484	9.04E-20	2.11E-18	33.86957
FGD2	0.75596	1.28237	18.55551	3.66E-59	6.85E-57	123.9041
IL33	0.755156	2.737448	7.503171	2.78E-13	4.18E-12	19.15067
POSTN	0.755087	5.188043	6.677912	6.33E-11	7.49E-10	13.82997
FOXP3	0.754789	1.604845	15.35796	4.67E-44	3.33E-42	89.32619
PIK3R5	0.754382	1.492811	17.32919	2.80E-53	3.34E-51	110.4338
PIK3CD	0.752339	2.525328	14.51126	3.25E-40	1.90E-38	80.538
DERL3	0.751326	3.210066	7.670115	8.72E-14	1.38E-12	20.28784
RPS4Y1	-0.75116	2.756139	-2.8454	0.004613	0.012881	-3.5148
ETV7	0.749693	2.455186	11.18663	3.85E-26	1.22E-24	48.38268
EMILIN1	0.749273	4.293937	8.418989	3.83E-16	7.14E-15	25.62844
NT5E	0.748441	3.487537	5.968269	4.48E-09	4.17E-08	9.673311
ADAP2	0.748223	2.494889	16.10227	1.69E-47	1.45E-45	97.20119
SOCS1	0.746178	2.82232	10.68143	3.52E-24	1.03E-22	43.91173
GNG2	0.745365	1.808955	17.68946	5.34E-55	7.23E-53	114.3692
TRAT1	0.745028	0.831549	17.76796	2.25E-55	3.20E-53	115.2293
ANXA1	0.744125	5.805298	6.16646	1.42E-09	1.41E-08	10.79391
TNFAIP8	0.742593	1.955918	15.88347	1.76E-46	1.41E-44	94.87295
SFTPA1	0.741606	7.873766	2.595277	0.009723	0.024658	-4.18489
TMEM156	0.739528	1.556217	9.142246	1.43E-18	3.09E-17	31.13969
STK10	0.737934	2.427265	17.18969	1.29E-52	1.49E-50	108.9155
CD28	0.737713	1.052502	18.80613	2.24E-60	4.69E-58	126.6805
BTN3A2	0.737633	3.232232	12.36847	6.27E-31	2.43E-29	59.31255
HES6	-0.73704	2.854508	-6.20364	1.14E-09	1.14E-08	11.00763
GPSM3	0.736471	4.389274	10.62232	5.92E-24	1.70E-22	43.39713
VIM	0.736425	7.018896	10.41926	3.48E-23	9.50E-22	41.6431
FCRLA	0.736134	1.025961	11.13036	6.41E-26	2.02E-24	47.87846
DDX60	0.73568	2.749064	9.755381	9.80E-21	2.39E-19	36.06547
PODXL2	-0.73524	4.064646	-5.41127	9.63E-08	7.38E-07	6.696145
SERPINA1	0.732527	6.727419	4.63827	4.47E-06	2.54E-05	2.99931
A2M	0.731248	7.09231	7.621152	1.23E-13	1.92E-12	19.95225
IFNG	0.730699	0.680639	13.55949	5.26E-36	2.52E-34	70.91204
LARGE2	-0.73052	3.300421	-7.19025	2.31E-12	3.15E-11	17.07348
JAK2	0.72996	2.022475	15.42567	2.28E-44	1.66E-42	90.03721
P2RY8	0.729724	1.504661	14.041	4.04E-38	2.13E-36	75.74578
SPON1	0.728507	3.102043	7.76822	4.38E-14	7.04E-13	20.96536
C3	0.728154	6.502366	5.496927	6.11E-08	4.83E-07	7.137219
FLI1	0.726665	1.800871	16.40266	6.67E-49	6.04E-47	100.4141
GLS	0.726476	4.00319	7.138385	3.25E-12	4.37E-11	16.73618
UCHL1	-0.72602	3.373471	-3.87979	0.000118	0.000497	-0.11666
THBS1	0.724831	4.99166	6.906602	1.48E-11	1.86E-10	15.25324
MAN1A1	0.72443	3.775137	10.18457	2.63E-22	6.96E-21	39.64345
APOBEC3(	0.723936	4.09329	10.619	6.09E-24	1.74E-22	43.36826
MCOLN2	0.723888	1.325423	14.63253	9.25E-41	5.62E-39	81.78455
MSC	0.723095	2.004417	10.23432	1.72E-22	4.57E-21	40.06489
ADGRF1	0.723051	2.154401	5.130371	4.11E-07	2.81E-06	5.293295

COL8A2	0.722697	2.532625	8.032849	6.60E-15	1.13E-13	22.82655
CRABP2	0.7222	6.089529	3.597424	0.000353	0.001327	-1.14323
TAP2	0.722025	3.020584	12.50734	1.65E-31	6.59E-30	60.63667
CTSB	0.722021	7.488247	12.04425	1.37E-29	5.08E-28	56.25235
SAMD9	0.721571	2.319142	9.650475	2.34E-20	5.58E-19	35.20688
VPREB3	0.720988	1.62857	8.352597	6.30E-16	1.16E-14	25.13955
CCN4	0.720538	1.971551	9.715919	1.36E-20	3.29E-19	35.74174
CHST11	0.71921	2.769685	11.3637	7.69E-27	2.51E-25	49.97952
PMP22	0.719125	4.53971	10.94142	3.50E-25	1.08E-23	46.19658
SAA2	0.719017	1.328563	6.429224	2.94E-10	3.20E-09	12.32803
APOBR	0.717624	2.315087	10.30332	9.49E-23	2.56E-21	40.65151
CBR1	-0.71729	5.15365	-6.06724	2.53E-09	2.43E-08	10.22893
GSTP1	-0.71624	8.297922	-8.92335	8.06E-18	1.66E-16	29.43652
LRRC15	0.714649	1.962081	6.355844	4.59E-10	4.85E-09	11.89411
GREM1	0.713546	1.888421	6.564833	1.28E-10	1.46E-09	13.14107
FASLG	0.713077	0.819023	15.69355	1.33E-45	1.03E-43	92.86081
CCL22	0.712128	2.176443	7.389866	6.02E-13	8.69E-12	18.3903
HLA-A	0.712112	9.005321	8.902487	9.48E-18	1.94E-16	29.27577
PRDM1	0.711204	2.454521	11.34787	8.89E-27	2.89E-25	49.83611
DOK3	0.709291	1.990824	14.09068	2.44E-38	1.31E-36	76.24882
DMBT1	0.709247	2.985044	3.75711	0.000192	0.000768	-0.57169
CD72	0.708966	1.4397	16.00194	4.96E-47	4.14E-45	96.13228
FMNL1	0.708455	2.865088	12.20468	3.00E-30	1.13E-28	57.76096
NRROS	0.707934	1.898328	14.01064	5.51E-38	2.86E-36	75.43876
THEMIS	0.706942	0.777043	17.73187	3.35E-55	4.69E-53	114.8338
FAM49A	0.705964	1.980754	13.05826	7.68E-34	3.40E-32	65.96535
CLEC4E	0.705708	1.169887	11.80616	1.29E-28	4.54E-27	54.03404
ICAM3	0.705297	1.099591	16.75863	1.41E-50	1.42E-48	104.2451
GPR132	0.704719	1.782036	12.06289	1.15E-29	4.28E-28	56.42706
MDFIC	0.703442	3.463179	10.76805	1.64E-24	4.86E-23	44.66917
CSTA	0.703172	2.429085	7.805054	3.37E-14	5.47E-13	21.2215
COL8A1	0.702537	3.717414	7.282287	1.25E-12	1.74E-11	17.677
MPP1	0.702214	2.430931	12.5615	9.79E-32	3.93E-30	61.15525
STAT5A	0.702149	2.727997	16.34005	1.31E-48	1.16E-46	99.74288
RSAD2	0.701643	2.249637	9.07965	2.36E-18	5.01E-17	30.64961
AKNA	0.699866	2.57304	14.00334	5.93E-38	3.07E-36	75.36498
ARHGAP1	0.699849	3.526135	13.2977	7.18E-35	3.28E-33	68.31717
CCL11	0.699743	1.337724	8.638287	7.29E-17	1.42E-15	27.26397
XCL2	0.699023	1.030878	13.02049	1.11E-33	4.91E-32	65.59626
CDH11	0.698945	2.990127	8.28635	1.03E-15	1.88E-14	24.65466
NLRC3	0.698766	1.208541	16.45979	3.60E-49	3.32E-47	101.0274
SPIB	0.698596	0.93262	10.53156	1.31E-23	3.67E-22	42.61041
ANKRD44	0.697688	1.543488	13.47114	1.27E-35	6.06E-34	70.03364
AP1S2	0.697291	2.262558	13.32153	5.67E-35	2.62E-33	68.55231
LRMP	0.696872	1.293348	12.06738	1.10E-29	4.11E-28	56.46919
PYHIN1	0.696568	0.830581	18.59678	2.31E-59	4.37E-57	124.3608
S100A9	0.696461	7.524594	3.88342	0.000116	0.000491	-0.10298
HGD	-0.69623	2.080374	-4.73999	2.77E-06	1.64E-05	3.456185
SIGLEC7	0.695807	1.2145	15.96446	7.40E-47	6.06E-45	95.73348
CLEC2D	0.695027	1.585563	12.80668	9.05E-33	3.84E-31	63.51737
GPT2	-0.69484	3.195749	-7.14432	3.13E-12	4.22E-11	16.77468
CRTAM	0.694471	0.811047	19.9351	7.17E-66	2.25E-63	139.2615
KCNAB2	0.694153	2.168927	13.42963	1.93E-35	9.13E-34	69.62187
COL6A1	0.69209	5.545965	7.184747	2.39E-12	3.26E-11	17.03762
ABCA1	0.691778	2.508515	10.4903	1.88E-23	5.23E-22	42.25423
TMEM150	0.691466	1.3717	12.45552	2.72E-31	1.07E-29	60.14166
SRPX2	0.691384	3.262719	6.326779	5.47E-10	5.70E-09	11.72341
MYLK	0.691052	2.347451	10.52815	1.35E-23	3.78E-22	42.58097

ITGA2	0.690898	3.440176	5.951402	4.94E-09	4.57E-08	9.57941
VSIR	0.69085	3.317941	12.90076	3.61E-33	1.56E-31	64.42999
FGD3	0.689941	1.788117	14.27697	3.62E-39	2.03E-37	78.14198
FURIN	-0.68944	5.798721	-6.73725	4.35E-11	5.24E-10	14.19541
SCNN1A	-0.68939	5.550215	-6.61972	9.10E-11	1.06E-09	13.4742
CLEC12A	0.688954	1.113064	11.75673	2.04E-28	7.08E-27	53.57661
MFNG	0.688391	2.582965	13.30772	6.50E-35	2.97E-33	68.41602
PRR36	-0.68829	2.374125	-8.02184	7.15E-15	1.23E-13	22.74816
TNFSF8	0.688275	1.071646	16.93544	2.07E-51	2.19E-49	106.1568
CIT	-0.68804	2.855977	-5.72697	1.74E-08	1.49E-07	8.352102
AGR2	-0.68703	6.911514	-4.3721	1.49E-05	7.66E-05	1.847065
AKR7A3	-0.68685	1.734785	-4.97539	8.91E-07	5.78E-06	4.548163
ARHGDIB	0.686827	6.684793	9.859836	4.10E-21	1.03E-19	36.92668
CLIC2	0.686224	3.545105	8.062975	5.30E-15	9.16E-14	23.0415
LGALS9	0.685338	3.799894	8.627242	7.93E-17	1.54E-15	27.18084
MSLN	-0.68474	5.302755	-2.6326	0.008729	0.022441	-4.0887
IQGAP2	0.684348	1.811445	9.871349	3.72E-21	9.34E-20	37.02198
ARHGAP1	0.684291	1.141824	17.7388	3.10E-55	4.38E-53	114.9096
IFIT2	0.681581	2.748942	9.526938	6.44E-20	1.52E-18	34.20406
NLRP3	0.681223	1.03027	16.87545	3.97E-51	4.03E-49	105.5076
TLR10	0.680955	0.881454	12.38015	5.61E-31	2.19E-29	59.42359
COL1A1	0.680746	7.631394	4.972705	9.02E-07	5.85E-06	4.535442
RARRES2	0.680384	5.005456	7.014929	7.32E-12	9.52E-11	15.9413
CPNE5	0.680198	1.606033	10.6585	4.31E-24	1.25E-22	43.71189
ZBED2	0.680145	1.340834	9.989369	1.38E-21	3.51E-20	38.00329
BPIFB2	-0.67896	1.239462	-4.07439	5.35E-05	0.000244	0.633342
GSTA1	-0.67831	2.906403	-3.61778	0.000327	0.001239	-1.07167
TPSB2	0.677254	2.881459	5.541164	4.81E-08	3.86E-07	7.367413
RTP4	0.677228	2.324915	8.92738	7.81E-18	1.61E-16	29.46762
PLA2G4A	-0.67646	3.554515	-4.92978	1.11E-06	7.10E-06	4.332808
PRRX1	0.676453	2.482158	8.903197	9.43E-18	1.93E-16	29.28124
HBB	0.676362	3.95214	4.197378	3.18E-05	0.000152	1.125081
FABP3	0.676352	3.415493	5.335927	1.43E-07	1.07E-06	6.313308
GAB3	0.676321	1.248882	17.32494	2.93E-53	3.48E-51	110.3875
FBN1	0.675944	2.845329	7.814476	3.16E-14	5.14E-13	21.28716
KLHDC7B	0.675833	1.371442	8.82694	1.71E-17	3.43E-16	28.69595
SLFN11	0.675826	2.98198	9.097398	2.05E-18	4.37E-17	30.78832
UBASH3A	0.675512	0.905811	18.12418	4.39E-57	7.31E-55	119.143
GM2A	0.675358	4.758225	12.36299	6.61E-31	2.56E-29	59.2604
LPAR6	0.675019	2.340119	12.51971	1.47E-31	5.86E-30	60.75499
IL1RN	0.674677	2.494859	7.70285	6.94E-14	1.10E-12	20.51316
PIP4K2A	0.674548	3.379703	13.07498	6.51E-34	2.89E-32	66.12882
CXCL1	0.674437	3.577748	4.985967	8.45E-07	5.53E-06	4.598363
KIAA1324	-0.67422	2.763796	-4.59274	5.51E-06	3.08E-05	2.797771
CD22	0.673454	1.186004	10.52762	1.36E-23	3.79E-22	42.57638
CDA	0.673286	3.026734	4.270493	2.33E-05	0.000114	1.423879
FNBP1	0.673027	3.144892	13.90459	1.62E-37	8.21E-36	74.36865
APOL1	0.672097	5.346711	6.939472	1.19E-11	1.53E-10	15.46108
CABYR	-0.67201	1.577096	-6.16261	1.45E-09	1.44E-08	10.77183
LY96	0.671994	4.295861	9.451103	1.20E-19	2.77E-18	33.59292
KRTCAP3	-0.67005	4.924951	-7.44539	4.13E-13	6.08E-12	18.76172
DPP4	0.669844	4.066519	4.542588	6.94E-06	3.80E-05	2.577879
IFIH1	0.669534	3.115318	10.71415	2.64E-24	7.74E-23	44.1974
IL6	0.669483	1.795764	6.840367	2.26E-11	2.79E-10	14.83692
SLC2A3	0.669069	3.176754	7.42978	4.59E-13	6.72E-12	18.65709
MMP1	0.668748	4.019874	3.51008	0.000487	0.001778	-1.44586
COL14A1	0.668167	2.434032	6.77102	3.52E-11	4.26E-10	14.4046
MCUB	0.667959	2.585243	11.85392	8.23E-29	2.95E-27	54.47702

LAP3	0.667815	5.015944	14.19316	8.55E-39	4.71E-37	77.28897
KCNA3	0.66722	1.291948	9.740908	1.10E-20	2.68E-19	35.94664
FBLN1	0.666463	4.248273	7.135473	3.32E-12	4.45E-11	16.7173
SLC9A9	0.666265	1.549369	14.88595	6.63E-42	4.28E-40	84.40295
COL1A2	0.66438	6.9437	5.790034	1.23E-08	1.07E-07	8.692779
STK17A	0.663706	3.544212	11.22024	2.84E-26	9.07E-25	48.68459
CD1E	0.663483	1.185143	8.26058	1.25E-15	2.25E-14	24.46683
SFRP4	0.663329	3.711665	5.226906	2.51E-07	1.80E-06	5.76784
INHBA	0.663316	2.613687	7.124615	3.56E-12	4.77E-11	16.64696
MICB	0.663234	2.194771	10.55858	1.03E-23	2.92E-22	42.84421
GNB4	0.663212	2.456903	11.32904	1.06E-26	3.42E-25	49.66576
PSMB10	0.661202	4.086027	9.923551	2.40E-21	6.06E-20	37.45505
NPW	-0.66107	1.06944	-5.75639	1.48E-08	1.28E-07	8.510627
LIMD2	0.660966	2.77287	9.739255	1.12E-20	2.72E-19	35.93307
DHRS9	0.659934	1.497804	6.7615	3.73E-11	4.52E-10	14.34555
FCRL5	0.658905	1.004676	10.26937	1.27E-22	3.41E-21	40.36253
RGS10	0.658391	4.471532	9.265169	5.37E-19	1.19E-17	32.10912
STEAP1	0.657882	3.444289	5.581693	3.87E-08	3.14E-07	7.579744
CCN1	0.656337	5.858335	6.5486	1.42E-10	1.60E-09	13.04299
RESF1	0.655761	3.111665	10.11673	4.69E-22	1.22E-20	39.07107
FAP	0.654958	1.886105	8.923965	8.02E-18	1.65E-16	29.44128
GXYLT2	0.65407	1.770766	9.228725	7.20E-19	1.58E-17	31.82075
MET	0.653777	4.617763	4.617965	4.91E-06	2.77E-05	2.909201
GIMAP1	0.653297	1.307004	16.67302	3.58E-50	3.47E-48	103.3215
TMIGD3	0.653101	1.408127	12.66895	3.46E-32	1.42E-30	62.18754
DCBLD2	0.651992	3.196257	6.456095	2.50E-10	2.74E-09	12.488
MCEMP1	0.650065	1.890757	5.701899	2.00E-08	1.70E-07	8.21755
GFPT2	0.648775	1.968524	9.10936	1.86E-18	3.99E-17	30.88192
RGS18	0.648616	0.958539	15.98712	5.81E-47	4.82E-45	95.97452
LILRA5	0.648283	1.458888	10.86619	6.85E-25	2.08E-23	45.53194
PARP15	0.647567	1.213644	10.42422	3.34E-23	9.12E-22	41.68567
NQO1	-0.64703	5.673455	-4.40194	1.31E-05	6.80E-05	1.973132
NFATC2	0.646984	1.876832	13.34926	4.30E-35	2.00E-33	68.82626
ENO3	-0.64617	1.441551	-6.40129	3.49E-10	3.74E-09	12.16237
GPR87	0.64489	1.877074	4.357584	1.59E-05	8.10E-05	1.786052
TACC2	-0.64478	2.632408	-7.46433	3.62E-13	5.39E-12	18.889
IFFO1	0.64452	1.61443	13.60328	3.39E-36	1.66E-34	71.34846
SSPN	0.644449	1.678855	11.06044	1.20E-25	3.74E-24	47.25396
HPGDS	0.643763	1.398652	9.066169	2.62E-18	5.55E-17	30.54438
BICC1	0.643727	1.778116	8.920122	8.26E-18	1.70E-16	29.41164
IGFBP2	-0.64354	4.337311	-4.07306	5.38E-05	0.000245	0.628098
CD40LG	0.643289	0.944739	14.11376	1.93E-38	1.04E-36	76.48282
RTN1	0.643083	0.990716	15.32655	6.51E-44	4.58E-42	88.99679
SCGB3A1	0.643034	5.366378	2.359085	0.018695	0.042982	-4.7625
OGFRL1	0.642789	2.54746	10.64011	5.06E-24	1.47E-22	43.55178
PDGFRA	0.642562	2.281425	8.668594	5.78E-17	1.13E-15	27.49246
IL18	0.642561	3.056576	8.33681	7.08E-16	1.30E-14	25.02373
PAPLN	0.641605	2.094433	10.16258	3.17E-22	8.36E-21	39.45762
RHOH	0.641462	1.380305	12.41723	3.93E-31	1.54E-29	59.77653
LGALS1	0.641271	7.357867	6.638839	8.08E-11	9.48E-10	13.59081
HHLA2	0.640432	1.555221	3.827792	0.000145	0.0006	-0.31121
PDLIM3	0.639671	2.071458	8.955704	6.26E-18	1.29E-16	29.68639
CD1A	0.639598	1.648277	5.147943	3.76E-07	2.59E-06	5.379086
SLFN5	0.637719	3.500717	9.568714	4.58E-20	1.08E-18	34.54217
P2RY12	0.637472	0.768253	15.11574	5.98E-43	4.06E-41	86.79249
SEMA7A	0.637397	2.209204	8.118562	3.54E-15	6.20E-14	23.43977
TIMP2	0.637023	5.624962	8.113775	3.66E-15	6.41E-14	23.40539
SIRPB2	0.636996	1.085371	16.44335	4.30E-49	3.95E-47	100.8508

ARHGAP3	0.636989	2.218154	10.18537	2.61E-22	6.92E-21	39.65021
VCAN	0.636981	3.758504	5.798313	1.17E-08	1.03E-07	8.737751
MMP13	0.636872	2.084548	3.905636	0.000107	0.000454	-0.01903
SPARCL1	0.636122	5.320653	7.44706	4.08E-13	6.02E-12	18.77296
FIBIN	0.634541	2.109124	7.592315	1.50E-13	2.32E-12	19.75541
LOX	0.634531	2.836738	7.300542	1.10E-12	1.56E-11	17.79745
GFI1	0.632952	1.02217	15.37009	4.11E-44	2.94E-42	89.45352
ALDH1A3	0.632952	1.923929	7.881429	1.96E-14	3.25E-13	21.75561
STAT4	0.632358	1.484321	13.38779	2.93E-35	1.37E-33	69.2074
HVCN1	0.631525	1.743707	15.8673	2.09E-46	1.66E-44	94.70136
FLNA	0.631365	6.514301	7.380208	6.43E-13	9.25E-12	18.32592
COL5A2	0.630308	4.489915	5.872209	7.74E-09	6.96E-08	9.141628
FCGBP	0.630237	2.164204	5.12262	4.27E-07	2.91E-06	5.25554
ADAM12	0.630233	1.670273	7.244429	1.61E-12	2.23E-11	17.42801
PDE4B	0.630119	1.736895	11.48723	2.48E-27	8.22E-26	51.10237
CCRL2	0.629883	1.670015	11.25524	2.07E-26	6.66E-25	48.99964
MT2A	0.629687	6.074648	5.468182	7.12E-08	5.56E-07	6.988516
STAP1	0.629541	0.952596	12.90943	3.31E-33	1.44E-31	64.51423
SMOC1	-0.62946	1.462149	-4.42544	1.18E-05	6.17E-05	2.072957
ADAMTSL	0.629021	2.098569	8.555672	1.37E-16	2.61E-15	26.64412
TXNIP	0.628887	7.409435	7.325265	9.33E-13	1.33E-11	17.96095
AHNAK	0.62888	4.780559	6.56219	1.30E-10	1.48E-09	13.12509
SPINK1	-0.62856	4.259715	-2.39666	0.016903	0.03951	-4.6742
CTSC	0.628356	4.755726	10.09146	5.81E-22	1.50E-20	38.85844
CALHM2	0.628141	2.516051	13.21218	1.68E-34	7.57E-33	67.47477
TRANK1	0.627777	2.210699	11.79777	1.39E-28	4.88E-27	53.95628
IRAK3	0.627204	1.824389	8.217721	1.71E-15	3.06E-14	24.15544
ARID5A	0.626608	2.975973	12.95177	2.19E-33	9.54E-32	64.92618
RAP1GAP	-0.62658	3.58574	-5.20153	2.86E-07	2.03E-06	5.642327
SERPINE1	0.626443	4.547119	5.144663	3.82E-07	2.63E-06	5.363051
XAF1	0.624985	1.503221	10.13111	4.15E-22	1.08E-20	39.19216
CCDC69	0.624833	3.178542	8.95004	6.54E-18	1.35E-16	29.64259
NCR3	0.624724	0.812394	14.6069	1.21E-40	7.26E-39	81.52071
PLN	0.624138	2.014142	7.121852	3.63E-12	4.85E-11	16.62907
FMNL3	0.624045	2.080533	15.90049	1.47E-46	1.18E-44	95.05373
AKR1B10	-0.62271	2.167306	-2.62339	0.008966	0.022984	-4.11256
SUCNR1	0.621902	1.203168	7.748033	5.05E-14	8.09E-13	20.82539
AOC3	0.621621	3.329964	6.868077	1.89E-11	2.36E-10	15.01068
IRF5	0.620659	2.436888	10.47448	2.16E-23	5.97E-22	42.11797
RGL1	0.619253	3.133395	9.284214	4.61E-19	1.03E-17	32.26015
STARD10	-0.61876	4.686905	-7.79133	3.72E-14	6.02E-13	21.12595
FYN	0.617991	2.728392	10.6254	5.76E-24	1.66E-22	43.42384
SLC2A5	0.617891	1.152212	10.8224	1.01E-24	3.04E-23	45.14637
TNS3	0.617155	3.746457	10.90495	4.85E-25	1.48E-23	45.87403
FGFBP1	0.615914	2.044235	4.279599	2.24E-05	0.00011	1.461427
TBX21	0.615571	0.841322	11.66762	4.67E-28	1.60E-26	52.75484
EOMES	0.614634	0.726543	15.12175	5.62E-43	3.83E-41	86.85519
APOBEC3I	0.614253	1.717902	11.78052	1.63E-28	5.71E-27	53.79665
TNFAIP6	0.613128	1.958666	7.971946	1.02E-14	1.74E-13	22.3939
ACKR1	0.61273	2.314855	5.659157	2.54E-08	2.11E-07	7.989388
ATP1B1	-0.61247	7.422524	-6.53841	1.51E-10	1.70E-09	12.98154
LRP1	0.610819	4.13056	8.16071	2.60E-15	4.60E-14	23.74315
SERPINF1	0.610617	5.173335	6.087616	2.25E-09	2.18E-08	10.34433
LTBP2	0.609561	4.424413	6.543663	1.46E-10	1.65E-09	13.0132
RAB31	0.609503	4.574417	8.793201	2.22E-17	4.44E-16	28.43817
FILIP1L	0.608969	3.062743	7.818468	3.07E-14	5.00E-13	21.31501
CTSO	0.608937	4.12767	10.01751	1.09E-21	2.78E-20	38.23843
RNASE2	0.608883	1.388951	9.484611	9.11E-20	2.12E-18	33.86253

GPR18	0.60812	0.695174	11.23438	2.50E-26	8.03E-25	48.81185
ALDH3A1	-0.60805	2.016703	-3.25136	0.001224	0.004022	-2.30059
ZBP1	0.607815	1.002414	12.70044	2.55E-32	1.06E-30	62.49092
NABP1	0.607465	1.820861	9.836435	4.99E-21	1.24E-19	36.73319
MEF2C	0.607256	2.029082	10.15106	3.50E-22	9.18E-21	39.36037
NFE2L3	0.606905	3.348537	7.45107	3.97E-13	5.87E-12	18.79987
SH3BGRL	0.606354	5.475394	12.39029	5.09E-31	1.99E-29	59.52003
CAV1	0.606258	4.471684	5.427504	8.84E-08	6.82E-07	6.779271
MMP19	0.605497	2.075487	8.560665	1.32E-16	2.52E-15	26.68146
ITGAV	0.6052	4.537344	8.27088	1.16E-15	2.10E-14	24.54185
PTPRO	0.604308	0.877691	17.31182	3.38E-53	3.97E-51	110.2445
ANXA6	0.602815	4.454492	10.72484	2.40E-24	7.07E-23	44.29084
CDH3	0.602704	3.713885	4.521404	7.64E-06	4.15E-05	2.485669
SMIM22	-0.60233	4.362008	-5.66661	2.43E-08	2.04E-07	8.029059
EFEMP1	0.600854	4.523156	6.278953	7.28E-10	7.48E-09	11.44398
AQP1	0.600551	5.910219	3.817164	0.000152	0.000622	-0.35066
SYK	0.600325	3.671068	10.58542	8.18E-24	2.33E-22	43.07674
SLC34A2	0.599962	7.908325	3.493564	0.000518	0.001879	-1.50229
RENBP	0.599792	3.03099	7.5232	2.42E-13	3.67E-12	19.28604
DPEP2	0.599352	1.242116	12.48931	1.96E-31	7.81E-30	60.46434
TM7SF2	-0.59916	3.37626	-8.00428	8.11E-15	1.39E-13	22.62326
PDLIM4	0.598545	2.512679	6.011491	3.50E-09	3.30E-08	9.914992
NTN4	0.598433	3.515579	7.164191	2.74E-12	3.72E-11	16.90377
POU2F2	0.597818	1.236479	12.37521	5.88E-31	2.29E-29	59.37658
OLFML2B	0.597679	3.218988	7.456713	3.82E-13	5.67E-12	18.83778
S1PR4	0.597581	2.054717	9.697246	1.59E-20	3.83E-19	35.58888
C1orf54	0.597388	2.61984	12.7726	1.26E-32	5.30E-31	63.18755
TGFB1	0.596818	4.498593	9.068079	2.58E-18	5.47E-17	30.55928
WNT2	0.596584	1.787339	8.075575	4.84E-15	8.39E-14	23.1316
IRS2	-0.59578	2.749094	-6.26274	8.02E-10	8.15E-09	11.34966
TNF	0.595676	0.996417	10.26181	1.36E-22	3.62E-21	40.29828
LXN	0.595526	3.567791	10.12088	4.53E-22	1.18E-20	39.10595
MRPS26	-0.59487	5.158824	-9.25284	5.93E-19	1.31E-17	32.0115
CAPG	0.59402	6.234575	8.102156	3.99E-15	6.96E-14	23.322
SPRR1B	0.593973	1.486346	3.351328	0.000864	0.002955	-1.97774
TFCP2L1	-0.59348	2.690618	-5.40792	9.80E-08	7.50E-07	6.679024
CD93	0.593458	3.635064	8.046967	5.96E-15	1.03E-13	22.9272
EGR3	0.593134	1.311085	9.367939	2.35E-19	5.37E-18	32.92668
NMB	-0.59261	3.051777	-6.9731	9.61E-12	1.23E-10	15.67453
FASN	-0.59229	4.97454	-5.95026	4.97E-09	4.59E-08	9.573087
RASSF5	0.592257	2.934275	10.68844	3.31E-24	9.68E-23	43.97291
TYMP	0.592225	5.301707	7.729163	5.77E-14	9.21E-13	20.69481
NCEH1	0.591784	3.362059	8.78955	2.28E-17	4.55E-16	28.41032
ISM1	0.591272	1.88318	7.124453	3.57E-12	4.77E-11	16.64591
SECTM1	0.590215	3.504682	6.534344	1.55E-10	1.74E-09	12.95702
GAS1	0.589886	1.765754	7.237945	1.68E-12	2.33E-11	17.38547
ANOS1	0.58978	2.215875	6.911325	1.43E-11	1.81E-10	15.28305
ENPP2	0.589749	3.063008	8.139654	3.03E-15	5.34E-14	23.59143
TESPA1	0.589135	0.851363	17.17466	1.52E-52	1.74E-50	108.7521
N4BP2L1	0.589092	1.721807	12.45827	2.65E-31	1.05E-29	60.16786
PAG1	0.588947	2.297562	8.138927	3.05E-15	5.36E-14	23.58621
ERAP1	0.588634	3.488265	10.63988	5.07E-24	1.47E-22	43.54981
CASS4	0.588155	1.270055	12.59552	7.04E-32	2.87E-30	61.48158
PALLD	0.588143	3.817286	8.160838	2.60E-15	4.60E-14	23.74407
CD33	0.588132	0.930844	17.52064	3.42E-54	4.42E-52	112.5226
DDR2	0.587875	1.820751	9.379363	2.14E-19	4.91E-18	33.01795
CCL3L3	0.587774	1.179661	10.06122	7.51E-22	1.93E-20	38.60452
FHL1	0.587563	2.346531	6.345029	4.90E-10	5.15E-09	11.83051

CMTM3	0.587479	4.093757	8.247547	1.37E-15	2.47E-14	24.37201
IL27RA	0.587472	3.592019	7.45458	3.87E-13	5.74E-12	18.82344
CSF2	0.586864	1.331208	6.495995	1.96E-10	2.18E-09	12.72656
PCED1B	0.586377	2.813739	8.551994	1.41E-16	2.68E-15	26.61663
AMBP	-0.58638	1.177987	-4.59881	5.36E-06	3.00E-05	2.824536
SH2D2A	0.586218	1.847221	10.30291	9.52E-23	2.56E-21	40.64802
CKB	-0.58616	4.834245	-4.76989	2.41E-06	1.44E-05	3.592224
UBA7	0.585931	3.703869	9.202231	8.89E-19	1.93E-17	31.61161
SPRED1	0.585788	3.345694	9.704082	1.50E-20	3.62E-19	35.64482
PLCB2	0.585612	2.331849	8.622729	8.21E-17	1.59E-15	27.1469
FAM20A	0.585549	3.158257	6.781144	3.30E-11	4.01E-10	14.46749

Table S4: 113 DEGs related to survival time in TCGA profiles

Table S4										
	**coef**	**se**	**z**	**p**	**HR**	**HRse**	**HRz**	**HRp**	**HRCILL**	**HRCIUL**
ABI3BP	-0.459	0.15	-3.063	0.002	0.632	0.095	-3.887	0	0.494	0.808
ACAP1	-0.395	0.15	-2.633	0.008	0.674	0.101	-3.229	0.001	0.527	0.862
ADA2	-0.457	0.15	-3.035	0.002	0.633	0.095	-3.847	0	0.495	0.811
ANKRD44	-0.428	0.151	-2.839	0.005	0.652	0.098	-3.543	0	0.509	0.835
ANOS1	-0.403	0.149	-2.702	0.007	0.669	0.1	-3.328	0.001	0.523	0.854
AREG	0.444	0.149	2.973	0.003	1.559	0.233	2.401	0.016	1.219	1.993
ARHGAP15	-0.604	0.152	-3.979	0	0.547	0.083	-5.463	0	0.426	0.702
ARHGAP25	-0.488	0.151	-3.24	0.001	0.614	0.092	-4.176	0	0.479	0.786
ARHGAP30	-0.427	0.151	-2.83	0.005	0.653	0.098	-3.529	0	0.509	0.836
ARHGAP9	-0.523	0.151	-3.455	0.001	0.593	0.09	-4.54	0	0.462	0.76
ARHGEF6	-0.45	0.152	-2.963	0.003	0.638	0.097	-3.743	0	0.497	0.819
BTK	-0.521	0.151	-3.449	0.001	0.594	0.09	-4.525	0	0.463	0.762
CCR2	-0.576	0.153	-3.768	0	0.562	0.086	-5.096	0	0.437	0.723
CD2	-0.409	0.149	-2.744	0.006	0.664	0.099	-3.391	0.001	0.52	0.849
CD28	-0.398	0.15	-2.654	0.008	0.672	0.101	-3.26	0.001	0.525	0.86
CD300LF	-0.442	0.149	-2.957	0.003	0.643	0.096	-3.718	0	0.503	0.822
CD33	-0.428	0.149	-2.869	0.004	0.652	0.097	-3.581	0	0.51	0.833
CD37	-0.388	0.149	-2.6	0.009	0.678	0.101	-3.176	0.001	0.531	0.867
CD40LG	-0.422	0.151	-2.802	0.005	0.656	0.099	-3.487	0	0.512	0.84
CD5	-0.428	0.15	-2.853	0.004	0.652	0.098	-3.561	0	0.509	0.834
CD74	-0.4	0.149	-2.686	0.007	0.67	0.1	-3.303	0.001	0.525	0.856
CD79B	-0.398	0.149	-2.668	0.008	0.672	0.1	-3.276	0.001	0.526	0.859
CH25H	-0.413	0.15	-2.761	0.006	0.662	0.099	-3.419	0.001	0.517	0.846
CHRDL1	-0.56	0.152	-3.683	0	0.571	0.087	-4.938	0	0.445	0.733
CIITA	-0.391	0.15	-2.603	0.009	0.676	0.102	-3.185	0.001	0.528	0.866
CLEC10A	-0.5	0.15	-3.326	0.001	0.607	0.091	-4.315	0	0.474	0.777
CLEC2D	-0.4	0.15	-2.659	0.008	0.671	0.101	-3.269	0.001	0.524	0.859
CLEC4A	-0.451	0.151	-2.994	0.003	0.637	0.096	-3.782	0	0.497	0.816
CXorf21	-0.641	0.152	-4.218	0	0.527	0.08	-5.91	0	0.411	0.677
CYP27A1	-0.393	0.149	-2.643	0.008	0.675	0.1	-3.238	0.001	0.528	0.862
DPEP2	-0.446	0.149	-2.988	0.003	0.64	0.096	-3.764	0	0.501	0.818
DUSP2	-0.402	0.15	-2.68	0.007	0.669	0.1	-3.298	0.001	0.523	0.856
EREG	0.446	0.15	2.983	0.003	1.563	0.234	2.406	0.016	1.222	1.999
EVI2B	-0.397	0.151	-2.639	0.008	0.672	0.101	-3.24	0.001	0.525	0.861
FCRLA	-0.449	0.15	-2.997	0.003	0.638	0.096	-3.783	0	0.499	0.817
FDCSP	-0.444	0.149	-2.982	0.003	0.641	0.096	-3.754	0	0.502	0.819
FGD2	-0.401	0.15	-2.673	0.008	0.67	0.1	-3.288	0.001	0.523	0.857
FGFBP1	0.407	0.148	2.742	0.006	1.502	0.223	2.252	0.024	1.177	1.918
FGL2	-0.415	0.152	-2.736	0.006	0.661	0.1	-3.39	0.001	0.515	0.848
FLI1	-0.415	0.15	-2.773	0.006	0.66	0.099	-3.436	0.001	0.516	0.845
FMNL3	-0.414	0.151	-2.752	0.006	0.661	0.099	-3.409	0.001	0.516	0.846
FNBP1	-0.409	0.149	-2.742	0.006	0.664	0.099	-3.388	0.001	0.52	0.849
FOLR2	-0.398	0.149	-2.673	0.008	0.671	0.1	-3.284	0.001	0.526	0.858
GAB3	-0.555	0.152	-3.647	0	0.574	0.087	-4.876	0	0.447	0.737
GIMAP4	-0.442	0.15	-2.943	0.003	0.643	0.097	-3.701	0	0.502	0.823
GIMAP6	-0.456	0.151	-3.025	0.002	0.634	0.096	-3.832	0	0.495	0.812
GIMAP7	-0.525	0.151	-3.469	0.001	0.592	0.09	-4.561	0	0.461	0.759
GIMAP8	-0.568	0.152	-3.731	0	0.567	0.086	-5.024	0	0.441	0.728
GPR174	-0.429	0.151	-2.836	0.005	0.651	0.098	-3.542	0	0.508	0.835
HLA-DMA	-0.445	0.149	-2.982	0.003	0.641	0.096	-3.755	0	0.502	0.819
HLA-DMB	-0.467	0.15	-3.118	0.002	0.627	0.094	-3.974	0	0.49	0.802
HLA-DOA	-0.389	0.15	-2.6	0.009	0.678	0.101	-3.178	0.001	0.53	0.867
HLA-DOB	-0.436	0.15	-2.916	0.004	0.646	0.097	-3.656	0	0.506	0.827
HLA-DPB1	-0.412	0.149	-2.766	0.006	0.663	0.099	-3.422	0.001	0.519	0.846
HLA-DQB1	-0.407	0.15	-2.719	0.007	0.666	0.1	-3.355	0.001	0.521	0.852
HLA-DRB1	-0.468	0.149	-3.133	0.002	0.626	0.094	-3.996	0	0.49	0.801

HLA-DRB5	-0.401	0.148	-2.699	0.007	0.67	0.099	-3.32	0.001	0.525	0.855
ICAM3	-0.417	0.15	-2.779	0.005	0.659	0.099	-3.448	0.001	0.514	0.843
IGSF6	-0.401	0.15	-2.67	0.008	0.67	0.101	-3.285	0.001	0.523	0.857
IKZF1	-0.439	0.15	-2.922	0.003	0.645	0.097	-3.668	0	0.503	0.825
IKZF3	-0.461	0.151	-3.062	0.002	0.631	0.095	-3.891	0	0.492	0.808
IL16	-0.433	0.151	-2.874	0.004	0.648	0.098	-3.598	0	0.506	0.831
IRF8	-0.493	0.151	-3.256	0.001	0.611	0.092	-4.208	0	0.476	0.784
ITGA4	-0.443	0.151	-2.935	0.003	0.642	0.097	-3.694	0	0.501	0.823
JAML	-0.587	0.154	-3.817	0	0.556	0.085	-5.191	0	0.432	0.716
KCTD12	-0.408	0.15	-2.722	0.006	0.665	0.1	-3.361	0.001	0.52	0.851
KIAA1324	-0.423	0.15	-2.826	0.005	0.655	0.098	-3.519	0	0.512	0.838
LPAR6	-0.391	0.149	-2.619	0.009	0.676	0.101	-3.204	0.001	0.529	0.865
LRMP	-0.399	0.15	-2.659	0.008	0.671	0.101	-3.267	0.001	0.524	0.859
MCOLN2	-0.416	0.15	-2.778	0.005	0.66	0.099	-3.446	0.001	0.515	0.844
MFNG	-0.437	0.151	-2.896	0.004	0.646	0.098	-3.632	0	0.504	0.828
MPEG1	-0.395	0.15	-2.627	0.009	0.674	0.101	-3.222	0.001	0.526	0.863
MS4A1	-0.576	0.151	-3.809	0	0.562	0.085	-5.152	0	0.438	0.721
N4BP2L1	-0.446	0.15	-2.969	0.003	0.64	0.096	-3.742	0	0.5	0.82
NCF4	-0.402	0.149	-2.698	0.007	0.669	0.1	-3.322	0.001	0.523	0.855
NCKAP1L	-0.401	0.15	-2.665	0.008	0.67	0.101	-3.279	0.001	0.523	0.858
NCR3	-0.448	0.15	-2.991	0.003	0.639	0.096	-3.773	0	0.499	0.817
NFATC2	-0.407	0.151	-2.699	0.007	0.665	0.1	-3.331	0.001	0.519	0.853
NLRC3	-0.513	0.151	-3.39	0.001	0.599	0.091	-4.43	0	0.467	0.768
NLRP3	-0.414	0.15	-2.758	0.006	0.661	0.099	-3.417	0.001	0.516	0.846
P2RY12	-0.444	0.151	-2.929	0.003	0.642	0.097	-3.686	0	0.5	0.823
P2RY13	-0.486	0.152	-3.203	0.001	0.615	0.093	-4.126	0	0.479	0.789
P2RY8	-0.465	0.151	-3.087	0.002	0.628	0.095	-3.93	0	0.49	0.805
PARP15	-0.471	0.151	-3.115	0.002	0.624	0.094	-3.979	0	0.487	0.801
PDE4B	-0.52	0.152	-3.428	0.001	0.594	0.09	-4.497	0	0.463	0.763
PIK3CD	-0.562	0.151	-3.724	0	0.57	0.086	-4.997	0	0.445	0.731
PIK3CG	-0.433	0.153	-2.835	0.005	0.649	0.099	-3.547	0	0.505	0.834
PPP1R16B	-0.392	0.15	-2.615	0.009	0.675	0.101	-3.202	0.001	0.528	0.865
PREX1	-0.463	0.151	-3.063	0.002	0.63	0.095	-3.894	0	0.491	0.807
PRKCB	-0.511	0.151	-3.377	0.001	0.6	0.091	-4.408	0	0.468	0.769
PSTPIP1	-0.512	0.152	-3.372	0.001	0.599	0.091	-4.403	0	0.467	0.769
PTPRC	-0.461	0.151	-3.063	0.002	0.63	0.095	-3.892	0	0.492	0.808
PYHIN1	-0.419	0.151	-2.782	0.005	0.658	0.099	-3.456	0.001	0.513	0.843
RASSF2	-0.432	0.151	-2.854	0.004	0.649	0.098	-3.571	0	0.506	0.833
RCSD1	-0.526	0.152	-3.466	0.001	0.591	0.09	-4.562	0	0.46	0.758
RHOH	-0.389	0.149	-2.605	0.009	0.678	0.101	-3.185	0.001	0.53	0.866
RTN1	-0.44	0.151	-2.914	0.004	0.644	0.097	-3.66	0	0.503	0.826
SCIMP	-0.489	0.151	-3.237	0.001	0.613	0.093	-4.174	0	0.479	0.786
SIT1	-0.4	0.15	-2.678	0.007	0.67	0.1	-3.293	0.001	0.524	0.857
SLAMF1	-0.52	0.152	-3.426	0.001	0.595	0.09	-4.492	0	0.463	0.763
SPRR1B	0.525	0.15	3.493	0	1.69	0.254	2.718	0.007	1.32	2.164
STAP1	-0.544	0.151	-3.607	0	0.58	0.088	-4.794	0	0.453	0.744
STEAP1	0.586	0.151	3.876	0	1.796	0.271	2.934	0.003	1.401	2.303
TBC1D10C	-0.399	0.15	-2.652	0.008	0.671	0.101	-3.258	0.001	0.524	0.86
TESPA1	-0.507	0.151	-3.364	0.001	0.603	0.091	-4.38	0	0.47	0.772
TLR10	-0.534	0.152	-3.519	0	0.586	0.089	-4.652	0	0.457	0.752
TLR2	-0.392	0.15	-2.617	0.009	0.675	0.101	-3.205	0.001	0.528	0.864
TLR7	-0.527	0.153	-3.448	0.001	0.59	0.09	-4.54	0	0.459	0.759
TMEM273	-0.509	0.152	-3.358	0.001	0.601	0.091	-4.378	0	0.469	0.771
TNFSF8	-0.485	0.152	-3.19	0.001	0.616	0.094	-4.106	0	0.479	0.791
TRAF3IP3	-0.445	0.152	-2.934	0.003	0.641	0.097	-3.695	0	0.5	0.822
ZAP70	-0.397	0.151	-2.632	0.008	0.672	0.101	-3.231	0.001	0.525	0.862
ZEB2	-0.504	0.151	-3.333	0.001	0.604	0.091	-4.333	0	0.471	0.775

Table S5: 326 genes with differential mutation frequency between high- and low-score groups

Table S5	
gene name	p
ABCA2	0.006557
ABCA6	0.03826
ABCB1	0.015799
ABCG1	0.014609
ADAL	0.030328
ADAMTS12	0.038447
AGAP1	0.036362
AGMO	0.046097
AIFM3	0.030328
AK5	0.020827
AKAP8L	0.030328
AKT2	0.030328
AL445989.1	0.011778
ALDH1A1	0.014982
ALDH8A1	0.020827
ALMS1	0.043023
ALPK2	0.01317
ANKRD34A	0.037404
AQR	0.031846
ARHGAP32	0.047461
ARMC5	0.002128
ASTN1	0.007922
ASXL2	0.032549
ASXL3	0.035261
ATM	0.003621
ATP8A1	0.036654
ATP9B	0.007386
BACH2	0.037491
BAI1	0.023596
BBS9	0.037404
BEND2	0.019329
BEND4	0.007386
BLK	0.020827
BMPER	0.010261
BTK	0.027379
C1GALT1	0.014982
C2CD5	0.020281
C6	0.047461
C9	0.010261
CACNA1E	0.009407
CACNB1	0.030328
CAND1	0.036362
CAPN10	0.014982
CARD11	0.030766
CCDC135	0.036362
CCDC40	0.001086
CCDC73	0.037491
CD163L1	0.046097
CDH18	0.043422
CDH22	0.010261
CDH4	0.003754
CENPF	0.001756
CEP170B	0.014982
CEP192	0.045338
CHD6	0.049383
CHD7	1.68E-05

CHN1	0.014982
COL11A1	0.001239
COL19A1	0.005358
COL1A2	0.049383
COL24A1	0.024686
COL28A1	0.006557
CPS1	0.048255
CPSF1	0.011778
CRB1	0.013769
CREBBP	0.005435
CSMD1	0.002859
CT47B1	0.019329
CYLC1	0.008975
CYP11B1	0.014647
DARC	0.003644
DDX27	0.037404
DDX3X	0.029701
DENND1A	0.031846
DGKG	0.005788
DIDO1	0.031094
DLD	0.014982
DNAH11	0.012207
DNAH5	2.35E-06
DNMBP	0.037404
DOCK3	0.017069
DOCK5	0.032549
DOCK8	0.020827
DSCAM	0.017107
DSG4	0.020258
DYSF	0.039192
EDIL3	0.047461
EGFR	0.023615
EML4	0.019794
EMR1	0.046537
ENPP2	0.028019
EPHA8	0.027379
F11	0.036362
F13B	0.047461
FAM179B	0.028019
FAM71C	0.014982
FBXO4	0.030328
FDXACB1	0.030328
FLNA	0.016351
FMR1	0.011778
FOXN4	0.003634
FOXO4	0.020281
FOXP1	0.020281
FOXR2	0.014982
FREM2	0.020258
FTSJ1	0.014982
GALNT15	0.045338
GALNT8	0.003754
GAS2L3	0.036654
GCC2	0.003644
GGN	0.030328
GLRA4	0.036362
GNAS	0.037491
GOLIM4	0.007386

GPLD1	0.037404
GPR26	0.036362
GPR52	0.030328
GPRIN3	0.011778
GRIK1	0.037491
GRM2	0.007386
HEXA	0.014609
HIF3A	0.020281
HSD17B4	0.014982
HSP90AB1	0.007386
IFT122	0.037404
IFT172	0.014647
IGF1R	0.036654
IGF2R	0.004717
IGSF21	0.037404
INSC	0.014647
IPO4	0.014982
KCNAB1	0.029701
KCNJ6	0.036654
KCNK13	0.011778
KCNQ2	0.019598
KCNT1	0.028019
KEAP1	3.36E-08
KIAA1462	0.003754
KIAA2018	0.02032
KIR2DL1	0.019794
KRAS	0.040755
KTI12	0.030328
KY	0.037404
LAMA4	0.005627
LEF1	0.031846
LGSN	0.030328
LHX8	0.019329
LPA	0.039242
LRP5	0.020827
LTN1	0.006557
LZTR1	0.036362
MAATS1	0.006557
MAPKBP1	0.036362
MCM4	0.020827
MECP2	0.036654
MED15	0.037404
MEGF6	0.020827
MEX3B	0.010578
MGA	0.024653
MIB2	0.010578
MTNR1A	0.032549
MYF6	0.020281
MYH15	0.023596
MYH6	0.044289
MYO9B	0.020827
MYOM3	0.023023
NACA	0.014609
NALCN	0.015183
NBPF9	0.030328
NCAPG2	0.035613
NCKAP5	0.044764
NCOR1	0.023596

NEGR1	0.031846
NELL1	0.035125
NLRP11	0.047461
NLRP14	0.021571
NLRP3	0.044764
NLRP5	0.012603
NNT	0.000208
NOS1	0.001928
NPHS1	0.019598
NPLOC4	0.014609
NTRK2	0.028019
NTSR1	0.036654
NUDCD1	0.000407
NUTM1	0.032549
NXPE4	0.031846
OR10A5	0.010883
OR13A1	0.020827
OR13C2	0.02032
OR2L13	0.001506
OR4A5	0.000355
OR4D5	0.036362
OR51A2	0.030328
OR51A4	0.010883
OR51E2	0.011778
OR5K4	0.037404
OR5R1	0.019329
OR5T1	0.028019
OR5W2	0.007674
OR7D4	0.002942
OR8J3	0.03826
OVCH1	0.019528
PABPC5	0.028019
PADI2	0.036654
PALLD	0.046537
PALM	0.030328
PARVG	0.010578
PC	0.037404
PCDH11Y	0.044454
PCDHB14	0.001726
PCDHB17	0.029701
PCDHB8	0.014793
PCDHGB2	0.023596
PCNXL3	0.036362
PCSK2	0.036362
PDS5A	0.019794
PGBD1	0.028019
PHIP	0.036362
PIGQ	0.030328
PIK3R6	0.029701
PLA2G4F	0.014982
PLEKHG5	0.036654
PNLIP	0.045338
PNPLA6	0.045338
PODXL2	0.014609
POM121L12	0.019528
POU6F2	0.045338
PPP1R15B	0.036654
PPP1R21	0.020281

PPP2R1A	0.02032
PRC1	0.030328
PRDM9	0.031943
PTPRB	0.039192
PTPRS	0.031846
RAD51AP2	0.030715
RASGRF2	0.001998
RAVER1	0.030328
RBM47	0.007171
REG3G	0.047461
RELN	0.031943
RNF216	0.007386
RNF219	0.020281
RPTN	0.045338
RSPH10B2	0.030328
RTTN	0.014647
RXRG	0.032549
SALL1	0.035125
SALL4	0.037491
SEMA3G	0.030328
SFRP4	0.014982
SGCZ	0.037491
SIGLEC14	0.035613
SLC22A25	0.036362
SLC26A3	0.016619
SLC2A10	0.032549
SLC38A7	0.030328
SLC39A12	0.002944
SLC4A2	0.020281
SLC5A12	0.028019
SLC5A4	0.020827
SLC6A18	0.011327
SLC8A3	0.025004
SLC9A5	0.037404
SLITRK5	0.015679
SMARCA4	0.014793
SMG5	0.036654
SORCS3	0.047648
SP100	0.011327
SPRED1	0.030328
SPTBN5	0.047461
SSH1	0.020281
SSMEM1	0.036654
ST18	0.016123
STK11	5.15E-05
SUN3	0.037404
SV2C	0.031846
SYMPK	0.036362
SYT16	0.045338
TAF1	0.047461
TAS2R16	0.020827
TBC1D16	0.010578
TBCK	0.014982
TDRD10	0.014982
THOC2	0.03826
TIE1	0.000874
TMC4	0.014982
TMEM260	0.037404

TMEM74	0.037404
TMEM81	0.030328
TMPRSS13	0.030328
TNFRSF9	0.030328
TNKS1BP1	0.011778
TOX2	0.020827
TPR	0.025004
TPTE	0.003947
TTLL11	0.014982
TTN	0.011973
UACA	0.020827
UBAP1	0.007386
UBR3	0.030328
UNC13D	0.037404
VN1R2	0.006585
WDHD1	0.030328
WDR16	0.02032
WDR64	0.011327
WWC2	0.030328
XPO6	0.030328
XXYLT1	0.030328
YES1	0.030328
ZBTB14	0.005788
ZFHX4	0.010468
ZNF134	0.007386
ZNF141	0.030328
ZNF215	0.020281
ZNF217	0.010883
ZNF292	0.047461
ZNF317	0.014609
ZNF33B	0.020281
ZNF423	0.018613
ZNF467	0.030328
ZNF676	0.008459
ZNF678	0.037404
ZNF679	0.010261
ZNF716	0.049383
ZNF816	0.037404

Table S6: 18430 genes with differential CNAs between high- and low-score groups

Table S6	
gene name	p
ACAP3	0.043756
ACTRT2	0.043756
AGRN	0.043756
ANKRD65	0.043756
ATAD3A	0.043756
ATAD3B	0.043756
ATAD3C	0.043756
AURKAIP1	0.043756
B3GALT6	0.043756
C1orf159	0.043756
C1orf170	0.043756
C1orf222	0.043756
C1orf233	0.043756
C1orf86	0.043756
CALML6	0.043756
CCNL2	0.043756
CDK11A	0.043756
CDK11B	0.043756
CPSF3L	0.043756
DDX11L1	0.043756
DVL1	0.043756
FAM132A	0.043756
FAM138A	0.043756
FAM213B	0.043756
FAM41C	0.043756
FAM87B	0.043756
GABRD	0.043756
GLTPD1	0.043756
GNB1	0.043756
HES4	0.043756
HES5	0.043756
ISG15	0.043756
KLHL17	0.043756
LINC00115	0.043756
LINC00982	0.043756
MIB2	0.043756
MIR200A	0.043756
MIR200B	0.043756
MIR429	0.043756
MMEL1	0.043756
MMP23B	0.043756
MORN1	0.043756
MRPL20	0.043756
MXRA8	0.043756
NADK	0.043756
NOC2L	0.043756
OR4F16	0.043756
OR4F29	0.043756
OR4F5	0.043756
PANK4	0.043756
PEX10	0.043756
PLCH2	0.043756
PLEKHN1	0.043756
PRDM16	0.043756
PRKCZ	0.043756
PUSL1	0.043756

RER1	0.043756
RN7SL657P	0.043756
RNF223	0.043756
SAMD11	0.043756
SCNN1D	0.043756
SDF4	0.043756
SKI	0.043756
SLC35E2B	0.043756
SLC35E2	0.043756
SSU72	0.043756
TAS1R3	0.043756
TMEM240	0.043756
TMEM52	0.043756
TMEM88B	0.043756
TNFRSF14	0.043756
TNFRSF18	0.043756
TNFRSF4	0.043756
TTC34	0.043756
TTLL10	0.043756
UBE2J2	0.043756
VWA1	0.043756
CCDC27	0.039863
LRRC47	0.049849
CEP104	0.049849
DFFB	0.049849
C1orf174	0.049849
AJAP1	0.047863
KCNAB2	0.036146
CHD5	0.034491
RNF207	0.039958
ICMT	0.039958
LINC00337	0.024625
GPR153	0.024625
HES3	0.024625
ACOT7	0.024625
HES2	0.028564
ESPN	0.029712
MIR4252	0.028564
PLEKHG5	0.029712
TNFRSF25	0.029712
NOL9	0.027709
TAS1R1	0.044573
ZBTB48	0.044573
KLHL21	0.044573
PHF13	0.044573
THAP3	0.044573
VAMP3	0.021151
PER3	0.021151
UTS2	0.02048
TNFRSF9	0.021973
PARK7	0.030774
ERRFI1	0.030774
RN7SL729P	0.033678
SLC45A1	0.035088
SNORA77|ENSG00000221083.1	0.046224
LZIC	0.03866
NMNAT1	0.03866
RN7SKP269	0.03866

MIR5697	0.03866
RBP7	0.03866
UBE4B	0.03866
KIF1B	0.03079
MIR1273D	0.03079
RN7SL731P	0.039802
APITD1	0.038847
CORT	0.038847
DFFA	0.038847
PEX14	0.03933
RN7SL614P	0.03933
CASZ1	0.03933
FBXO2	0.042459
FBXO44	0.042459
FBXO6	0.042459
MAD2L2	0.042459
DRAXIN	0.039719
AGTRAP	0.039719
C1orf167	0.03346
MTHFR	0.03346
CLCN6	0.03346
NPPA	0.03346
NPPB	0.03346
KIAA2013	0.03346
PLOD1	0.03346
MFN2	0.03346
MIIP	0.036376
RN7SL649P	0.036376
TNFRSF8	0.039719
VPS13D	0.036232
SNORA59A	0.037926
DHRS3	0.037926
RNU6ATAC18P	0.037926
AADACL4	0.028999
AADACL3	0.031632
snoU13|ENSG00000238771.1	0.031632
C1orf158	0.031632
HNRNPCL1	0.030295
PRAMEF10	0.030295
PRAMEF11	0.030295
PRAMEF12	0.030295
PRAMEF13	0.030295
PRAMEF14	0.030295
PRAMEF15	0.030295
PRAMEF16	0.030295
PRAMEF17	0.030295
PRAMEF18	0.030295
PRAMEF19	0.030295
PRAMEF1	0.030295
PRAMEF21	0.030295
PRAMEF22	0.030295
PRAMEF2	0.030295
PRAMEF3	0.030295
PRAMEF4	0.030295
PRAMEF5	0.030295
PRAMEF6	0.030295
PRAMEF7	0.030295
PRAMEF8	0.030295

PRAMEF9	0.030295
PRAMEF20	0.021918
LRRC38	0.021918
PDPN	0.022442
RNA5SP41	0.022442
SCARNA11|ENSG00000253085.1	0.021918
PRDM2	0.036025
KAZN	0.013528
TMEM51	0.024407
C1orf195	0.026161
FHAD1	0.020105
EFHD2	0.012011
CTRC	0.010738
CELA2A	0.010738
CELA2B	0.010738
CASP9	0.010738
DNAJC16	0.010738
SCARNA21|ENSG00000251866.1	0.010738
AGMAT	0.010738
DDI2	0.011338
RSC1A1	0.011338
PLEKHM2	0.015323
FBLIM1	0.015323
SLC25A34	0.015323
TMEM82	0.015323
SPEN	0.010868
snoU13|ENSG00000238818.1	0.012011
ZBTB17	0.012011
C1orf64	0.010738
HSPB7	0.010738
CLCNKA	0.010738
CLCNKB	0.010738
FAM131C	0.010738
EPHA2	0.008149
ARHGEF19	0.007689
ANO7P1	0.007689
C1orf134	0.007689
RSG1	0.007689
FBXO42	0.007689
SZRD1	0.008989
SPATA21	0.008989
NECAP2	0.008989
CROCCP3	0.012189
ATP13A2	0.016548
CROCCP2	0.011781
CROCC	0.011781
ESPNP	0.011781
MFAP2	0.011781
MIR3675	0.011781
MST1L	0.011781
NBPF1	0.011781
U1|ENSG00000228549.2	0.011781
U1|ENSG00000233421.3	0.011781
SDHB	0.004511
PADI2	0.004511
PADI1	0.006397
PADI3	0.006397
MIR3972	0.006397

PADI4	0.006397
PADI6	0.006664
RCC2	0.006664
snoU13|ENSG00000239020.1	0.006664
ARHGEF10L	0.004022
ACTL8	0.004779
IGSF21	0.008529
KLHDC7A	0.004777
PAX7	0.00592
TAS1R2	0.008185
ALDH4A1	0.008185
MIR4695	0.006645
MIR1290	0.006645
IFFO2	0.006645
UBR4	0.006645
EMC1	0.006645
MRTO4	0.006645
AKR7A3	0.006645
AKR7L	0.006645
AKR7A2	0.006645
PQLC2	0.006645
RN7SL85P	0.006645
CAPZB	0.010738
RN7SL277P	0.010738
snoU13|ENSG00000239027.1	0.006613
MINOS1	0.011257
NBL1	0.013956
HTR6	0.008056
TMCO4	0.008056
RNF186	0.009437
OTUD3	0.009945
PLA2G2E	0.009945
PLA2G2A	0.007607
RN7SL304P	0.007607
PLA2G5	0.007607
PLA2G2D	0.005617
PLA2G2F	0.002969
PLA2G2C	0.002969
UBXN10	0.002969
VWA5B1	0.002969
CAMK2N1	0.004264
MUL1	0.005784
FAM43B	0.005784
CDA	0.005784
PINK1	0.004052
DDOST	0.004052
KIF17	0.004052
SH2D5	0.004052
HP1BP3	0.004052
EIF4G3	0.005051
MIR1256	0.002763
ECE1	0.005051
NBPF3	0.003693
ALPL	0.0051
RAP1GAP	0.003917
USP48	0.003917
LDLRAD2	0.003917
HSPG2	0.008919

CDC42	0.008919
CELA3A	0.008919
CELA3B	0.008919
LINC00339	0.008919
RN7SL186P	0.008919
RN7SL386P	0.008919
RN7SL421P	0.008919
RN7SL768P	0.008919
WNT4	0.003917
MIR4418	0.004503
ZBTB40	0.009513
EPHA8	0.010566
C1QA	0.010566
C1QC	0.010566
C1QB	0.010566
EPHB2	0.010566
MIR4684	0.010566
MIR4253	0.010566
LACTBL1	0.008005
C1orf234	0.008005
KDM1A	0.008005
MIR3115	0.008005
MIR4419A	0.008005
LUZP1	0.011021
HTR1D	0.011021
HNRNPR	0.015182
ZNF436	0.015182
C1orf213	0.015182
TCEA3	0.015182
ASAP3	0.015182
E2F2	0.008271
ID3	0.006086
MDS2	0.004899
RPL11	0.003228
RN7SL532P	0.003228
TCEB3	0.002383
PITHD1	0.003142
GALE	0.003142
HMGCL	0.003142
LYPLA2	0.003142
FUCA1	0.003142
CNR2	0.002544
RN7SL24P	0.002544
snoU13|ENSG00000238538.1	0.002544
MIR378F	0.003142
PNRC2	0.003142
SRSF10	0.003142
MYOM3	0.003142
IL22RA1	0.003142
IFNLR1	0.004511
GRHL3	0.00436
STPG1	0.00811
NIPAL3	0.01035
RCAN3	0.01035
RN7SL857P	0.01035
NCMAP	0.01035
SRRM1	0.01035
snoU13|ENSG00000238986.1	0.01035

CLIC4	0.007478
RUNX3	0.003151
MIR4425	0.003209
C1orf63	0.004511
RHCE	0.002993
RHD	0.004511
SYF2	0.004511
TMEM50A	0.004511
snoU13|ENSG00000238889.1	0.004511
TMEM57	0.002256
LDLRAP1	0.002256
MAN1C1	0.003371
SEPN1	0.003371
MTFR1L	0.003371
AUNIP	0.003371
PAQR7	0.003371
STMN1	0.007676
MIR3917	0.007676
PAFAH2	0.003371
SCARNA17|ENSG00000252190.1	0.006725
SCARNA18|ENSG00000252691.1	0.006725
EXTL1	0.00495
SLC30A2	0.00495
TRIM63	0.00888
PDIK1L	0.00888
FAM110D	0.00888
ZNF593	0.00888
CNKSR1	0.00888
CATSPER4	0.00888
CEP85	0.008787
SH3BGRL3	0.008787
UBXN11	0.008787
AIM1L	0.012153
CD52	0.012153
RN7SL490P	0.012153
ZNF683	0.008787
LIN28A	0.008044
DHDDS	0.008044
HMGN2	0.008044
RPS6KA1	0.008044
MIR1976	0.008044
RN7SL679P	0.00888
snoU13|ENSG00000238316.1	0.004898
ARID1A	0.002536
RN7SL501P	0.003527
PIGV	0.004834
RN7SL165P	0.004721
ZDHHC18	0.004865
SFN	0.004865
GPN2	0.003298
GPATCH3	0.003298
NUDC	0.003298
NR0B2	0.003298
C1orf172	0.003298
TRNP1	0.004834
FAM46B	0.008816
SLC9A1	0.009114
WDTC1	0.015768

TMEM222	0.015768
SYTL1	0.015818
MAP3K6	0.015818
FCN3	0.015818
CD164L2	0.021215
GPR3	0.021215
WASF2	0.021382
AHDC1	0.015095
FGR	0.007886
IFI6	0.007886
FAM76A	0.005822
STX12	0.011013
PPP1R8	0.011013
SCARNA1	0.011013
THEMIS2	0.011013
RPA2	0.011013
SMPDL3B	0.011013
XKR8	0.011013
EYA3	0.011019
RN7SL559P	0.011019
PTAFR	0.015095
DNAJC8	0.015095
ATPIF1	0.015095
SESN2	0.015095
MED18	0.015095
PHACTR4	0.015095
RNU6ATAC27P	0.015095
RCC1	0.015095
SNHG3	0.015095
SNORA73B	0.015095
snoU13|ENSG00000238821.1	0.015095
TRNAU1AP	0.015095
SNHG12	0.015095
SNORD99	0.015095
TAF12	0.015095
RAB42	0.015095
RNU11	0.015095
GMEB1	0.015095
SCARNA24|ENSG00000252777.1	0.015095
YTHDF2	0.020226
OPRD1	0.012452
EPB41	0.01005
TMEM200B	0.01005
SRSF4	0.01005
MECR	0.013411
PTPRU	0.013411
LAPTM5	0.012821
MATN1	0.012821
MIR4420	0.012821
RN7SKP91	0.02209
SDC3	0.02209
PUM1	0.013814
SNORD103A	0.016696
SNORD103B	0.016696
SNORD85	0.016696
NKAIN1	0.009423
SNRNP40	0.015877
ZCCHC17	0.015877

FABP3	0.015877
SERINC2	0.015877
TINAGL1	0.018603
HCRTR1	0.018603
PEF1	0.018603
COL16A1	0.018603
BAI2	0.018603
MIR4254	0.014638
SPOCD1	0.014638
PTP4A2	0.014638
KHDRBS1	0.014638
TMEM39B	0.019638
MIR5585	0.019638
KPNA6	0.019638
TXLNA	0.02603
CCDC28B	0.02603
IQCC	0.02603
DCDC2B	0.02603
TMEM234	0.02603
EIF3I	0.02603
MTMR9LP	0.02603
FAM167B	0.02603
LCK	0.02603
HDAC1	0.02603
MARCKSL1	0.02603
TSSK3	0.02603
BSDC1	0.023335
FAM229A	0.02603
RN7SL122P	0.023335
ZBTB8B	0.023335
ZBTB8A	0.017487
ZBTB8OS	0.024575
RBBP4	0.036456
SYNC	0.036456
KIAA1522	0.047389
YARS	0.047389
ADC	0.046901
TRIM62	0.04575
ZNF362	0.03434
A3GALT2	0.03434
PHC2	0.034544
MIR3605	0.03434
RN7SKP16	0.03434
ZSCAN20	0.045436
CSMD2	0.030723
RNA5SP42	0.030817
C1orf94	0.030817
MIR552	0.030723
SMIM12	0.036947
GJB5	0.048224
GJB4	0.048224
GJB3	0.048224
GJA4	0.048224
DLGAP3	0.048224
SNORD112|ENSG00000252728.1	0.049536
ZMYM6NB	0.038061
ZMYM6	0.038061
ZMYM1	0.036755

SFPQ	0.036755
RN7SL136P	0.035291
RN7SL503P	0.035291
SNORA62|ENSG00000201542.1	0.035291
MACF1	0.038037
RNA5SP44	0.047717
BMP8A	0.047167
PABPC4	0.047167
SNORA55	0.047167
HEYL	0.036522
OXCT2	0.044854
CAP1	0.04251
PPT1	0.04251
ZFP69	0.034631
EXO5	0.034631
ZNF684	0.034631
RIMS3	0.035669
NFYC	0.03627
MIR30E	0.036409
MIR30C1	0.036409
KCNQ4	0.036409
RN7SL326P	0.036409
CITED4	0.036409
CTPS1	0.036409
SLFNL1	0.036409
SCMH1	0.03627
FOXO6	0.020485
RNA5SP45	0.020485
EDN2	0.020485
HIVEP3	0.020532
GUCA2B	0.018878
GUCA2A	0.018878
FOXJ3	0.018274
RIMKLA	0.024512
ZMYND12	0.024512
PPCS	0.024512
CCDC30	0.037588
PPIH	0.039027
YBX1	0.039027
SLC2A1	0.033195
FAM183A	0.033918
EBNA1BP2	0.033918
WDR65	0.033918
RNA5SP46	0.033918
TMEM125	0.033918
C1orf210	0.033918
TIE1	0.033918
MPL	0.033918
CDC20	0.033918
ELOVL1	0.033918
MED8	0.033918
SZT2	0.033918
HYI	0.033918
PTPRF	0.046461
SLC6A9	0.035403
KLF17	0.030087
RN7SL479P	0.023071
DMAP1	0.028498

ERI3	0.028498
RNF220	0.043234
MIR5584	0.042298
TMEM53	0.042298
C1orf228	0.042298
KIF2C	0.043207
BEST4	0.043207
BTBD19	0.043207
PLK3	0.043207
RPS8	0.043207
SNORD38A	0.043207
SNORD38B	0.043207
SNORD46|ENSG00000200913.1	0.043207
SNORD55	0.043207
TCTEX1D4	0.043207
PTCH2	0.043207
MKNK1	0.042282
MOB3C	0.042282
ATPAF1	0.037354
EFCAB14	0.04066
TEX38	0.046298
CYP4B1	0.033884
CYP4Z2P	0.04332
CYP4A11	0.034846
CYP4Z1	0.045145
LINC00853	0.047315
PDZK1IP1	0.047315
TAL1	0.047315
STIL	0.038486
CMPK1	0.038486
TRABD2B	0.018899
SKINTL	0.014978
SLC5A9	0.013574
SPATA6	0.023406
AGBL4	0.044945
BEND5	0.021554
snoU13|ENSG00000239144.1	0.018135
DAB1	0.049117
U3|ENSG00000199666.1	0.025003
BARHL2	0.029719
ZNF644	0.03242
HFM1	0.041297
TGFBR3	0.015529
RN7SL653P	0.029834
RN7SL235P	0.015529
BRDT	0.015529
BTBD8	0.027964
KIAA1107	0.048983
C1orf146	0.048983
ACTBP12	0.048983
GLMN	0.048983
RPAP2	0.048983
RN7SL824P	0.048983
GFI1	0.04132
EVI5	0.049757
BCAR3	0.048494
PRMT6	0.0142
NTNG1	0.014207

VAV3	0.014207
SLC25A24	0.036573
NBPF4	0.036573
SLC25A24P1	0.036573
NBPF5P	0.036573
NBPF6	0.036573
FAM102B	0.028677
HENMT1	0.028677
PRPF38B	0.028677
FNDC7	0.028677
STXBP3	0.02111
AKNAD1	0.020733
SPATA42	0.020733
GPSM2	0.020733
CLCC1	0.020733
WDR47	0.020733
TAF13	0.020733
C1orf194	0.020733
KIAA1324	0.015397
SCARNA2	0.020733
TMEM167B	0.020733
SARS	0.017367
CELSR2	0.017596
PSRC1	0.023182
MYBPHL	0.023182
SORT1	0.023182
PSMA5	0.023182
SYPL2	0.023182
ATXN7L2	0.023182
CYB561D1	0.023182
AMIGO1	0.023182
GPR61	0.023182
GNAI3	0.030402
RNU6V	0.023182
MIR197	0.030402
GNAT2	0.030402
AMPD2	0.030402
GSTM1	0.030402
GSTM2	0.030402
GSTM4	0.030402
GSTM5	0.030402
GSTM3	0.030402
EPS8L3	0.030402
CSF1	0.040137
AHCYL1	0.036736
STRIP1	0.040453
ALX3	0.03195
UBL4B	0.03195
SLC6A17	0.024151
KCNC4	0.032627
SNORA25|ENSG00000200536.1	0.040453
RBM15	0.029954
SLC16A4	0.040453
LAMTOR5	0.037413
PROK1	0.029954
CYMP	0.029954
KCNA10	0.037413
KCNA2	0.040453

KCNA3	0.040453
CD53	0.032627
LRIF1	0.030582
RNA5SP54	0.023796
DRAM2	0.029825
CEPT1	0.029825
DENND2D	0.029825
CHI3L2	0.029825
CHIAP2	0.038289
CHIA	0.029595
PIFO	0.029595
PGCP1	0.029595
OVGP1	0.029595
WDR77	0.02423
ATP5F1	0.02423
C1orf162	0.02423
ADORA3	0.02423
RAP1A	0.02423
FAM212B	0.02423
DDX20	0.02423
KCND3	0.032354
snoU13|ENSG00000239111.1	0.047671
snoU13|ENSG00000238761.1	0.03911
CTTNBP2NL	0.030011
WNT2B	0.030011
ST7L	0.030987
CAPZA1	0.030987
snoU13|ENSG00000238975.1	0.030987
MOV10	0.018622
RHOC	0.018622
PPM1J	0.018622
FAM19A3	0.018622
SLC16A1	0.01703
LRIG2	0.00827
MAGI3	0.009212
PHTF1	0.009212
RSBN1	0.007168
PTPN22	0.013038
BCL2L15	0.009212
AP4B1	0.009212
DCLRE1B	0.012188
HIPK1	0.012188
OLFML3	0.012188
SYT6	0.014788
TRIM33	0.019291
BCAS2	0.009037
DENND2C	0.005722
AMPD1	0.009037
RN7SL432P	0.009037
NRAS	0.009037
CSDE1	0.009037
SIKE1	0.005722
SYCP1	0.005722
TSHB	0.009581
TSPAN2	0.009581
NGF	0.004827
RN7SL420P	0.006737
SNORA42|ENSG00000207502.1	0.006737

VANGL1	0.007748
CASQ2	0.008044
NHLH2	0.007464
SLC22A15	0.012471
MAB21L3	0.012471
U3|ENSG00000221040.1	0.022851
ATP1A1	0.031202
ATP1A1OS	0.031202
CD58	0.0249
MIR548AC	0.0249
IGSF3	0.027374
MIR320B1	0.04347
C1orf137	0.04347
CD2	0.04347
CD101	0.033332
TTF2	0.033332
MIR942	0.033332
TRIM45	0.033332
VTCN1	0.033817
FAM46C	0.046944
SNORA40|ENSG00000212266.1	0.036545
GDAP2	0.016386
WDR3	0.016386
SPAG17	0.018529
RNA5SP56	0.025551
NOTCH2	0.026009
ACP6	0.032566
ANKRD20A12P	0.032566
ANKRD34A	0.032566
ANKRD35	0.032566
BCL9	0.032566
BOLA1	0.032566
CD160	0.032566
CHD1L	0.032566
FAM72B	0.032566
FAM72C	0.032566
FAM72D	0.032566
FCGR1A	0.032566
FCGR1B	0.032566
FCGR1C	0.032566
FMO5	0.032566
GJA5	0.032566
GJA8	0.032566
GNRHR2	0.032566
GPR89A	0.032566
GPR89B	0.032566
GPR89C	0.032566
HFE2	0.032566
HIST2H2AA3	0.032566
HIST2H2AA4	0.032566
HIST2H2AB	0.032566
HIST2H2AC	0.032566
HIST2H2BA	0.032566
HIST2H2BE	0.032566
HIST2H2BF	0.032566
HIST2H3A	0.032566
HIST2H3C	0.032566
HIST2H3D	0.032566

HIST2H4A	0.032566
HIST2H4B	0.032566
HYDIN2	0.032566
ITGA10	0.032566
LINC00623	0.032566
LINC00624	0.032566
LIX1L	0.032566
NBPF10	0.032566
NBPF11	0.032566
NBPF12	0.032566
NBPF14	0.032566
NBPF15	0.032566
NBPF16	0.032566
NBPF20	0.032566
NBPF24	0.032566
NBPF8	0.032566
NBPF9	0.032566
NOTCH2NL	0.032566
NUDT17	0.032566
PDE4DIP	0.032566
PDZK1P1	0.032566
PDZK1	0.032566
PEX11B	0.032566
PIAS3	0.032566
POLR3C	0.032566
POLR3GL	0.032566
PPIAL4A	0.032566
PPIAL4B	0.032566
PPIAL4C	0.032566
PPIAL4D	0.032566
PPIAL4G	0.032566
PRKAB2	0.032566
RBM8A	0.032566
RN7SKP88	0.032566
RN7SL261P	0.032566
RNA5SP57	0.032566
RNA5SP58	0.032566
RNA5SP59	0.032566
RNF115	0.032566
SEC22B	0.032566
SRGAP2B	0.032566
SV2A	0.011954
TXNIP	0.032566
SF3B4	0.006775
MTMR11	0.007552
OTUD7B	0.003793
VPS45	0.003774
PLEKHO1	0.003774
RN7SL480P	0.003663
ANP32E	0.003663
CA14	0.003663
snoU13|ENSG00000238526.1	0.003663
APH1A	0.003736
C1orf54	0.003736
C1orf51	0.003736
MRPS21	0.003736
PRPF3	0.003819
RPRD2	0.001

TARS2	0.001
ECM1	0.001
LINC00568	0.001
ADAMTSL4	0.000604
MIR4257	0.000604
C1orf138	0.000627
RN7SL473P	0.000627
RN7SL600P	0.000627
MCL1	0.000627
ENSA	0.000604
SNORA40|ENSG00000253047.1	0.000604
GOLPH3L	0.00087
HORMAD1	0.001251
CTSS	0.000759
CTSK	0.001974
ARNT	0.00155
SETDB1	0.00155
CERS2	0.001558
ANXA9	0.001581
FAM63A	0.001558
PRUNE	0.001558
BNIPL	0.001558
C1orf56	0.001558
CDC42SE1	0.001558
MLLT11	0.002693
GABPB2	0.002693
SEMA6C	0.004016
LYSMD1	0.004016
SCNM1	0.004016
TNFAIP8L2	0.004016
TMOD4	0.004016
VPS72	0.004016
PIP5K1A	0.003941
PSMD4	0.003941
ZNF687	0.006827
PI4KB	0.00721
RN7SL444P	0.00721
RFX5	0.007217
POGZ	0.007045
PSMB4	0.007045
SELENBP1	0.007045
RNY4P25	0.007045
CGN	0.003397
SNORA44	0.002264
TUFT1	0.002164
MIR554	0.003449
SNX27	0.003337
CELF3	0.003337
RIIAD1	0.003337
MRPL9	0.003163
OAZ3	0.003163
TDRKH	0.00204
LINGO4	0.00204
RORC	0.00204
C2CD4D	0.00204
THEM5	0.00204
THEM4	0.002264
S100A10	0.002264

NBPF18P	0.002264
S100A11	0.002264
TCHHL1	0.002264
TCHH	0.002264
RPTN	0.002264
HRNR	0.002264
FLG	0.002264
FLG2	0.002264
CRNN	0.002137
LCE5A	0.002137
CRCT1	0.002137
LCE3B	0.001969
LCE3C	0.001969
LCE3D	0.001969
LCE3E	0.001969
LCE3A	0.001969
LCE2D	0.001969
LINC00302	0.001969
LCE2B	0.002939
LCE2C	0.002939
LCE2A	0.002939
LCE4A	0.002939
C1orf68	0.002939
KPRP	0.004244
LCE1F	0.00449
LCE1C	0.00311
LCE1D	0.00311
LCE1E	0.00311
LCE1B	0.00311
LCE1A	0.003222
LCE6A	0.003222
SMCP	0.004739
IVL	0.006468
SPRR4	0.006468
SPRR1A	0.004417
SPRR3	0.005251
SNORA31|ENSG00000252920.1	0.005251
SPRR1B	0.006025
SPRR2D	0.006025
SPRR2A	0.004149
SPRR2B	0.004149
SPRR2E	0.004149
SPRR2F	0.004149
SPRR2G	0.003673
LELP1	0.003673
PRR9	0.003673
LOR	0.003673
PGLYRP3	0.002859
PGLYRP4	0.002859
S100A12	0.002859
S100A8	0.002859
S100A9	0.002859
S100A7A	0.001228
S100A7L2	0.000806
S100A7	0.000806
RN7SL44P	0.000806
S100A3	0.000527
S100A4	0.000527

S100A5	0.000527
S100A6	0.000527
S100A2	0.000557
S100A16	0.000557
S100A14	0.000557
S100A13	0.000645
S100A1	0.000645
CHTOP	0.000645
SNAPIN	0.000693
ILF2	0.000693
NPR1	0.000732
INTS3	0.001133
RN7SL372P	0.001133
snoU13|ENSG00000238511.1	0.001133
SLC27A3	0.001133
GATAD2B	0.001423
DENND4B	0.001423
CRTC2	0.001423
SLC39A1	0.001007
CREB3L4	0.001007
JTB	0.001007
RAB13	0.001007
NUP210L	0.001007
RPS27	0.001007
U3|ENSG00000252669.1	0.001007
MIR5698	0.001007
TPM3	0.00146
RN7SL431P	0.00146
MIR190B	0.00146
C1orf189	0.002025
C1orf43	0.001901
UBAP2L	0.00235
SNORA58|ENSG00000201129.1	0.00235
HAX1	0.00235
SNORD59|ENSG00000252682.1	0.00235
AQP10	0.002687
ATP8B2	0.002687
IL6R	0.002864
SHE	0.002864
TDRD10	0.002864
UBE2Q1	0.002864
CHRNB2	0.002864
ADAR	0.002864
KCNN3	0.002864
PMVK	0.003025
PBXIP1	0.002934
PYGO2	0.003025
SHC1	0.003025
CKS1B|ENSG00000173207.8	0.003025
MIR4258	0.003025
FLAD1	0.002735
LENEP	0.002735
ZBTB7B	0.002187
DCST2	0.001864
DCST1	0.001864
ADAM15	0.003904
EFNA3	0.003904
EFNA4	0.003904

EFNA1	0.004167
DPM3	0.003769
SLC50A1	0.003769
KRTCAP2	0.004112
TRIM46	0.004112
MIR92B	0.004112
MUC1	0.004112
THBS3	0.004112
MTX1	0.004112
GBAP1	0.004112
GBA	0.004112
FAM189B	0.004112
SCAMP3	0.004112
CLK2	0.003359
HCN3	0.003359
PKLR	0.003359
FDPS	0.003359
RUSC1	0.003359
ASH1L	0.002053
MIR555	0.003359
snoU13|ENSG00000238805.1	0.002754
MSTO1	0.005766
YY1AP1	0.013099
DAP3	0.013099
GON4L	0.008415
SYT11	0.004632
RIT1	0.00325
KIAA0907	0.002794
SNORA42|ENSG00000207475.1	0.002794
SCARNA4|ENSG00000252808.1	0.002794
RXFP4	0.002794
ARHGEF2	0.00396
SSR2	0.00396
UBQLN4	0.005488
LAMTOR2	0.005488
RAB25	0.005488
MEX3A	0.005525
LMNA	0.003625
SEMA4A	0.002302
SNORA26|ENSG00000252236.1	0.002302
SLC25A44	0.002007
PMF1	0.002007
BGLAP	0.003503
PAQR6	0.003503
SMG5	0.003503
C1orf85	0.003503
TMEM79	0.003503
VHLL	0.003503
CCT3	0.003503
TSACC	0.003503
RHBG	0.003503
C1orf61	0.004211
MEF2D	0.007948
IQGAP3	0.009018
snoU13|ENSG00000238843.1	0.009018
TTC24	0.009018
APOA1BP	0.007552
GPATCH4	0.007552

HAPLN2	0.00867
BCAN	0.011978
NES	0.01828
CRABP2	0.015623
ISG20L2	0.015623
RRNAD1	0.012922
MRPL24	0.012922
HDGF	0.012922
PRCC	0.008846
SH2D2A	0.008846
NTRK1	0.012168
INSRR	0.008846
PEAR1	0.011632
LRRC71	0.011632
ARHGEF11	0.011632
MIR765	0.011632
RN7SL612P	0.011632
ETV3L	0.013695
ETV3	0.013695
FCRL5	0.017873
FCRL4	0.017873
FCRL3	0.026905
FCRL2	0.035287
FCRL1	0.035287
CD5L	0.028552
KIRREL	0.026014
CD1D	0.033924
CD1A	0.014336
CD1C	0.022856
CD1B	0.022856
CD1E	0.022856
OR10T2	0.019471
OR10K2	0.019471
OR10K1	0.019471
OR10R2	0.019471
OR10X1	0.018996
OR6P1	0.018996
OR6Y1	0.018996
OR10Z1	0.012635
SPTA1	0.012635
OR6K2	0.012635
OR6K3	0.016385
OR6K6	0.016385
OR6N1	0.019471
OR6N2	0.019471
MNDA	0.019471
PYHIN1	0.019471
IFI16	0.0191
AIM2	0.010633
CADM3	0.015535
RNA5SP60	0.020201
DARC	0.015535
FCER1A	0.017812
OR10J3	0.017812
OR10J4	0.02744
OR10J1	0.02744
OR10J5	0.0195
APCS	0.0195

CRP	0.020072
DUSP23	0.020072
FCRL6	0.025714
SLAMF8	0.027869
C1orf204	0.027869
SNORD64|ENSG00000212161.1	0.027869
VSIG8	0.027869
CCDC19	0.027869
TAGLN2	0.027869
IGSF9	0.027869
SLAMF9	0.027869
PIGM	0.027869
KCNJ10	0.027869
KCNJ9	0.027869
IGSF8	0.027869
ATP1A2	0.027869
DCAF8	0.043624
PEX19	0.043624
COPA	0.047135
F11R	0.049491
TSTD1	0.049491
USF1	0.049491
ARHGAP30	0.049491
PVRL4	0.049491
KLHDC9	0.049491
PFDN2	0.049491
NIT1	0.045541
DEDD	0.045541
ACA64|ENSG00000238934.1	0.045541
UFC1	0.045541
USP21	0.045541
B4GALT3	0.045541
PPOX	0.045541
ADAMTS4	0.045541
NDUFS2	0.045541
FCER1G	0.046057
APOA2	0.046057
MIR5187	0.046057
NR1I3	0.046057
TOMM40L	0.046057
PCP4L1	0.046057
MPZ	0.046057
SDHC	0.045541
C1orf192	0.045541
FCGR2A	0.041419
FCGR2B	0.041419
FCGR2C	0.041419
FCGR3A	0.041419
FCGR3B	0.041419
HSPA6	0.041419
RPL31P11	0.041419
FCRLA	0.041419
FCRLB	0.041419
RN7SL466P	0.041419
DUSP12	0.041419
ATF6	0.041419
RGS5	0.046621
NUF2	0.038649

SNORD112|ENSG00000252740.1	0.035569
RNA5SP62	0.038649
RNA5SP63	0.033826
U3|ENSG00000212538.1	0.039964
PBX1	0.017329
SNORD112|ENSG00000252359.1	0.024594
LMX1A	0.020233
RXRG	0.016739
LRRC52	0.016739
MGST3	0.016739
ALDH9A1	0.016739
TMCO1	0.016739
UCK2	0.020417
RNA5SP64	0.033373
FAM78B	0.026404
MIR921	0.026404
FMO9P	0.038668
POGK	0.044857
TADA1	0.037518
ILDR2	0.046881
MAEL	0.01945
RNA5SP65	0.01945
snoU13|ENSG00000238325.1	0.027162
GPA33	0.027162
DUSP27	0.027162
POU2F1	0.04654
MPZL1	0.049501
MIR1255B2	0.049501
XCL1	0.044703
DPT	0.04009
LINC00626	0.04009
LINC00970	0.04009
RNA5SP66	0.04009
ATP1B1	0.04009
NME7	0.031295
BLZF1	0.048196
CCDC181	0.048196
SLC19A2	0.048196
SELP	0.045382
C1orf112	0.035201
SELL	0.045382
SELE	0.035201
METTL18	0.041029
SCYL3	0.041029
RN7SL333P	0.041029
KIFAP3	0.041029
RN7SL269P	0.041029
METTL11B	0.041029
MROH9	0.043933
FMO3	0.043933
MIR1295A	0.043933
FMO6P	0.043933
FMO2	0.043933
DNM3	0.034613
DNM3OS	0.03381
MIR214	0.03381
MIR199A2	0.03381
SNORD112|ENSG00000252354.1	0.03381

PIGC	0.040201
C1orf105	0.04241
SUCO	0.04241
FASLG	0.04241
TNFSF18	0.04241
TNFSF4	0.04241
snoU13|ENSG00000251817.1	0.04241
PRDX6	0.04241
SLC9C2	0.04241
ANKRD45	0.04241
snoU13|ENSG00000238430.1	0.04241
KLHL20	0.04241
RN7SKP160	0.04241
CENPL	0.04241
DARS2	0.04241
GAS5	0.03875
SNORD78|ENSG00000208317.1	0.03875
ZBTB37	0.03875
SERPINC1	0.03875
RNA5SP67	0.03875
RC3H1	0.03875
RNA5SP68	0.03875
RABGAP1L	0.027736
snoU13|ENSG00000238872.1	0.032418
GPR52	0.03381
CACYBP	0.024071
MRPS14	0.024071
TNN	0.018819
KIAA0040	0.018819
TNR	0.018963
RFWD2	0.045272
SCARNA3	0.034301
PAPPA2	0.040723
ASTN1	0.040533
MIR488	0.040533
FAM5B	0.040533
SEC16B	0.025875
RASAL2	0.024237
LINC00083	0.030229
TEX35	0.030229
C1ORF220	0.030229
C1orf220	0.030229
RNA5SP69	0.030229
MIR4424	0.030229
RALGPS2	0.025796
SNORA63|ENSG00000201791.1	0.03457
ANGPTL1	0.031028
FAM20B	0.031028
TOR3A	0.031028
ABL2	0.031028
SNORA67|ENSG00000201619.1	0.031028
SNORA67|ENSG00000212338.1	0.031028
SOAT1	0.031028
AXDND1	0.031028
RN7SL374P	0.031028
NPHS2	0.031028
TDRD5	0.031028
FAM163A	0.031028

TOR1AIP2	0.038631
IFRG15	0.038631
TOR1AIP1	0.031246
RN7SL230P	0.031246
CEP350	0.031246
QSOX1	0.026001
LHX4	0.019712
ACBD6	0.012394
MIR3121	0.023958
XPR1	0.019712
U6|ENSG00000272292.1	0.039046
KIAA1614	0.015978
STX6	0.012132
MR1	0.012132
IER5	0.012132
CACNA1E	0.012355
RNA5SP70	0.015978
RN7SKP229	0.015978
ZNF648	0.015978
GLUL	0.015414
TEDDM1	0.015414
LINC00272	0.015414
RGSL1	0.009642
RNASEL	0.009642
RGS16	0.009642
RGS8	0.009642
NPL	0.009642
DHX9	0.009642
SHCBP1L	0.0165
RNA5SP71	0.0165
LAMC1	0.0165
LAMC2	0.0165
NMNAT2	0.012395
SMG7	0.0165
NCF2	0.0165
ARPC5	0.0165
RGL1	0.019652
APOBEC4	0.0165
COLGALT2	0.019652
TSEN15	0.019652
RN7SL654P	0.0084
C1orf21	0.004444
SNORD112|ENSG00000252790.1	0.004593
EDEM3	0.004593
FAM129A	0.003322
RNA5SP72	0.003322
RNF2	0.004593
TRMT1L	0.004593
SWT1	0.004593
IVNS1ABP	0.004593
HMCN1	0.005549
PRG4	0.007666
TPR	0.007666
C1orf27	0.005549
OCLM	0.007666
PDC	0.005549
PTGS2	0.005549
PLA2G4A	0.007289

RN7SKP156	0.007128
RNA5SP73	0.01784
FAM5C	0.014354
RGS18	0.021998
RGS21	0.021998
RGS1	0.028041
RGS13	0.026667
RGS2	0.021998
RN7SKP126	0.021998
UCHL5	0.021998
snoU109|ENSG00000238754.1	0.021998
TROVE2	0.021998
GLRX2	0.021998
CDC73	0.021998
MIR1278	0.02321
B3GALT2	0.021998
U3|ENSG00000252241.1	0.027952
KCNT2	0.027384
MIR4735	0.027384
CFH	0.027384
CFHR1	0.027384
CFHR2	0.027384
CFHR3	0.027384
CFHR4	0.027384
CFHR5	0.027384
F13B	0.036354
ASPM	0.036354
ZBTB41	0.041539
CRB1	0.041539
DENND1B	0.018297
C1orf53	0.015599
LHX9	0.011725
NEK7	0.014034
ATP6V1G3	0.011519
PTPRC	0.011519
MIR181A1HG	0.012226
MIR181A1	0.01523
MIR181B1	0.01523
NR5A2	0.008883
LINC00862	0.008092
ZNF281	0.008092
KIF14	0.007488
DDX59	0.012802
CAMSAP2	0.012802
GPR25	0.008604
C1orf106	0.011074
KIF21B	0.011074
CACNA1S	0.011074
ASCL5	0.011074
TMEM9	0.020447
IGFN1	0.020447
PKP1	0.015278
TNNT2	0.015278
LAD1	0.015278
TNNI1	0.015278
PHLDA3	0.015278
CSRP1	0.015278
RPS10P7	0.015278

NAV1	0.00825
MIR5191	0.00722
MIR1231	0.005128
IPO9	0.002897
SHISA4	0.003265
LMOD1	0.001993
TIMM17A	0.00142
SNORA70|ENSG00000206637.1	0.00142
RNPEP	0.001928
ELF3	0.001928
GPR37L1	0.003335
ARL8A	0.003335
PTPN7	0.003335
PTPRVP	0.003335
LGR6	0.004493
snoU13|ENSG00000238571.1	0.004493
snoU13|ENSG00000239046.1	0.004493
UBE2T	0.002098
PPP1R12B	0.002098
U6|ENSG00000272262.1	0.002098
SNORA70|ENSG00000253042.1	0.002098
SYT2	0.002098
KDM5B	0.003794
PCAT6	0.003794
RABIF	0.003794
KLHL12	0.003794
ADIPOR1	0.003794
CYB5R1	0.003794
TMEM183A	0.003794
PPFIA4	0.003794
MYOG	0.003794
ADORA1	0.003794
MYBPH	0.003794
CHI3L1	0.003794
CHIT1	0.005116
BTG2	0.010159
FMOD	0.010159
PRELP	0.012643
OPTC	0.012643
ATP2B4	0.007383
SNORA77|ENSG00000221643.1	0.007383
LAX1	0.007383
ZBED6	0.007383
ZC3H11A	0.007383
SNRPE	0.009843
LINC00303	0.009843
SOX13	0.007383
ETNK2	0.007383
REN	0.007383
KISS1	0.007383
GOLT1A	0.007383
PLEKHA6	0.005073
PPP1R15B	0.005477
PIK3C2B	0.010674
MDM4	0.010674
RNA5SP74	0.010674
LRRN2	0.005073
RNA5SP75	0.006973

SCARNA20|ENSG00000251861.1	0.006973
NFASC	0.00651
SNORD112|ENSG00000252946.1	0.00651
CNTN2	0.006864
TMEM81	0.006784
RBBP5	0.006784
DSTYK	0.008872
TMCC2	0.008129
NUAK2	0.008129
KLHDC8A	0.008129
LEMD1	0.006329
MIR135B	0.006329
CDK18	0.006329
MFSD4	0.006329
ELK4	0.006329
SLC45A3	0.00548
NUCKS1	0.007475
SNORA72|ENSG00000201944.1	0.007475
RAB7L1	0.007475
SLC41A1	0.006942
PM20D1	0.007475
SLC26A9	0.007475
FAM72A	0.006469
AVPR1B	0.00519
C1orf186	0.00519
SNORD60|ENSG00000252692.1	0.00519
CTSE	0.004503
SRGAP2	0.005763
IKBKE	0.005763
C1orf147	0.005763
RASSF5	0.005763
EIF2D	0.005763
DYRK3	0.005763
SNORD112|ENSG00000252853.1	0.007627
MAPKAPK2	0.007627
IL10	0.007627
IL19	0.007627
IL20	0.007627
IL24	0.007627
FAIM3	0.007627
PIGR	0.007627
FCAMR	0.007627
C1orf116	0.007627
snoU13|ENSG00000238401.1	0.007627
YOD1	0.007627
PFKFB2	0.007627
C4BPB	0.007627
C4BPA	0.013022
CD55	0.013022
CR2	0.013022
CR1	0.010079
CR1L	0.010445
CD46	0.008046
MIR29B2	0.008046
MIR29C	0.008046
C1orf132	0.008046
CD34	0.004527
PLXNA2	0.006108

MIR205HG	0.002214
CAMK1G	0.003
LAMB3	0.003
MIR4260	0.003
G0S2	0.003
HSD11B1	0.003
TRAF3IP3	0.003
C1orf74	0.003
IRF6	0.003
DIEXF	0.003
SYT14	0.005656
SERTAD4	0.002111
HHAT	0.002191
KCNH1	0.001411
RCOR3	0.002373
TRAF5	0.001482
LINC00467	0.001482
RD3	0.001482
SLC30A1	0.001113
NEK2	0.000648
LPGAT1	0.001136
RN7SL344P	0.001136
INTS7	0.00161
SNORA26|ENSG00000212187.1	0.00161
DTL	0.00161
MIR3122	0.00161
RN7SKP98	0.00161
PPP2R5A	0.00161
SNORA16B	0.002213
TMEM206	0.002213
NENF	0.001267
ATF3	0.001267
RN7SL512P	0.001267
FAM71A	0.001267
BATF3	0.001267
NSL1	0.001267
TATDN3	0.001267
C1orf227	0.001024
FLVCR1	0.001024
VASH2	0.001024
ANGEL2	0.001409
RPS6KC1	0.002241
PROX1	0.001329
SMYD2	0.001146
PTPN14	0.00341
CENPF	0.00292
KCNK2	0.000876
KCTD3	0.003591
USH2A	0.003591
SNORD116|ENSG00000202498.1	0.003591
ESRRG	0.004673
GPATCH2	0.007918
SPATA17	0.011346
LINC00210	0.011346
RRP15	0.013645
TGFB2	0.013645
C1orf143	0.016383
U3|ENSG00000212610.1	0.023168

LYPLAL1	0.016383
SLC30A10	0.017314
RNA5SP76	0.017314
U3|ENSG00000221673.1	0.017314
EPRS	0.017314
snoU13|ENSG00000238798.1	0.017314
BPNT1	0.017314
IARS2	0.017314
MIR215	0.017314
snoU13|ENSG00000238576.1	0.017314
RAB3GAP2	0.017713
SNORA36B	0.017314
MARK1	0.020087
RN7SL464P	0.020087
HDAC1P2	0.020087
C1orf115	0.025278
2-Mar	0.025278
1-Mar	0.025278
RNU6ATAC35P	0.023168
HLX	0.023168
DUSP10	0.021019
HHIPL2	0.01761
TAF1A	0.024895
MIA3	0.021974
AIDA	0.035108
BROX	0.045596
RN7SL276P	0.045596
FAM177B	0.030386
DISP1	0.032149
snoU13|ENSG00000239054.1	0.030386
TLR5	0.033014
SUSD4	0.049561
C1orf65	0.049561
CAPN8	0.045522
CAPN2	0.048475
TP53BP2	0.048475
RN7SKP49	0.049702
FBXO28	0.049702
DEGS1	0.049702
SNORA72|ENSG00000201898.1	0.049702
NVL	0.020423
MIR320B2	0.020423
snoU13|ENSG00000238856.1	0.020423
CNIH4	0.023766
WDR26	0.023766
MIR4742	0.023766
CNIH3	0.020173
DNAH14	0.023766
LBR	0.026518
ENAH	0.025328
SRP9	0.02241
EPHX1	0.015074
TMEM63A	0.024468
LEFTY1	0.024468
PYCR2	0.024468
LEFTY2	0.019742
SDE2	0.019742
ACBD3	0.025864

MIXL1	0.0336
LIN9	0.0336
snoU13|ENSG00000238545.1	0.0336
snoU13|ENSG00000239094.1	0.0336
PARP1	0.0336
RN7SKP165	0.042597
C1orf95	0.040983
RNA5SP77	0.037972
ZNF678	0.037972
JMJD4	0.031472
SNAP47	0.030634
PRSS38	0.030634
WNT9A	0.037972
MIR5008	0.037972
WNT3A	0.031472
ARF1	0.031472
MIR3620	0.02609
C1orf35	0.02609
MRPL55	0.02609
GJC2	0.02609
GUK1	0.02609
C1orf148	0.02609
IBA57	0.02609
C1orf145	0.02609
OBSCN	0.021222
BTNL10	0.019504
HIST3H2A	0.019504
HIST3H2BB	0.019504
HIST3H3	0.019504
MIR4666A	0.019504
RNA5S10	0.019504
RNA5S11	0.019504
RNA5S12	0.019504
RNA5S13	0.019504
RNA5S14	0.019504
RNA5S15	0.019504
RNA5S16	0.019504
RNA5S17	0.019504
RNA5S1	0.019504
RNA5S2	0.019504
RNA5S3	0.019504
RNA5S4	0.019504
RNA5S5	0.019504
RNA5S6	0.019504
RNA5S7	0.019504
RNA5S8	0.019504
RNA5S9	0.019504
RNA5SP18	0.019504
RNA5SP19	0.019504
RNF187	0.019504
SNORA51|ENSG00000206878.1	0.019504
TRIM11	0.019504
TRIM17	0.019504
RHOU	0.030691
TMEM78	0.040938
RAB4A	0.040938
SPHAR	0.040938
CCSAP	0.040938

RN7SKP276	0.040938
ACTA1	0.040938
NUP133	0.032667
ABCB10	0.044532
RNA5SP78	0.038075
TAF5L	0.044532
URB2	0.044532
GALNT2	0.043461
PGBD5	0.020661
COG2	0.015975
AGT	0.015975
RN7SL467P	0.015975
CAPN9	0.01416
RNA5SP79	0.01416
C1orf198	0.01416
RN7SL837P	0.01989
snoU13|ENSG00000238985.1	0.01989
TTC13	0.01989
ARV1	0.01989
FAM89A	0.01989
MIR1182	0.01989
TRIM67	0.01989
C1orf131	0.016615
GNPAT	0.016615
RNA5SP80	0.016615
EXOC8	0.016615
SPRTN	0.016615
EGLN1	0.012948
TRAX	0.016615
TSNAX	0.016615
DISC1	0.012314
LINC00582	0.016615
RN7SL299P	0.003399
SIPA1L2	0.004986
MAP10	0.003112
NTPCR	0.005577
PCNXL2	0.005577
MLK4	0.003837
KCNK1	0.002976
MIR4427	0.002976
SLC35F3	0.014665
MIR4671	0.01265
COA6	0.01265
TARBP1	0.005017
U8|ENSG00000212144.1	0.004696
IRF2BP2	0.007057
LINC00184	0.007057
RNY4P16	0.007161
RN7SL668P	0.005799
TOMM20	0.00321
ARID4B	0.004177
RBM34	0.004177
SNORA14B	0.004177
MIR4753	0.006859
GGPS1	0.006859
TBCE	0.005454
B3GALNT2	0.005454
GNG4	0.005618

LYST	0.004411
MIR1537	0.004411
NID1	0.004337
GPR137B	0.004177
ERO1LB	0.004177
snoU13|ENSG00000252638.1	0.004177
EDARADD	0.005643
LGALS8	0.007034
HEATR1	0.007034
ACTN2	0.003998
MTR	0.007034
MT1HL1	0.006915
RYR2	0.002097
RN7SKP195	0.003575
MIR4428	0.004492
SNORA25|ENSG00000252290.1	0.005828
ZP4	0.005828
MTRNR2L11	0.004492
CHRM3	0.003826
FMN2	0.002324
GREM2	0.001802
RGS7	0.002659
MIR3123	0.002225
FH	0.004951
KMO	0.007521
OPN3	0.010841
CHML	0.010841
WDR64	0.013132
EXO1	0.013132
MAP1LC3C	0.009738
PLD5	0.007365
RNA5SP81	0.009738
RN7SKP12	0.009738
CEP170	0.01223
SDCCAG8	0.023239
MIR4677	0.015448
AKT3	0.012364
ZBTB18	0.015448
RN7SL148P	0.015448
C1orf100	0.020768
ADSS	0.017462
C1orf101	0.017462
DESI2	0.013845
COX20	0.018203
HNRNPU	0.018203
RN7SKP55	0.013156
EFCAB2	0.010523
KIF26B	0.009865
SMYD3	0.009549
AHCTF1	0.00615
CNST	0.0112
SCCPDH	0.0112
SNORA25|ENSG00000252011.1	0.0112
SNORD112|ENSG00000252495.1	0.0112
TFB2M	0.0112
ZNF670	0.004243
ZNF695	0.002863
ZNF669	0.003746

C1orf229	0.003746
ZNF124	0.003746
MIR3916	0.003746
RNA5SP82	0.003746
ZNF496	0.003746
NLRP3	0.004842
OR2B11	0.006101
OR2W5	0.006101
GCSAML	0.006412
OR2C3	0.006101
OR2G2	0.006101
OR2G3	0.006101
LYPD8	0.006766
MIR3124	0.006766
OR11L1	0.006766
OR13G1	0.006766
OR14A16	0.006766
OR14A2	0.006766
OR14C36	0.006766
OR14I1	0.006766
OR14K1	0.006766
OR1C1	0.006766
OR2AJ1	0.006766
OR2AK2	0.006766
OR2G6	0.006766
OR2L13	0.006766
OR2L2	0.006766
OR2L3	0.006766
OR2L5	0.006766
OR2L8	0.006766
OR2M2	0.006766
OR2M3	0.006766
OR2M4	0.006766
OR2M5	0.006766
OR2M7	0.006766
OR2T10	0.006766
OR2T11	0.006766
OR2T12	0.006766
OR2T1	0.006766
OR2T27	0.006766
OR2T29	0.006766
OR2T2	0.006766
OR2T33	0.006766
OR2T34	0.006766
OR2T35	0.006766
OR2T3	0.006766
OR2T4	0.006766
OR2T5	0.006766
OR2T6	0.006766
OR2T7	0.006766
OR2T8	0.006766
OR2W3	0.006766
OR6F1	0.006766
PGBD2	0.006766
SH3BP5L	0.006766
TRIM58	0.006766
ZNF672	0.006766
ZNF692	0.006766

ACP1	0.001653
FAM110C	0.001653
FAM150B	0.001653
SH3YL1	0.001653
TMEM18	0.002469
MYT1L	0.00057
PXDN	0.001253
SNTG2	0.001253
TPO	0.001253
snoU13|ENSG00000238722.1	0.000587
TSSC1	0.001245
TRAPPC12	0.001264
ADI1	0.00092
RNASEH1	0.00092
RPS7	0.00092
SNORA73|ENSG00000252531.1	0.00092
COLEC11	0.000616
ALLC	0.000616
DCDC2C	0.00092
RN7SL531P	0.001715
SNORA31|ENSG00000252238.1	0.003406
SOX11	0.003045
FLJ30594	0.002618
LINC00487	0.003045
CMPK2	0.003045
RSAD2	0.003045
RNF144A	0.003139
RN7SKP112	0.002189
RNU6ATAC37P	0.002238
LINC00299	0.001486
ID2	0.003045
KIDINS220	0.003045
MBOAT2	0.003139
snoU13|ENSG00000238888.1	0.003045
ASAP2	0.002434
ITGB1BP1	0.002415
CPSF3	0.002415
IAH1	0.002415
ADAM17	0.002415
snoU13|ENSG00000238462.1	0.002415
YWHAQ	0.001657
TAF1B	0.002238
GRHL1	0.003045
KLF11	0.003045
CYS1	0.003045
SNORA26|ENSG00000212558.1	0.003045
RRM2	0.003045
C2orf48	0.003139
SNORA2|ENSG00000206647.1	0.003139
MIR4261	0.003139
RN7SL66P	0.003139
SNORA51|ENSG00000206898.1	0.003139
HPCAL1	0.003045
ODC1	0.003045
SNORA80B	0.003045
NOL10	0.002174
RN7SL832P	0.002174
ATP6V1C2	0.002174

PDIA6	0.001204
KCNF1	0.000704
C2orf50	0.000404
PQLC3	0.000404
ROCK2	0.000618
LINC00570	0.000852
E2F6	0.000852
RNA5SP84	0.000852
GREB1	0.000852
MIR4429	0.000852
RNA5SP85	0.000852
RN7SL674P	0.000852
NTSR2	0.000852
LPIN1	0.000852
MIR548S	0.000598
MIR4262	0.000758
SNORD18|ENSG00000238503.1	0.000598
MIR3681	0.000598
TRIB2	0.000617
MIR3125	0.000424
LINC00276	0.00114
FAM84A	0.00083
NBAS	0.000462
DDX1	0.000448
snoU13|ENSG00000238371.1	0.000225
MYCNOS	0.000162
MYCN	0.000162
RN7SL104P	0.000162
SNORA40|ENSG00000251704.1	0.000266
FAM49A	0.000504
RN7SKP168	0.000675
RAD51AP2	0.000612
VSNL1	0.000351
SMC6	0.000485
GEN1	0.000485
MSGN1	0.000485
KCNS3	0.000685
SNORA40|ENSG00000212455.1	0.000967
NT5C1B	0.000842
RDH14	0.000842
MIR4757	0.000916
OSR1	0.000683
LINC00954	0.000933
TTC32	0.000933
WDR35	0.000933
MATN3	0.000933
LAPTM4A	0.000933
RN7SL140P	0.001786
SDC1	0.001786
PUM2	0.001786
RNA5SP86	0.001786
RHOB	0.001786
HS1BP3	0.001288
GDF7	0.001288
C2orf43	0.001288
APOB	0.000488
TDRD15	0.001137
RN7SL117P	0.000738

RNA5SP87	0.000992
RN7SKP27	0.000535
KLHL29	0.000922
ATAD2B	0.000548
UBXN2A	0.000501
RN7SL610P	0.000501
MFSD2B	0.000501
C2orf44	0.000501
FKBP1B	0.000501
SF3B14	0.000501
FAM228B	0.000501
TP53I3	0.000501
PFN4	0.000501
FAM228A	0.000501
ITSN2	0.000245
SCARNA21|ENSG00000251805.1	0.000349
NCOA1	0.000349
RNA5SP88	0.000501
PTRHD1	0.000948
CENPO	0.000948
ADCY3	0.000948
DNAJC27	0.000948
SNORD14	0.000948
EFR3B	0.000948
RN7SL856P	0.000948
POMC	0.000948
DNMT3A	0.000501
MIR1301	0.000501
DTNB	0.000461
ASXL2	0.000332
KIF3C	0.000169
RAB10	0.000169
GAREML	0.000332
HADHA	0.000349
HADHB	0.000349
EPT1	0.000349
GPR113	0.000349
DRC1	0.000349
OTOF	0.000501
C2orf70	0.000332
CIB4	0.000332
KCNK3	0.000332
CENPA	0.000332
SLC35F6	0.000332
DPYSL5	0.000332
MAPRE3	0.000501
TMEM214	0.000501
AGBL5	0.000501
OST4	0.000501
EMILIN1	0.000501
KHK	0.000501
CGREF1	0.000501
ABHD1	0.000501
PREB	0.000501
C2orf53	0.000501
TCF23	0.000501
SLC5A6	0.000501
ATRAID	0.000501

CAD	0.000501
SLC30A3	0.000501
DNAJC5G	0.000948
TRIM54	0.000948
MPV17	0.000948
UCN	0.000948
GTF3C2	0.000948
EIF2B4	0.000948
SNX17	0.000948
ZNF513	0.000948
PPM1G	0.000948
NRBP1	0.000948
IFT172	0.000948
KRTCAP3	0.000948
FNDC4	0.000948
GCKR	0.000948
C2orf16	0.000948
ZNF512	0.000948
CCDC121	0.000948
GPN1	0.000948
SNORA36|ENSG00000206731.1	0.000948
SUPT7L	0.000501
SLC4A1AP	0.000501
MRPL33	0.000501
RBKS	0.000501
BRE	0.000361
MIR4263	0.000501
FOSL2	0.001336
PLB1	0.000948
RNA5SP89	0.000948
PPP1CB	0.000948
FAM179A	0.000674
SNORD53	0.000948
SNORD92	0.000948
SPDYA	0.000948
TRMT61B	0.000948
WDR43	0.000948
C2orf71	0.000674
CLIP4	0.000876
ALK	0.000876
RN7SL516P	0.000987
YPEL5	0.00086
SNORA64|ENSG00000207187.1	0.00086
LBH	0.00086
LCLAT1	0.00086
CAPN13	0.00086
GALNT14	0.001431
CAPN14	0.00086
RNA5SP90	0.00086
EHD3	0.00086
XDH	0.00086
SRD5A2	0.00086
DPY30	0.00086
MEMO1	0.00086
SPAST	0.00086
SLC30A6	0.00086
NLRC4	0.00086
YIPF4	0.001912

BIRC6	0.002146
LINC00486	0.002146
LTBP1	0.001664
MIR4765	0.002146
MIR558	0.002146
SNORD112|ENSG00000252502.1	0.002146
TTC27	0.002146
RNA5SP91	0.000565
RNA5SP92	0.000565
RASGRP3	0.000565
FAM98A	0.000738
MYADML	0.000738
RN7SL602P	0.001917
MIR548AD	0.002587
CRIM1	0.001488
FEZ2	0.001488
VIT	0.001488
STRN	0.001154
HEATR5B	0.000868
GPATCH11	0.000868
EIF2AK2	0.000868
SULT6B1	0.000719
CEBPZ	0.000719
NDUFAF7	0.000719
PRKD3	0.001474
QPCT	0.001474
CDC42EP3	0.002255
LINC00211	0.002255
RMDN2	0.002255
CYP1B1	0.002854
ATL2	0.004354
HNRNPLL	0.003262
GALM	0.003213
GEMIN6	0.003213
SRSF7	0.003213
DHX57	0.003213
MORN2	0.003213
ARHGEF33	0.003213
RN7SL96P	0.003213
SOS1	0.003213
CDKL4	0.003213
MAP4K3	0.00259
SNORA67|ENSG00000252473.1	0.003213
TMEM178A	0.003958
THUMPD2	0.003958
SLC8A1	0.004102
HNRNPA1P57	0.003262
snoZ247	0.002449
C2orf91	0.004128
PKDCC	0.006787
EML4	0.004569
COX7A2L	0.005096
KCNG3	0.003756
SNORD75|ENSG00000221300.1	0.004516
MTA3	0.007645
OXER1	0.006463
HAAO	0.006463
THADA	0.002304

ZFP36L2	0.004596
PLEKHH2	0.002157
RN7SKP66	0.000915
DYNC2LI1	0.000915
ABCG5	0.000668
ABCG8	0.000668
LRPPRC	0.000668
PPM1B	0.000904
snoU13|ENSG00000239052.1	0.000904
SLC3A1	0.000904
PREPL	0.000904
CAMKMT	0.000668
SIX3	0.002135
SIX2	0.002135
SRBD1	0.000938
RN7SL414P	0.001248
PRKCE	0.00175
EPAS1	0.002034
TMEM247	0.002034
RN7SL817P	0.002034
ATP6V1E2	0.002034
RHOQ	0.002034
PIGF	0.001075
CRIPT	0.001075
SOCS5	0.001398
MCFD2	0.001398
TTC7A	0.001398
CALM2	0.001215
C2orf61	0.001215
BCYRN1	0.001584
EPCAM	0.001584
RN7SKP119	0.001584
MIR559	0.001343
MSH2	0.000827
KCNK12	0.002041
MSH6	0.002041
FBXO11	0.002041
RN7SKP224	0.002053
FOXN2	0.002053
PPP1R21	0.001522
STON1	0.002053
GTF2A1L	0.002417
LHCGR	0.002417
FSHR	0.003719
SNORA75|ENSG00000212580.1	0.001709
NRXN1	0.00061
MIR4431	0.000936
SCARNA16|ENSG00000251942.1	0.002222
ASB3	0.00085
CHAC2	0.00085
ERLEC1	0.00085
snoU13|ENSG00000238756.1	0.00087
MIR3682	0.00087
GPR75	0.00087
PSME4	0.001001
ACYP2	0.001474
C2orf73	0.001474
SPTBN1	0.001871

TSPYL6	0.001474
EML6	0.00119
RTN4	0.001576
CLHC1	0.00119
RPS27A	0.00119
MTIF2	0.00119
PRORSD1P	0.00119
CCDC88A	0.00119
CCDC104	0.000935
SMEK2	0.00119
SNORA12|ENSG00000212175.1	0.00119
PNPT1	0.000935
EFEMP1	0.00119
RN7SKP208	0.00119
MIR216A	0.00119
MIR217	0.00119
MIR216B	0.00119
CCDC85A	0.002044
RNA5SP93	0.00119
snoU13|ENSG00000238690.1	0.001553
SNORD78|ENSG00000212168.1	0.00091
VRK2	0.001559
FANCL	0.00253
RNA5SP94	0.00522
MIR4432	0.006507
BCL11A	0.004182
RN7SL361P	0.001759
PAPOLG	0.001008
RN7SL632P	0.001008
REL	0.00177
PUS10	0.00177
RNA5SP95	0.00177
PEX13	0.00177
KIAA1841	0.000574
C2orf74	0.000505
AHSA2	0.000761
USP34	0.000505
SNORA70B	0.000378
XPO1	0.000626
FAM161A	0.001703
CCT4	0.001703
COMMD1	0.00076
B3GNT2	0.001586
MIR5192	0.001586
RN7SL51P	0.001586
snoU13|ENSG00000238809.1	0.001586
RN7SL18P	0.000761
TMEM17	0.000761
EHBP1	0.0004
OTX1	0.000765
DBIL5P2	0.000857
WDPCP	0.001127
MDH1	0.001078
RPS4XP5	0.001078
UGP2	0.001748
ACA59|ENSG00000251775.1	0.001748
VPS54	0.001351
PELI1	0.001361

LINC00309	0.000683
MIR4433	0.000477
LGALSL	0.00074
AFTPH	0.000559
SERTAD2	0.000559
RN7SL341P	0.00074
RN7SL211P	0.00159
snoU13|ENSG00000238696.1	0.00159
SLC1A4	0.000569
CEP68	0.000799
RAB1A	0.000747
SNORA74|ENSG00000272025.1	0.000747
ACTR2	0.000747
SPRED2	0.000847
RN7SL635P	0.000847
Vault|ENSG00000251900.1	0.000847
MIR4778	0.000321
MEIS1	0.000451
ETAA1	0.000566
C1D	0.000786
WDR92	0.000786
PPP3R1	0.000451
PNO1	0.000786
CNRIP1	0.000451
PLEK	0.000451
APLF	0.00135
ARHGAP25	0.00135
FBXO48	0.00135
PROKR1	0.00135
BMP10	0.00179
GKN2	0.00179
GKN1	0.00179
ANTXR1	0.001479
MIR3126	0.00179
RNA5SP96	0.001479
GFPT1	0.00179
NFU1	0.001402
AAK1	0.000775
RN7SL604P	0.001402
SNORA36C	0.001402
ANXA4	0.000388
snoU13|ENSG00000238708.1	0.00103
GMCL1	0.000526
SNRNP27	0.000729
MXD1	0.000729
snoU13|ENSG00000238465.1	0.000634
snoU13|ENSG00000239072.1	0.000846
ASPRV1	0.000846
RN7SL470P	0.001117
PCBP1	0.001117
C2orf42	0.000933
TIA1	0.001369
PCYOX1	0.001369
SNRPG	0.001369
FAM136A	0.001369
TGFA	0.001369
ADD2	0.003304
FIGLA	0.003304

CLEC4F	0.003304
CD207	0.003304
VAX2	0.004082
ATP6V1B1	0.004082
snoU13|ENSG00000239064.1	0.004082
RN7SL160P	0.004082
ANKRD53	0.004082
TEX261	0.004082
NAGK	0.004776
MCEE	0.002403
MPHOSPH10	0.002403
PAIP2B	0.002403
ZNF638	0.002403
DYSF	0.004443
CYP26B1	0.002099
EXOC6B	0.001754
SNORD78|ENSG00000212378.1	0.002798
SPR	0.002798
EMX1	0.002798
SFXN5	0.002798
RAB11FIP5	0.002798
NOTO	0.002798
SMYD5	0.002798
CCT7	0.002798
PRADC1	0.002798
FBXO41	0.002798
EGR4	0.002627
ALMS1	0.00165
ALMS1P	0.00165
NAT8	0.00165
TPRKB	0.00165
DUSP11	0.00165
C2orf78	0.00165
STAMBP	0.00165
ACTG2	0.00165
DGUOK	0.002203
RNA5SP97	0.002806
TET3	0.002806
BOLA3	0.002203
MGC10955	0.002203
MOB1A	0.002203
MTHFD2	0.002203
SLC4A5	0.003119
DCTN1	0.003119
C2orf81	0.003119
WDR54	0.003119
RTKN	0.003119
INO80B	0.003119
WBP1	0.003119
MOGS	0.003119
CCDC142	0.003119
MRPL53	0.003119
TTC31	0.002203
LBX2	0.002203
PCGF1	0.002203
TLX2	0.002203
DQX1	0.002203
AUP1	0.002203

HTRA2	0.002203
LOXL3	0.002203
DOK1	0.002203
M1AP	0.002203
SEMA4F	0.002203
HK2	0.00165
POLE4	0.001562
TACR1	0.001562
MIR5000	0.001562
snoU13|ENSG00000238521.1	0.001917
EVA1A	0.001917
snoU109|ENSG00000238410.1	0.001917
GCFC2	0.00318
MRPL19	0.00318
U3|ENSG00000221638.1	0.00318
snoU13|ENSG00000239018.1	0.00318
RN7SKP203	0.000887
RN7SKP164	0.000867
LRRTM4	0.001289
RNA5SP98	0.001431
CTNNA2	0.001363
REG1A	0.000778
REG1B	0.000778
REG1P	0.000778
REG3A	0.000778
REG3G	0.000778
MIR4264	0.000778
LRRTM1	0.000991
RNA5SP99	0.003664
RN7SL201P	0.000788
SNORD112|ENSG00000252298.1	0.0019
FUNDC2P2	0.001931
SUCLG1	0.001569
DNAH6	0.001569
TRABD2A	0.001569
TMSB10	0.001569
KCMF1	0.004099
TCF7L1	0.000985
TGOLN2	0.002097
RETSAT	0.002097
ELMOD3	0.00268
RN7SL113P	0.003306
CAPG	0.003306
SH2D6	0.003306
RN7SL251P	0.003306
RN7SL830P	0.004919
MAT2A	0.004919
GGCX	0.003986
VAMP8	0.003986
RN7SL126P	0.003986
VAMP5	0.003289
RNF181	0.003289
C2orf68	0.003289
TMEM150A	0.003289
USP39	0.003289
SFTPB	0.003986
GNLY	0.003289
ATOH8	0.001851

RN7SKP83	0.00114
ST3GAL5	0.001475
POLR1A	0.001026
IMMT	0.001133
MIR4779	0.001133
MRPL35	0.001133
PTCD3	0.001133
REEP1	0.001486
SNORD94	0.001133
U8|ENSG00000202537.1	0.001486
SNORA19|ENSG00000251974.1	0.001486
KDM3A	0.001851
CHMP3	0.001026
RNF103	0.002096
RMND5A	0.001475
CD8A	0.001475
CD8B	0.001851
LINC00152	0.001851
PLGLB1	0.001851
PLGLB2	0.001851
RGPD1	0.001851
RGPD2	0.001851
snoU13|ENSG00000238979.1	0.003079
KRCC1	0.003079
SMYD1	0.003079
MIR4780	0.003079
FABP1	0.002411
THNSL2	0.002411
RNY4P15	0.00205
snoU13|ENSG00000239049.1	0.001499
FOXI3	0.001499
TEX37	0.001499
EIF2AK3	0.001499
RPIA	0.001499
ANKRD36BP2	0.001499
MIR4436A	0.00213
IGKC	0.001672
IGKJ1	0.001672
IGKJ2	0.001672
IGKJ3	0.001672
IGKJ4	0.001672
IGKJ5	0.001672
RNA5SP100	0.001672
SLC9B1P2	0.001672
ANKRD20A8P	0.001786
TEKT4	0.001786
RN7SL575P	0.002298
MAL	0.002298
MRPS5	0.002416
ZNF514	0.002416
ZNF2	0.002416
PROM2	0.002416
KCNIP3	0.001732
FAHD2A	0.001211
FLJ14082	0.000868
LINC00342	0.000868
TRIM43	0.000868
ANKRD36C	0.000449

FAHD2CP	0.000639
RN7SL210P	0.000639
GPAT2	0.000639
ADRA2B	0.000639
ASTL	0.000639
DUSP2	0.000639
STARD7	0.000639
TMEM127	0.002102
CIAO1	0.002102
SNRNP200	0.002102
ITPRIPL1	0.002102
NCAPH	0.002102
NEURL3	0.002102
ARID5A	0.002887
KANSL3	0.002887
FER1L5	0.002887
LMAN2L	0.002301
CNNM4	0.002011
MIR3127	0.002011
CNNM3	0.002011
ANKRD23	0.002011
ANKRD39	0.002011
SEMA4C	0.002011
FAM178B	0.002011
RNA5SP101	0.002252
snoU13|ENSG00000238760.1	0.002252
ACTR1B	0.00071
ANKRD36B	0.000971
ANKRD36	0.000971
COX5B	0.000971
FAHD2B	0.000971
RN7SL313P	0.000971
ZAP70	0.000582
TMEM131	0.001177
VWA3B	0.000963
CNGA3	0.001237
INPP4A	0.001363
COA5	0.001136
UNC50	0.001136
MGAT4A	0.000499
KIAA1211L	0.000322
TSGA10	0.000229
C2orf15	0.000229
LIPT1	0.000229
MRPL30	0.000229
MITD1	0.000229
LYG1	0.000229
LYG2	0.000229
TXNDC9	0.000229
EIF5B	0.000229
REV1	0.000272
AFF3	0.000123
LONRF2	0.000573
CHST10	0.000856
NMS	0.001903
RN7SL611P	0.001903
PDCL3	0.001903
snoU13|ENSG00000238328.1	0.001903

RN7SL360P	0.001903
NPAS2	0.000942
RPL31	0.001083
TBC1D8	0.000748
RN7SL548P	0.000486
CNOT11	0.000707
RNF149	0.000707
SNORD89	0.000707
MIR5696	0.000707
CREG2	0.000707
RFX8	0.001043
MAP4K4	0.000533
FLJ20373	0.00078
IL1R2	0.001298
IL1R1	0.000984
IL1RL2	0.000984
IL18R1	0.001862
IL1RL1	0.001862
IL18RAP	0.001969
MIR4772	0.001969
SLC9A4	0.001969
SLC9A2	0.002459
MFSD9	0.001955
TMEM182	0.001955
U3|ENSG00000199727.1	0.002826
SNORA72|ENSG00000207249.1	0.002574
POU3F3	0.002574
MRPS9	0.000591
GPR45	0.000349
TGFBRAP1	0.000306
C2orf49	0.000306
FHL2	0.000306
NCK2	0.002179
C2orf40	0.001348
UXS1	0.001348
PLGLA	0.003378
RGPD3	0.002547
CD8BP	0.002966
ST6GAL2	0.00115
GACAT1	0.000709
RGPD4	0.000709
SLC5A7	0.000709
SULT1C3	0.000511
SULT1C2	0.000511
SULT1C2P1	0.000511
SULT1C4	0.000511
GCC2	0.000456
LIMS1	0.000572
RANBP2	0.000511
CCDC138	0.000511
EDAR	0.000728
SH3RF3	0.000455
MIR4265	0.000527
MIR4266	0.000728
10-Sep	0.000984
SOWAHC	0.000984
BUB1	0.000984
LIMS3L	0.000984

LIMS3|ENSG00000256977.6	0.000984
LIMS3|ENSG00000257207.4	0.000984
LINC00116	0.000984
MALL	0.000984
MIR4267	0.000984
MIR4436B1	0.000984
MIR4436B2	0.000984
NPHP1	0.000984
RGPD5	0.000984
RGPD6	0.000984
ACOXL	0.001512
BCL2L11	0.002557
ANAPC1	0.002983
MERTK	0.002983
RN7SL297P	0.002983
TMEM87B	0.002983
FBLN7	0.002983
ZC3H8	0.002983
snoU13|ENSG00000238951.1	0.002983
ZC3H6	0.002983
RGPD8	0.004178
TTL	0.004178
POLR1B	0.005045
CHCHD5	0.005045
SLC20A1	0.005045
NT5DC4	0.006919
CKAP2L	0.006919
IL1A	0.005045
IL1B	0.005629
IL37	0.005629
IL36G	0.006007
IL36A	0.00477
IL36B	0.00477
IL36RN	0.00477
IL1F10	0.00477
IL1RN	0.006631
PSD4	0.004909
PAX8	0.002563
CBWD2	0.002722
FOXD4L1	0.002722
FAM138B	0.002722
RABL2A	0.002722
RPL23AP7	0.002722
WASH2P	0.002722
SLC35F5	0.002722
MIR4782	0.002722
snoU13|ENSG00000239077.1	0.003617
ACTR3	0.009286
U3|ENSG00000212182.1	0.009286
snoU13|ENSG00000238520.1	0.007645
DPP10	0.010524
DDX18	0.025476
HTR5BP	0.033589
CCDC93	0.033589
RN7SL111P	0.033589
INSIG2	0.033589
PTPN4	0.040769
snoU13|ENSG00000238368.1	0.040769

EPB41L5	0.04859
GLI2	0.04859
SAP130	0.044876
GAD1	0.045119
SLC25A12	0.04772
HAT1	0.04772
snoU13|ENSG00000239041.1	0.04772
RAPGEF4	0.04772
MLTK	0.04772
OLA1	0.04039
RN7SL65P	0.04784
CIR1	0.045045
SCRN3	0.045045
GPR155	0.045045
WIPF1	0.045045
CHRNA1	0.037348
CHN1	0.037348
ATF2	0.037348
MIR933	0.045119
ATP5G3	0.045119
KIAA1715	0.037348
EVX2	0.037348
HOXD13	0.037348
HOXD12	0.037348
HOXD10	0.029065
HOXD11	0.029065
HOXD9	0.029065
HOXD8	0.029065
HOXD3	0.029065
HOXD4	0.029065
MIR10B	0.029065
HOXD1	0.029065
MTX2	0.029065
RNU6ATAC14P	0.029065
RNA5SP112	0.017041
HNRNPA3	0.019313
NFE2L2	0.020762
MIR3128	0.024259
snoU13|ENSG00000238295.1	0.023239
AGPS	0.02146
TTC30B	0.034609
TTC30A	0.034609
PDE11A	0.037972
RBM45	0.030516
OSBPL6	0.025057
PRKRA	0.02326
DFNB59	0.02326
FKBP7	0.02326
PLEKHA3	0.02326
TTN	0.012005
CCDC141	0.013068
ACA59|ENSG00000252000.1	0.019236
SESTD1	0.013068
snoU13|ENSG00000238339.1	0.013068
ZNF385B	0.017582
MIR1258	0.013068
SNORA43|ENSG00000202216.1	0.013068
CWC22	0.013068

UBE2E3	0.019451
MIR4437	0.027611
ITGA4	0.027611
CERKL	0.044465
NEUROD1	0.036936
RNU6ATAC19P	0.036936
SSFA2	0.030533
PPP1R1C	0.030533
RNA5SP113	0.030533
PDE1A	0.025453
RN7SL267P	0.025453
DNAJC10	0.041673
snoU13|ENSG00000238306.1	0.049135
MIR548AE1	0.049135
ZNF804A	0.049135
FAM171B	0.047194
MYO1B	0.034966
MYL1	0.037003
LANCL1	0.037003
CPS1	0.015277
RNA5SP119	0.037521
RN7SL204P	0.04909
MYEOV2	0.039584
OTOS	0.039584
ANKMY1	0.03984
DUSP28	0.03984
RNPEPL1	0.03984
CAPN10	0.032156
GPR35	0.032156
AQP12A	0.026024
AQP12B	0.026024
KIF1A	0.028063
AGXT	0.022473
C2orf54	0.022473
SNED1	0.023559
MTERFD2	0.023559
PASK	0.018601
PPP1R7	0.018601
ANO7	0.018601
HDLBP	0.018601
2-Sep	0.017821
FARP2	0.017821
MIR3133	0.017821
STK25	0.014942
ATG4B	0.013991
BOK	0.013991
CXXC11	0.013991
D2HGDH	0.013991
DTYMK	0.013991
GAL3ST2	0.013991
ING5	0.013991
NEU4	0.013991
PDCD1	0.013991
RNA5SP122	0.013991
THAP4	0.013991
CHL1	4.98E-06
CNTN4	2.04E-06
CNTN6	4.98E-06

RN7SKP144	4.98E-06
RN7SL120P	4.98E-06
IL5RA	4.42E-06
SNORA43|ENSG00000253049.1	4.42E-06
TRNT1	4.42E-06
CRBN	4.42E-06
SUMF1	5.09E-07
LRRN1	9.69E-07
SETMAR	2.87E-07
ITPR1	4.77E-07
EGOT	8.82E-07
snoU13|ENSG00000239126.1	8.82E-07
BHLHE40	8.82E-07
ARL8B	8.82E-07
EDEM1	6.92E-07
MIR4790	1.01E-06
RN7SL553P	1.01E-06
GRM7	2.61E-07
RNU4ATAC17P	7.99E-07
LMCD1	1.03E-06
LINC00312	5.91E-07
SSUH2	5.91E-07
CAV3	5.91E-07
OXTR	5.91E-07
RAD18	1.03E-06
SNORA43|ENSG00000199815.1	1.13E-06
SRGAP3	1.32E-06
THUMPD3	1.96E-06
SETD5	1.03E-06
LHFPL4	2.33E-06
MTMR14	2.72E-06
CPNE9	2.14E-06
BRPF1	6.63E-06
OGG1	6.63E-06
CAMK1	6.63E-06
TADA3	6.63E-06
ARPC4	6.63E-06
TTLL3	6.63E-06
RPUSD3	3.68E-06
CIDEC	3.39E-06
JAGN1	3.39E-06
IL17RE	3.39E-06
IL17RC	3.39E-06
CRELD1	3.39E-06
PRRT3	3.39E-06
EMC3	2.10E-06
CIDECP	2.10E-06
FANCD2	2.10E-06
FANCD2OS	2.10E-06
BRK1	2.10E-06
snoU13|ENSG00000238642.1	2.10E-06
VHL	2.10E-06
snoU13|ENSG00000238345.1	2.10E-06
IRAK2	2.10E-06
TATDN2	2.42E-06
LINC00852	2.42E-06
GHRLOS	2.42E-06
GHRL	2.42E-06

SEC13	2.42E-06
ATP2B2	2.42E-06
MIR378B	2.42E-06
MIR885	1.73E-06
LINC00606	1.73E-06
SLC6A11	1.11E-06
SLC6A1	1.11E-06
HRH1	3.10E-07
ATG7	2.92E-07
VGLL4	2.92E-07
TAMM41	7.84E-07
RN7SL147P	5.24E-07
SYN2	5.24E-07
TIMP4	5.24E-07
PPARG	4.78E-07
TSEN2	1.00E-06
RNA5SP123	1.00E-06
C3orf83	5.24E-07
MKRN2	5.24E-07
RAF1	4.06E-07
snoU13|ENSG00000239140.1	7.71E-07
TMEM40	4.50E-07
CAND2	1.95E-06
RPL32	1.95E-06
SNORA7A	1.95E-06
IQSEC1	1.09E-06
NUP210	1.57E-06
HDAC11	1.82E-06
FBLN2	2.22E-06
LINC00620	1.82E-06
WNT7A	1.82E-06
FGD5P1	1.82E-06
TPRXL	1.82E-06
CHCHD4	1.82E-06
TMEM43	1.82E-06
XPC	1.82E-06
LSM3	1.82E-06
RNA5SP124	1.57E-06
SLC6A6	1.57E-06
GRIP2	9.69E-07
CCDC174	1.57E-06
C3orf20	1.57E-06
FGD5	2.56E-06
NR2C2	2.56E-06
MRPS25	2.56E-06
ZFYVE20	2.56E-06
COL6A4P1	2.56E-06
CAPN7	2.56E-06
SH3BP5	2.56E-06
snoU13|ENSG00000238891.1	2.56E-06
METTL6	2.56E-06
EAF1	2.56E-06
COLQ	2.96E-06
MIR4270	5.37E-06
RN7SL110P	4.25E-06
HACL1	1.82E-06
BTD	1.82E-06
ANKRD28	1.82E-06

MIR3134	1.82E-06
RN7SL4P	1.82E-06
MIR563	1.82E-06
GALNT15	1.67E-06
DPH3	5.12E-07
OXNAD1	5.59E-07
RFTN1	7.64E-07
LINC00690	3.55E-07
DAZL	3.55E-07
PLCL2	4.32E-07
MIR3714	4.32E-07
TBC1D5	3.38E-07
U7|ENSG00000271841.1	4.32E-07
SATB1	2.14E-07
KCNH8	1.31E-06
MIR4791	1.31E-06
EFHB	3.61E-07
RAB5A	3.61E-07
PP2D1	3.61E-07
KAT2B	6.94E-07
MIR3135A	6.94E-07
SGOL1	6.94E-07
RNY4P22	6.94E-07
ZNF385D	4.86E-06
HMGB1P5	1.60E-06
UBE2E2	1.60E-06
UBE2E1	1.26E-06
NKIRAS1	1.26E-06
RPL15	1.26E-06
NR1D2	2.58E-06
LINC00691	4.52E-06
THRB	2.54E-06
MIR4792	1.40E-06
RN7SL216P	4.41E-06
RNA5SP125	4.41E-06
RARB	5.34E-06
RNA5SP126	6.55E-06
SNORD5|ENSG00000272166.1	2.75E-06
TOP2B	2.75E-06
MIR4442	3.71E-06
NGLY1	5.49E-06
OXSM	5.49E-06
LINC00692	3.71E-06
LRRC3B	3.71E-06
NEK10	4.11E-06
SLC4A7	5.02E-06
RN7SL859P	5.02E-06
EOMES	2.22E-06
CMC1	2.96E-06
AZI2	1.78E-06
ZCWPW2	2.43E-06
LINC00693	3.90E-06
RBMS3	3.61E-06
U3|ENSG00000199927.1	9.41E-07
TGFBR2	9.41E-07
GADL1	1.90E-06
RNA5SP127	8.12E-07
STT3B	8.12E-07

OSBPL10	1.56E-06
ZNF860	8.12E-07
SNORA25|ENSG00000201701.1	2.90E-06
GPD1L	2.90E-06
snoU13|ENSG00000238646.1	2.90E-06
CMTM8	2.90E-06
CMTM7	2.90E-06
CMTM6	2.90E-06
DYNC1LI1	2.90E-06
CNOT10	2.90E-06
TRIM71	2.90E-06
CCR4	3.70E-06
GLB1	3.70E-06
TMPPE	3.70E-06
RN7SL296P	3.70E-06
CRTAP	3.70E-06
SUSD5	3.70E-06
FBXL2	4.39E-06
UBP1	3.70E-06
RNA5SP128	3.70E-06
CLASP2	3.70E-06
PDCD6IP	3.70E-06
ARPP21	3.89E-06
STAC	3.89E-06
RN7SKP227	5.12E-06
DCLK3	6.34E-06
HSPD1P6	6.34E-06
TRANK1	6.67E-06
RNU6ATAC4P	7.43E-06
EPM2AIP1	5.12E-06
MLH1	8.34E-06
LRRFIP2	5.12E-06
snoU13|ENSG00000238929.1	5.12E-06
GOLGA4	5.12E-06
RNA5SP129	5.12E-06
C3orf35	4.24E-06
ITGA9	5.06E-06
CTDSPL	5.06E-06
MIR26A1	5.06E-06
VILL	5.06E-06
PLCD1	5.06E-06
DLEC1	5.06E-06
ACAA1	5.06E-06
MYD88	5.06E-06
OXSR1	5.06E-06
SLC22A13	5.06E-06
SLC22A14	5.06E-06
XYLB	5.06E-06
ACVR2B	5.06E-06
EXOG	5.06E-06
SCN5A	5.06E-06
SCN10A	5.06E-06
SCN11A	1.40E-05
WDR48	7.47E-06
GORASP1	7.47E-06
TTC21A	7.47E-06
CSRNP1	7.47E-06
XIRP1	7.46E-06

CX3CR1	7.46E-06
CCR8	5.95E-06
SLC25A38	5.95E-06
RPSA	5.95E-06
SNORA62|ENSG00000202363.1	5.95E-06
SNORA6	5.95E-06
MOBP	5.95E-06
MYRIP	1.92E-06
RN7SL411P	1.92E-06
SNORA64|ENSG00000202517.1	1.61E-06
EIF1B	1.61E-06
ENTPD3	2.79E-06
RPL14	2.79E-06
ZNF619	2.79E-06
ZNF620	2.79E-06
ZNF621	2.79E-06
CTNNB1	5.11E-06
ULK4	2.77E-06
SCARNA21|ENSG00000252409.1	1.33E-06
RN7SKP58	1.46E-06
TRAK1	1.57E-06
U8|ENSG00000212145.2	1.57E-06
CCK	1.57E-06
LYZL4	1.57E-06
VIPR1	4.91E-06
SEC22C	4.91E-06
SS18L2	4.91E-06
NKTR	4.91E-06
ZBTB47	4.91E-06
RN7SL567P	4.91E-06
KLHL40	4.91E-06
CCDC13	4.91E-06
HHATL	4.91E-06
HIGD1A	4.59E-06
ACKR2	4.59E-06
KRBOX1	4.59E-06
CYP8B1	4.59E-06
ZNF662	4.59E-06
FAM198A	4.59E-06
GTDC2	4.59E-06
SNRK	3.41E-06
RN7SL517P	4.59E-06
ANO10	1.40E-05
ABHD5	7.65E-06
TOPAZ1	8.70E-06
TCAIM	8.70E-06
LINC00694	8.70E-06
ZNF445	8.70E-06
ZNF852	8.70E-06
ZKSCAN7	8.70E-06
ZNF660	8.70E-06
ZNF197	8.70E-06
ZNF35	8.70E-06
ZNF502	7.65E-06
ZNF501	7.65E-06
KIAA1143	7.65E-06
KIF15	7.65E-06
MIR564	7.65E-06

TMEM42	7.65E-06
TGM4	7.65E-06
ZDHHC3	7.65E-06
EXOSC7	7.65E-06
CLEC3B	7.65E-06
CDCP1	8.84E-06
TMEM158	7.65E-06
U3|ENSG00000202268.1	7.65E-06
LARS2	4.82E-06
LIMD1	4.82E-06
SACM1L	4.82E-06
RN7SL145P	4.82E-06
SLC6A20	4.82E-06
LZTFL1	4.82E-06
CCR9	4.82E-06
FYCO1	4.82E-06
CXCR6	4.82E-06
XCR1	4.82E-06
CCR3	5.79E-06
CCR1	5.79E-06
CCR2	5.79E-06
CCR5	5.79E-06
ACKR5	5.05E-06
LTF	5.05E-06
RTP3	5.05E-06
LRRC2	5.05E-06
LUZPP1	5.05E-06
TDGF1	2.84E-06
SNORD77|ENSG00000251967.1	3.30E-06
ALS2CL	3.30E-06
TMIE	3.30E-06
PRSS50	3.30E-06
PRSS46	3.30E-06
PRSS42	5.05E-06
PRSS44	5.05E-06
PRSS45	5.05E-06
MYL3	5.05E-06
PTH1R	5.05E-06
CCDC12	9.27E-06
NBEAL2	1.09E-05
SETD2	6.42E-06
snoU13|ENSG00000251938.1	9.27E-06
KIF9	3.30E-06
snoU13|ENSG00000239128.1	3.30E-06
KLHL18	5.92E-06
PTPN23	5.92E-06
SCAP	3.83E-06
snoU13|ENSG00000238350.1	5.92E-06
ELP6	3.83E-06
RN7SL870P	3.83E-06
CSPG5	3.83E-06
SMARCC1	8.81E-06
DHX30	1.17E-05
MAP4	8.35E-06
MIR1226	1.17E-05
RN7SL664P	3.93E-06
CDC25A	3.93E-06
MIR4443	3.93E-06

CAMP	3.93E-06
ZNF589	6.72E-06
NME6	6.72E-06
MIR2115	6.72E-06
SPINK8	6.72E-06
FBXW12	3.93E-06
RN7SL321P	3.93E-06
PLXNB1	3.93E-06
CCDC51	3.93E-06
TMA7	3.93E-06
ATRIP	5.67E-06
SHISA5	5.67E-06
TREX1	5.67E-06
PFKFB4	5.67E-06
UCN2	5.67E-06
COL7A1	5.67E-06
MIR711	7.09E-06
UQCRC1	1.01E-05
TMEM89	1.01E-05
SLC26A6	1.01E-05
CELSR3	1.01E-05
MIR4793	1.01E-05
NCKIPSD	1.01E-05
IP6K2	1.01E-05
PRKAR2A	1.01E-05
SLC25A20	1.01E-05
ARIH2OS	1.01E-05
ARIH2	1.01E-05
P4HTM	1.01E-05
WDR6	1.01E-05
DALRD3	1.01E-05
MIR191	1.01E-05
MIR425	1.01E-05
NDUFAF3	1.01E-05
IMPDH2	1.01E-05
QRICH1	5.67E-06
RN7SL182P	4.72E-06
QARS	4.72E-06
USP19	4.72E-06
LAMB2	4.72E-06
CCDC71	4.72E-06
KLHDC8B	4.72E-06
C3orf84	4.72E-06
CCDC36	7.05E-06
C3orf62	1.17E-05
MIR4271	1.17E-05
USP4	1.17E-05
GPX1	1.17E-05
RHOA	1.17E-05
TCTA	1.17E-05
AMT	1.17E-05
NICN1	1.17E-05
DAG1	1.17E-05
RNA5SP130	1.17E-05
BSN	1.04E-05
APEH	1.04E-05
MST1	1.04E-05
RNF123	1.04E-05

AMIGO3	8.49E-06
GMPPB	8.49E-06
IP6K1	8.49E-06
CDHR4	8.49E-06
FAM212A	8.49E-06
MIR5193	8.49E-06
UBA7	8.49E-06
TRAIP	8.49E-06
CAMKV	1.25E-05
RN7SL217P	1.25E-05
MST1R	1.25E-05
MON1A	1.25E-05
RBM6	8.18E-06
RBM5	8.18E-06
SEMA3F	1.17E-05
MIR566	1.17E-05
GNAT1	1.17E-05
SLC38A3	1.17E-05
GNAI2	1.17E-05
SEMA3B	1.17E-05
LSMEM2	1.17E-05
IFRD2	1.17E-05
HYAL3	1.17E-05
HYAL1	1.17E-05
NAT6	1.17E-05
HYAL2	1.17E-05
TUSC2	1.17E-05
RASSF1	1.17E-05
ZMYND10	1.17E-05
NPRL2	1.17E-05
CYB561D2	1.17E-05
TMEM115	1.17E-05
CACNA2D2	1.17E-05
RNA5SP131	1.17E-05
C3orf18	4.19E-06
HEMK1	5.82E-06
CISH	5.82E-06
MAPKAPK3	5.82E-06
DOCK3	5.82E-06
MIR4787	8.84E-06
MANF	1.20E-05
RBM15B	1.20E-05
VPRBP	1.20E-05
RAD54L2	9.79E-06
RNU6ATAC29P	9.79E-06
TEX264	9.79E-06
RNA5SP132	9.79E-06
GRM2	1.36E-05
IQCF6	9.96E-06
IQCF3	9.96E-06
IQCF2	9.96E-06
RN7SL504P	9.96E-06
IQCF5	9.96E-06
IQCF1	1.23E-05
RRP9	1.23E-05
PARP3	1.23E-05
GPR62	1.23E-05
PCBP4	1.23E-05

ABHD14B	1.23E-05
ABHD14A	1.23E-05
ACY1	1.82E-05
RPL29	1.82E-05
DUSP7	1.82E-05
LINC00696	1.82E-05
POC1A	1.23E-05
ALAS1	1.23E-05
TLR9	1.23E-05
TWF2	1.23E-05
PPM1M	9.96E-06
WDR82	9.96E-06
MIRLET7G	9.96E-06
GLYCTK	9.96E-06
MIR135A1	9.96E-06
DNAH1	6.37E-06
BAP1	6.37E-06
PHF7	6.37E-06
SEMA3G	9.96E-06
TNNC1	9.96E-06
NISCH	1.47E-05
STAB1	1.13E-05
NT5DC2	9.06E-06
SMIM4	2.06E-05
PBRM1	3.49E-05
RNU6ATAC16P	2.06E-05
GNL3	3.00E-05
SNORD19B|ENSG00000238862.1	3.00E-05
SNORD19B|ENSG00000252787.1	3.00E-05
SNORD19|ENSG00000212493.1	3.00E-05
SNORD19|ENSG00000222345.1	3.00E-05
SNORD69	3.00E-05
GLT8D1	3.00E-05
SPCS1	3.00E-05
NEK4	1.76E-05
ITIH1	1.76E-05
ITIH3	1.76E-05
ITIH4	1.76E-05
MUSTN1	1.76E-05
TMEM110	1.76E-05
SFMBT1	7.77E-06
RFT1	6.90E-06
PRKCD	6.90E-06
TKT	8.81E-06
snoU13|ENSG00000238565.1	1.40E-05
DCP1A	1.79E-05
RN7SL821P	1.40E-05
SNORD38|ENSG00000207109.1	1.79E-05
SNORA26|ENSG00000212608.1	1.79E-05
CACNA1D	2.48E-05
SNORD63|ENSG00000251987.1	2.83E-05
CHDH	3.04E-05
IL17RB	3.04E-05
ACTR8	3.04E-05
SELK	3.04E-05
CACNA2D3	2.13E-05
ESRG	1.76E-05
LRTM1	2.13E-05

WNT5A	2.13E-05
ERC2	1.79E-05
MIR3938	1.37E-05
RN7SKP45	2.13E-05
RNA5SP133	1.76E-05
CCDC66	2.13E-05
FAM208A	2.13E-05
ARHGEF3	2.13E-05
SPATA12	1.28E-05
IL17RD	1.28E-05
HESX1	1.28E-05
APPL1	1.28E-05
ASB14	1.28E-05
DNAH12	1.28E-05
snoU13|ENSG00000238905.1	1.28E-05
PDE12	1.28E-05
ARF4	7.51E-06
RNU6ATAC26P	1.10E-05
DENND6A	1.10E-05
SLMAP	4.22E-06
FLNB	1.25E-05
DNASE1L3	7.51E-06
ABHD6	7.51E-06
RPP14	7.51E-06
PXK	7.51E-06
PDHB	7.51E-06
KCTD6	7.51E-06
ACOX2	7.51E-06
FAM107A	1.02E-05
FAM3D	5.19E-06
C3orf67	5.19E-06
FHIT	2.51E-05
NPCDR1	3.06E-05
U3|ENSG00000212211.1	3.06E-05
PTPRG	4.18E-05
C3orf14	2.20E-05
FEZF2	2.20E-05
CADPS	2.47E-05
RN7SL863P	1.95E-05
LINC00698	6.21E-05
SYNPR	3.59E-05
RNA5SP134	5.53E-05
SNTN	7.30E-05
C3orf49	4.36E-05
THOC7	4.36E-05
ATXN7	2.65E-05
PSMD6	3.68E-05
U3|ENSG00000200222.1	5.17E-05
PRICKLE2	5.17E-05
ADAMTS9	2.92E-05
MAGI1	2.40E-05
SLC25A26	8.58E-05
RN7SL482P	8.58E-05
LRIG1	5.92E-05
KBTBD8	7.71E-05
SUCLG2	2.48E-05
FAM19A1	1.78E-05
FAM19A4	4.81E-05

RNA5SP135	2.26E-05
EOGT	2.00E-05
TMF1	3.74E-05
MIR3136	3.74E-05
UBA3	3.74E-05
ARL6IP5	3.74E-05
LMOD3	4.21E-05
FRMD4B	4.67E-05
MITF	6.25E-05
RN7SL418P	4.90E-05
FOXP1	6.31E-05
MIR1284	2.63E-05
EIF4E3	2.22E-05
GPR27	2.22E-05
PROK2	2.22E-05
RN7SL271P	1.23E-05
LINC00877	8.24E-05
LINC00870	8.24E-05
RYBP	9.51E-05
snoU13|ENSG00000238568.1	4.42E-05
RNA5SP136	2.18E-05
SHQ1	2.66E-05
GXYLT2	2.18E-05
PPP4R2	2.59E-05
EBLN2	1.81E-05
snoU13|ENSG00000238416.1	3.07E-05
PDZRN3	1.34E-05
CNTN3	3.07E-05
RN7SL294P	0.000288
FAM86DP	0.000136
FRG2C	0.000136
LINC00960	0.000136
MIR1324	0.000136
MIR4273	0.000136
RN7SL92P	0.000136
ZNF717	0.000136
ROBO2	5.99E-06
RN7SL647P	1.38E-05
RN7SKP61	1.59E-05
ROBO1	1.71E-05
RN7SL751P	1.20E-05
MIR3923	7.03E-06
GBE1	6.37E-06
LINC00971	5.12E-06
CADM2	1.06E-05
MIR5688	8.73E-06
snoZ40	5.32E-05
RN7SKP284	5.43E-05
VGLL3	0.000184
CHMP2B	0.000227
MIR4795	0.000284
POU1F1	0.000227
RNU6ATAC6P	6.33E-05
HTR1F	4.20E-05
CGGBP1	4.20E-05
ZNF654	4.20E-05
C3orf38	4.20E-05
EPHA3	0.000225

U3|ENSG00000212598.1	0.000428
ARL13B	0.000386
PROS1	0.000444
STX19	0.000563
DHFRL1	0.00583
NSUN3	0.005868
LINC00879	0.006134
MTHFD2P1	0.010189
MTRNR2L12	0.003614
EPHA6	0.004477
ARL6	0.003387
CRYBG3|ENSG00000233280.2	0.004624
CRYBG3|ENSG00000080200.5	0.003393
MINA	0.003393
GABRR3	0.003393
OR5AC1	0.003236
OR5AC2	0.003236
OR5H1	0.003236
OR5H14	0.003236
OR5H15	0.003236
OR5H6	0.005815
OR5H2	0.005815
OR5H8P	0.004432
OR5K4	0.004432
OR5K3	0.004482
OR5K1	0.003259
CLDND1	0.003259
OR5K2	0.003259
CPOX	0.004538
GPR15	0.003259
ST3GAL6	0.004252
DCBLD2	0.003328
LINC00973	0.004721
COL8A1	0.003471
CMSS1	0.003642
FILIP1L	0.003642
MIR3921	0.002541
TMEM30C	0.002541
SNORD61|ENSG00000238377.1	0.002541
TBC1D23	0.002541
NIT2	0.002541
TOMM70A	0.002541
LNP1	0.002541
TMEM45A	0.002222
GPR128	0.002222
SNORA31|ENSG00000252989.1	0.002222
TFG	0.002222
ABI3BP	0.001143
snoU13|ENSG00000238312.1	0.000813
IMPG2	0.000813
SENP7	0.00083
snoU13|ENSG00000238525.1	0.001183
TRMT10C	0.001183
PCNP	0.001183
ZBTB11	0.00083
RPL24	0.00083
CEP97	0.00083
NXPE3	0.00083

NFKBIZ	0.00083
ZPLD1	0.00048
MIR548AB	0.002197
ALCAM	0.000902
CBLB	0.001238
LINC00882	0.00435
LINC00883	0.002447
CCDC54	0.001792
BBX	0.001276
LINC00635	0.001805
LINC00636	0.001307
CD47	0.001307
IFT57	0.001576
HHLA2	0.001826
MYH15	0.002573
KIAA1524	0.002573
SNORA70|ENSG00000202379.1	0.002573
DZIP3	0.002451
RETNLB	0.003485
TRAT1	0.003485
DPPA2	0.003485
GUCA1C	0.003485
LINC00488	0.003485
MORC1	0.003485
U3|ENSG00000221633.1	0.003485
DPPA4	0.003377
MIR4445	0.002091
RNU6ATAC15P	0.003755
PVRL3	0.003558
CD96	0.005923
ZBED2	0.007717
PLCXD2	0.004632
PHLDB2	0.004632
ABHD10	0.004632
TAGLN3	0.007717
TMPRSS7	0.007329
C3orf52	0.007717
MIR567	0.007717
GCSAM	0.007717
SLC9C1	0.005923
CD200	0.005923
BTLA	0.005923
ATG3	0.006203
SLC35A5	0.006203
CCDC80	0.005996
CD200R1L	0.005996
CD200R1	0.005996
GTPBP8	0.004538
C3orf17	0.004538
SNORD112|ENSG00000253076.1	0.003268
BOC	0.003268
WDR52	0.003082
SPICE1	0.002926
SIDT1	0.00222
MIR4446	0.002926
RN7SL767P	0.00222
KIAA2018	0.00222
NAA50	0.00232

ATP6V1A	0.001745
GRAMD1C	0.001816
ZDHHC23	0.001816
KIAA1407	0.001816
QTRTD1	0.001745
DRD3	0.001816
ZNF80	0.001816
TIGIT	0.001816
MIR568	0.001816
ZBTB20	0.002401
MIR4796	0.001816
GAP43	0.001809
LSAMP	0.001223
RN7SL815P	0.001319
LINC00903	0.001317
RN7SL582P	0.001317
TUSC7	0.002016
MIR4447	0.002313
LINC00901	0.001581
IGSF11	0.000717
C3orf30	0.001041
UPK1B	0.001041
B4GALT4	0.001041
ARHGAP31	0.001308
TMEM39A	0.001308
POGLUT1	0.002468
TIMMDC1	0.001443
CD80	0.001443
ADPRH	0.001443
PLA1A	0.001443
POPDC2	0.001374
COX17	0.001267
MAATS1	0.001374
NR1I2	0.001096
GSK3B	0.001004
RN7SL762P	0.001316
RN7SL397P	0.001316
GPR156	0.001316
LRRC58	0.001316
FSTL1	0.001316
NDUFB4	0.002302
HGD	0.001675
RABL3	0.001675
GTF2E1	0.002451
STXBP5L	0.002595
MIR5682	0.003303
POLQ	0.002595
ARGFX	0.001815
FBXO40	0.001815
GOLGB1	0.001815
HCLS1	0.001815
RN7SL172P	0.001815
snoU13|ENSG00000238670.1	0.001815
IQCB1	0.003145
EAF2	0.003145
SLC15A2	0.003299
ILDR1	0.0026
CD86	0.0026

CASR	0.001927
SNORD112|ENSG00000252170.1	0.002454
CSTA	0.00147
CCDC58	0.00147
FAM162A	0.002172
WDR5B	0.002855
KPNA1	0.002916
PARP9	0.002843
DTX3L	0.003744
PARP15	0.003744
PARP14	0.0026
HSPBAP1	0.003744
snoU13|ENSG00000238480.1	0.003744
DIRC2	0.003744
SEMA5B	0.004469
PDIA5	0.008346
SEC22A	0.00609
ADCY5	0.004581
PTPLB	0.007625
MYLK	0.007625
SNORA5	0.007625
CCDC14	0.006088
ROPN1	0.005705
KALRN	0.005585
MIR5002	0.004655
UMPS	0.004655
MIR544B	0.004655
ITGB5	0.005624
MUC13	0.005624
HEG1	0.005624
RNA5SP137	0.005624
SLC12A8	0.005624
MIR5092	0.005624
ZNF148	0.00689
SNX4	0.005805
snoU13|ENSG00000238992.1	0.005805
OSBPL11	0.004013
MIR548I1	0.006749
ALG1L	0.006749
FAM86JP	0.006749
ROPN1B	0.005489
SLC41A3	0.005489
ALDH1L1	0.003938
U1|ENSG00000272020.1	0.003938
KLF15	0.004753
CCDC37	0.004753
ZXDC	0.004753
UROC1	0.004753
C3orf22	0.004753
CHST13	0.004753
RNA5SP138	0.004753
TXNRD3NB	0.004753
TXNRD3	0.004753
NUP210P1	0.004753
CHCHD6	0.004753
PLXNA1	0.004753
C3orf56	0.006503
TPRA1	0.012422

MCM2	0.012422
PODXL2	0.012422
ABTB1	0.012422
MGLL	0.012422
KBTBD12	0.013918
RNA5SP139	0.012422
SEC61A1	0.012422
RUVBL1	0.012422
EEFSEC	0.012422
MIR1280	0.008194
DNAJB8	0.008194
GATA2	0.008194
C3orf27	0.009666
RPN1	0.009666
SNORA24|ENSG00000207130.1	0.011369
RAB7A	0.011369
RN7SL698P	0.011369
ACAD9	0.009253
KIAA1257	0.009253
EFCC1	0.010958
GP9	0.010958
snoU13|ENSG00000252435.1	0.010958
snoU13|ENSG00000238874.1	0.010958
RAB43	0.010958
ISY1	0.010958
CNBP	0.010958
COPG1	0.0106
C3orf37	0.0106
H1FX	0.0106
RPL32P3	0.0106
SNORA7B	0.0106
EFCAB12	0.008936
MBD4	0.008936
IFT122	0.008936
RHO	0.008936
H1FOO	0.008936
PLXND1	0.015883
RN7SL752P	0.008936
TMCC1	0.006481
TRH	0.006481
ALG1L2	0.006481
FAM86HP	0.006481
COL6A4P2	0.00384
COL6A5	0.007197
COL6A6	0.007197
PIK3R4	0.009714
RN7SKP212	0.007005
ATP2C1	0.005928
ASTE1	0.005928
NEK11	0.008157
NUDT16P	0.006617
NUDT16	0.006617
MRPL3	0.00793
SNORA58|ENSG00000249020.1	0.00793
CPNE4	0.002248
MIR5704	0.00793
snoU13|ENSG00000238701.1	0.006617
ACPP	0.006617

DNAJC13	0.0098
ACAD11	0.00793
NPHP3	0.00793
ACKR4	0.00793
UBA5	0.00793
TMEM108	0.03608
BFSP2	0.005177
SNORA33|ENSG00000201827.1	0.00371
CDV3	0.00371
TOPBP1	0.00371
TFP1	0.00371
RNA5SP140	0.003083
TF	0.003083
SRPRB	0.002894
RAB6B	0.002894
C3orf36	0.002894
SLCO2A1	0.001716
RYK	0.002407
RPL39P5	0.002453
AMOTL2	0.002453
MIR4788	0.002453
ANAPC13	0.002453
CEP63	0.002453
EPHB1	0.00202
KY	0.002453
RNA5SP141	0.002453
U8|ENSG00000253004.1	0.004063
PPP2R3A	0.005221
MSL2	0.005221
PCCB	0.004494
STAG1	0.003175
RNY4P4	0.003958
SLC35G2	0.007217
NCK1	0.007217
IL20RB	0.007217
RNA5SP142	0.003175
SOX14	0.005172
CLDN18	0.0062
A4GNT	0.003634
DZIP1L	0.003634
DBR1	0.002911
ARMC8	0.002519
NME9	0.004306
MRAS	0.003689
ESYT3	0.006819
CEP70	0.006819
FAIM	0.003689
PIK3CB	0.001008
C3orf72	0.001506
FOXL2	0.001506
MRPS22	0.000876
PRR23A	0.001803
PRR23B	0.001803
PRR23C	0.001803
BPESC1	0.002137
COPB2	0.001517
RBP2	0.001276
RBP1	0.001276

NMNAT3	0.002607
RN7SKP124	0.003689
RN7SL724P	0.003689
CLSTN2	0.003634
TRIM42	0.005321
SLC25A36	0.003847
SPSB4	0.004552
ACPL2	0.004552
ZBTB38	0.004552
RASA2	0.003109
RNF7	0.010213
GRK7	0.00858
ATP1B3	0.00858
TFDP2	0.013764
GK5	0.00858
XRN1	0.007264
ATR	0.009036
RNA5SP143	0.010175
PLS1	0.007409
RN7SKP25	0.007409
TRPC1	0.006456
PCOLCE2	0.006456
PAQR9	0.006456
U2SURP	0.006456
CHST2	0.010175
PBX2P1	0.010175
SLC9A9	0.006456
C3orf58	0.004874
RNA5SP144	0.00487
PLOD2	0.011654
PLSCR4	0.017517
PLSCR2	0.017328
PLSCR1	0.012856
PLSCR5	0.017542
U3|ENSG00000251800.1	0.019348
ZIC4	0.008015
ZIC1	0.005859
AGTR1	0.01565
CPB1	0.024969
CPA3	0.028108
GYG1	0.028108
HLTF	0.028108
HPS3	0.023829
CP	0.027884
CPHL1P	0.046402
TM4SF18	0.046402
WWTR1	0.040912
COMMD2	0.044094
ANKUB1	0.044094
RNF13	0.044094
PFN2	0.040657
snoU13|ENSG00000243321.2	0.040657
TSC22D2	0.035237
SERP1	0.041821
EIF2A	0.041821
SELT	0.041821
FAM194A	0.041821
SIAH2	0.041821

FAM188B2	0.041821
CLRN1	0.041821
RNA5SP145	0.041821
GPR171	0.041821
IGSF10	0.034655
MIR5186	0.02806
AADACL2	0.030009
AADAC	0.023954
SUCNR1	0.023954
MBNL1	0.013201
TMEM14E	0.016646
P2RY1	0.01335
RAP2B	0.015983
RN7SL300P	0.015983
C3orf79	0.015983
U8|ENSG00000201398.1	0.025366
ARHGEF26	0.031893
DHX36	0.019711
snoU13|ENSG00000271922.1	0.019711
GPR149	0.019711
MME	0.010239
PLCH1	0.005217
C3orf33	0.006544
SLC33A1	0.006544
GMPS	0.008545
KCNAB1	0.01797
SSR3	0.022827
TIPARP	0.020013
LINC00886	0.020013
LEKR1	0.028424
RN7SKP177	0.028424
LINC00880	0.025143
LINC00881	0.025143
CCNL1	0.025143
RNA5SP146	0.025143
SHOX2	0.048085
RSRC1	0.049837
MLF1	0.039985
GFM1	0.030187
LXN	0.030187
RARRES1	0.031893
MFSD1	0.026353
IQCJ	0.032011
MIR3919	0.03295
SCHIP1	0.033676
IL12A	0.033676
C3orf80	0.03062
IFT80	0.026125
SMC4	0.018321
MIR15B	0.024027
TRIM59	0.018321
KPNA4	0.024027
SCARNA7	0.024027
KRT8P12	0.024027
ARL14	0.024027
SNORA72|ENSG00000207084.1	0.024027
PPM1L	0.03026
B3GALNT1	0.03026

NMD3	0.04142
SPTSSB	0.03026
OTOL1	0.031663
CT64	0.029627
snoU13|ENSG00000238398.1	0.022318
MIR1263	0.01441
MIR720	0.036996
SI	0.038434
SLITRK3	0.038434
ZBBX	0.043327
SERPINI2	0.042818
WDR49	0.040275
PDCD10	0.036386
SERPINI1	0.036386
GOLIM4	0.031264
EGFEM1P	0.030964
MIR551B	0.017183
MECOM	0.022806
ACTRT3	0.018001
TERC	0.018001
MYNN	0.018001
LRRC34	0.018001
LRRIQ4	0.018001
LRRC31	0.018001
SAMD7	0.017415
SEC62	0.017415
GPR160	0.017415
PHC3	0.015821
PRKCI	0.015821
SKIL	0.015821
CLDN11	0.008845
SLC7A14	0.01515
RNY5P3	0.010786
RPL22L1	0.010786
EIF5A2	0.010786
SLC2A2	0.013393
TNIK	0.017833
MIR569	0.013393
PLD1	0.017694
PP13439	0.019858
TMEM212	0.019858
snoU13|ENSG00000238359.1	0.019858
FNDC3B	0.020558
RN7SL141P	0.02458
GHSR	0.0225
TNFSF10	0.012762
NCEH1	0.015517
ECT2	0.014352
SNORA72|ENSG00000200355.1	0.014352
SPATA16	0.005586
snoU13|ENSG00000239141.1	0.006986
NLGN1	0.004129
RN7SKP234	0.006067
NAALADL2	0.008824
RN7SKP40	0.004347
MIR4789	0.00195
snoU13|ENSG00000271842.1	0.00195
RNA5SP147	0.00694

TBL1XR1	0.008396
LINC00501	0.00868
LINC00578	0.008438
RN7SKP52	0.011893
SNORA18|ENSG00000200288.1	0.011975
KCNMB2	0.002954
RNA5SP148	0.003736
ZMAT3	0.003506
PIK3CA	0.004962
SNORA25|ENSG00000201957.1	0.004962
KCNMB3	0.004533
ZNF639	0.004533
MFN1	0.003772
GNB4	0.005224
ACTL6A	0.005224
MRPL47	0.005224
NDUFB5	0.005224
snoU13|ENSG00000239096.1	0.005224
USP13	0.005872
PEX5L	0.008076
RNA5SP149	0.007465
U8|ENSG00000201810.1	0.011012
TTC14	0.011012
CCDC39	0.00767
RN7SL229P	0.00767
FXR1	0.005452
DNAJC19	0.00659
SOX2	0.011685
RN7SL703P	0.007001
RNA5SP150	0.007001
RN7SKP265	0.007001
ATP11B	0.007001
DCUN1D1	0.007001
MCCC1	0.005291
LAMP3	0.005291
MCF2L2	0.001889
B3GNT5	0.007001
RNA5SP151	0.007001
ABCC5	0.002556
AP2M1	0.002556
CYP2AB1P	0.002556
DVL3	0.002556
EIF2B5	0.001669
HTR3C	0.002556
HTR3D	0.002556
HTR3E	0.002556
KLHL24	0.002556
KLHL6	0.002556
LINC00888	0.002556
MAP6D1	0.002556
MIR4448	0.002556
PARL	0.002556
SNORA4|ENSG00000251730.1	0.002556
SNORA63|ENSG00000199363.1	0.002556
SNORA63|ENSG00000201229.1	0.002556
SNORA81|ENSG00000253092.1	0.002556
YEATS2	0.002556
ABCF3	0.004155

VWA5B2	0.003991
ALG3	0.003991
ECE2	0.003991
MIR1224	0.003991
CAMK2N2	0.003991
PSMD2	0.003991
EIF4G1	0.002884
SNORD66|ENSG00000212158.1	0.003991
FAM131A	0.002884
CLCN2	0.002884
POLR2H	0.002884
THPO	0.002821
CHRD	0.002821
EPHB3	0.002017
MAGEF1	0.002017
VPS8	0.002821
C3orf70	0.002821
EHHADH	0.002821
MAP3K13	0.001744
snoU13|ENSG00000239146.1	0.002017
TMEM41A	0.002017
LIPH	0.002017
SENP2	0.002364
IGF2BP2	0.003347
C3orf65	0.003164
MIR548AQ	0.002966
TRA2B	0.003469
RN7SL637P	0.003047
ETV5	0.003536
DGKG	0.002845
CRYGS	0.003536
TBCCD1	0.003536
DNAJB11	0.004491
AHSG	0.003889
FETUB	0.003889
HRG	0.005941
KNG1	0.005941
EIF4A2	0.006491
SNORD2|ENSG00000238942.1	0.004226
RFC4	0.004226
SNORA4|ENSG00000263776.1	0.004226
SNORA63|ENSG00000200320.1	0.004226
SNORA63|ENSG00000200418.1	0.004226
SNORA81|ENSG00000221420.2	0.004226
ADIPOQ	0.003039
ST6GAL1	0.004238
RPL39L	0.00489
RTP1	0.005202
MASP1	0.005202
RTP4	0.007286
snoU13|ENSG00000239093.1	0.009454
SST	0.010652
RTP2	0.010652
BCL6	0.010652
LPP	0.003539
MIR28	0.008586
TPRG1	0.004102
TP63	0.008411

MIR944	0.014893
LEPREL1	0.011015
RN7SL486P	0.010086
CLDN1	0.012886
CLDN16	0.012886
TMEM207	0.017469
IL1RAP	0.007644
RN7SKP296	0.009924
GMNC	0.005845
OSTN	0.007467
UTS2B	0.00948
CCDC50	0.006868
PYDC2	0.006868
RN7SKP222	0.005698
FGF12	0.00521
snoU13|ENSG00000238902.1	0.007512
MB21D2	0.005278
HRASLS	0.008456
ATP13A5	0.006243
ATP13A4	0.006243
OPA1	0.006243
RN7SL447P	0.006243
HES1	0.010337
RN7SL215P	0.010337
LINC00887	0.00748
CPN2	0.007951
LRRC15	0.007951
GP5	0.007951
ATP13A3	0.007951
LINC00884	0.007951
TMEM44	0.008459
LSG1	0.008459
FAM43A	0.008459
XXYLT1	0.008443
MIR3137	0.008459
RN7SL36P	0.008459
ACAP2	0.008459
APOD	0.008459
LINC00969	0.008459
MIR570	0.008459
MUC20	0.008459
MUC4	0.008459
PPP1R2	0.008459
RN7SL773P	0.008459
RNU6ATAC24P	0.008459
SDHAP1	0.008459
TNK2	0.008459
TFRC	0.007866
LINC00885	0.007866
ZDHHC19	0.007866
SLC51A	0.007866
PCYT1A	0.007866
TCTEX1D2	0.007866
TM4SF19	0.007866
UBXN7	0.007866
RN7SL434P	0.007866
RN7SL738P	0.007866
RNF168	0.007866

SMCO1	0.007866
WDR53	0.007866
FBXO45	0.007866
NRROS	0.005482
PIGX	0.005482
CEP19	0.005053
DLG1	0.003573
MFI2	0.005053
NCBP2	0.005053
PAK2	0.005053
PIGZ	0.005053
SENP5	0.005053
U4|ENSG00000272359.1	0.005053
MIR4797	0.003897
snoU13|ENSG00000238491.1	0.003897
BDH1	0.0071
KIAA0226	0.00465
MIR922	0.006573
FYTTD1	0.004173
LRCH3	0.00776
ANKRD18DP	0.00776
FAM157A	0.00776
IQCG	0.00776
LMLN	0.00776
RPL35A	0.00776
ABCA11P	2.27E-06
ATP5I	2.27E-06
CPLX1	2.27E-06
DGKQ	2.27E-06
FGFRL1	2.27E-06
GAK	2.27E-06
IDUA	2.27E-06
MFSD7	2.27E-06
MIR571	2.27E-06
MYL5	2.27E-06
PCGF3	2.27E-06
PDE6B	2.27E-06
PIGG	2.27E-06
RN7SL358P	2.27E-06
RNF212	2.27E-06
SLC26A1	2.27E-06
TMEM175	2.27E-06
ZNF141	2.27E-06
ZNF595	2.27E-06
ZNF718	2.27E-06
ZNF721	2.27E-06
ZNF732	2.27E-06
ZNF876P	2.27E-06
TMED11P	2.27E-06
SNORA48|ENSG00000212458.1	2.27E-06
SPON2	2.27E-06
CTBP1	2.27E-06
MAEA	1.36E-06
CRIPAK	2.52E-07
UVSSA	2.52E-07
FAM53A	1.73E-07
SLBP	7.66E-08
TMEM129	7.66E-08

TACC3	7.66E-08
FGFR3	7.66E-08
LETM1	7.66E-08
RN7SL671P	1.17E-07
WHSC1	1.17E-07
SCARNA22	1.97E-07
MIR943	1.97E-07
NELFA	1.97E-07
C4orf48	1.97E-07
NAT8L	1.97E-07
POLN	1.97E-07
HAUS3	3.38E-07
MXD4	3.38E-07
MIR4800	3.38E-07
ZFYVE28	3.38E-07
RN7SL589P	3.38E-07
RNF4	3.00E-07
FAM193A	1.82E-07
TNIP2	1.82E-07
SH3BP2	1.54E-07
ADD1	1.54E-07
MFSD10	2.21E-07
NOP14	2.21E-07
GRK4	2.21E-07
HTT	3.16E-07
MSANTD1	5.03E-07
RGS12	7.62E-07
HGFAC	4.05E-07
DOK7	4.05E-07
LRPAP1	4.05E-07
LINC00955	3.63E-07
ADRA2C	7.62E-07
FAM86EP	1.72E-06
OTOP1	3.72E-06
TMEM128	3.72E-06
LYAR	4.36E-06
ZBTB49	4.36E-06
NSG1	4.36E-06
STX18	2.37E-06
MSX1	2.26E-06
RN7SKP113	1.61E-06
CYTL1	1.61E-06
STK32B	2.15E-06
RN7SKP275	1.82E-06
C4orf6	2.26E-06
EVC2	2.52E-06
EVC	2.26E-06
CRMP1	3.13E-06
MIR378D1	3.13E-06
C4orf50	3.13E-06
JAKMIP1	5.26E-06
WFS1	5.26E-06
PPP2R2C	9.78E-06
MAN2B2	9.52E-06
MRFAP1	9.52E-06
S100P	9.52E-06
MRFAP1L1	9.52E-06
BLOC1S4	1.14E-05

KIAA0232	1.50E-05
TBC1D14	7.96E-06
RN7SKP292	7.28E-06
CCDC96	5.91E-06
TADA2B	5.91E-06
GRPEL1	7.28E-06
RN7SKP36	7.28E-06
SORCS2	8.16E-06
MIR4798	9.55E-06
MIR4274	9.55E-06
PSAPL1	9.55E-06
AFAP1	2.56E-05
ABLIM2	1.42E-05
MIR95	1.19E-05
SH3TC1	1.42E-05
HTRA3	1.42E-05
ACOX3	1.42E-05
RNA5SP152	1.42E-05
TRMT44	1.42E-05
GPR78	7.78E-06
CPZ	7.78E-06
DEFB131	8.55E-06
FAM90A26	8.55E-06
HMX1	8.55E-06
MIR548I2	8.55E-06
RNA5SP153	8.55E-06
USP17L10	8.55E-06
USP17L11	8.55E-06
USP17L12	8.55E-06
USP17L13	8.55E-06
USP17L15	8.55E-06
USP17L17	8.55E-06
USP17L18	8.55E-06
USP17L19	8.55E-06
USP17L20	8.55E-06
USP17L21	8.55E-06
USP17L22	8.55E-06
USP17L23	8.55E-06
USP17L24	8.55E-06
USP17L25	8.55E-06
USP17L26	8.55E-06
USP17L27	8.55E-06
USP17L28	8.55E-06
USP17L29	8.55E-06
USP17L30	8.55E-06
USP17L5	8.55E-06
SLC2A9	4.69E-06
DRD5	3.70E-06
RNA5SP154	3.70E-06
WDR1	5.24E-06
MIR3138	5.24E-06
RNA5SP155	5.24E-06
ZNF518B	1.00E-05
CLNK	7.09E-06
MIR572	9.06E-06
HS3ST1	9.06E-06
RNA5SP156	9.06E-06
HSP90AB2P	2.41E-05

RAB28	2.51E-05
BOD1L1	4.05E-05
MIR5091	4.05E-05
LINC00504	4.12E-05
SNORA63|ENSG00000202449.1	4.95E-05
CPEB2	5.70E-05
RN7SKP170	5.70E-05
C1QTNF7	3.54E-05
CC2D2A	3.54E-05
FBXL5	3.54E-05
FAM200B	3.94E-05
BST1	3.94E-05
CD38	6.09E-05
FGFBP1	1.58E-05
FGFBP2	2.57E-05
PROM1	2.57E-05
TAPT1	2.66E-05
ZEB2P1	3.23E-05
LDB2	9.32E-05
SNORA75|ENSG00000206780.1	0.000169
QDPR	0.000145
CLRN2	0.000145
snoU13|ENSG00000238536.1	0.000145
LAP3	0.000145
RN7SL315P	0.000145
MED28	0.000145
FAM184B	0.000145
DCAF16	0.000184
NCAPG	0.000184
LCORL	0.000184
RNA5SP157	0.00021
SLIT2	9.83E-05
PACRGL	8.34E-05
KCNIP4	0.000356
GPR125	0.001036
snoU13|ENSG00000238383.1	0.001036
GBA3	0.000637
PPARGC1A	0.000471
DHX15	0.000178
MIR573	0.000208
RN7SL16P	0.000138
SOD3	9.90E-05
CCDC149	9.90E-05
LGI2	0.000138
SEPSECS	0.000155
PI4K2B	0.000155
ZCCHC4	0.000155
ANAPC4	0.000155
SLC34A2	0.000264
SEL1L3	0.000254
SMIM20	0.000234
RBPJ	0.000262
CCKAR	0.000139
TBC1D19	0.000139
SNORD74|ENSG00000200999.1	0.000139
STIM2	0.000119
RN7SL101P	0.000283
PCDH7	0.00019

snoU13|ENSG00000238694.1	4.75E-06
ARAP2	1.12E-06
DTHD1	2.37E-06
KIAA1239	2.48E-06
MIR4801	2.48E-06
C4orf19	3.93E-06
RELL1	3.11E-06
PGM2	2.40E-06
TBC1D1	2.25E-06
PTTG2	2.25E-06
KLF3	5.68E-06
RNA5SP158	5.68E-06
TLR10	5.68E-06
TLR1	5.68E-06
TLR6	5.68E-06
FAM114A1	9.56E-06
MIR574	5.68E-06
TMEM156	3.13E-06
KLHL5	2.62E-06
WDR19	4.33E-06
RFC1	4.33E-06
KLB	4.33E-06
MIR5591	5.05E-06
RPL9	5.05E-06
LIAS	5.05E-06
UGDH	6.34E-06
SMIM14	6.34E-06
UBE2K	1.03E-05
RN7SL558P	1.03E-05
PDS5A	1.98E-05
RNA5SP159	1.98E-05
N4BP2	1.36E-05
SNORA51|ENSG00000201863.1	1.36E-05
RHOH	7.99E-06
CHRNA9	7.99E-06
RBM47	7.99E-06
MIR4802	7.99E-06
NSUN7	5.28E-06
APBB2	4.03E-06
snoU13|ENSG00000238351.1	4.89E-06
RNA5SP160	5.28E-06
UCHL1	3.68E-06
LIMCH1	3.36E-06
PHOX2B	3.65E-06
LINC00682	9.59E-06
TMEM33	1.50E-05
DCAF4L1	9.07E-06
SLC30A9	9.07E-06
BEND4	5.36E-06
SHISA3	3.56E-06
ATP8A1	1.82E-06
GRXCR1	3.63E-06
RN7SKP82	3.63E-06
RN7SL691P	1.92E-06
RN7SL193P	6.66E-06
KCTD8	1.07E-05
YIPF7	1.32E-05
GUF1	1.32E-05

GNPDA2	1.32E-05
RN7SKP199	8.30E-06
GABRG1	5.22E-06
GABRA2	2.85E-06
COX7B2	1.61E-06
GABRA4	1.27E-06
GABRB1	1.27E-06
snoU13|ENSG00000238301.1	1.80E-06
COMMD8	8.97E-07
ATP10D	8.97E-07
CORIN	8.97E-07
RN7SKP215	8.97E-07
NFXL1	1.62E-06
NIPAL1	1.82E-06
CNGA1	1.82E-06
TXK	5.85E-06
CWH43	2.15E-05
FRYL	2.15E-05
OCIAD1	2.15E-05
OCIAD2	2.15E-05
SLAIN2	2.15E-05
SLC10A4	2.15E-05
TEC	2.15E-05
ZAR1	2.15E-05
DCUN1D4	0.001393
LRRC66	0.001341
SGCB	0.000831
SPATA18	0.000831
snoU13|ENSG00000239068.1	0.001999
USP46	0.001399
DANCR	0.000816
MIR4449	0.000816
SNORA26|ENSG00000212588.1	0.000816
SNORA26|ENSG00000212490.1	0.000718
RASL11B	0.000689
SCFD2	0.000823
FIP1L1	0.002528
LNX1	0.002531
CHIC2	0.002583
GSX2	0.002583
snoU13|ENSG00000238753.1	0.002583
PDGFRA	0.003893
KIT	0.003691
RN7SL424P	0.004058
KDR	0.004518
RN7SL822P	0.005563
SRD5A3	0.007068
TMEM165	0.007068
CLOCK	0.007132
RN7SKP30	0.007068
PDCL2	0.007068
NMU	0.007068
EXOC1	0.009055
CEP135	0.008374
RNA5SP161	0.004105
KIAA1211	0.001756
RNA5SP162	0.002913
AASDH	0.002913

PPAT	0.003784
PAICS	0.004116
SRP72	0.004116
ARL9	0.004116
THEGL	0.002336
HOPX	0.002336
RN7SL492P	0.001936
RN7SL357P	0.001936
SPINK2	0.001936
REST	0.002886
NOA1	0.002886
POLR2B	0.002886
snoU13|ENSG00000238579.1	0.002886
IGFBP7	0.004786
snoU13|ENSG00000238541.1	0.005549
snoU13|ENSG00000238925.1	0.007334
MIR548AG1	0.004253
LPHN3	0.001433
TECRL	0.004302
EPHA5	0.008491
MIR1269A	0.000396
CENPC	0.003055
STAP1	0.003055
UBA6	0.003055
GNRHR	0.003964
SNORA62|ENSG00000202374.1	0.005895
TMPRSS11D	0.00239
TMPRSS11A	0.00239
TMPRSS11F	0.00235
TMPRSS11GP	0.00239
FTLP10	0.00235
TMPRSS11BNL	0.00235
TMPRSS11B	0.00235
YTHDC1	0.003492
TMPRSS11E	0.003492
UGT2A3	0.003492
UGT2B10	0.003492
UGT2B11	0.003492
UGT2B15	0.003492
UGT2B17	0.003492
UGT2B28	0.003492
UGT2B7	0.003492
UGT2B4	0.003713
UGT2A1	0.003289
UGT2A2	0.003289
SULT1B1	0.002763
SULT1E1	0.001877
CSN1S1	0.001877
CSN2	0.001877
STATH	0.001877
HTN3	0.001405
HTN1	0.001405
CSN1S2AP	0.001405
C4orf40	0.001405
ODAM	0.00194
FDCSP	0.001405
CSN3	0.000991
CABS1	0.000957

SMR3A	0.000957
SMR3B	0.001214
PROL1	0.000957
MUC7	0.000957
AMTN	0.001317
AMBN	0.001317
ENAM	0.001317
IGJ	0.001286
UTP3	0.001286
RUFY3	0.001432
snoU13|ENSG00000238318.1	0.001432
GRSF1	0.001502
MOB1B	0.001502
DCK	0.002124
SLC4A4	0.001363
GC	0.001392
RNA5SP163	0.001735
NPFFR2	0.001888
ADAMTS3	0.001187
RNU4ATAC9P	0.001161
RNU6ATAC5P	0.001662
COX18	0.001151
ANKRD17	0.001151
SNORA3|ENSG00000221639.1	0.001151
ALB	0.001886
AFP	0.001773
AFM	0.001773
RASSF6	0.002658
IL8	0.001599
CXCL6	0.001599
PF4V1	0.001599
CXCL1	0.001599
PF4	0.001599
PPBP	0.001599
CXCL5	0.001599
RN7SL218P	0.001599
CXCL3	0.001599
PPBPP2	0.001599
CXCL2	0.001599
MTHFD2L	0.001599
EPGN	0.001599
EREG	0.001599
AREG	0.001599
AREGB	0.001599
BTC	0.000758
PARM1	0.001128
RCHY1	0.002241
THAP6	0.002241
C4orf26	0.002241
CDKL2	0.001394
G3BP2	0.001394
USO1	0.001394
PPEF2	0.001776
NAAA	0.002113
SDAD1	0.00194
CXCL9	0.00194
ART3	0.001474
CXCL10	0.00194

CXCL11	0.001474
NUP54	0.002113
SCARB2	0.001949
FAM47E	0.001949
CCDC158	0.001585
SNORD50|ENSG00000199857.1	0.001585
SHROOM3	0.001397
MIR4450	0.002124
MIR548AH	0.002124
SOWAHB	0.002997
11-Sep	0.003172
CCNI	0.005252
CCNG2	0.005252
CXCL13	0.004979
SNORD75|ENSG00000221711.1	0.007401
CNOT6L	0.007401
MRPL1	0.007401
FRAS1	0.005652
ANXA3	0.004294
snoU13|ENSG00000238816.1	0.005579
BMP2K	0.006275
MIR5096	0.004732
PAQR3	0.004732
RN7SL127P	0.004732
NAA11	0.003276
GK2	0.003343
LINC00989	0.003343
OR7E94P	0.003343
PCAT4	0.004844
SNORA75|ENSG00000212620.1	0.004844
ANTXR2	0.005842
PRDM8	0.005842
FGF5	0.005842
C4orf22	0.003032
BMP3	0.003232
PRKG2	0.003232
RASGEF1B	0.001167
SNORA31|ENSG00000252762.1	0.001169
HNRNPD	0.000618
SNORD42|ENSG00000202440.1	0.000618
HNRNPDL	0.000618
ENOPH1	0.000618
TMEM150C	0.000618
LINC00575	0.000618
SCD5	0.000694
MIR575	0.000617
SEC31A	0.000897
THAP9	0.000833
LIN54	0.001111
snoR442|ENSG00000252834.1	0.000833
COPS4	0.001534
PLAC8	0.001534
COQ2	0.001534
HPSE	0.001534
HELQ	0.001534
MRPS18C	0.001534
FAM175A	0.001534
AGPAT9	0.001534

CDS1	0.000821
WDFY3	0.001305
RN7SL552P	0.000821
RN7SKP48	0.001631
ARHGAP24	0.000376
MIR4451	0.001101
MAPK10	0.000215
RN7SKP96	0.000329
MIR4452	0.000329
PTPN13	0.000436
SLC10A6	0.000852
C4orf36	0.000852
AFF1	0.001164
KLHL8	0.000655
MIR5705	0.000605
HSD17B13	0.000605
HSD17B11	0.000605
RN7SL681P	0.000605
NUDT9	0.000605
SPARCL1	0.000605
DSPP	0.001588
DMP1	0.001588
snoU13|ENSG00000238652.1	0.001205
IBSP	0.000873
MEPE	0.000786
SPP1	0.000786
PKD2	0.000786
ABCG2	0.000789
RNU6ATAC31P	0.000789
PPM1K	0.001204
HERC6	0.001204
HERC5	0.001204
HERC3	0.001204
PIGY	0.001204
PYURF	0.001204
RN7SKP244	0.001204
NAP1L5	0.001204
FAM13A	0.000887
TIGD2	0.001019
GPRIN3	0.001019
SNCA	0.001433
MMRN1	0.000973
CCSER1	2.12E-05
RN7SKP248	0.001027
GRID2	0.000436
RNA5SP164	0.000208
ATOH1	0.000177
SMARCAD1	0.000139
HPGDS	0.000139
PDLIM5	0.000218
BMPR1B	0.000136
UNC5C	0.00019
PDHA2	0.000296
RN7SKP28	0.000421
STPG2	0.000366
RAP1GDS1	0.000379
TSPAN5	0.000196
EIF4E	0.000129

METAP1	0.000142
MIR3684	0.000142
ADH5	0.000142
ADH4	0.000142
PCNAP1	0.000142
ADH6	0.000142
ADH1A	8.62E-05
ADH1B	8.62E-05
ADH1C	8.62E-05
ADH7	0.000123
C4orf17	9.71E-05
TRMT10A	8.62E-05
MTTP	8.62E-05
DAPP1	6.60E-05
LAMTOR3	9.48E-05
DNAJB14	9.48E-05
H2AFZ	9.48E-05
DDIT4L	9.48E-05
EMCN	0.000105
PPP3CA	5.66E-05
MIR1255A	0.00028
BANK1	0.000215
SLC39A8	0.000215
RN7SL728P	0.000215
NFKB1	0.000301
MANBA	0.000451
UBE2D3	0.000451
CISD2	0.000451
snoU13|ENSG00000238948.1	0.000451
SLC9B1	0.000502
SLC9B2	0.000301
BDH2	0.000301
CENPE	0.000301
TACR3	0.000112
CXXC4	5.89E-05
SNORA31|ENSG00000252136.1	5.89E-05
TET2	5.89E-05
RN7SL89P	5.89E-05
PPA2	8.67E-05
ARHGEF38	0.000183
INTS12	0.000235
GSTCD	0.000235
NPNT	0.000323
TBCK	0.000191
AIMP1	0.000191
GIMD1	0.000191
DKK2	2.48E-05
SNORD112|ENSG00000252566.1	0.000104
PAPSS1	1.15E-05
SGMS2	1.68E-05
CYP2U1	1.68E-05
HADH	1.65E-05
LEF1	1.82E-05
RPL34	5.44E-05
OSTC	5.44E-05
ETNPPL	5.44E-05
COL25A1	3.36E-05
SEC24B	6.36E-05

RN7SL55P	6.36E-05
MIR576	6.36E-05
CCDC109B	6.36E-05
CASP6	6.36E-05
PLA2G12A	6.36E-05
CFI	6.36E-05
GAR1	9.31E-05
RRH	6.36E-05
LRIT3	6.36E-05
EGF	4.31E-05
ELOVL6	3.46E-05
RN7SL275P	3.46E-05
ENPEP	2.41E-05
PITX2	2.33E-05
MIR297	4.88E-05
C4orf32	3.82E-05
AP1AR	3.82E-05
TIFA	3.82E-05
ALPK1	3.82E-05
NEUROG2	3.82E-05
C4orf21	3.82E-05
LARP7	3.82E-05
MIR302A	3.82E-05
MIR302B	3.82E-05
MIR302C	3.82E-05
MIR302D	3.82E-05
MIR367	3.82E-05
ANK2	5.05E-05
RN7SL184P	4.34E-05
CAMK2D	2.85E-05
ARSJ	5.14E-05
UGT8	3.15E-05
MIR577	2.53E-05
RN7SL808P	1.54E-05
NDST4	2.34E-05
MTRNR2L13	1.70E-05
TRAM1L1	2.69E-05
NT5C3AP1	1.22E-05
NDST3	2.86E-05
SNHG8	2.86E-05
PRSS12	2.86E-05
CEP170P1	4.36E-05
METTL14	3.54E-05
SEC24D	5.72E-05
SYNPO2	4.23E-05
MYOZ2	9.34E-05
USP53	9.34E-05
C4orf3	0.000108
FABP2	0.000108
PDE5A	6.35E-05
SNORA11|ENSG00000221245.1	0.00011
MAD2L1	6.35E-05
PRDM5	1.63E-05
NDNF	2.75E-05
RN7SKP137	2.75E-05
TNIP3	2.75E-05
QRFPR	2.75E-05
ANXA5	4.92E-05

TMEM155	3.54E-05
EXOSC9	3.54E-05
CCNA2	3.54E-05
BBS7	3.54E-05
TRPC3	3.54E-05
RN7SL335P	1.54E-05
KIAA1109	1.54E-05
ADAD1	1.54E-05
IL2	1.05E-05
IL21	1.08E-05
BBS12	1.08E-05
FGF2	6.60E-06
NUDT6	6.60E-06
SPATA5	2.89E-06
SPRY1	6.60E-06
ANKRD50	0.000112
FAT4	6.28E-05
INTU	6.28E-05
SLC25A31	6.28E-05
HSPA4L	6.28E-05
PLK4	9.36E-05
MFSD8	9.36E-05
C4orf29	9.36E-05
LARP1B	5.66E-05
PGRMC2	5.66E-05
snoU13|ENSG00000238802.1	5.45E-05
PHF17	5.45E-05
SCLT1	6.86E-05
C4orf33	7.43E-05
RNY5P4	2.20E-05
RN7SL205P	1.42E-05
SNORA70|ENSG00000252014.1	1.42E-05
PCDH10	6.93E-05
PABPC4L	4.29E-05
LINC00613	5.45E-05
PCDH18	4.60E-05
LINC00616	9.29E-05
SLC7A11	0.000133
LINC00498	0.000133
LINC00499	0.000133
LINC00500	0.000133
CCRN4L	0.000129
ELF2	9.80E-05
RN7SL382P	0.000129
RN7SL311P	8.49E-05
MGARP	8.49E-05
NDUFC1	8.49E-05
NAA15	8.49E-05
ACA64|ENSG00000239005.1	0.000113
RAB33B	0.000113
SETD7	0.000113
MGST2	0.000113
RN7SKP237	0.000113
MAML3	0.000113
RN7SKP253	0.000113
SCOC	0.000113
SNORD112|ENSG00000252300.1	0.000113
CLGN	0.000113

ELMOD2	0.000147
UCP1	0.000147
RN7SL152P	0.000147
TBC1D9	0.000147
RNF150	0.000179
ZNF330	0.000179
snoU13|ENSG00000238695.1	0.000179
IL15	0.00021
INPP4B	0.000166
USP38	0.000138
GAB1	9.03E-05
MIR3139	9.03E-05
SMARCA5	6.99E-05
GUSBP5	1.00E-04
FREM3	8.63E-05
GYPA	6.76E-05
GYPB	6.76E-05
GYPE	6.76E-05
HHIP	0.000116
ANAPC10	0.00011
RN7SKP235	0.00011
ABCE1	0.00011
OTUD4	0.00011
SMAD1	0.000194
MMAA	0.000194
C4orf51	0.000136
ZNF827	0.000136
LSM6	9.64E-05
SLC10A7	9.64E-05
POU4F2	0.000118
TTC29	0.000328
MIR548G	0.000138
EDNRA	0.000138
TMEM184C	0.000138
PRMT10	0.000108
ARHGAP10	0.000108
RNA5SP165	0.000108
MIR4799	0.000108
RN7SL254P	0.000108
NR3C2	2.87E-05
RNA5SP166	1.87E-05
RNA5SP167	7.38E-05
DCLK2	6.55E-05
LRBA	5.13E-05
MAB21L2	8.10E-05
RNA5SP168	7.90E-05
RPS3A	7.90E-05
SH3D19	4.17E-05
SNORD73A	7.90E-05
SNORD73	7.90E-05
PRSS48	7.44E-05
FAM160A1	0.000251
RN7SKP35	0.000256
PET112	0.000169
RNA5SP169	0.000158
RN7SL446P	0.000418
FBXW7	0.000167
MIR3140	0.000167

MIR4453	0.000167
TMEM154	0.000167
TIGD4	0.000167
ARFIP1	0.000209
FHDC1	0.000209
TRIM2	0.000183
MND1	0.000148
RN7SL419P	0.000148
KIAA0922	9.39E-05
TLR2	4.70E-05
RNF175	9.48E-05
SFRP2	9.48E-05
DCHS2	0.000144
PLRG1	0.000113
FGB	0.000113
FGA	0.000113
FGG	0.000113
LRAT	0.000127
RBM46	0.000127
NPY2R	8.36E-05
MAP9	8.36E-05
GUCY1A3	0.000286
GUCY1B3	0.000286
ASIC5	0.000286
TDO2	0.000286
CTSO	0.000286
PDGFC	0.000261
GLRB	0.000195
GRIA2	0.000195
FAM198B	0.000111
TMEM144	7.59E-05
RXFP1	0.000133
C4orf46	0.000133
ETFDH	0.000169
U3|ENSG00000271817.1	0.000169
PPID	0.000169
FNIP2	0.000104
C4orf45	0.000104
RAPGEF2	0.000153
U3|ENSG00000251979.1	0.000267
FSTL5	0.00056
NAF1	0.000196
NPY1R	0.000137
NPY5R	9.59E-05
TKTL2	0.000137
TMA16	9.58E-05
1-Mar	3.51E-05
RN7SKP105	0.000137
FAM218A	0.000158
TRIM61	0.000158
TRIM60	0.000123
TMEM192	6.08E-05
KLHL2	0.000158
MSMO1	0.000158
CPE	0.000158
MIR578	0.000158
TLL1	0.000148
RNA5SP170	0.000224

SPOCK3	5.08E-05
RNA5SP171	0.000119
RN7SL776P	8.72E-05
RN7SKP188	0.000103
ANXA10	0.000103
DDX60	0.000103
snoU13|ENSG00000238744.1	0.000103
DDX60L	0.000103
SNORA51|ENSG00000207171.1	0.000103
PALLD	0.000105
CBR4	7.29E-05
RNY4P17	0.000105
SH3RF1	7.29E-05
NEK1	3.55E-05
CLCN3	2.00E-05
C4orf27	1.56E-05
MFAP3L	1.56E-05
AADAT	1.56E-05
HSP90AA6P	7.58E-05
RNU6ATAC13P	7.58E-05
GALNTL6	4.11E-05
RN7SL253P	6.19E-05
GALNT7	6.88E-05
MIR548T	6.88E-05
HMGB2	4.95E-05
SAP30	4.95E-05
SCRG1	4.95E-05
HAND2	6.66E-05
FBXO8	0.000195
CEP44	0.000165
MIR4276	0.000195
HPGD	0.000195
GLRA3	0.000165
ADAM29	0.000378
GPM6A	3.86E-05
WDR17	0.000152
SNORA51|ENSG00000201516.1	0.000195
SPATA4	0.000195
ASB5	0.000195
SPCS3	0.000195
VEGFC	0.000136
RN7SKP136	0.000165
NEIL3	0.000198
AGA	0.000132
RNA5SP172	0.000132
snoU13|ENSG00000252388.1	0.000132
RNA5SP173	0.000132
SNORD65|ENSG00000212191.1	0.000132
LINC00290	1.82E-05
RN7SKP13	2.16E-05
TENM3	2.26E-05
MIR1305	3.25E-05
DCTD	3.81E-05
FAM92A1P2	3.81E-05
RN7SKP67	3.81E-05
U3|ENSG00000252048.1	3.81E-05
WWC2	2.61E-05
snoU13|ENSG00000238319.1	3.81E-05

CLDN22	2.61E-05
CLDN24	2.61E-05
snoU13|ENSG00000238596.1	2.61E-05
CDKN2AIP	2.61E-05
ING2	2.61E-05
snoU13|ENSG00000239116.1	2.26E-05
ENPP6	2.26E-05
RWDD4	2.26E-05
STOX2	2.26E-05
TRAPPC11	2.26E-05
RN7SL28P	3.89E-05
IRF2	2.58E-05
SNORD79	3.89E-05
CASP3	3.26E-05
CCDC111	3.26E-05
MLF1IP	3.26E-05
ACSL1	3.26E-05
HELT	3.26E-05
KIAA1430	3.26E-05
LRP2BP	3.26E-05
MIR3945	3.26E-05
MIR4455	3.26E-05
SLC25A4	3.26E-05
SNX25	3.26E-05
ANKRD37	2.71E-05
UFSP2	2.71E-05
C4orf47	2.71E-05
CCDC110	2.71E-05
PDLIM3	2.71E-05
SORBS2	4.63E-05
snoU13|ENSG00000239034.1	4.63E-05
TLR3	4.63E-05
FAM149A	3.22E-05
SNORA31|ENSG00000253013.1	3.22E-05
CYP4V2	3.22E-05
KLKB1	3.22E-05
F11	2.27E-05
MTNR1A	2.27E-05
FAT1	1.52E-05
DUX4L2	0.000109
DUX4L3	0.000109
DUX4L4	0.000109
DUX4L5	0.000109
DUX4L6	0.000109
DUX4L7	0.000109
DUX4	0.000109
FRG1	0.000109
FRG2	0.000109
HSP90AA4P	0.000109
RNA5SP174	0.000109
RNA5SP175	0.000109
TRIML1	0.000109
TRIML2	0.000109
ZFP42	0.000109
CDH6	0.025166
DROSHA	0.019244
C5orf22	0.019244
PDZD2	0.014699

snoU13|ENSG00000238864.1	0.010687
MIR4279	0.013185
GOLPH3	0.014784
MTMR12	0.013185
ZFR	0.010687
MIR579	0.011285
SUB1	0.012838
NPR3	0.005261
TARS	0.015164
ADAMTS12	0.011433
RXFP3	0.010004
AMACR	0.009433
C1QTNF3	0.009433
RAI14	0.009433
SLC45A2	0.009433
TTC23L	0.011732
RAD1	0.007459
BRIX1	0.007459
DNAJC21	0.007459
AGXT2	0.005777
PRLR	0.006437
U3|ENSG00000201368.1	0.010629
SPEF2	0.007152
IL7R	0.005122
CAPSL	0.008281
UGT3A1	0.008281
UGT3A2	0.014119
LMBRD2	0.014119
MIR580	0.014119
SKP2	0.020681
NADK2	0.020681
RANBP3L	0.014119
RNA5SP181	0.018774
SLC1A3	0.01428
NIPBL	0.029041
C5orf42	0.028512
RN7SL37P	0.019179
NUP155	0.019179
WDR70	0.017312
GDNF	0.017512
EGFLAM	0.015358
LIFR	0.013856
MIR3650	0.013856
OSMR	0.009011
RICTOR	0.009011
FYB	0.006935
C9	0.010878
DAB2	0.008397
LINC00603	0.013389
LINC00604	0.015591
SNORA63|ENSG00000199552.1	0.015312
PTGER4	0.01199
TTC33	0.01199
PRKAA1	0.01199
SNORA57|ENSG00000212567.1	0.01199
RPL37	0.01199
SNORD72	0.01199
CARD6	0.016195

C7	0.032093
MROH2B	0.028237
C6	0.036928
PLCXD3	0.03551
OXCT1	0.037159
GHR	0.029492
CCDC152	0.023457
SEPP1	0.020571
ANXA2R	0.027747
ZNF131	0.032128
NIM1	0.016892
HMGCS1	0.016892
CCL28	0.016892
C5orf28	0.016892
C5orf34	0.019416
PAIP1	0.019416
NNT	0.013684
FGF10	0.012102
RN7SL383P	0.012102
MRPS30	0.008099
HCN1	2.10E-05
EMB	8.26E-06
PARP8	6.87E-05
snoU13|ENSG00000238702.1	0.000573
ISL1	0.000374
RNA5SP182	0.00174
ITGA1	0.002924
PELO	0.003122
ITGA2	0.000587
MOCS2	0.000635
FST	0.001118
NDUFS4	0.001118
ARL15	0.001145
MIR581	0.000391
RN7SL801P	0.000391
MIR4459	0.000391
HSPB3	0.002151
SNX18	0.002156
ESM1	0.001826
GZMK	0.001826
GZMA	0.001542
CDC20B	0.001542
GPX8	0.001948
MIR449A	0.001296
MIR449B	0.001296
MIR449C	0.001296
MCIDAS	0.001542
CCNO	0.001542
DHX29	0.00229
SKIV2L2	0.001867
PPAP2A	0.001996
MIR5687	0.004444
SLC38A9	0.00201
DDX4	0.002132
RNA5SP183	0.00261
IL31RA	0.001582
IL6ST	0.001582
snoU13|ENSG00000238326.1	0.001582

ANKRD55	0.002733
RNA5SP184	0.002733
RNA5SP185	0.005378
RNU6ATAC2P	0.005378
MAP3K1	0.003333
snoU13|ENSG00000238717.1	0.003333
SETD9	0.003333
MIER3	0.003333
GPBP1	0.00402
ACTBL2	0.007113
snoU13|ENSG00000238899.1	0.007237
PLK2	0.003196
GAPT	0.00505
MIR548AE2	0.00505
RAB3C	0.004172
PDE4D	0.000487
MIR582	0.003203
FKSG52	0.003638
PART1	0.002988
DEPDC1B	0.001417
ELOVL7	0.000367
ERCC8	0.00049
NDUFAF2	0.000325
SMIM15	0.000325
ZSWIM6	0.00037
C5orf64	0.000431
RN7SKP157	8.94E-05
KIF2A	0.000152
DIMT1	0.000152
IPO11	0.000152
CKS1B|ENSG00000268942.1	0.000123
LRRC70	0.000186
HTR1A	0.001735
RNF180	0.001347
RGS7BP	0.001464
RN7SL169P	0.001464
FAM159B	0.001464
SREK1IP1	0.001464
CWC27	0.001367
ADAMTS6	0.000268
CENPK	4.97E-05
PPWD1	4.97E-05
TRIM23	3.16E-05
TRAPPC13	3.16E-05
SGTB	3.87E-05
NLN	3.87E-05
ERBB2IP	7.79E-05
SNORA76|ENSG00000252904.1	4.97E-05
SREK1	0.000186
snoU13|ENSG00000238400.1	0.000186
MAST4	3.87E-05
CD180	7.79E-05
PIK3R1	0.000159
U8|ENSG00000212249.2	0.000117
7SK|ENSG00000249352.3	0.000117
SLC30A5	0.000163
RN7SL103P	0.000163
SNORA50|ENSG00000220986.1	0.000163

CCNB1	0.000163
snoU13|ENSG00000238334.1	0.000163
CENPH	0.000163
MRPS36	0.000163
CDK7	0.000163
CCDC125	0.000163
TAF9	0.000135
RAD17	0.000135
MARVELD2	8.82E-05
GTF2H2B	1.93E-05
GTF2H2C	1.93E-05
GTF2H2	1.93E-05
GUSBP3	1.93E-05
NAIP	1.93E-05
OCLN	1.93E-05
RN7SL476P	1.93E-05
RN7SL616P	1.93E-05
RN7SL9P	1.93E-05
SERF1A	1.93E-05
SERF1B	1.93E-05
SMN1	1.93E-05
SMN2	1.93E-05
snoU13|ENSG00000238451.1	1.93E-05
snoU13|ENSG00000238740.1	1.93E-05
PMCHL2	3.75E-05
BDP1	0.000142
MCCC2	0.000112
CARTPT	0.000118
MAP1B	0.000222
MIR4803	0.000222
MRPS27	0.000133
RN7SL153P	0.000133
PTCD2	0.000133
ZNF366	0.000133
TNPO1	0.000113
MIR4804	0.000113
FCHO2	0.000129
TMEM171	0.000129
TMEM174	0.00017
FOXD1	0.000255
BTF3	0.000255
ANKRA2	0.000255
UTP15	0.000255
ARHGEF28	0.000129
RN7SL814P	6.17E-05
ENC1	3.65E-05
HEXB	1.92E-05
GFM2	2.59E-05
NSA2	3.47E-05
FAM169A	3.47E-05
SNORA40|ENSG00000212363.1	1.35E-05
GCNT4	1.35E-05
ANKRD31	2.59E-05
COL4A3BP	1.80E-05
HMGCR	1.80E-05
POLK	1.15E-05
ANKDD1B	1.15E-05
POC5	8.33E-06

SV2C	4.13E-06
RNA5SP186	8.33E-06
IQGAP2	1.94E-05
F2RL2	2.78E-05
F2R	4.31E-05
F2RL1	4.31E-05
RN7SL208P	4.31E-05
snoU13|ENSG00000238688.1	4.31E-05
S100Z	1.94E-05
RNU6ATAC36P	1.94E-05
CRHBP	1.94E-05
AGGF1	1.94E-05
ZBED3	1.94E-05
SNORA47	1.94E-05
PDE8B	2.03E-05
WDR41	2.28E-05
OTP	2.28E-05
TBCA	1.43E-05
AP3B1	1.12E-05
SCAMP1	1.43E-05
LHFPL2	2.97E-05
ARSB	9.12E-05
DMGDH	9.86E-05
BHMT2	4.90E-05
BHMT	4.90E-05
RNY3P1	4.90E-05
SNORA18|ENSG00000206592.1	4.90E-05
JMY	3.48E-05
HOMER1	3.48E-05
PAPD4	4.90E-05
CMYA5	4.50E-05
MTX3	4.50E-05
THBS4	4.50E-05
SERINC5	5.51E-05
snoU13|ENSG00000239159.1	4.50E-05
SNORA31|ENSG00000251828.1	2.99E-05
SPZ1	2.09E-05
ZFYVE16	2.25E-05
FAM151B	2.37E-05
ANKRD34B	2.37E-05
DHFR	2.37E-05
MTRNR2L2	2.37E-05
MSH3	2.37E-05
RASGRF2	2.37E-05
CKMT2	5.58E-05
ZCCHC9	3.60E-05
ACOT12	3.60E-05
SSBP2	1.69E-05
ATG10	1.69E-05
RN7SL378P	2.36E-05
RPS23	1.69E-05
ATP6AP1L	1.21E-05
TMEM167A	3.04E-05
SCARNA18|ENSG00000238835.1	2.27E-05
XRCC4	1.95E-05
VCAN	5.92E-05
HAPLN1	2.27E-05
RN7SKP295	2.27E-05

EDIL3	6.76E-05
NBPF22P	5.95E-05
COX7C	5.34E-05
MIR3607	5.34E-05
RN7SKP34	3.61E-05
MIR4280	3.61E-05
RASA1	1.36E-05
RN7SL629P	2.30E-05
CCNH	1.45E-05
TMEM161B	3.65E-05
RNA5SP187	3.65E-05
SNORA70|ENSG00000206958.1	2.37E-05
LINC00461	2.03E-05
MEF2C	3.46E-05
MIR3660	3.38E-05
CETN3	7.48E-05
MBLAC2	3.10E-05
POLR3G	4.73E-05
LYSMD3	4.73E-05
GPR98	2.56E-05
LUCAT1	2.56E-05
ARRDC3	2.56E-05
NR2F1	3.13E-05
FAM172A	2.68E-05
MIR2277	3.13E-05
POU5F2	2.25E-05
KIAA0825	3.01E-05
ANKRD32	2.53E-05
MCTP1	1.70E-05
FAM81B	1.69E-05
TTC37	3.22E-05
ARSK	3.22E-05
GPR150	3.22E-05
RFESD	3.22E-05
SPATA9	3.22E-05
RHOBTB3	3.22E-05
GLRX	3.22E-05
C5orf27	3.22E-05
ELL2	3.22E-05
MIR583	3.37E-05
PCSK1	3.31E-05
CAST	2.34E-05
ERAP1	2.34E-05
ERAP2	3.50E-05
LNPEP	2.53E-05
LIX1	3.66E-05
RIOK2	3.66E-05
RGMB	3.28E-05
CHD1	3.28E-05
FAM174A	2.59E-05
RN7SKP62	8.69E-05
ST8SIA4	8.69E-05
MIR548P	8.69E-05
RN7SL802P	3.41E-05
RNA5SP188	0.00011
SLCO4C1	0.00011
RN7SKP68	0.00011
SLCO6A1	8.23E-05

LINC00492	5.32E-05
LINC00491	5.32E-05
PAM	6.86E-05
GIN1	8.46E-05
PPIP5K2	6.50E-05
C5orf30	3.63E-05
NUDT12	3.98E-05
RN7SL255P	4.88E-05
RNA5SP189	1.37E-05
SNORA31|ENSG00000252337.1	2.99E-05
EFNA5	3.50E-05
RN7SL782P	8.36E-06
RN7SKP122	8.36E-06
FBXL17	2.01E-05
FER	1.24E-05
PJA2	6.09E-06
MAN2A1	4.32E-06
RN7SKP230	2.90E-06
TMEM232	2.74E-06
MIR548F3	4.14E-06
SNORA51|ENSG00000207177.1	2.75E-06
SLC25A46	2.75E-06
TSLP	2.15E-06
WDR36	2.15E-06
CAMK4	2.66E-06
STARD4	2.08E-06
NREP	4.40E-06
RN7SKP57	2.08E-06
EPB41L4A	1.81E-06
SNORA13	3.44E-06
FLJ11235	4.06E-06
APC	1.14E-05
SRP19	2.81E-05
REEP5	1.76E-05
ZRSR1	2.81E-05
DCP2	1.28E-05
MCC	1.53E-05
TSSK1B	1.22E-05
RNU4ATAC13P	1.36E-05
YTHDC2	1.49E-05
RN7SKP89	4.56E-06
KCNN2	7.31E-06
TRIM36	4.70E-06
PGGT1B	6.09E-06
CCDC112	6.39E-06
FEM1C	1.17E-05
TICAM2	1.86E-05
TMED7	2.15E-05
CDO1	7.68E-06
ATG12	6.39E-06
AP3S1	6.39E-06
AQPEP	6.39E-06
ARL14EPL	6.39E-06
COMMD10	3.84E-06
SEMA6A	7.68E-06
DTWD2	7.57E-06
DMXL1	4.00E-06
snoU13|ENSG00000239011.1	7.57E-06

MIR5706	7.57E-06
TNFAIP8	9.24E-06
RN7SL174P	9.24E-06
RNA5SP190	1.21E-05
HSD17B4	1.24E-05
snoU13|ENSG00000239084.1	1.21E-05
snoU13|ENSG00000239067.1	1.12E-05
FAM170A	1.12E-05
PRR16	4.88E-06
FTMT	4.14E-06
SRFBP1	5.87E-06
LOX	9.40E-06
ZNF474	9.40E-06
SNCAIP	9.40E-06
SNX2	9.40E-06
snoU13|ENSG00000252295.1	9.40E-06
SNX24	9.40E-06
snoU13|ENSG00000239103.1	1.49E-05
PPIC	1.49E-05
RN7SL689P	1.49E-05
RN7SL711P	1.49E-05
PRDM6	1.49E-05
CEP120	3.21E-05
CSNK1G3	2.74E-05
ZNF608	6.58E-05
RN7SKP117	6.58E-05
GRAMD3	4.32E-05
ALDH7A1	2.67E-05
PHAX	2.67E-05
C5orf48	2.67E-05
LMNB1	1.71E-05
3-Mar	1.71E-05
C5orf63	1.71E-05
MEGF10	1.71E-05
PRRC1	1.71E-05
CTXN3	3.34E-05
SLC12A2	3.98E-05
FBN2	5.70E-05
SLC27A6	3.08E-05
ISOC1	1.79E-05
MIR4633	1.79E-05
MIR4460	1.92E-05
ADAMTS19	1.92E-05
KIAA1024L	1.42E-05
CHSY3	1.42E-05
RNU6ATAC10P	2.22E-05
RNA5SP191	1.42E-05
HINT1	1.46E-05
LYRM7	1.46E-05
CDC42SE2	1.83E-05
RAPGEF6	2.54E-05
FNIP1	1.61E-05
ACSL6	6.19E-06
IL3	5.78E-06
CSF2	5.78E-06
snoZ6|ENSG00000253067.1	5.78E-06
P4HA2	9.05E-06
PDLIM4	9.05E-06

SLC22A4	1.33E-05
MIR3936	1.33E-05
SLC22A5	1.33E-05
C5orf56	1.33E-05
IRF1	1.33E-05
IL5	1.33E-05
RAD50	1.33E-05
IL13	2.29E-05
IL4	2.29E-05
KIF3A	1.46E-05
CCNI2	1.46E-05
8-Sep	1.62E-05
SOWAHA	1.62E-05
SHROOM1	2.38E-05
RNA5SP192	2.38E-05
GDF9	2.38E-05
UQCRQ	2.38E-05
LEAP2	2.38E-05
AFF4	1.54E-05
ZCCHC10	1.54E-05
HSPA4	9.75E-06
FSTL4	9.75E-06
snoU13|ENSG00000238796.1	9.75E-06
C5orf15	9.75E-06
VDAC1	9.75E-06
TCF7	9.75E-06
SKP1	1.03E-05
PPP2CA	1.03E-05
CDKL3	1.03E-05
MIR3661	1.03E-05
UBE2B	1.03E-05
CDKN2AIPNL	1.03E-05
RN7SL541P	1.03E-05
PHF15	1.03E-05
SAR1B	9.75E-06
SEC24A	6.17E-06
CAMLG	3.81E-06
DDX46	3.81E-06
C5orf24	7.74E-06
TXNDC15	7.74E-06
PCBD2	7.74E-06
MIR4461	7.74E-06
CATSPER3	7.74E-06
PITX1	1.21E-05
H2AFY	1.90E-05
C5orf20	1.90E-05
TIFAB	1.90E-05
NEUROG1	1.41E-05
CXCL14	1.41E-05
MIR5692C1	1.41E-05
SLC25A48	1.77E-05
IL9	1.41E-05
LECT2	1.41E-05
FBXL21	1.41E-05
TGFBI	2.19E-05
SMAD5	2.19E-05
TRPC7	2.19E-05
RNA5SP193	2.97E-05

SPOCK1	3.35E-05
KLHL3	4.29E-05
MIR874	8.59E-05
HNRNPA0	6.50E-05
NPY6R	6.50E-05
MYOT	6.50E-05
PKD2L2	6.50E-05
FAM13B	6.50E-05
WNT8A	9.93E-05
NME5	9.93E-05
snoU13|ENSG00000238605.1	9.93E-05
BRD8	8.35E-05
KIF20A	8.35E-05
CDC23	8.35E-05
GFRA3	8.35E-05
RN7SL682P	8.35E-05
CDC25C	8.35E-05
FAM53C	8.35E-05
KDM3B	8.35E-05
REEP2	8.35E-05
EGR1	8.35E-05
ETF1	0.000113
HSPA9	0.000188
SNORD63|ENSG00000222937.1	0.000173
SNORD63|ENSG00000206989.1	0.000173
CTNNA1	0.000138
snoU13|ENSG00000238745.1	0.000188
RN7SL867P	8.95E-05
LRRTM2	6.48E-05
SIL1	7.98E-05
RNA5SP194	4.25E-05
MATR3	5.87E-05
SNORA74|ENSG00000252213.1	5.87E-05
SNORA74A	7.92E-05
RNA5SP195	7.92E-05
RN7SKP64	7.92E-05
PAIP2	7.92E-05
SLC23A1	7.92E-05
MZB1	7.92E-05
PROB1	7.92E-05
SPATA24	7.92E-05
DNAJC18	7.92E-05
ECSCR	7.92E-05
TMEM173	7.92E-05
UBE2D2	7.92E-05
CXXC5	0.000149
PSD2	0.00011
NRG2	0.00011
PURA	0.00011
IGIP	0.00011
CYSTM1	8.71E-05
PFDN1	8.71E-05
HBEGF	8.71E-05
SLC4A9	8.71E-05
ANKHD1	8.71E-05
SNORD45|ENSG00000200051.1	8.71E-05
SRA1	8.71E-05
EIF4EBP3	8.71E-05

APBB3	8.71E-05
SLC35A4	8.71E-05
SNORA27|ENSG00000200235.1	8.71E-05
CD14	8.71E-05
NDUFA2	8.71E-05
TMCO6	8.71E-05
IK	8.71E-05
MIR3655	8.71E-05
WDR55	8.71E-05
DND1	8.71E-05
HARS	8.71E-05
HARS2	8.71E-05
ZMAT2	8.71E-05
PCDHA1	8.71E-05
PCDHA2	8.71E-05
PCDHA3	8.71E-05
PCDHA4	8.71E-05
PCDHA10	8.71E-05
PCDHA11	8.71E-05
PCDHA14	0.000115
PCDHA5	8.71E-05
PCDHA6	8.71E-05
PCDHA7	8.71E-05
PCDHA8	8.71E-05
PCDHA9	8.71E-05
PCDHA12	8.71E-05
PCDHA13	8.71E-05
PCDHAC1	8.71E-05
PCDHAC2	8.71E-05
PCDHB1	0.00013
PCDHB2	9.94E-05
PCDHB3	7.80E-05
PCDHB17	9.94E-05
PCDHB4	9.94E-05
PCDHB5	9.94E-05
PCDHB6	9.94E-05
PCDHB7	7.08E-05
PCDHB16	7.08E-05
PCDHB8	7.08E-05
PCDHB10	0.000105
PCDHB11	0.000105
PCDHB12	0.000105
PCDHB13	0.000105
PCDHB14	0.000105
PCDHB18	0.000105
PCDHB15	0.000105
SLC25A2	8.10E-05
TAF7	8.10E-05
PCDHGA1	8.94E-05
PCDHGA2	8.94E-05
PCDHGA3	0.000123
PCDHGB1	0.000123
PCDHGA4	0.000123
PCDHGB2	0.000123
PCDHGA5	0.000123
PCDHGB3	0.000123
PCDHGA6	0.000123
PCDHGA7	0.000123

PCDHGB4	0.000123
PCDHGA8	0.000123
PCDHGA9	0.000123
PCDHGA10	0.000123
PCDHGA11	0.000123
PCDHGB6	0.000123
PCDHGB7	0.000123
PCDHGB8P	0.000113
PCDHGA12	0.000123
PCDHGC3	0.000123
RN7SL68P	0.000123
PCDHGC4	0.000123
PCDHGC5	0.000123
DIAPH1	0.000123
HDAC3	0.000163
RELL2	0.000163
FCHSD1	0.000163
ARAP3	0.000163
PCDH1	0.000326
KIAA0141	0.000224
PCDH12	0.000224
RNF14	0.000261
GNPDA1	0.000261
NDFIP1	5.95E-05
SPRY4	2.21E-05
FGF1	2.97E-05
ARHGAP26	5.52E-05
NR3C1	4.63E-05
MIR5197	7.03E-05
HMHB1	9.52E-05
RN7SL87P	8.47E-05
YIPF5	8.47E-05
KCTD16	0.000117
RN7SKP246	8.53E-05
PRELID2	6.60E-05
GRXCR2	6.60E-05
SH3RF2	9.52E-05
PLAC8L1	9.52E-05
LARS	8.47E-05
RBM27	9.32E-05
POU4F3	9.32E-05
TCERG1	5.79E-05
GPR151	5.79E-05
RNA5SP196	5.79E-05
PPP2R2B	8.27E-05
RN7SL791P	5.79E-05
STK32A	0.000107
DPYSL3	0.000141
JAKMIP2	0.000162
SPINK1	0.000177
SCGB3A2	0.000174
C5orf46	0.000174
SPINK5	0.000162
SPINK14	0.000244
SPINK6	0.000162
SPINK13	0.000217
SPINK7	0.000217
SPINK9	0.000162

FBXO38	0.000162
HTR4	0.000177
ADRB2	0.000143
SH3TC2	0.000143
MIR584	0.000129
RN7SKP145	0.000171
ABLIM3	0.00015
AFAP1L1	0.000187
GRPEL2	0.000187
PCYOX1L	0.000187
IL17B	0.000187
MIR143HG	0.000187
MIR143	0.00028
MIR145	0.00028
CSNK1A1	0.000137
ARHGEF37	8.99E-05
U3|ENSG00000221043.1	0.000121
RN7SL868P	0.000121
MIR378A	0.000121
PPARGC1B	8.21E-05
PDE6A	0.000121
SLC26A2	0.000121
TIGD6	0.000121
HMGXB3	0.000121
snoU13|ENSG00000238369.1	0.000121
CSF1R	0.000121
PDGFRB	0.000121
CDX1	0.000121
SLC6A7	0.000121
CAMK2A	0.000121
ARSI	0.000121
TCOF1	0.000121
CD74	0.000121
RPS14	0.000121
NDST1	0.000183
snoU13|ENSG00000239191.1	0.000178
SYNPO	0.000178
MYOZ3	0.000269
RBM22	0.000269
DCTN4	0.000269
SMIM3	0.000269
IRGM	0.000414
ZNF300	0.000414
ZNF300P1	0.000414
GPX3	0.000426
TNIP1	0.000414
ANXA6	0.000414
CCDC69	0.000541
GM2A	0.000541
SLC36A3	0.000207
SLC36A2	0.000207
SLC36A1	0.000321
RNA5SP197	0.000321
FAT2	0.000321
SPARC	0.000478
RN7SKP232	0.000478
ATOX1	0.000478
G3BP1	0.000478

GLRA1	0.000478
RNA5SP198	0.000478
NMUR2	0.000485
GRIA1	0.000323
RN7SL177P	0.000223
FAM114A2	0.000163
MFAP3	0.000163
GALNT10	0.000173
MIR1294	0.00013
RN7SL655P	0.00013
SAP30L	0.00013
HAND1	0.00013
MIR3141	8.48E-05
MIR1303	0.000176
RN7SL439P	0.000176
LARP1	0.000124
FAXDC2	0.000124
MIR378H	0.000176
CNOT8	0.000263
GEMIN5	0.000263
MRPL22	0.000126
KIF4B	0.000137
RNA5SP199	9.44E-05
SGCD	0.000181
TIMD4	0.000391
HAVCR1	0.000312
HAVCR2	0.0003
MED7	0.0003
ITK	0.0003
FAM71B	0.0003
CYFIP2	0.0003
FNDC9	0.0003
ADAM19	0.000312
NIPAL4	0.0003
SOX30	0.000958
C5orf52	0.001367
THG1L	0.001367
LSM11	0.001367
CLINT1	0.001314
EBF1	0.000461
RNF145	0.000501
SNORA68|ENSG00000252458.1	0.000501
UBLCP1	0.000501
IL12B	0.000501
RNU4ATAC2P	0.000501
ADRA1B	0.000249
TTC1	0.000179
PWWP2A	0.000179
FABP6	0.000126
CCNJL	0.000126
RN7SL295P	0.000126
C1QTNF2	0.000126
C5orf54	0.000177
SLU7	0.000177
PTTG1	0.000177
MIR146A	0.000177
MIR3142	0.000177
ATP10B	0.000253

GABRB2	0.000187
GABRA6	0.000187
GABRA1	0.000138
GABRG2	0.000201
CCNG1	8.28E-05
NUDCD2	8.28E-05
HMMR	8.28E-05
MAT2B	0.000111
RN7SKP60	0.000405
TENM2	0.000348
SNORA40|ENSG00000253065.1	0.00044
WWC1	0.000227
RARS	0.000501
FBLL1	0.000501
PANK3	0.000501
MIR103A1	0.000501
SLIT3	0.000481
MIR585	0.000356
SPDL1	0.000673
DOCK2	0.000621
FAM196B	0.000697
MIR378E	0.000673
FOXI1	0.000673
C5orf58	0.000673
LCP2	0.000673
KCNIP1	0.000653
KCNMB1	0.000673
MIR4454	0.000582
GABRP	0.000582
RANBP17	0.000285
RN7SL623P	0.000447
snoU13|ENSG00000252387.1	0.000434
TLX3	0.000363
RN7SL339P	0.000363
SNORA70|ENSG00000206909.1	0.000363
MIR3912	0.000363
NPM1	0.000363
FGF18	0.000363
C5orf50	0.000621
FBXW11	0.000155
EFCAB9	0.000155
STK10	0.000155
UBTD2	0.000155
SNORA57|ENSG00000212529.1	0.000155
SH3PXD2B	0.000151
NEURL1B	0.000146
MIR5003	0.000146
DUSP1	9.21E-05
ERGIC1	9.21E-05
RPL26L1	6.45E-05
ATP6V0E1	7.79E-05
SNORA74B	7.79E-05
CREBRF	7.79E-05
BNIP1	8.05E-05
RNA5SP200	8.05E-05
STC2	8.05E-05
BOD1	8.76E-05
CPEB4	0.000132

C5orf47	0.000132
NSG2	7.52E-05
MIR4634	4.71E-05
MSX2	4.71E-05
DRD1	3.05E-05
snoU13|ENSG00000239026.1	3.05E-05
SFXN1	3.51E-05
RN7SKP148	1.25E-05
HRH2	9.44E-06
CPLX2	8.33E-06
THOC3	5.23E-05
FAM153B	5.03E-05
SIMC1	5.03E-05
KIAA1191	5.03E-05
ARL10	5.03E-05
MIR1271	5.03E-05
NOP16	5.03E-05
HIGD2A	5.03E-05
CLTB	5.03E-05
FAF2	5.03E-05
RNF44	4.89E-05
CDHR2	7.56E-05
RN7SL684P	7.56E-05
GPRIN1	7.56E-05
SNCB	7.56E-05
EIF4E1B	7.56E-05
HK3	7.56E-05
MIR4281	7.56E-05
TSPAN17	7.56E-05
UNC5A	7.56E-05
UIMC1	7.56E-05
ZNF346	3.00E-05
FGFR4	3.00E-05
NSD1	1.38E-05
MXD3	2.10E-05
RAB24	2.10E-05
PRELID1	2.10E-05
LMAN2	2.66E-05
RN7SL562P	2.66E-05
RGS14	2.66E-05
SLC34A1	3.22E-05
F12	3.22E-05
GRK6	3.22E-05
PFN3	3.22E-05
PRR7	3.22E-05
DBN1	3.22E-05
PDLIM7	3.22E-05
DOK3	3.22E-05
DDX41	3.22E-05
FAM193B	3.22E-05
TMED9	3.22E-05
B4GALT7	3.22E-05
FAM153A	2.30E-05
PROP1	2.86E-05
FAM153C	7.02E-05
N4BP3	7.02E-05
RMND5B	7.02E-05
NHP2	7.02E-05

HNRNPAB	7.02E-05
PHYKPL	7.02E-05
COL23A1	7.97E-05
RN7SL646P	2.29E-05
CLK4	3.46E-05
RN7SKP70	3.46E-05
ZNF354A	4.80E-05
AACSP1	4.80E-05
ZNF354B	3.46E-05
ZFP2	2.61E-05
ZNF454	3.46E-05
GRM6	3.46E-05
ZNF879	3.23E-05
ZNF354C	3.23E-05
ADAMTS2	3.37E-05
RN7SL71P	5.04E-05
RUFY1	2.99E-05
HNRNPH1	5.04E-05
C5orf60	5.12E-05
CANX	8.53E-05
CBY3	5.12E-05
HMGB3P22	8.53E-05
MAML1	0.000126
LTC4S	0.000109
MGAT4B	0.000109
MIR1229	0.000109
SQSTM1	0.000109
C5orf45	0.000109
RN7SKP150	0.000109
TBC1D9B	0.000126
RNF130	4.00E-05
MIR340	3.23E-05
RASGEF1C	0.000156
MAPK9	4.46E-05
GFPT2	4.46E-05
CNOT6	5.01E-05
SCGB3A1	5.01E-05
FLT4	2.04E-05
MGAT1	2.04E-05
OR2Y1	2.04E-05
LINC00847	2.04E-05
ZFP62	2.04E-05
BTNL8	1.03E-05
BTNL3	1.09E-05
BTNL9	1.09E-05
GNB2L1	1.09E-05
MIR4638	1.09E-05
OR2V1	1.09E-05
OR2V2	1.09E-05
OR4F3	1.09E-05
SNORD95|ENSG00000264549.1	1.09E-05
SNORD96A	1.09E-05
TRIM41	1.09E-05
TRIM52	1.09E-05
TRIM7	1.09E-05
DUSP22	2.32E-07
EXOC2	2.32E-07
HUS1B	2.32E-07

IRF4	2.32E-07
snoU13|ENSG00000238438.1	3.10E-08
FOXQ1	3.10E-08
FOXF2	5.36E-08
RN7SL352P	8.03E-08
FOXC1	4.67E-08
GMDS	2.68E-08
C6orf195	1.03E-08
MYLK4	9.50E-09
WRNIP1	3.62E-08
SERPINB1	1.07E-08
MIR4645	1.07E-08
SERPINB9	1.07E-08
SERPINB6	1.80E-08
NQO2	1.80E-08
RIPK1	1.05E-08
RNA5SP201	1.05E-08
BPHL	1.05E-08
TUBB2A	1.05E-08
TUBB2B	5.97E-09
PSMG4	5.97E-09
SLC22A23	5.97E-09
PXDC1	1.05E-08
FAM50B	1.05E-08
C6ORF50	4.73E-08
PRPF4B	6.65E-08
FAM217A	6.65E-08
snoU13|ENSG00000252668.1	4.73E-08
C6orf201	6.65E-08
ECI2	6.65E-08
RNA5SP202	5.77E-08
snoU13|ENSG00000238801.1	5.45E-08
CDYL	3.74E-08
RPP40	2.75E-08
RMRPP2	2.75E-08
PPP1R3G	4.79E-08
LYRM4	1.98E-08
MIR3691	4.79E-08
FARS2	7.94E-08
RN7SL221P	1.12E-08
NRN1	1.12E-08
F13A1	2.39E-08
MIR5683	1.36E-08
LY86	3.52E-08
RN7SL554P	9.70E-09
snoU13|ENSG00000251762.1	8.22E-09
RREB1	8.22E-09
SSR1	2.76E-08
CAGE1	2.59E-08
RIOK1	2.59E-08
DSP	6.96E-09
SNRNP48	6.96E-09
BMP6	1.64E-08
TXNDC5	1.64E-08
BLOC1S5	1.64E-08
EEF1E1	1.20E-08
SLC35B3	9.71E-09
HULC	9.71E-09

OFCC1	5.66E-08
RNU6ATAC21P	4.70E-08
TFAP2A	4.70E-08
LINC00518	4.70E-08
MIR5689	4.70E-08
GCNT2	4.70E-08
C6orf52	4.70E-08
GCNT6	4.70E-08
PAK1IP1	4.70E-08
TMEM14C	4.70E-08
SYCP2L	4.70E-08
TMEM14B	4.70E-08
RNA5SP203	4.70E-08
MAK	4.70E-08
GCM2	4.70E-08
ELOVL2	4.70E-08
snoU13|ENSG00000238896.1	5.32E-08
SMIM13	5.32E-08
NEDD9	5.99E-08
TMEM170B	2.74E-08
SNORA67|ENSG00000207419.1	3.65E-08
ADTRP	3.65E-08
HIVEP1	4.67E-08
EDN1	4.04E-08
RN7SKP293	3.09E-08
PHACTR1	2.18E-08
TBC1D7	5.10E-09
GFOD1	9.15E-09
RN7SKP204	9.15E-09
SIRT5	9.15E-09
NOL7	9.15E-09
RANBP9	9.15E-09
MCUR1	9.68E-09
RNF182	5.37E-09
CD83	9.68E-09
RN7SL332P	1.27E-09
JARID2	6.78E-10
DTNBP1	1.14E-09
MYLIP	3.16E-10
MIR4639	3.16E-10
U3|ENSG00000251793.1	3.16E-10
GMPR	3.16E-10
ATXN1	1.48E-10
STMND1	4.59E-09
RBM24	4.59E-09
CAP2	4.59E-09
FAM8A1	6.20E-09
NUP153	6.20E-09
RNA5SP204	1.09E-08
KIF13A	1.09E-08
NHLRC1	4.84E-09
TPMT	4.84E-09
KDM1B	4.84E-09
DEK	4.84E-09
RNF144B	5.00E-09
snoU13|ENSG00000238458.1	3.69E-09
MIR548A1	1.99E-08
RNA5SP205	2.49E-07

ID4	2.64E-07
MBOAT1	1.38E-07
E2F3	4.45E-07
RN7SL128P	4.45E-07
CDKAL1	2.02E-06
SOX4	6.47E-07
LINC00340	6.50E-07
RN7SKP240	2.93E-07
PRL	2.71E-07
HDGFL1	6.12E-07
NRSN1	7.75E-08
SNORD46|ENSG00000251830.1	6.56E-08
DCDC2	6.56E-08
KAAG1	3.85E-08
MRS2	4.97E-08
GPLD1	4.97E-08
ALDH5A1	4.97E-08
KIAA0319	6.56E-08
TDP2	6.56E-08
ACOT13	6.56E-08
C6orf62	6.56E-08
GMNN	4.71E-08
FAM65B	1.09E-07
RN7SL334P	1.09E-07
CMAHP	3.92E-08
RNY5P5	3.92E-08
LRRC16A	1.93E-08
snoU13|ENSG00000238322.1	3.92E-08
HIST1H2AA	3.04E-08
HIST1H2BA	3.04E-08
SCGN	3.04E-08
SLC17A4	3.04E-08
SLC17A1	3.04E-08
SLC17A3	3.04E-08
SLC17A2	3.04E-08
TRIM38	6.61E-08
HIST1H1A	6.61E-08
HIST1H3A	6.61E-08
HIST1H4A	6.61E-08
HIST1H4B	6.61E-08
HIST1H2AB	6.61E-08
HIST1H3B	6.61E-08
HIST1H1C	6.61E-08
HIST1H2BB	6.61E-08
HIST1H3C	6.61E-08
HFE	6.61E-08
HIST1H4C	6.61E-08
HIST1H1T	6.61E-08
HIST1H2BC	6.61E-08
HIST1H2AC	6.61E-08
HIST1H1E	6.61E-08
HIST1H2BD	2.21E-07
HIST1H2BE	2.21E-07
HIST1H4D	2.21E-07
HIST1H3D	2.21E-07
HIST1H2AD	2.21E-07
HIST1H2BF	2.21E-07
HIST1H4E	2.21E-07

HIST1H2BG	3.01E-07
HIST1H2AE	3.01E-07
HIST1H3E	3.01E-07
HIST1H1D	3.01E-07
HIST1H4F	3.01E-07
HIST1H4G	3.01E-07
HIST1H3F	3.01E-07
HIST1H2BH	3.01E-07
HIST1H2BI	3.01E-07
HIST1H3G	3.01E-07
HIST1H4H	3.01E-07
BTN3A2	3.38E-07
BTN2A2	3.38E-07
BTN3A1	3.38E-07
BTN2A1	3.38E-07
BTN2A3P	3.38E-07
BTN3A3	3.38E-07
BTN1A1	3.38E-07
HCG11	3.38E-07
HMGN4	3.38E-07
ABT1	3.38E-07
ZNF322	3.38E-07
GUSBP2	2.47E-07
LINC00240	1.99E-07
TRNAI2	2.47E-07
HIST1H2BJ	2.47E-07
HIST1H2AG	4.09E-07
HIST1H2BK	4.09E-07
HIST1H4I	4.09E-07
HIST1H2AH	4.09E-07
MIR3143	4.09E-07
PRSS16	4.09E-07
POM121L2	7.38E-08
ZNF391	7.38E-08
ZNF184	1.21E-07
TRNAI6	1.35E-06
HIST1H2AI	2.13E-06
HIST1H2AJ	2.13E-06
HIST1H2BL	2.13E-06
HIST1H2BM	2.13E-06
HIST1H3H	2.13E-06
HIST1H4J	1.60E-06
HIST1H4K	1.60E-06
HIST1H2AK	1.60E-06
HIST1H2BN	1.60E-06
HIST1H2AL	1.60E-06
HIST1H1B	1.60E-06
HIST1H3I	1.60E-06
HIST1H4L	1.60E-06
HIST1H3J	1.60E-06
HIST1H2AM	1.60E-06
HIST1H2BO	1.60E-06
OR2B2	9.97E-07
OR2B6	9.97E-07
U3|ENSG00000199851.1	9.97E-07
ZNF165	2.17E-06
ZSCAN12P1	2.17E-06
ZSCAN16	2.17E-06

ZKSCAN8	2.17E-06
ZNF192P1	2.17E-06
ZSCAN9	2.17E-06
ZKSCAN4	2.17E-06
NKAPL	2.17E-06
PGBD1	2.17E-06
ZSCAN31	1.36E-06
ZKSCAN3	1.36E-06
ZSCAN12	1.81E-06
ZSCAN23	3.40E-06
GPX6	3.40E-06
GPX5	3.40E-06
SCAND3	4.06E-06
LINC00533	4.06E-06
HCG14	8.34E-06
TRIM27	8.34E-06
C6orf100	7.57E-06
RN7SL471P	7.57E-06
HCG15	6.25E-06
HCG16	6.25E-06
ZNF311	6.25E-06
OR2W1	6.25E-06
OR2B3	6.25E-06
OR2J1	6.25E-06
OR2J2	6.25E-06
OR2J3	6.25E-06
OR14J1	6.09E-06
OR5V1	5.46E-06
OR12D3	5.46E-06
OR12D2	5.46E-06
OR12D1P	5.46E-06
OR11A1	5.46E-06
OR10C1	5.46E-06
OR2H1	5.46E-06
MAS1L	5.46E-06
GABBR1	9.57E-06
UBD	9.57E-06
OR2H2	9.57E-06
SNORD32B	9.57E-06
MOG	1.03E-05
ZFP57	1.03E-05
HCG9	2.69E-06
ZNRD1	2.69E-06
PPP1R11	2.69E-06
RNF39	2.69E-06
TRIM31	2.69E-06
SNORA48|ENSG00000252228.1	2.69E-06
TRIM40	2.69E-06
TRIM10	2.69E-06
TRIM15	2.69E-06
TRIM26	2.69E-06
HCG17	3.24E-06
HCG18	3.14E-06
RPP21	3.14E-06
TRIM39	3.14E-06
GNL1	2.19E-06
PRR3	2.19E-06
ABCF1	2.19E-06

MIR877	2.19E-06
PPP1R10	2.19E-06
MRPS18B	2.19E-06
ATAT1	2.19E-06
C6orf136	2.19E-06
DHX16	2.19E-06
NRM	2.19E-06
PPP1R18	2.19E-06
MDC1	2.19E-06
TUBB	2.19E-06
FLOT1	1.50E-06
IER3	2.40E-06
RN7SL353P	2.40E-06
HCG20	2.40E-06
LINC00243	3.62E-06
DDR1	3.62E-06
RN7SKP186	3.62E-06
MIR4640	3.62E-06
GTF2H4	3.62E-06
RN7SL175P	3.62E-06
VARS2	3.62E-06
SFTA2	3.62E-06
DPCR1	3.62E-06
HCG21	3.62E-06
MUC21	3.62E-06
MUC22	3.62E-06
HCG22	3.93E-06
C6orf15	3.82E-06
CDSN	3.82E-06
PSORS1C1	7.95E-06
PSORS1C2	7.74E-06
CCHCR1	1.60E-05
TCF19	1.60E-05
POU5F1	5.98E-06
PSORS1C3	5.98E-06
HCG27	5.98E-06
ABHD16A	1.68E-06
AGER	1.68E-06
AGPAT1	1.68E-06
AIF1	1.68E-06
APOM	1.68E-06
ATF6B	1.68E-06
ATP6V1G2	1.68E-06
BAG6	1.68E-06
BTNL2	1.68E-06
C2	1.68E-06
C4A	1.68E-06
C4B	1.68E-06
C6orf10	1.68E-06
C6orf25	1.68E-06
C6orf47	1.68E-06
C6orf48	1.68E-06
CFB	1.68E-06
CLIC1	1.68E-06
CSNK2B	1.68E-06
CYP21A1P	1.68E-06
CYP21A2	1.68E-06
DDAH2	1.68E-06

DDX39B	1.68E-06
DOM3Z	1.68E-06
EGFL8	1.68E-06
EHMT2	1.68E-06
FKBPL	1.68E-06
GPANK1	1.68E-06
GPSM3	1.68E-06
HCG23	1.68E-06
HCP5	1.68E-06
HSPA1A	1.68E-06
HSPA1B	1.68E-06
HSPA1L	1.68E-06
LSM2	1.68E-06
LST1	1.68E-06
LTA	1.68E-06
LTB	1.68E-06
LY6G5B	1.68E-06
LY6G5C	1.68E-06
LY6G6C	1.68E-06
LY6G6D	1.68E-06
LY6G6E	1.68E-06
LY6G6F	1.68E-06
MCCD1	1.68E-06
MEGT1	1.68E-06
MICA	1.68E-06
MICB	1.68E-06
MIR1236	1.68E-06
MIR3135B	1.68E-06
MIR4646	1.68E-06
MSH5	1.68E-06
NCR3	1.68E-06
NELFE	1.68E-06
NEU1	1.68E-06
NFKBIL1	1.68E-06
NOTCH4	1.68E-06
PBX2	1.68E-06
PPP1R2P1	1.68E-06
PPT2	1.68E-06
PRRC2A	1.68E-06
PRRT1	1.68E-06
PSMB8	1.68E-06
PSMB9	1.68E-06
RNA5SP206	1.68E-06
RNF5	1.68E-06
SAPCD1	1.68E-06
SKIV2L	1.68E-06
SLC44A4	1.68E-06
SNORA38|ENSG00000200816.1	1.68E-06
SNORD117	1.68E-06
SNORD48	1.68E-06
SNORD52	1.68E-06
SNORD84	1.68E-06
STK19	1.68E-06
TAP1	1.68E-06
TAP2	1.68E-06
TNF	1.68E-06
TNXB	1.68E-06
VARS	1.68E-06

VWA7	1.68E-06
ZBTB12	1.68E-06
BRD2	2.08E-06
HCG24	1.57E-06
COL11A2	1.57E-06
RXRB	1.57E-06
HSD17B8	1.57E-06
RNY4P10	1.57E-06
SLC39A7	1.57E-06
RING1	1.57E-06
HCG25	1.12E-06
VPS52	1.12E-06
RPS18	1.12E-06
B3GALT4	1.12E-06
WDR46	1.12E-06
PFDN6	1.12E-06
RGL2	1.12E-06
TAPBP	1.12E-06
DAXX	1.43E-06
ZBTB22	1.43E-06
KIFC1	1.43E-06
CUTA	1.89E-06
PHF1	1.89E-06
SYNGAP1	9.48E-07
MIR5004	9.48E-07
ZBTB9	1.14E-06
RN7SL26P	1.77E-06
BAK1	1.14E-06
GGNBP1	1.14E-06
LINC00336	1.14E-06
ITPR3	8.23E-07
MNF1	1.14E-06
SBP1	1.14E-06
MIR3934	1.14E-06
IP6K3	1.14E-06
LEMD2	9.48E-07
MLN	9.48E-07
MIR1275	9.93E-07
GRM4	1.90E-06
HMGA1	1.90E-06
C6orf1	1.90E-06
NUDT3	2.54E-06
RPS10	2.54E-06
PACSIN1	2.54E-06
SPDEF	1.88E-06
C6orf106	1.08E-06
RN7SL200P	1.08E-06
snoU13|ENSG00000239059.1	1.08E-06
snoU13|ENSG00000238484.1	1.08E-06
ANKS1A	1.24E-06
SNRPC	2.02E-06
TAF11	2.02E-06
UHRF1BP1	2.02E-06
TCP11	1.07E-06
SCUBE3	8.14E-07
ZNF76	1.54E-06
DEF6	1.54E-06
PPARD	1.54E-06

FANCE	2.02E-06
RPL10A	2.02E-06
TEAD3	2.02E-06
TULP1	4.53E-06
FKBP5	5.68E-06
SNORA40|ENSG00000212579.1	2.02E-06
MIR5690	2.02E-06
ARMC12	2.51E-06
CLPSL1	2.51E-06
CLPSL2	2.51E-06
CLPS	2.51E-06
LHFPL5	2.51E-06
SRPK1	3.19E-06
SLC26A8	3.19E-06
MAPK14	3.19E-06
MAPK13	2.51E-06
BRPF3	2.51E-06
PNPLA1	2.51E-06
C6orf222	2.76E-06
ETV7	2.76E-06
PXT1	2.76E-06
KCTD20	2.76E-06
RN7SL502P	2.76E-06
STK38	2.76E-06
RN7SL748P	2.76E-06
SRSF3	2.76E-06
MIR3925	2.76E-06
CDKN1A	2.76E-06
RAB44	2.76E-06
CPNE5	2.76E-06
PPIL1	2.76E-06
C6orf89	2.25E-06
PI16	2.25E-06
MTCH1	3.83E-06
FGD2	3.68E-06
COX6A1P2	3.83E-06
PIM1	7.81E-06
SNORD112|ENSG00000252687.1	7.81E-06
TMEM217	7.81E-06
snoU13|ENSG00000238375.1	7.81E-06
TBC1D22B	7.81E-06
RN7SL273P	7.81E-06
RNF8	7.81E-06
FTSJD2	7.50E-06
CCDC167	6.67E-06
MIR4462	8.15E-06
MDGA1	7.50E-06
ZFAND3	7.95E-06
RN7SL285P	8.60E-06
BTBD9	1.52E-06
SNORD45|ENSG00000200706.1	1.29E-06
GLO1	3.33E-06
DNAH8	1.33E-06
RN7SL465P	3.22E-06
SNORA8|ENSG00000212586.1	3.22E-06
GLP1R	2.84E-06
SAYSD1	6.08E-06
KCNK5	2.42E-06

KCNK17	2.42E-06
KCNK16	2.42E-06
KIF6	2.56E-06
DAAM2	1.07E-06
MOCS1	1.94E-06
TDRG1	5.63E-06
LINC00951	5.63E-06
LRFN2	1.93E-06
OARD1	3.33E-06
UNC5CL	3.33E-06
TSPO2	3.33E-06
APOBEC2	3.33E-06
NFYA	1.99E-06
ADCY10P1	1.99E-06
TREML1	1.99E-06
TREM2	1.99E-06
TREML2	1.99E-06
TREML3P	1.99E-06
TREML4	1.99E-06
RNA5SP207	1.99E-06
TREM1	1.99E-06
NCR2	3.85E-06
FOXP4	3.85E-06
MIR4641	3.85E-06
MDFI	3.85E-06
TFEB	6.22E-06
PGC	6.47E-06
FRS3	6.47E-06
PRICKLE4	6.47E-06
TOMM6	6.47E-06
USP49	3.88E-06
SNORA8|ENSG00000206977.1	6.22E-06
MED20	3.88E-06
BYSL	3.88E-06
CCND3	3.88E-06
TAF8	3.88E-06
C6orf132	6.06E-06
GUCA1A	5.20E-06
GUCA1B	4.26E-06
MRPS10	4.26E-06
TRERF1	4.26E-06
U3|ENSG00000221252.1	4.26E-06
snoU13|ENSG00000238611.1	4.26E-06
UBR2	4.26E-06
PRPH2	4.26E-06
TBCC	2.90E-06
GLTSCR1L	2.90E-06
RPL7L1	2.90E-06
C6orf226	2.90E-06
PTCRA	4.57E-06
CNPY3	4.57E-06
GNMT	4.57E-06
PEX6	4.57E-06
PPP2R5D	4.57E-06
MEA1	4.57E-06
KLHDC3	4.57E-06
RRP36	4.57E-06
RN7SL403P	4.57E-06

CUL7	4.57E-06
KLC4	2.38E-05
MRPL2	7.50E-06
PTK7	1.18E-05
SRF	7.50E-06
CUL9	9.80E-06
DNPH1	9.80E-06
TTBK1	9.80E-06
SLC22A7	9.80E-06
CRIP3	9.80E-06
ZNF318	8.12E-06
ABCC10	8.12E-06
DLK2	8.12E-06
TJAP1	9.57E-06
LRRC73	9.57E-06
POLR1C	9.57E-06
YIPF3	9.57E-06
XPO5	9.57E-06
SCARNA15|ENSG00000252218.1	9.57E-06
POLH	9.57E-06
GTPBP2	9.57E-06
MAD2L1BP	9.57E-06
RSPH9	9.57E-06
MRPS18A	5.27E-06
VEGFA	5.27E-06
C6orf223	7.77E-07
MRPL14	7.77E-07
TMEM63B	7.77E-07
CAPN11	7.77E-07
SLC29A1	1.24E-06
HSP90AB1	1.24E-06
MIR4647	1.24E-06
SLC35B2	1.24E-06
NFKBIE	1.24E-06
TMEM151B	1.38E-06
TCTE1	1.49E-06
AARS2	2.86E-06
SPATS1	4.30E-06
CDC5L	5.57E-06
MIR4642	8.84E-06
SUPT3H	7.52E-06
MIR586	5.88E-06
RUNX2	7.09E-06
CLIC5	1.13E-05
ENPP4	9.40E-06
ENPP5	9.40E-06
RCAN2	8.17E-06
CYP39A1	2.53E-05
SLC25A27	2.50E-05
TDRD6	2.50E-05
PLA2G7	2.50E-05
ANKRD66	2.50E-05
MEP1A	2.97E-05
GPR116	1.56E-05
GPR110	3.48E-06
TNFRSF21	2.37E-06
CD2AP	6.12E-06
GPR111	6.40E-06

GPR115	2.59E-06
RN7SKP116	2.59E-06
OPN5	2.59E-06
PTCHD4	2.59E-06
MUT	8.86E-07
CENPQ	8.86E-07
GLYATL3	8.86E-07
C6orf141	1.33E-06
RHAG	1.01E-06
CRISP2	8.32E-07
CRISP3	8.32E-07
PGK2	8.32E-07
CRISP1	5.91E-07
DEFB133	9.31E-07
DEFB114	9.31E-07
DEFB113	9.31E-07
DEFB110	1.09E-06
DEFB112	1.09E-06
TFAP2D	1.24E-05
TFAP2B	1.30E-05
SNORD66|ENSG00000212532.1	1.15E-06
PKHD1	6.75E-07
RN7SL580P	1.31E-06
MIR206	1.18E-06
MIR133B	1.18E-06
IL17A	8.31E-07
IL17F	8.31E-07
MCM3	8.31E-07
PAQR8	1.23E-05
EFHC1	1.23E-05
TRAM2	1.23E-05
TMEM14A	1.04E-05
GSTA1	7.80E-06
GSTA2	5.95E-06
GSTA5	7.80E-06
GSTA3	8.31E-06
GSTA4	2.36E-06
RN7SK	2.36E-06
ICK	1.49E-06
FBXO9	1.49E-06
RN7SL244P	1.49E-06
GCM1	3.59E-06
U3|ENSG00000251930.1	3.59E-06
ELOVL5	3.13E-06
MIR5685	2.57E-06
RN7SKP256	2.57E-06
GCLC	9.95E-07
KLHL31	1.29E-06
LRRC1	1.00E-06
MLIP	6.67E-07
TINAG	2.14E-06
FAM83B	1.98E-06
HCRTR2	5.08E-06
GFRAL	4.54E-06
HMGCLL1	4.66E-06
BMP5	1.40E-05
COL21A1	3.83E-06
DST	2.18E-06

BEND6	2.61E-06
KIAA1586	2.45E-06
ZNF451	1.55E-06
BAG2	1.55E-06
RAB23	1.55E-06
PRIM2	1.11E-06
LINC00680	3.18E-06
MIR548U	3.18E-06
MTRNR2L9	1.97E-06
KHDRBS2	7.94E-08
FKBP1C	3.99E-07
LGSN	2.34E-07
PTP4A1	1.96E-07
PHF3	3.06E-07
EYS	1.49E-06
SNORD65|ENSG00000212229.1	1.44E-07
RNA5SP208	5.10E-07
BAI3	2.05E-08
LMBRD1	9.37E-07
COL19A1	2.05E-07
COL9A1	3.00E-07
FAM135A	2.11E-07
C6orf57	2.36E-07
SMAP1	1.16E-07
B3GAT2	1.58E-07
U3|ENSG00000221345.1	1.15E-07
OGFRL1	1.87E-07
LINC00472	2.87E-07
MIR30C2	2.87E-07
MIR30A	2.87E-07
RIMS1	2.93E-08
KCNQ5	7.81E-08
MIR4282	6.82E-07
KHDC1L	4.29E-07
KHDC1	4.29E-07
DPPA5	2.72E-07
KHDC3L	2.72E-07
OOEP	2.72E-07
DDX43	2.72E-07
snoU13|ENSG00000238464.1	2.72E-07
MB21D1	4.44E-07
MTO1	4.44E-07
EEF1A1	4.44E-07
SLC17A5	4.44E-07
CD109	3.60E-06
COL12A1	2.45E-07
COX7A2	4.08E-07
TMEM30A	4.08E-07
FILIP1	1.53E-07
U3|ENSG00000221332.1	1.53E-07
MIR4463	2.55E-07
SENP6	9.03E-08
RN7SKP163	3.38E-08
MYO6	2.57E-07
RNA5SP209	6.79E-07
snoU13|ENSG00000239132.1	6.79E-07
IMPG1	1.17E-06
U6|ENSG00000272445.1	2.87E-06

HTR1B	5.16E-07
SNORD112|ENSG00000252932.1	1.41E-06
IRAK1BP1	1.80E-07
PHIP	4.41E-07
HMGN3	7.07E-07
LCA5	5.04E-07
SH3BGRL2	3.42E-07
ELOVL4	1.77E-07
TTK	2.91E-07
BCKDHB	2.91E-07
FAM46A	1.59E-07
RNA5SP210	1.59E-07
SNORA70|ENSG00000206886.1	1.59E-07
IBTK	1.19E-07
TPBG	1.19E-07
UBE3D	1.69E-07
DOPEY1	2.35E-07
PGM3	2.35E-07
RWDD2A	2.35E-07
ME1	1.87E-07
PRSS35	3.14E-07
SNAP91	3.14E-07
RIPPLY2	4.94E-07
CYB5R4	4.94E-07
MRAP2	4.57E-07
KIAA1009	4.57E-07
TBX18	4.50E-07
NT5E	7.84E-07
SNX14	7.84E-07
SYNCRIP	7.84E-07
SNHG5	7.15E-07
RN7SL643P	1.29E-06
HTR1E	1.02E-06
RN7SKP209	1.72E-06
CGA	1.72E-06
ZNF292	3.07E-06
GJB7	3.07E-06
SMIM8	3.07E-06
C6orf163	1.65E-06
C6orf164	1.65E-06
C6ORF165	1.65E-06
C6orf165	1.65E-06
SLC35A1	1.65E-06
RARS2	1.65E-06
ORC3	1.65E-06
AKIRIN2	2.59E-06
snoU13|ENSG00000238628.1	2.59E-06
RN7SL183P	3.90E-06
SPACA1	2.94E-06
CNR1	2.94E-06
RNGTT	4.21E-06
SNORA73|ENSG00000222145.1	5.61E-06
PNRC1	1.58E-06
RN7SL336P	1.58E-06
SRSF12	1.58E-06
PM20D2	1.58E-06
GABRR1	1.58E-06
GABRR2	1.58E-06

UBE2J1	1.58E-06
RRAGD	1.58E-06
ANKRD6	8.92E-07
RN7SL11P	9.43E-07
LYRM2	8.92E-07
MDN1	8.92E-07
CASP8AP2	9.68E-07
snoU13|ENSG00000238747.1	9.68E-07
GJA10	9.68E-07
BACH2	6.40E-07
RN7SKP110	6.35E-07
MIR4464	5.65E-07
MAP3K7	1.64E-06
MIR4643	2.52E-06
RN7SL415P	4.13E-06
U3|ENSG00000200492.1	2.71E-06
U3|ENSG00000221455.1	3.93E-06
EPHA7	5.62E-06
SNORA18|ENSG00000252249.1	5.68E-06
MANEA	2.43E-07
FUT9	1.12E-06
RN7SL797P	3.13E-06
UFL1	3.06E-06
FHL5	3.06E-06
GPR63	4.72E-06
NDUFAF4	4.72E-06
RN7SL509P	4.72E-06
KLHL32	4.72E-06
MMS22L	1.04E-06
MIR2113	6.09E-06
POU3F2	5.82E-06
FBXL4	5.82E-06
MIR548AI	3.22E-06
FAXC	2.17E-06
COQ3	3.22E-06
PNISR	3.22E-06
USP45	3.22E-06
TSTD3	2.34E-06
CCNC	2.34E-06
PRDM13	3.53E-06
MCHR2	3.53E-06
SIM1	9.18E-06
ASCC3	2.94E-05
snoU13|ENSG00000238999.1	2.73E-05
GRIK2	1.15E-05
SNORA33|ENSG00000202283.1	1.09E-05
HACE1	1.23E-05
LINC00577	1.23E-05
LIN28B	1.23E-05
BVES	1.23E-05
POPDC3	1.23E-05
PREP	1.18E-05
RN7SKP211	1.14E-05
PRDM1	1.14E-05
ATG5	1.14E-05
RN7SL47P	1.14E-05
RNA5SP211	1.14E-05
AIM1	3.85E-05

RTN4IP1	3.85E-05
QRSL1	1.33E-05
MIR587	1.33E-05
C6orf203	2.27E-05
BEND3	2.27E-05
PDSS2	2.27E-05
SOBP	2.27E-05
SCML4	6.00E-05
SEC63	2.46E-05
SNORA73|ENSG00000253090.1	2.71E-05
OSTM1	2.71E-05
NR2E1	2.71E-05
SNX3	2.71E-05
snoU13|ENSG00000238974.1	4.07E-05
RNA5SP212	4.07E-05
LACE1	2.05E-05
FOXO3	3.62E-05
LINC00222	3.62E-05
ZNF259P1	3.62E-05
ARMC2	2.46E-05
SESN1	2.46E-05
CEP57L1	2.16E-05
C6orf183	2.16E-05
CCDC162P	2.91E-05
snoU13|ENSG00000238474.1	2.91E-05
CD164	1.30E-05
PPIL6	1.30E-05
SMPD2	1.96E-05
MICAL1	1.96E-05
ZBTB24	1.30E-05
AK9	1.35E-05
FIG4	7.68E-06
GPR6	4.39E-06
WASF1	3.42E-06
CDC40	1.47E-06
METTL24	1.18E-05
DDO	1.18E-05
SLC22A16	3.67E-05
RN7SL617P	3.67E-05
CDK19	7.08E-05
SNORA40|ENSG00000212587.1	7.08E-05
AMD1	7.08E-05
GTF3C6	4.96E-05
snoU13|ENSG00000238775.1	4.96E-05
RPF2	4.96E-05
SLC16A10	4.00E-05
KIAA1919	4.00E-05
REV3L	4.00E-05
TRAF3IP2	3.12E-05
C6orf3	4.96E-05
snoU13|ENSG00000239015.1	2.57E-05
FYN	2.57E-05
WISP3	4.96E-05
TUBE1	4.96E-05
FAM229B	4.96E-05
LAMA4	4.96E-05
RFPL4B	2.78E-05
snoU13|ENSG00000239095.1	1.17E-05

U3|ENSG00000253091.1	2.59E-05
snoU13|ENSG00000238563.1	4.01E-05
MARCKS	4.01E-05
HDAC2	4.30E-05
HS3ST5	0.000153
RNA5SP213	4.30E-05
FRK	6.12E-05
NT5DC1	6.12E-05
COL10A1	6.12E-05
TSPYL4	6.12E-05
DSE	3.59E-05
TSPYL1	6.12E-05
FAM26F	3.59E-05
TRAPPC3L	3.24E-05
FAM26E	3.24E-05
FAM26D	3.24E-05
RWDD1	3.24E-05
RSPH4A	6.12E-05
ZUFSP	6.12E-05
KPNA5	6.12E-05
FAM162B	4.24E-05
GPRC6A	4.40E-05
RFX6	4.24E-05
RNA5SP214	4.24E-05
VGLL2	7.78E-05
ROS1	3.53E-05
RN7SKP18	7.78E-05
RN7SKP51	7.78E-05
GOPC	5.82E-05
DCBLD1	5.40E-05
NUS1	1.88E-05
SLC35F1	1.38E-05
CEP85L	1.38E-05
MCM9	1.38E-05
PLN	1.38E-05
ASF1A	1.38E-05
FAM184A	4.87E-05
MIR548B	2.17E-05
MAN1A1	5.80E-05
MIR3144	7.35E-05
RNA5SP215	3.88E-05
TBC1D32	3.99E-05
SIGLECP3	4.25E-05
GJA1	4.25E-05
HSF2	3.99E-05
SERINC1	3.99E-05
PKIB	5.02E-05
RN7SL564P	2.84E-05
FABP7	2.84E-05
SMPDL3A	2.84E-05
CLVS2	9.69E-06
TRDN	5.50E-06
NKAIN2	9.81E-06
RNF217	4.60E-06
TPD52L1	7.35E-06
HDDC2	3.05E-06
HEY2	1.95E-06
NCOA7	2.42E-06

RN7SKP56	3.46E-06
HINT3	1.70E-05
RNA5SP216	1.07E-05
TRMT11	4.70E-06
CENPW	9.94E-06
MIR588	1.61E-05
PRELID1P1	1.61E-05
RSPO3	1.34E-05
RNF146	5.09E-06
ECHDC1	3.34E-06
RNA5SP217	4.96E-06
SOGA3	3.34E-06
KIAA0408	3.34E-06
C6orf58	3.34E-06
THEMIS	6.06E-06
PTPRK	2.19E-06
snoU13|ENSG00000238938.1	8.83E-06
LAMA2	3.50E-05
ARHGAP18	1.76E-05
TMEM244	7.99E-06
L3MBTL3	7.99E-06
SAMD3	7.99E-06
TMEM200A	1.46E-05
SMLR1	1.46E-05
EPB41L2	1.46E-05
AKAP7	1.37E-05
ARG1	1.24E-05
MED23	1.24E-05
ENPP3	2.59E-05
OR2A4	2.59E-05
CTAGE9	2.59E-05
MIR548H5	1.37E-05
ENPP1	1.37E-05
RN7SKP245	1.37E-05
CTGF	2.28E-05
MIR548AJ1	9.06E-06
MOXD1	8.26E-06
STX7	8.26E-06
TAAR9	8.26E-06
TAAR8	8.26E-06
TAAR6	8.26E-06
TAAR5	7.27E-06
TAAR3	1.13E-05
TAAR2	1.13E-05
TAAR1	1.13E-05
VNN1	1.13E-05
VNN3	1.13E-05
VNN2	1.13E-05
SLC18B1	1.13E-05
RPS12	1.13E-05
SNORA33|ENSG00000200534.1	1.13E-05
SNORD100	1.13E-05
SNORD101	1.13E-05
LINC00326	3.41E-05
EYA4	4.00E-05
TCF21	4.68E-05
TBPL1	4.68E-05
SLC2A12	4.68E-05

RN7SL408P	4.68E-05
SGK1	4.68E-05
snoU13|ENSG00000238631.1	5.42E-05
RNA5SP218	7.13E-05
ALDH8A1	6.92E-05
HBS1L	4.48E-05
MYB	1.03E-05
MIR548A2	1.26E-05
AHI1	1.93E-05
LINC00271	6.78E-05
PDE7B	0.000132
COX5BP2	0.000207
MTFR2	8.44E-05
BCLAF1	8.44E-05
MAP7	5.55E-05
7SK|ENSG00000271765.1	8.67E-05
MAP3K5	8.78E-05
RNA5SP219	8.67E-05
PEX7	8.78E-05
SNORA27|ENSG00000201807.1	8.78E-05
SLC35D3	7.46E-05
IL20RA	0.000112
IL22RA2	7.46E-05
IFNGR1	7.46E-05
OLIG3	0.000129
SNORD112|ENSG00000252476.1	4.61E-05
TNFAIP3	7.46E-05
PERP	6.98E-05
KIAA1244	7.46E-05
PBOV1	7.46E-05
HEBP2	7.46E-05
NHSL1	4.92E-05
MIR3145	7.46E-05
CCDC28A	4.08E-05
ECT2L	4.08E-05
REPS1	6.13E-05
ABRACL	6.13E-05
HECA	7.17E-05
TXLNB	7.17E-05
CITED2	7.17E-05
RNA5SP220	5.48E-05
MIR3668	5.19E-05
MIR4465	2.03E-05
RN7SKP106	4.38E-05
NMBR	3.81E-05
GJE1	3.81E-05
VTA1	3.81E-05
GPR126	3.81E-05
HIVEP2	5.74E-05
AIG1	3.67E-05
ADAT2	3.81E-05
PEX3	3.81E-05
RNA5SP221	3.81E-05
FUCA2	3.81E-05
PHACTR2	3.18E-05
LTV1	3.18E-05
ZC2HC1B	3.18E-05
PLAGL1	2.93E-05

SF3B5	2.93E-05
STX11	2.49E-05
UTRN	0.0001
EPM2A	5.24E-05
FBXO30	9.25E-05
SHPRH	9.25E-05
GRM1	9.25E-05
RNA5SP222	9.25E-05
RAB32	5.24E-05
ADGB	0.000148
STXBP5	5.74E-05
SAMD5	4.22E-05
SASH1	2.95E-05
UST	7.42E-05
TAB2	4.66E-05
RN7SL234P	4.66E-05
SUMO4	4.66E-05
ZC3H12D	4.66E-05
PPIL4	4.66E-05
GINM1	4.66E-05
SNORA2|ENSG00000202343.1	4.66E-05
KATNA1	4.66E-05
LATS1	4.66E-05
NUP43	4.66E-05
PCMT1	4.66E-05
LRP11	4.66E-05
RAET1E	4.66E-05
RAET1G	4.66E-05
ULBP2	4.66E-05
ULBP1	4.82E-05
RAET1K	4.82E-05
RAET1L	6.79E-05
ULBP3	6.79E-05
PPP1R14C	9.32E-05
snoU13|ENSG00000238594.1	9.32E-05
IYD	0.000149
PLEKHG1	8.05E-05
MTHFD1L	5.58E-05
AKAP12	4.95E-05
RNY4P20	4.95E-05
RN7SKP268	4.95E-05
snoU13|ENSG00000238939.1	4.95E-05
ZBTB2	4.95E-05
RMND1	4.95E-05
C6orf211	4.95E-05
CCDC170	6.64E-05
ESR1	9.44E-05
SYNE1	3.73E-05
RNA5SP223	7.97E-05
MYCT1	0.000118
VIP	0.000118
FBXO5	9.18E-05
MTRF1L	9.18E-05
RGS17	9.18E-05
RNA5SP224	6.12E-05
RNA5SP225	9.18E-05
OPRM1	5.61E-05
IPCEF1	4.91E-05

CNKSR3	4.21E-05
SCAF8	4.73E-05
TIAM2	5.89E-05
MIR1273C	4.73E-05
U8|ENSG00000238963.1	4.73E-05
TFB1M	5.89E-05
CLDN20	5.89E-05
NOX3	5.42E-05
MIR1202	0.000112
SNORD28|ENSG00000212295.1	9.05E-05
ARID1B	2.11E-05
MIR4466	6.10E-05
TMEM242	0.0001
snoU13|ENSG00000252609.1	0.0001
ZDHHC14	0.000105
MIR3692	0.000118
SNX9	0.000113
SYNJ2	0.000113
SERAC1	0.000113
GTF2H5	0.000113
TULP4	0.000143
RN7SL173P	0.000113
TMEM181	0.000143
DYNLT1	0.000143
SYTL3	0.000143
EZR	9.55E-05
MIR3918	0.000143
OSTCP1	5.00E-05
C6orf99	3.72E-05
RSPH3	4.05E-05
TAGAP	4.05E-05
FNDC1	3.08E-05
SOD2	1.27E-05
RNU4ATAC18P	2.32E-05
WTAP	1.27E-05
ACAT2	1.05E-05
TCP1	1.05E-05
SNORA20|ENSG00000207392.1	1.05E-05
SNORA29	1.05E-05
MRPL18	1.05E-05
PNLDC1	1.05E-05
MAS1	1.05E-05
IGF2R	1.27E-05
AIRN	1.05E-05
SLC22A1	1.24E-05
SLC22A2	1.24E-05
SLC22A3	1.27E-05
LPAL2	8.73E-06
LPA	8.73E-06
PLG	8.73E-06
MAP3K4	1.24E-05
AGPAT4	1.26E-05
PARK2	7.07E-06
PACRG	1.80E-05
snoU13|ENSG00000239136.1	2.20E-05
CAHM	4.10E-05
QKI	9.49E-05
RN7SL366P	0.000121

C6orf118	5.62E-05
PDE10A	5.29E-05
RNA5SP226	3.80E-05
SDIM1	3.18E-05
LINC00473	3.18E-05
SNORD45|ENSG00000231297.2	4.75E-05
T	2.82E-05
PRR18	4.75E-05
SFT2D1	4.75E-05
MPC1	4.75E-05
RPS6KA2	3.70E-05
MIR1913	5.49E-05
RNASET2	3.70E-05
FGFR1OP	3.48E-05
MIR3939	3.48E-05
CCR6	3.48E-05
TCP10L2	3.48E-05
C6orf123	3.48E-05
GPR31	3.48E-05
MLLT4	2.05E-05
TCP10	3.48E-05
TTLL2	3.48E-05
UNC93A	3.48E-05
DACT2	1.96E-05
FRMD1	1.96E-05
KIF25	1.96E-05
SMOC2	2.86E-05
THBS2	1.73E-05
WDR27	1.73E-05
C6orf120	1.73E-05
PHF10	1.70E-05
TCTE3	2.65E-05
C6orf70	2.67E-05
LINC00242	2.59E-05
LINC00574	2.59E-05
DLL1	2.59E-05
FAM120B	2.25E-05
MIR4644	2.25E-05
PSMB1	2.43E-05
TBP	2.43E-05
PDCD2	1.65E-05
DDC	0.040494
GRB10	0.036631
POM121L12	0.032447
snoU13|ENSG00000238354.1	0.012265
VSTM2A	0.010638
SEC61G	0.013619
SNORA73|ENSG00000252054.1	0.019622
EGFR	0.024689
LANCL2	0.021537
VOPP1	0.015539
FKBP9L	0.015287
14-Sep	0.015287
MRPS17	0.013703
ZNF713	0.013703
RN7SL64P	0.013703
GBAS	0.014501
PSPH	0.011439

CCT6A	0.011439
SNORA15|ENSG00000207168.1	0.011439
SNORA22|ENSG00000206603.1	0.011439
SUMF2	0.011439
PHKG1	0.011439
CHCHD2	0.011439
snoU13|ENSG00000238673.1	0.011439
NUPR1L	0.016094
RN7SL816P	0.022184
ZNF479	0.022184
MIR3147	0.009003
ZNF716	0.009003
RN7SL855P	0.019141
ZNF733P	0.019141
RN7SL371P	0.032468
WBSCR17	0.044072
MTRNR2L6	0.044628
PRSS1	0.044628
PRSS3P2	0.044628
PRSS3P3	0.044628
TRBC2	0.044628
TRBV19	0.044628
TRBV27	0.044628
TRBV28	0.044628
TRBV2	0.044628
TRBV9	0.044628
RN7SL207P	0.034311
CNTNAP2	0.039497
MIR548F4	0.03251
RNA5SP249	0.042982
RN7SL456P	0.043238
KMT2C	0.039318
RNA5SP250	0.039267
snoU13|ENSG00000238557.1	0.039267
SNORA26|ENSG00000212590.1	0.030758
ERICH1	0.00101
FBXO25	0.000716
OR4F21	0.000716
RPL23AP53	0.000716
TDRP	0.000716
ZNF596	0.000716
DLGAP2	0.000366
CLN8	0.000421
MIR596	0.000421
ARHGEF10	0.000414
KBTBD11	0.001149
MYOM2	0.001161
CSMD1	0.003793
RNA5SP251	0.001699
RN7SL872P	0.00503
SNORA70|ENSG00000206661.1	0.005242
RN7SL318P	0.005242
RN7SKP159	0.002765
MCPH1	0.012443
ANGPT2	0.006819
AGPAT5	0.011022
MIR4659A	0.008781
XKR5	0.00956

DEFB1	0.01096
DEFA6	0.016507
DEFA4	0.016507
ALG1L13P	0.004984
DEFA1B	0.004984
DEFA1	0.004984
DEFA3	0.004984
DEFA5	0.004984
DEFB103A	0.004984
DEFB103B	0.004984
DEFB104A	0.004984
DEFB104B	0.004984
DEFB105A	0.004984
DEFB105B	0.004984
DEFB106A	0.004984
DEFB106B	0.004984
DEFB107A	0.004984
DEFB107B	0.004984
DEFB4A	0.004984
DEFB4B	0.004984
FAM66B	0.004984
FAM66E	0.004984
FAM85B	0.004984
FAM86B3P	0.004984
FAM90A24P	0.004984
LRLE1	0.004984
MIR548I3	0.004984
SGK223	0.004984
SPAG11A	0.004984
SPAG11B	0.004984
ZNF705B	0.004984
ZNF705G	0.004984
RN7SL178P	0.005967
CLDN23	0.005967
MFHAS1	0.003908
SNORA70|ENSG00000207244.1	0.002963
ERI1	0.00328
MIR4660	0.00328
PPP1R3B	0.00328
U3|ENSG00000252543.1	0.00328
TNKS	0.00431
MIR597	0.006496
snoU13|ENSG00000239065.1	0.009554
LINC00599	0.009554
snoU13|ENSG00000238496.1	0.004881
MSRA	0.004881
PRSS51	0.004881
PRSS55	0.007299
RP1L1	0.009535
MIR4286	0.007299
C8orf74	0.009535
RNA5SP252	0.007991
SOX7	0.009313
PINX1	0.009313
SNORD112|ENSG00000252565.1	0.009313
XKR6	0.004777
MIR598	0.003753
LINC00529	0.00502

MTMR9	0.005566
SLC35G5	0.005566
TDH	0.005566
C8orf12	0.007591
RN7SL293P	0.005641
FAM167A	0.009382
BLK	0.007591
LINC00208	0.007591
GATA4	0.004406
C8orf49	0.006252
NEIL2	0.006252
CTSB	0.002504
DEFB130|ENSG00000232948.1	0.002504
DEFB130|ENSG00000233050.1	0.002504
DEFB134	0.002504
DEFB135	0.002504
DEFB136	0.002504
FAM66A	0.002504
FAM66D	0.002504
FAM86B1	0.002504
FAM86B2	0.002504
FDFT1	0.002504
RNA5SP253	0.002504
RNA5SP254	0.002504
USP17L2	0.002504
ZNF705D	0.002504
MIR5692A2	0.004983
LONRF1	0.004983
LINC00681	0.005526
KIAA1456	0.006605
DLC1	0.011093
RNA5SP255	0.009019
C8orf48	0.005625
SGCZ	0.007563
MIR383	0.006453
TUSC3	0.00477
MSR1	0.009808
RN7SL474P	0.003531
FGF20	0.001843
MICU3	0.002163
ZDHHC2	0.001606
CNOT7	0.001606
VPS37A	0.001606
MTMR7	0.002163
SLC7A2	0.00289
PDGFRL	0.00289
MTUS1	0.00208
MIR548V	0.003773
RNA5SP256	0.00208
FGL1	0.003845
PCM1	0.003845
ASAH1	0.003845
NAT1	0.003426
NAT2	0.002042
PSD3	0.00071
SNORA62|ENSG00000201157.1	0.002327
SH2D4A	0.001922
CSGALNACT1	0.003015

INTS10	0.003649
LPL	0.004638
SLC18A1	0.004638
ATP6V1B2	0.004638
LZTS1	0.004638
U3|ENSG00000251944.1	0.002652
GFRA2	0.004931
DOK2	0.004931
XPO7	0.005946
NPM2	0.005946
snoU13|ENSG00000238466.1	0.005946
FGF17	0.005946
DMTN	0.005946
FAM160B2	0.005946
NUDT18	0.005946
HR	0.005946
REEP4	0.005946
LGI3	0.005946
SFTPC	0.005946
BMP1	0.008944
PHYHIP	0.007399
MIR320A	0.008537
POLR3D	0.008537
PIWIL2	0.006676
PPP3CC	0.005731
SLC39A14	0.005731
SORBS3	0.005731
PDLIM2	0.005731
C8orf58	0.005731
KIAA1967	0.005731
BIN3	0.005731
EGR3	0.005731
PEBP4	0.005007
RN7SL303P	0.005731
RHOBTB2	0.005007
TNFRSF10B	0.005007
TNFRSF10C	0.005007
TNFRSF10D	0.009036
TNFRSF10A	0.010965
CHMP7	0.010965
R3HCC1	0.012687
LOXL2	0.007987
ENTPD4	0.005408
SLC25A37	0.006648
FP15737	0.005134
SNORA67|ENSG00000207027.1	0.006053
STC1	0.006053
ADAM28	0.004021
ADAMDEC1	0.005433
ADAM7	0.005433
NEFM	0.008422
NEFL	0.008422
RN7SL651P	0.0075
DOCK5	0.0075
GNRH1	0.0075
KCTD9	0.0075
CDCA2	0.0075
EBF2	0.008662

RNA5SP258	0.008662
PPP2R2A	0.01193
BNIP3L	0.019845
SDAD1P1	0.009543
PNMA2	0.011223
DPYSL2	0.011223
ADRA1A	0.009091
MIR548H4	0.009091
STMN4	0.008318
TRIM35	0.009874
PTK2B	0.009874
CHRNA2	0.009874
EPHX2	0.009874
CLU	0.007711
SCARA3	0.009593
MIR3622B	0.007622
CCDC25	0.007622
snoU13|ENSG00000238624.1	0.007622
ESCO2	0.007622
PBK	0.006513
SCARA5	0.008186
MIR4287	0.008186
NUGGC	0.008186
ELP3	0.006888
PNOC	0.009884
ZNF395	0.005508
FBXO16	0.006513
FZD3	0.011842
MIR4288	0.011842
RNA5SP259	0.011842
EXTL3	0.014261
INTS9	0.014872
HMBOX1	0.014872
RNA5SP260	0.014872
KIF13B	0.010815
RN7SL781P	0.014872
DUSP4	0.010815
LINC00589	0.008489
FAM183CP	0.014388
MIR3148	0.014388
TMEM66	0.014388
LEPROTL1	0.009557
MBOAT4	0.013134
DCTN6	0.013134
TUBBP1	0.013134
RBPMS	0.016158
GTF2E2	0.014756
SMIM18	0.016903
GSR	0.014756
UBXN8	0.021327
PPP2CB	0.017089
TEX15	0.012326
PURG	0.012326
WRN	0.017089
RNA5SP261	0.023022
NRG1	0.00439
RNA5SP262	0.012109
RNA5SP263	0.012109

FUT10	0.014569
TTI2	0.01396
MAK16	0.01396
SNORD13	0.01396
SNORA70|ENSG00000252505.1	0.01396
RNF122	0.010721
RN7SL621P	0.010018
DUSP26	0.010018
RN7SL457P	0.01156
UNC5D	0.007573
RN7SKP201	0.005202
RNA5SP264	0.001585
KCNU1	0.003435
ZNF703	2.36E-05
ERLIN2	1.62E-05
GPR124	7.72E-05
PROSC	1.41E-05
BRF2	7.83E-05
RAB11FIP1	0.000115
RN7SL709P	8.11E-05
GOT1L1	8.11E-05
ADRB3	7.62E-05
EIF4EBP1	2.77E-05
ASH2L	5.60E-06
STAR	1.94E-05
LSM1	1.56E-05
BAG4	1.23E-05
DDHD2	1.06E-05
PPAPDC1B	1.06E-05
WHSC1L1	7.06E-06
LETM2	1.49E-05
FGFR1	7.31E-06
RPS20P22	1.27E-05
C8orf86	3.08E-05
TACC1	0.000117
PLEKHA2	5.92E-05
HTRA4	7.93E-05
TM2D2	7.93E-05
ADAM9	4.81E-05
SNORD38|ENSG00000207199.1	0.000156
ADAM32	7.93E-05
ADAM5	5.14E-05
ADAM3A	5.40E-05
ADAM18	8.83E-05
ADAM2	5.46E-05
IDO1	0.000207
IDO2	0.000118
C8orf4	4.05E-05
ZMAT4	9.11E-06
SFRP1	0.000163
MIR548AO	0.000185
SNORD65|ENSG00000238936.1	7.98E-05
GOLGA7	6.51E-05
GINS4	6.51E-05
AGPAT6	1.67E-05
ANK1	5.90E-06
MIR486	1.24E-05
RN7SL149P	7.14E-06

KAT6A	9.02E-06
SNORD112|ENSG00000238966.1	9.02E-06
AP3M2	1.49E-05
PLAT	1.81E-05
snoU13|ENSG00000238714.1	9.38E-06
IKBKB	2.56E-05
POLB	0.000112
DKK4	8.79E-05
VDAC3	8.79E-05
SLC20A2	0.000142
SMIM19	0.00028
CHRNB3	6.89E-05
CHRNA6	6.51E-05
THAP1	0.000238
RNF170	0.000212
RN7SL806P	0.000212
HOOK3	0.000525
MIR4469	0.000212
FNTA	3.08E-05
HGSNAT	1.66E-05
POTEA	1.66E-05
RN7SKP41	1.66E-05
SGK196	1.66E-05
U3|ENSG00000201329.1	1.66E-05
LINC00293	0.000293
RN7SKP32	0.00027
SPIDR	0.000246
CEBPD	0.000447
PRKDC	0.000349
MCM4	0.000281
UBE2V2	0.000526
EFCAB1	0.000175
SNAI2	0.000445
C8orf22	0.000361
RN7SKP294	0.000216
SNTG1	3.54E-05
SNORA7|ENSG00000201316.1	0.000117
PXDNL	2.94E-05
PCMTD1	4.36E-05
ST18	3.45E-05
FAM150A	4.16E-05
RB1CC1	4.26E-05
NPBWR1	4.51E-05
OPRK1	3.06E-05
ATP6V1H	2.17E-05
RGS20	5.46E-05
TCEA1	4.81E-05
LYPLA1	9.55E-06
MRPL15	9.30E-06
RNU6ATAC32P	9.30E-06
RNU105C	4.14E-06
RN7SL250P	4.14E-06
SOX17	4.14E-06
RP1	2.95E-06
XKR4	5.16E-06
TMEM68	2.57E-06
RNA5SP265	3.58E-06
TGS1	3.15E-06

LYN	1.63E-06
SNORA1|ENSG00000199405.1	3.15E-06
RN7SL798P	1.63E-06
RN7SL323P	2.23E-06
RPS20	2.23E-06
SNORD54	2.23E-06
MOS	2.23E-06
SNORA3|ENSG00000221093.1	2.23E-06
PLAG1	1.58E-06
CHCHD7	1.58E-06
SDR16C5	1.10E-06
SDR16C6P	1.10E-06
PENK	1.23E-06
LINC00968	1.10E-06
IMPAD1	1.30E-06
RNA5SP266	4.61E-06
LINC00588	1.06E-05
FAM110B	1.07E-06
UBXN2B	1.03E-06
CYP7A1	1.03E-06
SDCBP	1.03E-06
NSMAF	1.29E-06
snoU13|ENSG00000238433.1	1.29E-06
TOX	2.06E-06
SNORA51|ENSG00000206853.1	2.06E-06
RNA5SP267	6.44E-06
CA8	1.91E-05
RAB2A	2.03E-05
CHD7	1.97E-05
CLVS1	3.63E-05
ASPH	7.93E-05
RN7SKP97	6.01E-05
MIR4470	7.93E-05
NKAIN3	5.36E-05
GGH	0.000209
TTPA	0.000209
YTHDF3	0.000122
RN7SL135P	0.000122
RN7SKP135	0.000136
LINC00966	0.00041
BHLHE22	0.000345
CYP7B1	0.000345
ARMC1	0.000571
MTFR1	0.000571
PDE7A	0.000818
DNAJC5B	0.000414
TRIM55	0.000414
CRH	0.000414
LINC00967	0.000414
ADHFE1	0.000414
RRS1	0.000414
C8orf46	0.000999
MYBL1	0.000348
VCPIP1	0.000348
C8orf44	0.000348
SGK3	0.000348
MCMDC2	0.000238
SNHG6	0.000238

SNORD87	0.000238
TCF24	0.000348
PPP1R42	0.000348
COPS5	0.000205
CSPP1	0.000171
RNA5SP268	0.000205
ARFGEF1	0.000248
CPA6	0.000297
PREX2	5.93E-05
C8orf34	9.91E-05
RNA5SP269	0.000117
SULF1	0.000375
SLCO5A1	0.000511
RN7SKP29	0.000244
RN7SL675P	0.000347
RNA5SP270	0.000347
PRDM14	0.000671
NCOA2	0.000452
TRAM1	0.000242
snoU13|ENSG00000238450.1	0.000242
LACTB2	0.000242
RN7SL19P	0.000294
XKR9	0.000294
EYA1	0.000709
U8|ENSG00000200191.1	0.000377
MSC	0.000377
TRPA1	0.000761
RNA5SP271	0.000361
KCNB2	0.000272
TERF1	0.000251
SBSPON	0.000251
RPL7	0.00021
RDH10	0.00021
STAU2	0.000367
UBE2W	0.000403
TCEB1	0.000273
TMEM70	0.000273
LY96	0.000273
JPH1	0.000273
GDAP1	0.000273
MIR5681A	0.000323
PI15	0.000298
CRISPLD1	0.000196
CASC9	0.000199
HNF4G	0.000317
snoU13|ENSG00000238595.1	0.000307
ZFHX4	0.000225
PEX2	0.000143
PKIA	0.000755
ZC2HC1A	0.000648
IL7	0.000547
STMN2	0.000213
HEY1	0.000243
MRPS28	0.000362
TPD52	0.000362
RN7SL41P	0.000362
MIR5708	0.000362
SNORA20|ENSG00000206649.1	0.000362

ZBTB10	0.000461
RN7SL107P	0.000525
ZNF704	0.0006
RN7SL308P	0.000664
PAG1	0.000709
FABP5	0.000723
PMP2	0.000939
FABP9	0.000939
FABP4	0.000918
FABP12	0.001106
IMPA1P	0.001628
IMPA1	0.001143
SLC10A5	0.001106
ZFAND1	0.001106
CHMP4C	0.001106
SNX16	0.000918
RALYL	0.002037
snoU13|ENSG00000238566.1	0.001355
LRRCC1	0.001355
E2F5	0.001323
C8orf59	0.001323
CA13	0.001323
CA1	0.001285
CA3	0.002125
CA2	0.001114
REXO1L10P|ENSG00000255940.1	0.001868
REXO1L10P|ENSG00000270416.1	0.001868
REXO1L11P	0.001868
REXO1L1	0.001868
ATP6V0D2	0.001932
PSKH2	0.001932
SLC7A13	0.001485
WWP1	0.001876
RMDN1	0.002843
CPNE3	0.002843
CNGB3	0.002339
CNBD1	0.001953
DCAF4L2	0.003988
MMP16	0.004885
RNA5SP272	0.002958
RIPK2	0.006473
OSGIN2	0.003767
NBN	0.002904
DECR1	0.002868
CALB1	0.00301
LINC00534	0.004921
RNA5SP273	0.008867
TMEM64	0.007956
NECAB1	0.008247
TMEM55A	0.011309
OTUD6B	0.011586
LRRC69	0.011586
RN7SL777P	0.011586
MIR4661	0.011586
SLC26A7	0.011586
snoU13|ENSG00000239134.1	0.01385
RN7SKP231	0.012639
RUNX1T1	0.016844

TRIQK	0.020507
C8orf87	0.015525
LINC00535	0.011443
RNA5SP274	0.019615
FAM92A1	0.01426
RBM12B	0.01426
TMEM67	0.01426
PDP1	0.01426
MIR378D2	0.01426
CDH17	0.015333
GEM	0.01426
FSBP	0.014425
RAD54B	0.014425
KIAA1429	0.012993
ESRP1	0.014961
DPY19L4	0.017596
INTS8	0.017596
CCNE2	0.020091
NDUFAF6	0.020091
snoU13|ENSG00000238791.1	0.020091
TP53INP1	0.020091
MIR3150B	0.020091
PLEKHF2	0.020091
C8orf37	0.030136
GDF6	0.019269
MTERFD1	0.03249
PTDSS1	0.041443
UQCRB	0.03249
SDC2	0.03479
CPQ	0.0394
MATN2	0.031125
RPL30	0.031125
SNORA72|ENSG00000207067.1	0.031125
C8orf47	0.031125
HRSP12	0.04013
POP1	0.04013
NIPAL2	0.038008
KCNS2	0.044841
VPS13B	0.009758
MIR599	0.022943
MIR875	0.022943
RN7SL350P	0.03016
COX6C	0.023616
RGS22	0.032614
SNORD77|ENSG00000212414.1	0.032192
MIR1273A	0.032488
FBXO43	0.040453
POLR2K	0.040453
SPAG1	0.031516
RNF19A	0.026511
MIR4471	0.018693
ANKRD46	0.024591
SNX31	0.024835
PABPC1	0.024055
RNU6ATAC41P	0.024055
YWHAZ	0.024055
RN7SL685P	0.022638
RN7SKP249	0.027383

ZNF706	0.028906
NACAP1	0.018446
RN7SL563P	0.018446
GRHL2	0.012339
NCALD	0.02192
snoU13|ENSG00000238372.1	0.016036
MIR5680	0.022139
RRM2B	0.023183
UBR5	0.022139
snoU13|ENSG00000238533.1	0.021278
RNU6ATAC8P	0.021278
ODF1	0.027
KLF10	0.020924
AZIN1	0.018228
ATP6V1C1	0.022239
C8orf56	0.021278
BAALC	0.021278
MIR3151	0.021278
FZD6	0.021278
CTHRC1	0.015842
SLC25A32	0.015842
DCAF13	0.020973
RIMS2	0.021923
snoU13|ENSG00000238687.1	0.016077
DPYS	0.00778
DCSTAMP	0.00778
MIR548A3	0.009006
LRP12	0.009006
ZFPM2	0.021923
OXR1	0.015839
ABRA	0.017257
ANGPT1	0.018834
RNA5SP275	0.012263
RSPO2	0.012263
EIF3E	0.015595
EMC2	0.015595
TMEM74	0.008974
TRHR	0.008259
NUDCD1	0.004844
ENY2	0.004844
PKHD1L1	0.004844
EBAG9	0.004844
SYBU	0.004844
SNORD112|ENSG00000252559.1	0.004844
KCNV1	0.006036
CSMD3	0.003249
snoU13|ENSG00000238656.1	0.008283
MIR2053	0.007529
TRPS1	0.002176
LINC00536	0.001869
RNA5SP276	0.001915
EIF3H	0.001933
UTP23	0.001505
RAD21	0.001505
MIR3610	0.001505
AARD	0.001495
SLC30A8	0.001086
RN7SL228P	0.001495

RN7SL826P	0.000778
SNORA31|ENSG00000252852.1	0.000554
MED30	0.000531
EXT1	0.00062
SAMD12	0.000824
TNFRSF11B	0.000711
COLEC10	0.000646
MAL2	0.00095
SNORA32|ENSG00000206776.1	0.000721
NOV	0.000721
ENPP2	0.000709
RN7SKP153	0.000747
TAF2	0.00085
DSCC1	0.001672
RN7SL396P	0.001672
DEPTOR	0.002102
RNA5SP277	0.001672
COL14A1	0.000851
MRPL13	0.001548
MTBP	0.001375
SNTB1	0.001226
HAS2	0.001087
snoU13|ENSG00000238901.1	0.001747
ZHX2	0.002475
DERL1	0.002897
RNY4P5	0.002897
TBC1D31	0.003435
FAM83A	0.001962
U3|ENSG00000221461.1	0.002486
MIR4663	0.001962
C8orf76	0.001962
ZHX1	0.002303
snoU13|ENSG00000238422.1	0.001692
ATAD2	0.001923
MIR548AA1	0.001591
WDYHV1	0.001597
FBXO32	0.002692
RN7SKP155	0.002692
KLHL38	0.002692
ANXA13	0.002694
FAM91A1	0.002694
FER1L6	0.002119
TMEM65	0.002508
TRMT12	0.002172
RNF139	0.002172
TATDN1	0.002172
NDUFB9	0.00167
MTSS1	0.00167
MIR4662B	0.001841
LINC00964	0.001841
ZNF572	0.001841
SQLE	0.001841
KIAA0196	0.001841
NSMCE2	0.002058
RN7SL329P	0.001914
TRIB1	0.001987
RN7SL590P	0.002282
LINC00861	0.001517

FAM84B	0.002543
PCAT1	0.001074
PCAT2	0.001469
CCAT1	0.002056
CASC8	0.003457
POU5F1B	0.003202
MYC	0.000615
PVT1	0.000129
TMEM75	0.000434
MIR1205	0.000322
MIR1207	0.00021
MIR1208	0.000804
RN7SKP226	0.000404
LINC00977	0.0018
RN7SKP206	0.002204
CCDC26	0.001544
MIR3686	0.003594
GSDMC	0.002529
FAM49B	0.001686
SNORA25|ENSG00000200075.1	0.002049
MIR5194	0.001079
ASAP1	0.001401
SNORA12|ENSG00000212342.1	0.000839
ADCY8	0.000933
SNORA72|ENSG00000252158.1	0.000218
EFR3A	0.000486
OC90	0.000419
HHLA1	0.000571
KCNQ3	0.000564
HPYR1	0.001153
LRRC6	0.00089
TMEM71	0.001297
PHF20L1	0.001037
TG	0.001037
SLA	0.001081
WISP1	0.001319
NDRG1	0.000937
ST3GAL1	0.000904
SNORA40|ENSG00000212273.1	0.00107
ZFAT	0.001193
MIR30B	0.001345
MIR30D	0.001345
KHDRBS3	0.001544
FAM135B	0.000176
COL22A1	0.000304
SNORA25|ENSG00000251744.1	0.000436
KCNK9	0.000454
TRAPPC9	0.000634
C8orf17	0.000893
CASC7	0.001053
CHRAC1	0.001053
AGO2	0.000598
DENND3	0.000412
MIR151A	0.000505
PTK2	0.000505
RNA5SP278	0.000505
SLC45A4	0.000412
GPR20	0.000292

LINC00051	0.000292
MROH5	0.000292
PTP4A3	0.000292
SNORD5|ENSG00000238854.1	0.000292
TSNARE1	0.000292
BAI1	0.000382
ARC	0.000521
JRK	0.000394
PSCA	0.000394
LY6K	0.000394
THEM6	0.000394
SLURP1	0.000394
LYPD2	0.000394
LY6D	0.000394
LYNX1	0.000394
GML	0.000394
CYP11B1	0.000394
CYP11B2	0.000394
LY6E	0.000394
C8orf31	0.000345
LY6H	0.000675
GPIHBP1	0.000524
ZFP41	0.000524
GLI4	0.000524
ZNF696	0.000524
TOP1MT	0.000524
RHPN1	0.000552
MAFA	0.000552
ZC3H3	0.000606
7SK|ENSG00000254144.2	0.000606
GSDMD	0.000606
MROH6	0.000606
EEF1D	0.000606
NAPRT1	0.000606
TIGD5	0.000749
PYCRL	0.000749
TSTA3	0.000749
ZNF623	0.000966
CCDC166	0.000966
FAM83H	0.000966
MAPK15	0.000966
MIR4664	0.000966
ZNF707	0.000966
SCRIB	0.000966
MIR937	0.000966
PUF60	0.000966
NRBP2	0.000966
EPPK1	0.000966
PLEC	0.000534
MIR661	0.000668
PARP10	0.000534
GRINA	0.000534
SPATC1	0.000534
OPLAH	0.000534
EXOSC4	0.000534
GPAA1	0.000534
CYC1	0.000534
SHARPIN	0.000778

KIAA1875	0.000778
MAF1	0.000778
FAM203A	0.000778
MROH1	0.000815
ADCK5	0.00139
ARHGAP39	0.00139
BOP1	0.00139
C8orf33	0.00139
C8orf82	0.00139
COMMD5	0.00139
CPSF1	0.00139
CYHR1	0.00139
DGAT1	0.00139
FAM203B	0.00139
FBXL6	0.00139
FOXH1	0.00139
GPT	0.00139
HSF1	0.00139
KIFC2	0.00139
LRRC14	0.00139
LRRC24	0.00139
MFSD3	0.00139
MIR1234	0.00139
MIR939	0.00139
PPP1R16A	0.00139
RECQL4	0.00139
RN7SL395P	0.00139
RPL8	0.00139
SCRT1	0.00139
SCXA	0.00139
SCXB	0.00139
SLC39A4	0.00139
SLC52A2	0.00139
TMEM249	0.00139
TONSL	0.00139
VPS28	0.00139
ZNF16	0.00139
ZNF250	0.00139
ZNF251	0.00139
ZNF252P	0.00139
ZNF34	0.00139
ZNF517	0.00139
ZNF7	0.00139
C9orf66	0.00341
CBWD1	0.00341
DOCK8	0.00341
FAM138C	0.00341
FOXD4	0.00341
KANK1	0.00341
RN7SL412P	0.00341
DMRT1	0.005211
DMRT2	0.004689
DMRT3	0.004689
RNA5SP279	0.004689
SMARCA2	0.004433
RN7SL592P	0.004092
VLDLR	0.003271
KCNV2	0.001724

KIAA0020	0.001862
CARM1P1	0.005842
RFX3	0.004288
GLIS3	0.012144
SLC1A1	0.007357
SPATA6L	0.007357
PPAPDC2	0.007357
CDC37L1	0.007357
AK3	0.008545
RCL1	0.008044
JAK2	0.005932
INSL4	0.007715
INSL6	0.007715
RLN2	0.01366
RLN1	0.01366
PLGRKT	0.01366
CD274	0.01366
PDCD1LG2	0.01366
KIAA1432	0.01366
ERMP1	0.010831
KIAA2026	0.010545
MLANA	0.010545
snoU13|ENSG00000238654.1	0.011673
MIR4665	0.013377
RANBP6	0.013377
IL33	0.028685
C9orf38	0.028685
GLDC	0.028685
KDM4C	0.023858
RN7SL123P	0.028685
RN7SL25P	0.028685
TPD52L3	0.028685
UHRF2	0.028685
snoU13|ENSG00000252110.1	0.028685
C9orf123	0.008738
PTPRD	0.032879
SNORD27|ENSG00000251699.1	0.003011
RN7SL5P	0.024694
TYRP1	0.019061
RN7SL849P	0.024562
LURAP1L	0.014047
MPDZ	0.011827
LINC00583	0.009293
NFIB	0.012747
ZDHHC21	0.001575
CER1	0.001819
FREM1	0.001819
TTC39B	0.005523
RN7SL157P	0.003069
SNAPC3	0.003069
PSIP1	0.002648
RN7SL98P	0.002648
CCDC171	0.001269
C9orf92	0.00572
BNC2	0.004076
RN7SL720P	0.004155
CNTLN	0.009271
SH3GL2	0.001406

ADAMTSL1	0.000371
MIR3152	0.001164
RN7SKP258	0.000912
FAM154A	0.000912
RRAGA	0.00138
HAUS6	0.00138
SCARNA8	0.00138
RN7SL158P	0.00138
PLIN2	0.00138
DENND4C	0.000546
snoU13|ENSG00000238348.1	0.000732
RPS6	0.000991
ACER2	0.002105
SLC24A2	0.001249
MLLT3	0.000745
MIR4473	0.001891
MIR4474	0.001545
FOCAD	0.001619
MIR491	0.004919
SNORA30|ENSG00000202189.1	0.007541
PTPLAD2	0.001276
IFNB1	0.004019
IFNW1	0.006823
IFNA21	0.005443
IFNA4	0.006525
IFNA10	0.006416
IFNA16	0.006416
IFNA7	0.006416
IFNA17	0.006416
IFNA14	0.006416
IFNA5	0.006558
KLHL9	0.005948
IFNA6	0.005195
IFNA13	0.005195
IFNA2	0.006107
IFNA8	0.006107
IFNA1	0.005871
MIR31HG	0.003143
IFNE	0.003679
MIR31	0.003143
SNORD39|ENSG00000264379.1	0.003143
RN7SL151P	0.005362
MTAP	0.015599
C9orf53	0.018814
CDKN2A	0.017487
CDKN2B	0.008741
DMRTA1	0.038028
ELAVL2	0.026496
IZUMO3	0.018514
RMRPP5	0.006884
RN7SKP120	0.006495
TUSC1	0.00296
SNORA31|ENSG00000252580.1	0.011119
CAAP1	0.012092
RN7SL100P	0.007963
PLAA	0.0072
IFT74	0.011268
LRRC19	0.012969

TEK	0.005266
RNA5SP280	0.014629
LINC00032	0.009536
EQTN	0.009536
MOB3B	0.006474
IFNK	0.015591
C9orf72	0.018774
PAX5	0.038684
ZCCHC7	0.047644
GRHPR	0.036575
ZBTB5	0.024891
RN7SL463P	0.030416
POLR1E	0.030416
FBXO10	0.030416
TOMM5	0.035418
U8|ENSG00000200026.1	0.042739
snoU13|ENSG00000238313.1	0.042739
ALDH1B1	0.034391
IGFBPL1	0.042739
ANKRD18A	0.028616
SNX18P3	0.019212
FAM201A	0.028616
ANKRD20A1	0.017283
ANKRD20A2	0.017283
ANKRD20A3	0.017283
ANKRD20A4	0.017283
CBWD3	0.017283
CBWD5	0.017283
CBWD6	0.017283
CBWD7	0.017283
CNTNAP3B	0.017283
CNTNAP3	0.017283
FAM27A	0.017283
FAM27B	0.017283
FAM27C	0.017283
FAM27D1	0.017283
FAM27E1	0.017283
FAM27E2	0.017283
FAM27E3	0.017283
FAM74A1	0.017283
FAM74A2	0.017283
FAM74A3	0.017283
FAM74A4	0.017283
FAM74A5	0.017283
FAM74A6	0.017283
FAM95B1	0.017283
FOXD4L2	0.017283
FOXD4L3	0.017283
FOXD4L4	0.017283
FOXD4L5	0.017283
FOXD4L6	0.017283
MIR1299	0.017283
MIR4477A	0.017283
PGM5P2	0.017283
PGM5	0.004581
RN7SL343P	0.017283
RN7SL422P	0.017283
RN7SL462P	0.017283

RN7SL544P	0.017283
RN7SL565P	0.017283
RN7SL640P	0.017283
RN7SL722P	0.017283
RN7SL763P	0.017283
RN7SL787P	0.017283
RNA5SP283	0.017283
RNA5SP284	0.017283
SNORA70|ENSG00000252133.1	0.017283
SNORA70|ENSG00000252617.1	0.017283
SNORA70|ENSG00000252724.1	0.017283
SNORA70|ENSG00000252878.1	0.017283
SPATA31A1	0.017283
SPATA31A2	0.017283
SPATA31A3	0.017283
SPATA31A4	0.017283
SPATA31A5	0.017283
SPATA31A6	0.017283
SPATA31A7	0.017283
ZNF658	0.017283
PIP5K1B	0.014982
FAM122A	0.01975
PRKACG	0.016916
FXN	0.013095
TJP2	0.018038
FAM189A2	0.023644
APBA1	0.026944
PTAR1	0.02413
C9orf135	0.02413
RN7SL570P	0.026944
MAMDC2	0.017702
SMC5	0.015493
KLF9	0.015493
TRPM3	0.017662
MIR204	0.021142
TMEM2	0.024598
ABHD17B	0.024598
C9orf85	0.024598
C9orf57	0.024598
GDA	0.024598
RNA5SP285	0.024598
ZFAND5	0.024598
TMC1	0.03167
snoU13|ENSG00000238402.1	0.023765
ALDH1A1	0.017065
ANXA1	0.021214
RNA5SP286	0.003076
RORB	0.003067
TRPM6	0.003076
RN7SKP47	0.003739
RNY4P1	0.003739
C9orf40	0.003739
C9orf41	0.003739
NMRK1	0.003739
OSTF1	0.004234
snoU13|ENSG00000238598.1	0.003483
PCSK5	0.016375
RFK	0.019458

GCNT1	0.02537
PRUNE2	0.012581
PCA3	0.015633
FOXB2	0.012581
VPS13A	0.009007
GNA14	0.012607
GNAQ	0.018089
RN7SKP59	0.008206
CEP78	0.008206
PSAT1	0.008206
TLE4	0.019076
TLE1	0.017559
RNA5SP287	0.014375
SNORD95|ENSG00000200969.1	0.022392
SPATA31D5P	0.022392
SPATA31D4	0.022392
SPATA31D3	0.022392
SPATA31D1	0.022392
RASEF	0.02726
RN7SKP242	0.01383
FRMD3	0.017364
snoU13|ENSG00000238608.1	0.020224
SNORD112|ENSG00000252256.1	0.020224
IDNK	0.017364
UBQLN1	0.017364
GKAP1	0.027454
KIF27	0.03107
C9orf64	0.021356
HNRNPK	0.028779
RMI1	0.028779
SLC28A3	0.028779
NTRK2	0.021782
AGTPBP1	0.027188
NAA35	0.02458
GOLM1	0.02458
C9orf153	0.029235
RN7SKP264	0.029235
ISCA1	0.029235
ZCCHC6	0.029235
GAS1	0.029235
C9orf170	0.020967
SNORA26|ENSG00000212421.1	0.020967
DAPK1	0.027688
CTSL	0.024474
CTSL3P	0.024474
SPATA31E1	0.026485
SPATA31C1	0.026485
CDK20	0.026485
U6|ENSG00000271923.1	0.026485
U3|ENSG00000252299.1	0.028619
SPIN1	0.028619
NXNL2	0.028619
MIR4289	0.017151
C9orf47	0.014737
S1PR3	0.014737
SHC3	0.017384
CKS2	0.010493
MIR3153	0.010493

SECISBP2	0.010581
SEMA4D	0.013699
GADD45G	0.013699
MIR4290	0.010714
DIRAS2	0.008057
SYK	0.008057
LINC00484	0.010449
AUH	0.010449
NFIL3	0.010449
ROR2	0.011555
SPTLC1	0.012588
LINC00475	0.020531
snoU13|ENSG00000238996.1	0.012588
IARS	0.020531
SNORA84	0.020531
NOL8	0.020531
ASPN	0.020531
CENPP	0.020531
ECM2	0.020531
MIR4670	0.020531
OGN	0.020531
OMD	0.020531
IPPK	0.02665
BICD2	0.020531
ANKRD19P	0.020531
ZNF484	0.020531
FGD3	0.020531
SUSD3	0.020531
C9orf89	0.020531
NINJ1	0.020531
WNK2	0.039769
C9orf129	0.039769
FAM120AOS	0.032365
FAM120A	0.032117
PHF2	0.027802
MIR4291	0.028316
BARX1	0.025326
PTPDC1	0.020719
MIRLET7A1	0.020719
MIRLET7DHG	0.025326
MIRLET7F1	0.020719
MIRLET7D	0.025326
snoU13|ENSG00000238792.1	0.025326
ZNF169	0.025326
NUTM2F	0.025326
HIATL1	0.017275
PCAT7	0.017486
FBP2	0.017486
FBP1	0.017486
C9orf3	0.019577
MIR2278	0.01907
MIR23B	0.017486
MIR27B	0.017486
MIR3074	0.017486
FANCC	0.025326
RNA5SP288	0.017486
snoU13|ENSG00000238746.1	0.021481
PTCH1	0.02644

LINC00476	0.022927
DKFZP434H0512	0.022927
ERCC6L2	0.022927
RNA5SP289	0.022927
LINC00092	0.022927
HSD17B3	0.015959
SLC35D2	0.012948
ZNF367	0.012948
HABP4	0.01851
CDC14B	0.01851
AAED1	0.026707
ZNF510	0.024551
ZNF782	0.031422
HIATL2	0.034652
NUTM2G	0.044702
CTSV	0.039024
CCDC180	0.031422
TDRD7	0.034652
TMOD1	0.034652
TSTD2	0.044702
NCBP1	0.044702
XPA	0.034036
FOXE1	0.028977
C9orf156	0.033449
HEMGN	0.033449
ANP32B	0.033449
NANS	0.028929
TRIM14	0.035821
CORO2A	0.044457
TBC1D2	0.035821
GABBR2	0.028977
ANKS6	0.029244
GALNT12	0.029244
COL15A1	0.020082
TGFBR1	0.020082
RNA5SP290	0.020082
RN7SL794P	0.020082
ALG2	0.020082
SEC61B	0.020082
RN7SKP225	0.020082
NR4A3	0.033235
STX17	0.033235
ERP44	0.025194
INVS	0.016959
RN7SL75P	0.025194
TEX10	0.022641
MSANTD3	0.021515
TMEFF1	0.017268
MURC	0.026242
RN7SKP87	0.026242
LPPR1	0.041189
RNF20	0.040033
PPP3R2	0.04488
LINC00587	0.025966
CYLC2	0.022656
RNA5SP291	0.005744
SMC2	0.00623
OR13F1	0.011844

OR13C4	0.011844
OR13C3	0.011844
OR13C8	0.011844
OR13C2	0.011844
OR13C5	0.011844
OR13C9	0.011844
OR13D1	0.011844
NIPSNAP3A	0.011844
NIPSNAP3B	0.011844
ABCA1	0.009682
RN7SKP191	0.011052
SLC44A1	0.015276
FSD1L	0.015276
FKTN	0.010782
TAL2	0.010782
TMEM38B	0.01166
RN7SKP77	0.009274
RNA5SP292	0.016034
ZNF462	0.015674
RAD23B	0.015674
KLF4	0.015674
RN7SL659P	0.018206
RNA5SP293	0.018206
ACTL7B	0.025382
ACTL7A	0.025382
IKBKAP	0.025382
FAM206A	0.025382
CTNNAL1	0.02709
TMEM245	0.020506
MIR32	0.020506
FRRS1L	0.015814
EPB41L4B	0.015814
PTPN3	0.015814
MIR3927	0.015814
PALM2	0.013097
AKAP2	0.008485
C9orf152	0.011033
TXNDC8	0.011033
TXN	0.011033
SVEP1	0.006055
MUSK	0.014117
LPAR1	0.015479
RNY4P18	0.021983
OR2K2	0.017552
KIAA0368	0.021983
RNA5SP294	0.030959
ZNF483	0.030959
PTGR1	0.030959
LRRC37A5P	0.032964
DNAJC25	0.032964
GNG10	0.032964
C9orf84	0.032964
MIR4668	0.048688
PTBP3	0.031868
RN7SL57P	0.031868
RNA5SP295	0.031868
RN7SL430P	0.039508
HSDL2	0.0245

C9orf147	0.0245
KIAA1958	0.023547
INIP	0.028995
SNX30	0.020613
SLC46A2	0.020613
FAM225A	0.019049
FAM225B	0.019049
ZFP37	0.019049
ZNF883	0.019049
SLC31A2	0.019049
FKBP15	0.019049
SLC31A1	0.019049
CDC26	0.019049
PRPF4	0.019049
RNF183	0.019049
WDR31	0.019049
BSPRY	0.011728
HDHD3	0.011728
ALAD	0.016783
C9orf43	0.016783
POLE3	0.016783
RGS3	0.016783
ZNF618	0.018269
AMBP	0.018269
KIF12	0.018269
COL27A1	0.018269
MIR455	0.018269
ORM1	0.018269
ORM2	0.018269
AKNA	0.018269
DFNB31	0.018269
snoU13|ENSG00000238530.1	0.023487
ATP6V1G1	0.023487
C9orf91	0.023487
TNFSF15	0.028479
TNFSF8	0.024474
TNC	0.02223
1-Dec	0.024474
PAPPA	0.030742
ASTN2	0.008567
TRIM32	0.024408
SNORA70C	0.010309
RN7SKP128	0.010309
RN7SKP125	0.010309
TLR4	0.015088
DBC1	0.013711
MIR147A	0.018857
CDK5RAP2	0.013997
MEGF9	0.015703
FBXW2	0.021448
PSMD5	0.021448
PHF19	0.024277
TRAF1	0.019585
C5	0.021325
CNTRL	0.021325
RAB14	0.019085
RN7SL181P	0.019085
GSN	0.019085

STOM	0.020749
GGTA1P	0.020749
RN7SL187P	0.020749
DAB2IP	0.040664
MIR4478	0.038945
NDUFA8	0.038945
MORN5	0.038945
LHX6	0.038945
RBM18	0.038945
MRRF	0.038945
RN7SL227P	0.038945
PTGS1	0.038945
OR1J1	0.038945
OR1J2	0.038945
OR1J4	0.038945
OR1N1	0.038945
OR1N2	0.038945
OR1L8	0.038945
OR1Q1	0.038945
OR1B1	0.038945
OR1L1	0.038945
OR1L3	0.038945
OR1L4	0.034177
OR1L6	0.034177
OR5C1	0.028751
OR1K1	0.032956
PDCL	0.032956
RC3H2	0.032956
SNORD90	0.032956
ZBTB6	0.032956
ZBTB26	0.038945
RABGAP1	0.030923
GPR21	0.030923
MIR600HG	0.038945
STRBP	0.02831
CRB2	0.020075
DENND1A	0.028608
MIR601	0.02831
LHX2	0.014439
NEK6	0.018418
PSMB7	0.021476
GPR144	0.021476
NR5A1	0.021476
NR6A1	0.021476
RN7SL302P	0.021476
MIR181A2HG	0.021476
MIR181A2	0.021476
MIR181B2	0.021476
OLFML2A	0.021476
WDR38	0.021476
RPL35	0.021476
ARPC5L	0.021476
GOLGA1	0.021476
SCAI	0.01315
PPP6C	0.016056
RABEPK	0.016056
HSPA5	0.016056
GAPVD1	0.016056

MAPKAP1	0.020021
RN7SL30P	0.016056
PBX3	0.03948
MVB12B	0.011488
NRON	0.014442
SNORD116|ENSG00000252985.1	0.014442
LMX1B	0.014442
ZBTB43	0.01315
ZBTB34	0.024443
RALGPS1	0.01997
ANGPTL2	0.024443
GARNL3	0.019961
SLC2A8	0.024443
ZNF79	0.024443
RPL12	0.024443
SNORA65	0.024443
LRSAM1	0.024443
FAM129B	0.01395
STXBP1	0.01395
MIR3911	0.01395
PTRH1	0.01395
C9orf117	0.01395
TTC16	0.01395
TOR2A	0.01395
SH2D3C	0.01395
CDK9	0.01395
MIR3960	0.01395
FPGS	0.01395
ENG	0.012067
RNA5SP296	0.012067
AK1	0.012067
MIR4672	0.012067
ST6GALNAC6	0.006248
ST6GALNAC4	0.01395
PIP5KL1	0.01395
DPM2	0.01395
FAM102A	0.015466
NAIF1	0.027361
SLC25A25	0.022378
PTGES2	0.019273
LCN2	0.019273
C9orf16	0.019273
CIZ1	0.015795
DNM1	0.009178
MIR199B	0.009178
MIR3154	0.009178
GOLGA2	0.009178
SWI5	0.015795
TRUB2	0.015795
COQ4	0.015795
SLC27A4	0.015795
URM1	0.015795
CERCAM	0.011201
ODF2	0.009536
GLE1	0.013919
SPTAN1	0.009536
WDR34	0.009536
HMGA1P4	0.009583

SET	0.009583
PKN3	0.009583
RN7SL560P	0.009583
ZDHHC12	0.009583
ZER1	0.007982
snoU13|ENSG00000239055.1	0.007982
TBC1D13	0.011654
C9orf114	0.011654
ENDOG	0.011654
CCBL1	0.011654
LRRC8A	0.0186
PHYHD1	0.0186
DOLK	0.0186
NUP188	0.0186
SH3GLB2	0.0186
FAM73B	0.0186
DOLPP1	0.022905
CRAT	0.022905
PPP2R4	0.022905
IER5L	0.033144
C9orf106	0.033144
RN7SL159P	0.026809
LINC00963	0.026809
C9orf50	0.033144
NTMT1	0.033144
ASB6	0.033144
PRRX2	0.033144
PTGES	0.033144
TOR1B	0.040062
TOR1A	0.040062
C9orf78	0.040062
USP20	0.040062
FNBP1	0.040062
GPR107	0.029883
NCS1	0.029883
HMCN2	0.038756
RN7SL665P	0.038756
ASS1	0.038756
snoU13|ENSG00000238298.1	0.038756
FUBP3	0.038756
PRDM12	0.038756
EXOSC2	0.038756
ABL1	0.038756
QRFP	0.038756
FIBCD1	0.038756
RAPGEF1	0.04498
SNORA67|ENSG00000212395.1	0.04498
MED27	0.04498
PNPLA7	0.046955
DPH7	0.046955
MRPL41	0.046955
ZMYND19	0.046955
ARRDC1	0.046955
C9orf37	0.046955
EHMT1	0.032572
MIR602	0.046955
CACNA1B	0.024626
FAM157B	0.024626

TUBBP5	0.024626
DIP2C	0.005041
RNA5SP297	0.003547
RNA5SP298	0.003547
TUBB8	0.003547
ZMYND11	0.003547
RN7SL754P	0.005041
MIR5699	0.003227
PRR26	0.003227
LARP4B	0.002953
GTPBP4	0.005447
ADARB2	0.004311
IDI1	0.004941
IDI2	0.004941
LINC00200	0.004941
WDR37	0.004941
LINC00700	0.002765
LINC00701	0.002368
PFKP	0.002196
PITRM1	0.002326
KLF6	0.002326
LINC00702	0.002874
LINC00703	0.002874
LINC00704	0.002326
LINC00705	0.002326
AKR1E2	0.004083
AKR1C1	0.00591
U8|ENSG00000238840.1	0.004353
AKR1C2	0.001992
U8|ENSG00000239148.1	0.001326
AKR1C3	0.002611
U8|ENSG00000251740.1	0.003907
U8|ENSG00000239142.1	0.003147
AKR1CL1	0.002866
AKR1C4	0.005173
U8|ENSG00000251909.1	0.004374
AKR1C7P	0.005511
UCN3	0.005511
TUBAL3	0.00703
NET1	0.00703
CALML5	0.00703
CALML3	0.004882
ASB13	0.006857
RN7SL445P	0.006857
FAM208B	0.006857
GDI2	0.00638
ANKRD16	0.006928
FBXO18	0.003434
IL15RA	0.003434
IL2RA	0.003434
SNORA14	0.003434
RBM17	0.002921
PFKFB3	0.003799
RN7SKP78	0.002301
MIR3155A	0.002301
DKFZP667F0711	0.001998
PRKCQ	0.001679
LINC00707	0.003694

SFMBT2	0.002962
ITIH5	0.001719
ITIH2	0.001719
KIN	0.001719
ATP5C1	0.001485
TAF3	0.00045
GATA3	0.000492
LINC00708	0.000733
RNA5SP299	0.000492
LINC00709	0.000757
SFTA1P	0.000421
LINC00710	0.000621
CELF2	0.000328
USP6NL	0.000213
ECHDC3	0.000214
PROSER2	0.000214
UPF2	0.000143
DHTKD1	0.000331
MIR548AK	0.000331
SEC61A2	0.000273
U6|ENSG00000272507.1	0.000331
snoU13|ENSG00000238900.1	0.000273
NUDT5	0.000222
CDC123	0.000222
RN7SL232P	0.000222
RN7SL198P	0.000222
CAMK1D	0.000434
SNORD45|ENSG00000252438.1	0.000222
RNU6ATAC39P	0.000222
MIR4480	0.000534
MIR548Q	0.000226
CCDC3	0.000226
RNA5SP300	0.000226
OPTN	0.000226
MCM10	0.000226
UCMA	0.000226
PHYH	0.000226
SEPHS1	0.000226
BEND7	0.000227
PRPF18	0.000147
FRMD4A	5.80E-05
RNA5SP301	0.000113
MIR4293	0.000117
MIR1265	0.000151
FAM107B	0.000153
RNA5SP302	5.11E-05
CDNF	6.65E-05
HSPA14	6.65E-05
SUV39H2	6.65E-05
DCLRE1C	6.65E-05
MEIG1	5.75E-05
OLAH	9.17E-05
ACBD7	9.17E-05
C10orf111	9.17E-05
NMT2	9.17E-05
RPP38	9.17E-05
FAM171A1	9.17E-05
ITGA8	0.000223

FAM188A	0.000121
snoU13|ENSG00000239130.1	0.000121
SNORA31|ENSG00000252537.1	0.000121
snoU13|ENSG00000238552.1	0.000121
PTER	0.000121
C1QL3	0.000121
RSU1	9.55E-05
CUBN	0.000148
TRDMT1	0.000148
VIM	0.000148
ST8SIA6	0.000148
PTPLA	0.000148
STAM	8.29E-05
snoR442|ENSG00000251959.1	8.29E-05
MRC1L1	7.31E-05
MRC1	7.31E-05
SLC39A12	7.31E-05
TMEM236|ENSG00000148483.7	7.31E-05
TMEM236|ENSG00000184040.7	7.31E-05
CACNB2	3.62E-05
NSUN6	5.75E-05
ARL5B	5.75E-05
C10orf112	0.000156
U3|ENSG00000200545.1	0.000289
RNA5SP303	0.000289
PLXDC2	0.000336
MIR4675	0.000419
NEBL	0.000302
C10orf113	0.000191
U3|ENSG00000251749.1	0.000236
CASC10	0.000236
MIR1915	0.000236
SKIDA1	0.000236
MLLT10	0.000236
DNAJC1	0.000192
RN7SKP219	0.000192
RN7SKP37	0.000116
EBLN1	0.000143
BMI1	0.000116
COMMD3	0.000116
SPAG6	0.000116
PIP4K2A	9.36E-05
ARMC3	9.01E-05
RNA5SP304	9.01E-05
MSRB2	0.000105
SNORA40|ENSG00000252049.1	0.000105
PTF1A	0.000105
C10orf115	0.000105
C10orf67	0.000105
snoU13|ENSG00000238515.1	0.000105
OTUD1	0.000105
KIAA1217	9.03E-05
MIR603	0.000107
ARHGAP21	0.000131
RNA5SP305	0.000131
PRTFDC1	0.000202
RN7SKP241	0.000202
ENKUR	0.000202

THNSL1	0.000202
GPR158	0.000128
RN7SKP220	0.000141
RNA5SP306	8.93E-05
MYO3A	7.63E-05
GAD2	7.78E-05
APBB1IP	0.000134
RNA5SP307	0.000134
LINC00264	8.82E-05
PDSS1	8.82E-05
ABI1	6.29E-05
SNORA57|ENSG00000223027.1	8.82E-05
snoU13|ENSG00000238414.1	6.29E-05
LINC00614	8.59E-05
ANKRD26	9.48E-05
YME1L1	9.48E-05
MASTL	9.48E-05
ACBD5	9.48E-05
LRRC37A6P	9.48E-05
ARMC4P1	6.13E-05
PTCHD3	6.13E-05
RAB18	9.88E-05
MKX	6.13E-05
ARMC4	8.59E-05
RN7SKP132	6.13E-05
MPP7	8.59E-05
U3|ENSG00000222666.1	4.21E-05
RN7SKP39	4.21E-05
WAC	1.44E-05
RNU4ATAC6P	1.54E-05
BAMBI	1.54E-05
LINC00837	2.57E-05
C10orf126	1.61E-05
RNA5SP308	1.76E-05
LYZL1	4.22E-05
PTCHD3P1	4.83E-05
SVIL	4.44E-05
MIR604	4.44E-05
SNORD115|ENSG00000212411.1	4.44E-05
MIR938	4.44E-05
KIAA1462	4.32E-05
MTPAP	2.40E-05
RN7SL241P	2.40E-05
MAP3K8	2.40E-05
RN7SL63P	2.40E-05
LYZL2	1.57E-05
SVILP1	1.57E-05
ZNF438	8.87E-06
RNA5SP309	3.81E-05
ZEB1	3.69E-05
ARHGAP12	3.50E-05
RN7SL825P	2.12E-05
KIF5B	3.50E-05
EPC1	3.06E-05
CCDC7	3.50E-05
C10orf68	8.43E-06
ITGB1	1.24E-05
RN7SL847P	7.92E-06

NRP1	9.56E-06
RN7SL398P	1.43E-05
LINC00838	6.52E-06
PARD3	9.55E-06
CUL2	8.10E-06
MIR3611	8.89E-06
CREM	4.99E-06
CCNY	8.71E-06
GJD4	1.25E-05
FZD8	2.05E-05
MIR4683	2.05E-05
SNORA40|ENSG00000237002.2	1.86E-05
NAMPTL	1.86E-05
ANKRD30A	7.76E-06
RN7SL314P	4.92E-06
MTRNR2L7	7.50E-06
ZNF248	1.22E-06
ZNF25	1.55E-06
ZNF33A	2.52E-06
ZNF37A	2.92E-06
HSD17B7P2	4.37E-06
SEPT7P9	4.37E-06
CCNYL2	7.52E-06
LINC00839	0.000614
ZNF37BP	0.000441
ZNF33B	0.000385
BMS1	0.000577
snoU13|ENSG00000238732.1	0.000577
MIR5100	0.001017
RET	0.001017
CSGALNACT2	0.001017
RASGEF1A	0.000935
RNU6ATAC11P	0.000697
FXYD4	0.000644
HNRNPF	0.000644
U3|ENSG00000221400.1	0.000644
ZNF487	0.0002
ZNF239	0.000565
ZNF485	0.000831
ZNF32	0.001335
LINC00619	0.000895
LINC00840	0.000895
LINC00841	0.001215
CXCL12	0.000765
TMEM72	0.001465
RASSF4	0.001465
C10orf10	0.001465
C10orf25	0.001465
CEP164P1	0.000655
ZNF22	0.001016
RSU1P2	0.000464
OR13A1	0.000655
ALOX5	0.000281
8-Mar	0.000415
ZFAND4	0.000255
AGAP10	0.000255
AGAP4	0.000255
AGAP9	0.000255

ANTXRLP1	0.000255
ANTXRL	0.000255
ANXA8L1	0.000255
ANXA8L2	0.000255
ANXA8	0.000255
ASAH2C	0.000255
BMS1P1	0.000255
BMS1P2	0.000255
C10orf43	0.000255
FAM21B	0.000255
FAM21C	0.000255
FAM25B	0.000255
FAM25E	0.000255
FAM25G	0.000255
FAM35BP	0.000255
GPRIN2	0.000255
LINC00842	0.000255
NPY4R	0.000255
PTPN20A	0.000255
RBP3	0.000255
RN7SL248P	0.000255
RN7SL453P	0.000255
RNA5SP310	0.000255
RNA5SP311	0.000255
RNA5SP312	0.000255
RNA5SP313	0.000255
SYT15	0.000255
ZNF488	0.000255
GDF2	9.43E-05
GDF10	5.42E-05
BMS1P5	8.07E-06
FAM25C	8.07E-06
FRMPD2	8.07E-06
GLUD1P2	8.07E-06
PTPN20B	8.07E-06
RN7SL527P	8.07E-06
RNA5SP314	8.07E-06
RNA5SP315	8.07E-06
MAPK8	4.05E-05
ARHGAP22	4.05E-05
WDFY4	9.45E-05
LRRC18	9.86E-05
MIR4294	7.05E-05
VSTM4	7.05E-05
FAM170B	7.05E-05
C10orf128	7.05E-05
C10orf71	7.63E-05
DRGX	7.63E-05
ERCC6	7.63E-05
PGBD3	3.34E-05
CHAT	3.34E-05
SLC18A3	3.34E-05
C10orf53	3.34E-05
OGDHL	3.34E-05
PARG	5.68E-05
AGAP7	4.06E-05
AGAP8	4.06E-05
FAM21D	4.06E-05

FAM25D	4.06E-05
MSMB	4.06E-05
RNA5SP316	4.06E-05
SNORA74|ENSG00000223182.1	4.06E-05
TIMM23B	4.06E-05
NCOA4	7.71E-05
AGAP6	0.000101
ASAH2	0.000101
FAM21A	0.000101
LINC00843	0.000101
RMRPP4	0.000101
RNA5SP317	0.000101
SGMS1	0.000101
SNORA74|ENSG00000223111.1	0.000101
TIMM23	0.000101
ASAH2B	0.000144
A1CF	0.000207
PRKG1	0.000296
MIR605	0.000402
CSTF2T	0.000427
DKK1	0.000916
MBL2	0.000544
RNA5SP318	0.000363
PCDH15	0.000529
MIR548F1	0.000808
snoU13|ENSG00000238355.1	0.000279
MTRNR2L5	0.000279
ZWINT	0.000732
SNORD2|ENSG00000238707.1	0.000415
MIR3924	0.000464
IPMK	0.00073
snoU13|ENSG00000238970.1	0.00073
CISD1	0.00073
UBE2D1	0.00073
TFAM	0.00073
BICC1	0.002096
RN7SKP196	0.002036
LINC00844	0.000952
PHYHIPL	0.001388
FAM13C	0.001908
SLC16A9	0.002389
LINC00948	0.001547
CCDC6	0.001404
C10orf40	0.001547
ANK3	0.003868
CDK1	0.005145
RHOBTB1	0.0047
LINC00845	0.0047
TMEM26	0.005118
C10orf107	0.002701
ARID5B	0.000794
RTKN2	0.001506
RN7SL591P	0.001506
ZNF365	0.001506
ADO	0.001506
EGR2	0.001675
NRBF2	0.002186
JMJD1C	0.000709

MIR1296	0.002926
REEP3	0.000528
CTNNA3	0.003813
snoR442|ENSG00000252203.1	0.002825
snoU13|ENSG00000239000.1	0.002825
LRRTM3	0.004689
DNAJC12	0.001135
RN7SL394P	0.001135
SIRT1	0.000941
HERC4	0.000941
RN7SL220P	0.001236
MYPN	0.001135
RN7SKP202	0.001135
ATOH7	0.000941
PBLD	0.000941
HNRNPH3	0.001011
RUFY2	0.001474
DNA2	0.001474
RNA5SP319	0.001474
LINC00849	0.001474
SLC25A16	0.001474
TET1	0.002044
snoU13|ENSG00000251926.1	0.001373
CCAR1	0.001828
SNORD98	0.001373
STOX1	0.001373
DDX50	0.001373
DDX21	0.001373
RN7SL373P	0.001373
KIAA1279	0.000941
SRGN	0.000941
VPS26A	0.000941
SUPV3L1	0.000941
HKDC1	0.000941
HK1	0.000941
TACR2	0.000941
TSPAN15	0.000941
NEUROG3	0.000941
C10orf35	0.000524
COL13A1	0.000898
H2AFY2	0.000908
AIFM2	0.000908
TYSND1	0.000674
SAR1A	0.00044
PPA1	0.00044
NPFFR1	0.00044
LRRC20	0.000614
EIF4EBP2	0.000908
NODAL	0.000908
PALD1	0.000614
PRF1	0.000614
ADAMTS14	0.000614
TBATA	0.000614
SGPL1	0.000614
PCBD1	0.000614
UNC5B	0.000908
SLC29A3	0.000908
snoU13|ENSG00000238918.1	0.000908

CDH23	0.000583
C10orf105	0.000583
C10orf54	0.000583
PSAP	0.000583
CHST3	0.001005
SPOCK2	0.001005
ASCC1	0.001123
SNORA36|ENSG00000200294.1	0.001645
ANAPC16	0.001123
DDIT4	0.000667
DNAJB12	0.001005
MICU1	0.00153
MCU	0.001803
MIR4676	0.00153
OIT3	0.00153
PLA2G12B	0.002032
P4HA1	0.002032
NUDT13	0.002032
SNORA11|ENSG00000221164.1	0.002032
ECD	0.002032
FAM149B1	0.002277
DNAJC9	0.002277
MRPS16	0.002277
TTC18	0.002707
snoU13|ENSG00000238983.1	0.002277
ANXA7	0.002707
MSS51	0.002707
PPP3CB	0.002707
USP54	0.002707
MYOZ1	0.002707
SYNPO2L	0.002707
AGAP5	0.002707
RNA5SP320	0.002707
BMS1P4	0.002707
GLUD1P3	0.002707
SEC24C	0.002707
FUT11	0.002707
RMRPP1	0.002707
CHCHD1	0.002707
ZSWIM8	0.001818
NDST2	0.001818
CAMK2G	0.001818
C10orf55	0.001818
PLAU	0.001818
VCL	0.001818
AP3M1	0.001818
ADK	0.003335
KAT6B	0.003664
DUPD1	0.004067
DUSP13	0.004067
SAMD8	0.004067
VDAC2	0.004067
COMTD1	0.004067
ZNF503	0.001815
MIR606	0.001534
C10orf11	0.000489
RN7SL518P	0.000674
SNORA31|ENSG00000252888.1	0.001226

KCNMA1	0.001658
RNA5SP321	0.0015
DLG5	0.000933
RN7SL284P	0.000685
POLR3A	0.000685
RPS24	0.000685
LINC00856	0.001325
LINC00595	0.0015
SNORA71|ENSG00000201393.1	0.001962
ZMIZ1	0.001962
PPIF	0.001962
ZCCHC24	0.001325
ANXA11	0.001361
EIF5AL1	0.001361
LINC00857	0.001361
MBL1P	0.001361
NUTM2B	0.001361
NUTM2E	0.001361
PLAC9	0.001361
SFTPA1	0.001361
SFTPA2	0.001361
SFTPD	0.001361
TMEM254	0.001361
MAT1A	0.003052
DYDC1	0.003052
DYDC2	0.003052
FAM213A	0.003324
TSPAN14	0.003052
SH2D4B	0.003052
NRG3	0.014876
GHITM	0.024785
C10orf99	0.024785
CDHR1	0.018358
LRIT2	0.018358
LRIT1	0.018358
RGR	0.013097
LINC00858	0.013097
CCSER2	0.008702
RN7SKP84	0.006027
GRID1	0.010254
RN7SKP238	0.006027
RNA5SP322	0.015928
MIR346	0.021002
WAPAL	0.021002
OPN4	0.011094
LDB3	0.011094
U3|ENSG00000252189.1	0.014192
BMPR1A	0.014192
MMRN2	0.009305
SNCG	0.009305
ADIRF	0.009305
AGAP11	0.009305
FAM25A	0.009305
GLUD1	0.009305
FAM35A	0.009305
LINC00863	0.009305
LINC00864	0.008591
NUTM2A	0.009305

NUTM2D	0.009305
RN7SL733P	0.009305
MINPP1	0.008591
PAPSS2	0.008591
ATAD1	0.017254
CFL1P1	0.017254
RN7SL78P	0.019181
KLLN	0.019181
PTEN	0.016889
SNORD74|ENSG00000200891.1	0.016889
RNLS	0.012155
LIPJ	0.012155
LIPF	0.017254
LIPK	0.018791
LIPN	0.018791
LIPM	0.018791
ANKRD22	0.018791
STAMBPL1	0.018815
ACTA2	0.018815
FAS	0.018815
snoU13|ENSG00000238991.1	0.018815
CH25H	0.014805
LIPA	0.010346
IFIT2	0.013258
IFIT3	0.013258
IFIT1B	0.014805
IFIT1	0.014805
IFIT5	0.014805
SLC16A12	0.014805
PANK1	0.005249
MIR107	0.007205
KIF20B	0.007902
LINC00865	0.011139
RN7SKP143	0.011139
HTR7	0.008704
RPP30	0.008704
ANKRD1	0.008704
LINC00502	0.008704
PCGF5	0.020985
HECTD2	0.020985
PPP1R3C	0.020985
TNKS2	0.026338
FGFBP3	0.026338
BTAF1	0.026338
CPEB3	0.017398
SNORA25|ENSG00000252993.1	0.023578
5-Mar	0.015265
HHEX	0.015265
IDE	0.015265
KIF11	0.015265
RN7SL644P	0.015265
EXOC6	0.009894
CYP26C1	0.012813
CYP26A1	0.012813
MYOF	0.012813
CEP55	0.012813
RNA5SP323	0.011464
FFAR4	0.0229

RBP4	0.0229
PDE6C	0.016848
FRA10AC1	0.016848
LGI1	0.0229
SLC35G1	0.015966
PIPSL	0.015966
PLCE1	0.034936
RNY4P26	0.039665
NOC3L	0.022162
TBC1D12	0.022162
HELLS	0.022162
CYP2C18	0.022162
CYP2C19	0.024392
CYP2C9	0.024392
CYP2C8	0.024392
C10orf129	0.024392
PDLIM1	0.019962
SORBS1	0.036143
ALDH18A1	0.022162
TCTN3	0.022162
ENTPD1	0.022162
C10orf131	0.022162
CC2D2B	0.025813
CCNJ	0.022162
MIR3157	0.022162
ZNF518A	0.022162
BLNK	0.022162
DNTT	0.019962
OPALIN	0.036143
TLL2	0.039665
TM9SF3	0.022162
PIK3AP1	0.015966
RNA5SP324	0.022162
MIR607	0.015966
LCOR	0.015966
C10orf12	0.015966
SLIT1	0.015966
ARHGAP19	0.015966
FRAT1	0.015966
FRAT2	0.013585
RRP12	0.013585
PGAM1	0.013585
EXOSC1	0.013585
ZDHHC16	0.013585
MMS19	0.013585
UBTD1	0.015857
ANKRD2	0.015857
HOGA1	0.015857
PI4K2A	0.015857
C10orf62	0.015857
MORN4	0.015857
AVPI1	0.015857
MARVELD1	0.015857
ZFYVE27	0.015857
SFRP5	0.015857
LINC00866	0.022895
GOLGA7B	0.022895
CRTAC1	0.015553

R3HCC1L	0.01075
LOXL4	0.01075
PYROXD2	0.012296
MIR1287	0.012296
HPS1	0.012296
MIR4685	0.012296
HPSE2	0.026062
CNNM1	0.011324
GOT1	0.015928
snoU13|ENSG00000238588.1	0.015928
SLC25A28	0.028142
ENTPD7	0.020826
CUTC	0.011324
COX15	0.011324
ABCC2	0.01036
DNMBP	0.019441
snoU13|ENSG00000238472.1	0.015645
CPN1	0.015645
ERLIN1	0.015645
CHUK	0.015645
CWF19L1	0.013935
SNORA12|ENSG00000212464.1	0.013935
BLOC1S2	0.013935
PKD2L1	0.013935
SCD	0.013935
LINC00263	0.013935
WNT8B	0.009349
SEC31B	0.009349
NDUFB8	0.009349
HIF1AN	0.009349
PAX2	0.00722
FAM178A	0.00722
MIR608	0.00722
MRPL43	0.009349
SEMA4G	0.009349
C10orf2	0.009349
LZTS2	0.009349
PDZD7	0.009349
SFXN3	0.009349
KAZALD1	0.009349
TLX1NB	0.009349
HUG1	0.009349
TLX1	0.009349
LBX1	0.009349
SNORD112|ENSG00000252844.1	0.009349
BTRC	0.007242
snoU13|ENSG00000239091.1	0.009349
DPCD	0.007242
POLL	0.007242
FBXW4	0.007242
FGF8	0.007242
MGEA5	0.007242
NPM3	0.007242
KCNIP2	0.007242
C10orf76	0.007242
HPS6	0.007242
SNORD112|ENSG00000253068.1	0.007242
LDB1	0.007242

PPRC1	0.007242
NOLC1	0.007242
ELOVL3	0.007242
PITX3	0.007242
GBF1	0.00503
NFKB2	0.007242
PSD	0.007242
CUEDC2	0.007242
FBXL15	0.007242
MIR146B	0.007242
C10orf95	0.007242
TMEM180	0.007242
ACTR1A	0.007242
SUFU	0.00494
RN7SL21P	0.007242
TRIM8	0.00494
ARL3	0.00494
SFXN2	0.00403
WBP1L	0.003997
CYP17A1	0.006551
C10ORF32	0.006551
C10orf32	0.006551
AS3MT	0.006551
CNNM2	0.006551
NT5C2	0.006551
INA	0.005937
PCGF6	0.005937
TAF5	0.00412
CALHM1	0.00412
CALHM2	0.00412
MIR1307	0.00412
PDCD11	0.00412
USMG5	0.00412
CALHM3	0.005765
NEURL	0.005765
SH3PXD2A	0.006528
OBFC1	0.006528
SLK	0.005667
RN7SL524P	0.006528
COL17A1	0.005667
MIR936	0.005667
SFR1	0.005667
WDR96	0.008165
MIR609	0.008165
GSTO1	0.008165
GSTO2	0.008165
ITPRIP	0.008165
CCDC147	0.008165
snoU13|ENSG00000238620.1	0.008165
SORCS3	0.010116
SORCS1	0.007072
RNA5SP325	0.007157
RNA5SP326	0.004966
RN7SKP278	0.016917
XPNPEP1	0.033202
U4|ENSG00000272160.1	0.016163
ADD3	0.033202
RN7SL450P	0.033202

MXI1	0.033202
SMNDC1	0.04277
DUSP5	0.04277
SMC3	0.023964
snoU13|ENSG00000239125.1	0.033119
RN7SL686P	0.023964
RBM20	0.023964
RN7SKP288	0.023964
RNA5SP327	0.023964
PDCD4	0.023964
BBIP1	0.021143
MIR4680	0.023964
SHOC2	0.021143
MIR548E	0.021143
ADRA2A	0.021143
GPAM	0.009694
TECTB	0.003629
GUCY2GP	0.004911
ACSL5	0.003408
ZDHHC6	0.003408
VTI1A	0.002932
MIR4295	0.001898
TCF7L2	0.001521
HABP2	0.004341
NRAP	0.004341
CASP7	0.00634
PLEKHS1	0.00634
SNORA17|ENSG00000212589.1	0.005059
DCLRE1A	0.005059
NHLRC2	0.005059
ADRB1	0.00442
C10orf118	0.005581
MIR2110	0.005581
TDRD1	0.005581
VWA2	0.006329
AFAP1L2	0.006329
RN7SL384P	0.006329
ABLIM1	0.005003
snoU13|ENSG00000238577.1	0.006329
FAM160B1	0.005003
TRUB1	0.00096
ATRNL1	0.005753
GFRA1	0.015891
CCDC172	0.022329
PNLIPRP3	0.020793
PNLIP	0.013532
PNLIPRP1	0.013532
PNLIPRP2	0.017264
C10orf82	0.017264
HSPA12A	0.024993
ENO4	0.024993
KIAA1598	0.024993
VAX1	0.017264
MIR3663	0.017264
KCNK18	0.017264
SLC18A2	0.017264
PDZD8	0.017264
EMX2OS	0.017264

EMX2	0.017264
RAB11FIP2	0.018572
CASC2	0.016404
FAM204A	0.018572
LINC00867	0.018572
PRLHR	0.020793
CACUL1	0.015394
U3|ENSG00000251836.1	0.020793
EIF3A	0.016116
NANOS1	0.016116
SNORA19|ENSG00000207468.1	0.016116
SNORA19|ENSG00000222588.1	0.016116
FAM45A	0.016116
SFXN4	0.016116
PRDX3	0.016116
GRK5	0.014487
RN7SL749P	0.016116
MIR4681	0.016116
RGS10	0.014487
TIAL1	0.014487
BAG3	0.014487
INPP5F	0.022277
RN7SL846P	0.022277
MCMBP	0.022277
SEC23IP	0.022277
MIR4682	0.022277
PPAPDC1A	0.01172
C10orf85	0.015692
WDR11	0.013896
RN7SKP167	0.006334
FGFR2	0.007732
ATE1	0.010301
NSMCE4A	0.010301
TACC2	0.010301
BTBD16	0.008565
PLEKHA1	0.016512
MIR3941	0.016512
ARMS2	0.021485
HTRA1	0.031623
DMBT1	0.031623
C10orf120	0.031623
CUZD1	0.031623
FAM24B	0.031623
FAM24A	0.023315
C10orf88	0.016857
PSTK	0.016857
IKZF5	0.016857
ACADSB	0.016857
HMX2	0.020549
HMX3	0.020549
BUB3	0.020549
GPR26	0.015819
CPXM2	0.01312
CHST15	0.015819
OAT	0.044188
LHPP	0.028481
FAM53B	0.01788
METTL10	0.028706

FAM175B	0.03228
ZRANB1	0.03228
CTBP2	0.019404
MIR4296	0.036271
TEX36	0.031611
C10orf137	0.03504
MMP21	0.03504
UROS	0.035298
MIR4484	0.035298
BCCIP	0.035298
DHX32	0.04735
FANK1	0.04735
ADAM12	0.028574
RNA5SP328	0.037734
LINC00601	0.028574
C10orf90	0.044454
SNORD60|ENSG00000199321.1	0.044454
NPS	0.040615
FOXI2	0.040615
CLRN3	0.033318
PTPRE	0.030064
MKI67	0.040615
MGMT	0.048661
EBF3	0.048661
MIR4297	0.048661
LINC00959	0.048661
GLRX3	0.048661
MIR378C	0.048661
PPP2R2D	0.022681
BNIP3	0.017694
JAKMIP3	0.028126
DPYSL4	0.038208
STK32C	0.038208
LRRC27	0.038208
PWWP2B	0.038208
C10orf91	0.028126
INPP5A	0.031607
TTC40	0.031607
GPR123	0.015615
KNDC1	0.027783
UTF1	0.027783
VENTX	0.027783
MIR202	0.027783
ADAM8	0.027783
TUBGCP2	0.015615
ZNF511	0.02241
CALY	0.015615
PRAP1	0.02241
FUOM	0.029936
ECHS1	0.029936
MIR3944	0.029936
PAOX	0.029936
MTG1	0.029936
CYP2E1	0.029936
FRG2B	0.029936
SPRN	0.029936
SYCE1	0.029936
ANO9	7.18E-05

ATHL1	7.18E-05
B4GALNT4	7.18E-05
BET1L	7.18E-05
IFITM1	7.18E-05
IFITM2	7.18E-05
IFITM3	7.18E-05
IFITM5	7.18E-05
NLRP6	7.18E-05
ODF3	7.18E-05
PKP3	7.18E-05
PSMD13	7.18E-05
PTDSS2	0.000108
RIC8A	7.18E-05
RN7SL838P	7.18E-05
SCGB1C1	7.18E-05
SIGIRR	7.18E-05
SIRT3	7.18E-05
RNH1	0.000108
HRAS	0.000108
LRRC56	0.000108
C11orf35	0.000104
RASSF7	0.000104
MIR210HG	0.000104
MIR210	0.000104
PHRF1	8.53E-05
CDHR5	8.53E-05
IRF7	8.53E-05
SCT	8.53E-05
DRD4	8.53E-05
DEAF1	7.17E-05
EPS8L2	7.17E-05
TMEM80	7.17E-05
TALDO1	0.000202
PDDC1	0.000202
CEND1	0.000202
SLC25A22	0.000213
PIDD	0.000213
RPLP2	0.000213
SNORA52	0.000213
PNPLA2	0.000246
CD151	0.000224
EFCAB4A	0.000224
POLR2L	0.000224
TSPAN4	0.000224
CHID1	0.000224
AP2A2	0.000196
MUC6	0.000196
MUC2	0.000246
MUC5AC	0.000246
MUC5B	0.000246
TOLLIP	0.000246
BRSK2	0.000349
MOB2	0.000349
DUSP8	0.000349
CTSD	0.00026
FAM99A	0.00026
FAM99B	0.00026
IFITM10	0.00026

LSP1|ENSG00000130592.9	0.00026
MIR4298	0.00026
SYT8	0.00026
TNNI2	0.00026
C11orf89	0.00026
TNNT3	0.000238
MRPL23	0.00026
H19	0.00022
IGF2	0.000309
MIR483	0.000309
INS	0.000442
MIR4686	0.000442
TH	0.000442
ASCL2	0.000442
C11orf21	0.000756
TSPAN32	0.000756
RPL26P30	0.000619
CD81	0.000619
TRPM5	0.000619
TSSC4	0.000619
KCNQ1	0.000619
KCNQ1OT1	0.000619
KCNQ1DN	0.000619
CDKN1C	0.000619
SLC22A18AS	0.000619
SLC22A18	0.000619
PHLDA2	0.000619
NAP1L4	0.000734
SNORA54	0.000734
CARS	0.000734
OSBPL5	0.000206
MRGPRE	0.000402
MRGPRG	0.000402
ZNF195	0.000471
TSSC2	0.000471
TRPC2	0.00061
ART1	0.00061
ART5	0.00061
CHRNA10	0.00061
NUP98	0.000863
snoU13|ENSG00000238686.1	0.000863
PGAP2	0.000863
RHOG	0.00074
MIR4687	0.000863
STIM1	0.00061
SNORA7|ENSG00000206976.1	0.001645
RRM1	0.001172
OR52B4	0.000826
TRIM21	0.000826
OR52K2	0.00061
OR52K1	0.00061
OR52M1	0.000826
C11orf40	0.000826
OR52I2	0.000826
OR52I1	0.000826
TRIM68	0.000826
OR51D1	0.000826
OR51E1	0.000826

OR51E2	0.000826
OR51C1P	0.000826
MMP26	0.001203
SNORA62|ENSG00000201980.1	0.000826
OR51F1	0.000826
OR52R1	0.000826
OR51F2	0.000826
OR51S1	0.000826
OR51H1P	0.000826
OR51T1	0.000826
OR51A7	0.001022
OR51G2	0.001022
OR51G1	0.001022
OR51A2	0.001022
OR51A4	0.001022
OR51L1	0.000867
OR52J3	0.000867
OR52E2	0.000867
OR52A4	0.000867
OR52A5	0.000867
OR52A1	0.000867
OR51V1	0.000867
HBB	0.000867
HBD	0.000867
HBBP1	0.000867
HBG1	0.000867
HBG2	0.000525
HBE1	0.000525
OR51B4	0.000867
OR51B2	0.000867
OR51B5	0.000867
OR51B6	0.000449
OR51M1	0.000867
OR51J1	0.001022
OR51Q1	0.001022
OR51I1	0.001212
OR51I2	0.001212
OR52D1	0.001212
UBQLN3	0.000862
UBQLNL	0.000862
OR52H1	0.000862
OR52B6	0.000862
TRIM6	0.000862
TRIM34	0.000862
TRIM5	0.000605
TRIM22	0.000605
OR56B1	0.000605
OR52N4	0.000605
OR52N1	0.000605
OR52N5	0.000605
OR52N2	0.000605
OR52E6	0.000605
OR52E4	0.000605
OR52E8	0.000605
OR56A3	0.000605
OR52L1	0.000605
OR56A1	0.000605
OR56A4	0.000605

OR56B4	0.000605
RNA5SP329	0.000605
OR52B2	0.000605
OR52W1	0.000605
C11orf42	0.000605
FAM160A2	0.000605
CNGA4	0.000605
CCKBR	0.000605
PRKCDBP	0.000605
APBB1	0.000605
SMPD1	0.000605
HPX	0.000605
TRIM3	0.000605
ARFIP2	0.000605
TIMM10B	0.000605
DNHD1	0.000605
RRP8	0.000605
ILK	0.000605
TAF10	0.000605
TPP1	0.000605
DCHS1	0.000605
MRPL17	0.000502
GVINP1	0.000399
OR2AG2	0.000502
OR2AG1	0.000502
OR6A2	0.000502
OR10A5	0.000857
OR10A2	0.000857
OR10A4	0.000857
OR2D2	0.000857
OR2D3	0.000857
ZNF215	0.000598
ZNF214	0.00035
NLRP14	0.00035
RBMXL2	0.00035
MIR302E	0.000857
SYT9	0.001243
OLFML1	0.000382
PPFIBP2	0.002385
CYB5R2	0.000262
OVCH2	0.000262
OR10AB1P	0.000262
OR5P2	0.000409
OR5P3	0.000274
OR10A6	0.000274
OR10A3	0.000274
NLRP10	0.000274
EIF3F	0.000274
TUB	0.000274
RIC3	0.000274
LMO1	0.001885
STK33	0.000428
SCARNA20|ENSG00000252778.1	0.000188
TRIM66	0.000188
RPL27A	0.00022
SNORA3|ENSG00000200983.1	0.000188
SNORA45	0.000188
ST5	0.000249

RNA5SP330	0.000255
AKIP1	0.000112
C11orf16	0.000144
ASCL3	0.000144
TMEM9B	0.000142
NRIP3	0.000161
SCUBE2	7.82E-05
MIR5691	0.000122
DENND5A	0.000122
TMEM41B	8.20E-05
IPO7	5.95E-05
SNORA23	5.95E-05
ZNF143	5.21E-05
RN7SL56P	7.55E-05
WEE1	8.49E-05
snoU13|ENSG00000238387.1	7.55E-05
SWAP70	3.29E-05
RN7SKP50	3.68E-05
SBF2	0.000686
ADM	0.000729
AMPD3	0.000729
RNU6ATAC33P	0.000176
MTRNR2L8	0.000138
RNF141	0.000138
LYVE1	0.000138
MRVI1	0.000138
CTR9	0.000165
EIF4G2	0.000165
SNORD97	0.000165
ZBED5	0.000277
GALNT18	0.000429
CSNK2A3	0.000639
MIR4299	0.000614
USP47	0.00084
DKK3	0.00108
MICAL2	0.000581
MICALCL	0.000581
PARVA	0.000581
TEAD1	0.000591
SCARNA16|ENSG00000252329.1	0.000418
LINC00958	0.001135
ARNTL	0.00111
RN7SKP151	0.001176
BTBD10	0.001377
PTH	0.001135
FAR1	0.001176
RNA5SP331	0.000954
SPON1	0.001435
RNA5SP332	0.001404
RRAS2	0.002273
COPB1	0.001436
PSMA1	0.001663
PDE3B	0.002478
CYP2R1	0.002685
CALCB	0.002685
CALCA	0.001252
INSC	0.000726
SOX6	0.0003

RN7SL188P	0.0003
C11orf58	0.000258
PLEKHA7	0.000258
RN7SKP90	0.000258
OR7E14P	0.000258
RPS13	0.000221
SNORD14A	0.000221
SNORD14B	0.000221
PIK3C2A	0.000179
NUCB2	0.000232
NCR3LG1	0.000232
KCNJ11	0.000232
ABCC8	0.000232
USH1C	0.000344
OTOG	0.000344
MYOD1	0.000295
KCNC1	0.000295
SERGEF	0.000221
TPH1	0.000221
SAAL1	0.000352
SAA3P	0.000352
MRGPRX3	0.000352
MRGPRX4	0.000544
SAA4	0.000544
SAA2	0.000544
RNA5SP333	0.000544
RNA5SP334	0.000544
SAA1	0.000544
HPS5	0.000809
GTF2H1	0.000544
MIR3159	0.000544
LDHA	0.000413
LDHC	0.000413
LDHAL6A	0.000352
TSG101	0.000352
UEVLD	0.000352
SPTY2D1	0.000352
TMEM86A	0.000366
IGSF22	0.000366
PTPN5	0.000366
MRGPRX1	0.000251
MRGPRX2	0.000251
ZDHHC13	0.000326
CSRP3	0.000326
E2F8	0.000326
NAV2	0.000265
RNA5SP335	0.000326
MIR4486	0.000333
SNORA1|ENSG00000207407.1	0.000333
MIR4694	0.000333
DBX1	0.000246
HTATIP2	0.000245
PRMT3	0.000308
SLC6A5	0.000196
NELL1	0.000251
RNA5SP336	0.000308
RNA5SP337	0.000381
ANO5	0.000122

SLC17A6	0.000212
FANCF	0.000174
GAS2	0.000259
RNA5SP338	0.000372
SVIP	0.000428
CCDC179	0.000498
LUZP2	0.000812
ANO3	0.000366
MUC15	0.000366
SLC5A12	0.000328
FIBIN	0.000241
BBOX1	0.000363
CCDC34	0.00031
LGR4	0.000297
LIN7C	0.00031
RNA5SP339	0.00031
LINC00678	0.00021
BDNF	0.000235
KIF18A	0.000132
MIR610	0.000108
METTL15	0.000106
RN7SKP158	0.000147
RN7SL240P	0.000164
KCNA4	0.000164
FSHB	0.000134
ARL14EP	8.66E-05
MPPED2	0.000222
DCDC1	0.000547
DNAJC24	0.000419
IMMP1L	0.000677
ELP4	0.000664
PAX6	0.000338
RCN1	0.000895
U3|ENSG00000212551.1	0.000295
WT1	0.000756
EIF3M	0.000291
CCDC73	0.000291
PRRG4	0.000436
QSER1	0.000757
DEPDC7	0.001051
TCP11L1	0.001051
CSTF3	0.000824
HIPK3	0.000509
KIAA1549L	0.000716
C11orf91	0.00093
CD59	0.00093
FBXO3	0.00093
LMO2	0.00093
CAPRIN1	0.00093
NAT10	0.000614
ABTB2	0.00082
CAT	0.00082
ELF5	0.000776
EHF	0.000278
APIP	0.000545
PDHX	0.000813
MIR1343	0.000813
CD44	0.001254

SLC1A2	0.000941
PAMR1	0.000961
FJX1	0.001486
TRIM44	0.001486
LDLRAD3	0.000986
MIR3973	0.00067
COMMD9	0.00067
LINC00610	0.00067
PRR5L	0.000502
TRAF6	0.000502
RAG1	0.000573
RAG2	0.000573
C11orf74	0.000573
SNORA31|ENSG00000251838.1	0.000375
LRRC4C	0.000576
HNRNPKP3	0.000332
API5	0.000244
TTC17	0.000129
RN7SKP287	0.00028
HSD17B12	0.000303
MIR670	0.000255
ALKBH3	0.000815
C11orf96	0.000815
ACCSL	0.000311
ACCS	0.000311
EXT2	0.000532
ALX4	0.000614
CD82	0.001366
TSPAN18	0.000964
TP53I11	0.00139
PRDM11	0.00077
SYT13	0.00077
CHST1	0.000725
SLC35C1	0.000725
CRY2	0.000725
MAPK8IP1	0.001341
C11orf94	0.001341
PEX16	0.001341
GYLTL1B	0.001341
PHF21A	0.000642
CREB3L1	0.000501
DGKZ	0.00037
CHRM4	0.00037
MDK	0.00037
MIR4688	0.00037
AMBRA1	0.000273
HARBI1	0.000643
ATG13	0.000643
ARHGAP1	0.000643
ZNF408	0.000841
F2	0.000324
CKAP5	0.000324
MIR5582	0.000324
SNORD67|ENSG00000252427.1	0.000324
SNORD67|ENSG00000212135.1	0.000324
LRP4	0.000324
C11orf49	0.000462
ARFGAP2	0.000303

PACSIN3	0.000303
RN7SL772P	0.000303
DDB2	0.00022
ACP2	0.00022
NR1H3	0.00022
MADD	0.000148
MYBPC3	0.00022
SPI1	0.00022
MIR4487	0.00022
SLC39A13	0.00022
PSMC3	0.00022
RAPSN	0.000186
CELF1	0.000323
RN7SL652P	0.000468
NDUFS3	0.000468
PTPMT1	0.000468
KBTBD4	0.000468
C1QTNF4	0.000468
FAM180B	0.000468
MTCH2	0.00044
AGBL2	0.00044
FNBP4	0.00044
snoU13|ENSG00000252447.1	0.00044
NUP160	0.00041
RNA5SP340	0.000589
PTPRJ	0.000785
MIR3161	0.000635
OR4B1	0.001007
OR4X2	0.001402
OR4X1	0.001885
OR4S1	0.001885
OR4C3	0.001885
OR4A47	0.001316
OR4C5	0.001316
TRIM49B	0.001524
TRIM64C	0.003452
FOLH1	0.001073
OR4C13	0.001106
OR4C12	0.001106
OR10AG1	0.000199
OR4A15	0.000199
OR4A16	0.000199
OR4A5	0.000199
OR4C11	0.000199
OR4C15	0.000199
OR4C16	0.000199
OR4C46	0.000199
OR4C6	0.000199
OR4P4	0.000199
OR4S2	0.000199
OR5AS1	0.000199
OR5D13	0.000199
OR5D14	0.000199
OR5D16	0.000199
OR5D18	0.000199
OR5F1	0.000199
OR5I1	0.000199
OR5J2	0.000199

OR5L1	0.000199
OR5L2	0.000199
OR5T2	0.000199
OR5W2	0.000199
OR8H2	0.000199
OR8H3	0.000199
OR8I2	0.000199
OR8J3	0.000199
OR8K5	0.000199
TRIM48	0.000199
TRIM51HP	0.000199
TRIM51	0.000199
OR5T3	0.002021
OR5T1	0.003882
OR8H1	0.003882
OR8K3	0.009389
OR8K1	0.005974
OR8J1	0.007368
OR8U1	0.005128
OR5R1	0.005128
OR5M9	0.003579
OR5M3	0.003579
OR5M8	0.001284
OR5M11	0.001284
OR5M10	0.001284
OR5M1	0.001284
OR5AP2	0.001097
OR5AR1	0.001097
OR9G1	0.000826
OR9G4	0.000826
OR5G5P	0.000826
OR5AK2	0.002301
LRRC55	0.003713
APLNR	0.002621
TNKS1BP1	0.003863
SSRP1	0.004222
snoU13|ENSG00000238692.1	0.004222
P2RX3	0.004222
PRG3	0.004222
PRG2	0.004222
SLC43A3	0.003255
RNA5SP341	0.003255
RN7SKP259	0.003255
RTN4RL2	0.004239
SLC43A1	0.005419
TIMM10	0.006888
SMTNL1	0.006888
UBE2L6	0.006888
SERPING1	0.005031
MIR130A	0.005031
YPEL4	0.005031
CLP1	0.005031
ZDHHC5	0.006888
MED19	0.003852
TMX2	0.003852
BTBD18	0.003069
C11orf31	0.003069
CTNND1	0.003069

OR9Q1	0.008536
OR6Q1	0.008536
OR9I1	0.003891
OR9Q2	0.003891
OR1S2	0.003891
OR1S1	0.003891
OR10Q1	0.003891
OR10W1	0.003891
OR5B17	0.002781
OR5B3	0.002781
OR5B2	0.002781
OR5B12	0.002781
OR5B21	0.001429
LPXN	0.00208
ZFP91	0.00208
CNTF	0.001866
GLYAT	0.00331
GLYATL2	0.00331
GLYATL1P2	0.001497
GLYATL1	0.001497
FAM111B	0.003503
FAM111A	0.003503
DTX4	0.003852
MPEG1	0.003852
RN7SL42P	0.005188
RN7SL435P	0.002848
OR5AN1	0.002162
OR5A2	0.002162
OR5A1	0.002162
OR4D6	0.001454
OR4D10	0.001454
OR4D11	0.000773
OR4D9	0.000773
OSBP	0.000773
MIR3162	0.001152
PATL1	0.000569
RN7SKP192	0.000817
OR10V1	0.000817
STX3	0.000633
MRPL16	0.000633
GIF	0.000809
TCN1	0.000809
OOSP1	0.000845
PLAC1L	0.000845
MS4A3	0.000845
MS4A2	0.00066
MS4A6A	0.00066
MS4A4E	0.00066
MS4A4A	0.000748
MS4A6E	0.000748
MS4A14	0.000748
MS4A7	0.000748
MS4A5	0.000748
MS4A1	0.000748
MS4A12	0.000748
MS4A13	0.000748
LINC00301	0.000503
MS4A8	0.000307

MS4A18	0.001071
MS4A15	0.000611
MS4A10	0.000611
CCDC86	0.000611
PTGDR2	0.000611
ZP1	0.000611
PRPF19	0.000467
TMEM109	0.000467
TMEM132A	0.000467
SLC15A3	0.000467
CD6	0.000821
CD5	0.000821
VPS37C	0.001381
PGA3	0.001293
PGA4	0.001293
PGA5	0.001293
VWCE	0.000639
DDB1	0.000639
DAK	0.000639
CYB561A3	0.000639
TMEM138	0.000639
TMEM216	0.000399
CPSF7	0.000113
SDHAF2	0.000113
RN7SL23P	0.000113
PPP1R32	0.000113
LRRC10B	0.000113
MIR4488	0.000113
SYT7	0.000113
RPLP0P2	0.000158
DAGLA	0.000119
MYRF	0.000119
TMEM258	0.000119
FADS2	0.000345
FEN1	0.000119
MIR611	0.000119
FADS1	0.000119
MIR1908	0.000119
FADS3	0.000464
RAB3IL1	0.000529
BEST1	0.000874
FTH1	0.000874
INCENP	0.000528
SCGB1D1	0.000528
SCGB2A1	0.000528
SCGB1D2	0.000528
SCGB2A2	0.000528
SCGB1D4	0.000768
ASRGL1	0.000768
SCGB1A1	0.000768
AHNAK	0.001783
EEF1G	0.001378
MIR3654	0.001378
TUT1	0.001378
MTA2	0.001378
EML3	0.001378
ROM1	0.001378
B3GAT3	0.001378

GANAB	0.001378
INTS5	0.001378
C11orf48	0.001775
C11orf83	0.001775
METTL12	0.001775
SNORA57|ENSG00000206597.1	0.001775
UBXN1	0.001775
LRRN4CL	0.001775
BSCL2	0.001775
GNG3	0.001775
HNRNPUL2	0.001775
TTC9C	0.001556
POLR2G	0.001061
ZBTB3	0.001061
TAF6L	0.001061
TMEM223	0.001061
TMEM179B	0.001061
NXF1	0.001061
STX5	0.001061
RN7SL119P	0.001061
WDR74	0.001061
SNHG1	0.001061
SLC3A2	0.001061
CHRM1	0.000862
RN7SL259P	0.000862
SLC22A6	0.000862
SLC22A8	0.000862
SLC22A24	0.000862
SLC22A10	0.000701
SLC22A25	0.000463
SLC22A9	0.00061
HRASLS5	0.000345
LGALS12	0.000433
RARRES3	0.000433
HRASLS2	0.000433
PLA2G16	0.000433
ATL3	0.000433
RTN3	0.000476
C11orf95	0.000476
RN7SL596P	0.000396
C11orf84	0.000396
MARK2	0.000611
RCOR2	0.000838
NAA40	0.000838
COX8A	0.000838
OTUB1	0.000838
MACROD1	0.000484
FLRT1	0.00057
STIP1	0.000407
FERMT3	0.000407
TRPT1	0.000495
NUDT22	0.000495
DNAJC4	0.000495
VEGFB	0.000495
FKBP2	0.000509
PLCB3	0.00035
PPP1R14B	0.000509
BAD	0.000187

GPR137	0.000187
KCNK4	0.000187
TEX40	0.000179
ESRRA	0.000179
PRDX5	0.000179
TRMT112	0.000179
CCDC88B	0.000165
RPS6KA4	0.000231
MIR1237	0.000231
SLC22A11	0.000395
SLC22A12	0.000395
NRXN2	0.000568
RASGRP2	0.000568
PYGM	0.000401
SF1	0.000401
MAP4K2	0.000387
MEN1	0.000348
CDC42BPG	0.000208
AP5B1	0.000164
ARL2	0.000164
ATG2A	0.000164
BATF2	0.000164
C11orf85	0.000164
CAPN1	0.000164
CCDC85B	0.000164
CDC42EP2	0.000164
CDCA5	0.000164
CFL1	0.000164
CTSW	0.000164
DPF2	0.000164
EFEMP2	0.000164
EHBP1L1	0.000164
EHD1	0.000164
FAM89B	0.000164
FAU	0.000164
FIBP	0.000164
FOSL1	0.000164
FRMD8	0.000164
GPHA2	0.000164
KAT5	0.000164
KCNK7	0.000164
LTBP3	0.000164
MALAT1	0.000164
MAP3K11	0.000164
MIR192	0.000164
MIR4489	0.000164
MIR4690	0.000164
MRPL49	0.000164
MUS81	0.000164
NAALADL1	0.000164
NEAT1	0.000164
OVOL1	0.000164
PCNXL3	0.000164
POLA2	0.000164
PPP2R5B	0.000164
RELA	0.000164
RN7SL114P	0.000164
RN7SL309P	0.000164

RNASEH2C	0.000164
SAC3D1	0.000164
SCYL1	0.000164
SIPA1	0.000164
SLC22A20	0.000164
SLC25A45	0.000164
SNX15	0.000164
SNX32	0.000164
SPDYC	0.000164
SSSCA1	0.000164
SYVN1	0.000164
TIGD3	0.000164
TM7SF2	0.000164
VPS51	0.000164
ZFPL1	0.000164
ZNHIT2	0.000164
C11orf68	6.14E-05
DRAP1	6.14E-05
TSGA10IP	3.34E-05
SART1	2.43E-05
EIF1AD	1.40E-05
BANF1	1.62E-05
CST6	1.62E-05
CATSPER1	1.62E-05
GAL3ST3	1.62E-05
SF3B2	1.39E-05
snoU13|ENSG00000238752.1	1.39E-05
PACS1	1.64E-05
snoU13|ENSG00000238763.1	1.26E-05
KLC2	1.15E-05
RAB1B	1.15E-05
CNIH2	1.15E-05
YIF1A	7.83E-06
TMEM151A	7.83E-06
CD248	7.47E-06
RIN1	7.47E-06
BRMS1	7.47E-06
B3GNT1	7.47E-06
SLC29A2	1.28E-05
NPAS4	1.52E-05
SNORA43|ENSG00000201733.1	1.52E-05
MRPL11	9.19E-06
PELI3	1.52E-05
DPP3	1.52E-05
BBS1	2.18E-05
ZDHHC24	1.36E-05
ACTN3	2.23E-05
CTSF	1.82E-05
CCDC87	1.58E-05
CCS	1.58E-05
RBM14	1.58E-05
RBM4	1.58E-05
RBM4B	1.91E-05
SPTBN2	2.14E-05
RN7SL12P	2.40E-05
C11orf80	3.76E-05
PC	3.77E-05
RCE1	3.77E-05

LRFN4	3.77E-05
MIR3163	2.28E-05
C11orf86	2.28E-05
U3|ENSG00000252709.1	1.26E-05
SYT12	1.26E-05
RHOD	1.32E-05
KDM2A	1.26E-05
snoU13|ENSG00000238892.1	1.07E-05
ADRBK1	1.07E-05
ANKRD13D	1.07E-05
SSH3	1.07E-05
POLD4	6.51E-06
RN7SKP239	6.51E-06
CLCF1	6.51E-06
RAD9A	8.46E-06
PPP1CA	8.46E-06
TBC1D10C	8.46E-06
CARNS1	8.46E-06
RPS6KB2	8.46E-06
CORO1B	8.46E-06
PTPRCAP	8.46E-06
CABP4	8.46E-06
GPR152	8.46E-06
TMEM134	8.46E-06
AIP	8.46E-06
PITPNM1	8.46E-06
CDK2AP2	8.46E-06
CABP2	7.65E-06
ACY3	5.02E-06
ALDH3B2	5.02E-06
C11orf72	5.02E-06
DOC2GP	5.02E-06
FAM86C2P	5.02E-06
GSTP1	5.02E-06
NDUFV1	5.02E-06
NUDT8	5.02E-06
RN7SL59P	5.02E-06
TBX10	5.02E-06
UNC93B1	5.02E-06
ALDH3B1	8.62E-06
NDUFS8	8.62E-06
MIR4691	8.62E-06
TCIRG1	8.62E-06
CHKA	8.62E-06
SUV420H1	4.54E-06
C11orf24	5.18E-06
LRP5	9.12E-06
PPP6R3	7.42E-06
GAL	8.41E-06
MTL5	7.00E-06
CPT1A	2.00E-05
MRPL21	1.07E-05
IGHMBP2	1.14E-05
MRGPRD	1.12E-05
MRGPRF	1.12E-05
TPCN2	8.70E-06
MIR3164	8.70E-06
MYEOV	1.51E-05

CCND1	1.50E-05
ORAOV1	1.50E-05
FGF19	1.50E-05
FGF4	3.08E-05
FGF3	3.08E-05
ANO1	1.29E-05
FADD	1.39E-05
PPFIA1	1.58E-05
MIR548K	1.18E-05
CTTN	2.88E-05
SHANK2	4.30E-05
MIR3664	4.58E-05
DHCR7	1.80E-05
NADSYN1	1.80E-05
UNC93B6	1.80E-05
ENPP7P8	8.35E-06
FAM86C1	8.78E-06
ZNF705E	7.64E-06
DEFB108B	7.64E-06
RNA5SP342	7.64E-06
RNF121	4.78E-06
IL18BP	7.69E-06
NUMA1	7.69E-06
MIR3165	7.56E-06
LRTOMT	7.56E-06
LAMTOR1	7.56E-06
snoU13|ENSG00000238768.1	3.22E-06
ANAPC15	3.22E-06
FOLR3	2.14E-06
FOLR1	4.39E-06
FOLR2	4.39E-06
INPPL1	4.39E-06
PHOX2A	4.39E-06
CLPB	4.36E-06
PDE2A	2.29E-06
MIR139	3.21E-06
ARAP1	1.50E-06
STARD10	1.50E-06
MIR4692	1.50E-06
ATG16L2	1.08E-06
FCHSD2	3.23E-07
P2RY2	8.33E-07
P2RY6	5.39E-07
ARHGEF17	5.08E-07
RELT	7.77E-07
FAM168A	7.98E-07
PLEKHB1	7.77E-07
RAB6A	4.70E-07
MRPL48	5.22E-07
RN7SKP243	1.10E-06
COA4	9.82E-07
PAAF1	8.07E-07
DNAJB13	1.24E-06
UCP2	1.24E-06
UCP3	1.20E-06
C2CD3	8.41E-07
PPME1	3.86E-07
RNA5SP343	3.86E-07

P4HA3	3.86E-07
SNORA7|ENSG00000206913.1	3.86E-07
PGM2L1	1.76E-06
MIR548AL	1.76E-06
KCNE3	1.68E-06
LIPT2	2.14E-06
POLD3	1.68E-06
RN7SKP297	2.92E-06
CHRDL2	3.12E-06
SNORD43|ENSG00000212277.1	2.92E-06
MIR4696	3.12E-06
RNF169	2.07E-06
XRRA1	2.69E-06
RN7SL239P	2.70E-06
SPCS2	2.69E-06
NEU3	2.13E-06
OR2AT4	2.69E-06
SLCO2B1	1.74E-06
TPBGL	5.68E-07
ARRB1	1.34E-06
MIR326	1.71E-06
RPS3	5.56E-07
SNORD15A	5.56E-07
SNORD15B	5.56E-07
KLHL35	5.56E-07
GDPD5	7.00E-07
SERPINH1	1.99E-06
MAP6	1.67E-06
MOGAT2	1.84E-06
RN7SL786P	2.87E-06
DGAT2	2.87E-06
UVRAG	1.16E-06
RNA5SP344	1.89E-06
WNT11	3.46E-06
PRKRIR	7.76E-06
C11orf30	8.02E-06
LRRC32	5.23E-06
GUCY2EP	5.23E-06
TSKU	1.25E-05
ACER3	5.81E-06
B3GNT6	4.86E-06
CAPN5	3.67E-06
OMP	3.67E-06
MYO7A	5.17E-06
GDPD4	2.79E-06
PAK1	2.48E-06
DKFZP434E1119	2.48E-06
CLNS1A	2.48E-06
AQP11	4.08E-06
RSF1	5.06E-06
AAMDC	5.06E-06
INTS4	3.21E-06
KCTD14	2.47E-06
THRSP	4.19E-06
NDUFC2	4.19E-06
ALG8	2.65E-06
KCTD21	2.74E-06
USP35	4.26E-06

GAB2	1.69E-06
NARS2	2.70E-06
TENM4	2.77E-06
MIR708	1.46E-06
MIR5579	1.46E-06
MIR4300	2.65E-07
SNORD112|ENSG00000252592.1	3.57E-06
FAM181B	4.14E-06
PRCP	8.29E-06
snoU13|ENSG00000238995.1	4.43E-06
C11orf82	8.29E-06
RAB30	4.21E-06
SNORA70E	5.99E-06
PCF11	5.99E-06
ANKRD42	5.99E-06
CCDC90B	5.61E-06
DLG2	9.37E-06
TMEM126B	9.97E-07
TMEM126A	9.97E-07
CREBZF	9.97E-07
CCDC89	9.97E-07
SYTL2	6.05E-07
CCDC83	6.05E-07
PICALM	1.06E-06
snoU13|ENSG00000238666.1	1.06E-06
EED	9.88E-07
C11orf73	7.38E-07
RN7SL225P	7.38E-07
CCDC81	7.38E-07
ME3	4.86E-07
PRSS23	5.36E-07
FZD4	5.36E-07
TMEM135	4.29E-07
RAB38	2.37E-06
MIR3166	2.37E-06
CTSC	2.37E-06
GRM5	3.38E-06
TYR	9.62E-06
NOX4	1.04E-05
FOLH1B	9.91E-06
TRIM49C	1.51E-05
TRIM49D1	1.51E-05
TRIM49	1.51E-05
TRIM64B	1.51E-05
TRIM77	1.51E-05
SNORD56|ENSG00000207299.1	9.12E-06
NAALAD2	4.61E-06
CHORDC1	5.92E-06
DISC1FP1	5.92E-06
MIR4490	7.05E-06
MIR1261	7.33E-06
FAT3	5.26E-05
snoU13|ENSG00000239086.1	2.57E-05
MTNR1B	7.43E-05
CCDC67	5.26E-05
SLC36A4	5.26E-05
SMCO4	5.71E-05
RN7SL223P	3.80E-05

KIAA1731	3.01E-05
SCARNA9	3.01E-05
SNORA25|ENSG00000207112.1	3.01E-05
TAF1D	5.41E-05
SNORA1|ENSG00000206834.1	3.01E-05
SNORA32|ENSG00000206799.1	3.01E-05
SNORA8|ENSG00000207304.1	3.01E-05
SNORD6	3.01E-05
MIR1304	3.01E-05
SNORA18|ENSG00000207145.1	3.01E-05
SNORD5|ENSG00000239195.1	3.01E-05
SNORA40|ENSG00000210825.1	3.01E-05
C11orf54	5.41E-05
MED17	2.36E-05
RN7SL195P	2.36E-05
snoU13|ENSG00000238437.1	2.36E-05
VSTM5	2.36E-05
HEPHL1	6.11E-06
PANX1	6.11E-06
FOLR4	6.11E-06
GPR83	7.39E-06
MRE11A	7.39E-06
MIR548L	7.39E-06
ANKRD49	7.39E-06
FUT4	7.39E-06
PIWIL4	8.36E-06
AMOTL1	1.11E-05
CWC15	5.91E-06
KDM4D	5.91E-06
KDM4E	5.91E-06
SRSF8	5.68E-06
ENDOD1	7.97E-06
SESN3	7.51E-06
FAM76B	1.11E-05
CEP57	1.11E-05
MTMR2	1.11E-05
RNA5SP345	1.11E-05
MAML2	1.16E-05
MIR1260B	1.19E-05
CCDC82	1.19E-05
JRKL	1.28E-05
RNA5SP346	1.28E-05
RNA5SP347	9.26E-06
CNTN5	0.000159
RN7SKP53	0.000104
RN7SL222P	0.00012
ARHGAP42	0.000153
RN7SKP115	8.53E-05
snoU13|ENSG00000238388.1	0.000196
TMEM133	0.000196
PGR	0.000157
TRPC6	0.000138
MIR3920	0.000138
ANGPTL5	0.000176
KIAA1377	0.000197
C11orf70	0.000197
snoU13|ENSG00000252679.1	0.000197
snoU13|ENSG00000239154.1	0.000197

YAP1	0.000197
BIRC2	0.000197
BIRC3	0.000197
TMEM123	0.000158
MMP7	0.000125
MMP20	9.81E-05
MMP27	7.11E-05
MMP8	5.54E-05
WTAPP1	1.98E-05
MMP10	3.35E-05
MMP1	1.98E-05
MMP3	1.98E-05
MMP12	1.98E-05
MMP13	3.52E-05
DCUN1D5	2.62E-05
DYNC2H1	2.98E-05
MIR4693	3.05E-05
PDGFD	2.06E-05
DDI1	3.05E-05
RNA5SP348	2.76E-05
CASP12	4.75E-05
CASP4	4.75E-05
CASP5	4.75E-05
CASP1	2.12E-05
CARD16	2.12E-05
CARD17	2.12E-05
CARD18	2.67E-05
GRIA4	2.78E-05
MSANTD4	3.00E-05
KBTBD3	4.32E-05
AASDHPPT	4.32E-05
GUCY1A2	1.74E-05
CWF19L2	3.09E-05
ALKBH8	3.09E-05
ELMOD1	4.01E-05
SLN	5.55E-05
SLC35F2	7.97E-05
CUL5	7.40E-05
RAB39A	7.40E-05
ACAT1	7.76E-05
NPAT	7.76E-05
ATM	0.000136
C11orf65	0.000136
KDELC2	0.000136
EXPH5	0.000136
DDX10	6.52E-05
RNA5SP349	9.04E-05
SNORD39|ENSG00000264997.1	7.76E-05
C11orf87	6.64E-05
ZC3H12C	2.32E-05
RDX	1.79E-05
FDX1	2.99E-05
ARHGAP20	4.77E-05
RNA5SP350	0.000119
C11orf53	6.27E-05
C11orf92	8.42E-05
C11orf93	8.42E-05
MIR4491	6.27E-05

POU2AF1	6.27E-05
BTG4	4.84E-05
C11orf88	5.75E-05
MIR34B	5.75E-05
MIR34C	5.75E-05
LAYN	5.75E-05
SIK2	0.000106
RN7SKP273	5.58E-05
PPP2R1B	0.00016
ALG9	0.00016
FDXACB1	8.02E-05
C11orf1	8.02E-05
CRYAB	8.02E-05
HSPB2	8.02E-05
C11orf52	8.02E-05
DIXDC1	8.02E-05
RNA5SP351	8.02E-05
DLAT	8.02E-05
PIH1D2	0.0001
C11orf57	0.0001
TIMM8B	0.0001
SDHD	0.000171
IL18	8.30E-05
TEX12	8.30E-05
BCO2	8.30E-05
PTS	6.01E-05
C11orf34	8.30E-05
snosnR66	6.41E-05
NCAM1	4.89E-05
TTC12	5.63E-05
ANKK1	9.02E-05
DRD2	9.02E-05
MIR4301	9.02E-05
TMPRSS5	7.07E-05
ZW10	9.72E-05
CLDN25	9.72E-05
ATF4P4	9.72E-05
USP28	9.72E-05
HTR3B	7.84E-05
HTR3A	7.84E-05
ZBTB16	7.26E-05
NNMT	7.26E-05
C11orf71	7.26E-05
RBM7	7.26E-05
REXO2	7.26E-05
NXPE1	7.26E-05
snoU13|ENSG00000238724.1	7.26E-05
NXPE4	7.26E-05
NXPE2	0.000101
ACA59|ENSG00000252870.1	5.01E-05
CADM1	3.50E-05
snoU13|ENSG00000239153.1	3.90E-05
LINC00900	3.90E-05
snoU13|ENSG00000238625.1	5.91E-05
BUD13	4.14E-05
ZNF259	4.14E-05
APOA5	4.14E-05
APOA4	4.14E-05

APOC3	4.14E-05
APOA1	4.14E-05
SIK3	3.19E-05
RNY4P6	4.14E-05
PAFAH1B2	1.52E-05
SIDT2	1.52E-05
TAGLN	1.52E-05
PCSK7	1.52E-05
RNF214	1.52E-05
SCARNA11|ENSG00000252992.1	1.52E-05
BACE1	1.52E-05
CEP164	2.52E-05
DSCAML1	1.08E-05
FXYD2	2.34E-05
FXYD6	2.34E-05
TMPRSS13	2.03E-05
IL10RA	2.03E-05
TMPRSS4	3.85E-05
SCN4B	3.85E-05
SCN2B	3.85E-05
AMICA1	3.85E-05
MPZL3	2.14E-05
MPZL2	2.14E-05
CD3E	2.14E-05
CD3D	2.14E-05
CD3G	3.85E-05
UBE4A	4.83E-05
ATP5L	3.72E-05
RN7SL86P	3.72E-05
KMT2A	2.83E-05
TMEM25	2.01E-05
TTC36	2.01E-05
IFT46	3.32E-05
ARCN1	3.32E-05
PHLDB1	3.32E-05
TREH	3.32E-05
DDX6	2.60E-05
CXCR5	3.42E-05
BCL9L	3.42E-05
MIR4492	3.42E-05
UPK2	3.42E-05
RN7SL688P	3.42E-05
FOXR1	4.43E-05
RN7SL529P	4.43E-05
CCDC84	4.43E-05
RPS25	4.43E-05
MIR3656	4.43E-05
TRAPPC4	4.43E-05
SLC37A4	4.43E-05
HYOU1	4.43E-05
VPS11	4.43E-05
HMBS	3.50E-05
DPAGT1	3.50E-05
H2AFX	3.50E-05
C2CD2L	3.50E-05
HINFP	3.50E-05
ABCG4	4.95E-05
NLRX1	4.95E-05

PDZD3	4.95E-05
CCDC153	4.95E-05
CBL	3.29E-05
MCAM	2.26E-05
ACA64|ENSG00000252119.1	2.26E-05
RNF26	2.26E-05
C1QTNF5	2.26E-05
MFRP	2.26E-05
USP2	1.38E-05
THY1	1.38E-05
PVRL1	1.38E-05
TRIM29	1.92E-05
OAF	1.92E-05
POU2F3	3.40E-05
TMEM136	1.72E-05
ARHGEF12	1.72E-05
GRIK4	1.57E-05
TBCEL	1.72E-05
TECTA	1.72E-05
SC5D	3.35E-05
SORL1	6.30E-05
MIR125B1	0.000134
BLID	0.000108
MIRLET7A2	0.000108
MIR100	0.000108
RNU4ATAC10P	8.52E-05
RNU4ATAC5P	8.52E-05
UBASH3B	6.63E-05
snoU13|ENSG00000239079.1	8.52E-05
CRTAM	6.61E-05
C11orf63	5.38E-05
BSX	8.51E-05
HSPA8	8.51E-05
SNORD14E	8.51E-05
SNORD14C	8.51E-05
SNORD14D	8.51E-05
CLMP	6.03E-05
U8|ENSG00000200496.1	6.61E-05
MIR4493	6.61E-05
GRAMD1B	8.06E-05
SCN3B	8.06E-05
ZNF202	8.06E-05
OR6X1	8.06E-05
OR6M1	8.06E-05
TMEM225	0.000105
OR8D4	8.06E-05
OR4D5	8.06E-05
OR6T1	0.000131
OR10S1	8.06E-05
OR10G4	0.000187
OR10G9	0.000187
OR10G8	0.000187
OR10G7	0.000187
VWA5A	0.000166
OR10D3	0.000166
OR8D1	9.85E-05
OR8D2	9.85E-05
OR8B2	0.000113

OR8B3	0.000113
OR8B4	0.000113
OR8B8	0.000113
OR8B12	6.33E-05
OR8A1	6.33E-05
PANX3	6.33E-05
TBRG1	4.78E-05
SIAE	4.78E-05
RNA5SP352	4.78E-05
SPA17	4.78E-05
NRGN	4.78E-05
VSIG2	4.78E-05
ESAM	4.78E-05
MSANTD2	4.78E-05
ROBO3	5.78E-05
ROBO4	5.78E-05
HEPACAM	5.78E-05
HEPN1	5.78E-05
CCDC15	0.000103
SLC37A2	0.000221
TMEM218	0.000103
KRT18P59	0.000103
PKNOX2	0.000118
FEZ1	0.000162
EI24	0.000117
STT3A	0.000151
CHEK1	0.000151
ACRV1	0.000151
PATE1	0.000151
PATE2	0.000151
PATE3	0.000151
PATE4	9.74E-05
HYLS1	9.74E-05
PUS3	9.74E-05
DDX25	9.74E-05
CDON	6.14E-05
RPUSD4	1.40E-05
FAM118B	1.40E-05
RN7SL351P	1.40E-05
SRPR	1.40E-05
FOXRED1	1.40E-05
TIRAP	5.19E-06
snoU13|ENSG00000238855.1	5.19E-06
DCPS	5.19E-06
ST3GAL4	1.40E-05
KIRREL3	8.73E-06
MIR3167	5.39E-06
RN7SKP121	5.73E-06
RN7SKP279	5.73E-06
ETS1	6.32E-07
FLI1	6.32E-07
KCNJ1	6.32E-07
KCNJ5	6.32E-07
C11orf45	6.32E-07
TP53AIP1	6.32E-07
ARHGAP32	8.18E-07
BARX2	1.79E-06
TMEM45B	2.57E-06

NFRKB	3.66E-06
PRDM10	2.57E-06
LINC00167	2.57E-06
APLP2	5.23E-06
ST14	5.23E-06
ZBTB44	3.32E-06
ADAMTS8	3.32E-06
ADAMTS15	3.32E-06
C11orf44	2.73E-06
SNX19	4.13E-06
RN7SL167P	4.13E-06
NTM	1.35E-06
RNU6ATAC12P	3.77E-06
OPCML	1.07E-05
snoU13|ENSG00000238693.1	0.000115
SPATA19	0.000103
MIR4697	0.000156
IGSF9B	0.000123
JAM3	0.00016
NCAPD3	0.000242
VPS26B	0.000242
THYN1	0.000242
ACAD8	0.000242
B3GAT1	0.000242
GLB1L2	0.000242
GLB1L3	0.000242
B4GALNT3	0.003294
CCDC77	0.003294
FAM138D	0.003294
IQSEC3	0.003294
KDM5A	0.003294
NINJ2	0.003294
RNU4ATAC16P	0.003294
SLC6A12	0.003294
SLC6A13	0.003294
WNK1	0.002623
RAD52	0.004117
ERC1	0.000969
RN7SL852P	0.004117
ADIPOR2	0.000771
FBXL14	0.001235
LINC00942	0.001235
MIR3649	0.001235
WNT5B	0.001235
CACNA2D4	0.000632
LRTM2	0.000632
LINC00940	0.000452
DCP1B	0.000452
CACNA1C	0.00062
CBX3P4	0.000239
FKBP4	0.000239
ITFG2	0.000239
NRIP2	0.000343
FOXM1	0.000343
RHNO1	0.000343
TULP3	0.000343
TEAD4	0.000117
TSPAN9	0.001156

PRMT8	0.000334
EFCAB4B	5.09E-05
PARP11	5.94E-05
CCND2	4.51E-05
C12orf5	7.05E-05
FGF23	9.17E-05
FGF6	8.53E-05
C12orf4	8.53E-05
RAD51AP1	0.000124
DYRK4	0.000124
NDUFA9	0.000124
AKAP3	0.000124
GALNT8	0.000124
KCNA6	9.66E-05
KCNA1	9.66E-05
KCNA5	9.66E-05
NTF3	6.27E-05
ANO2	1.87E-05
VWF	2.50E-05
RN7SL69P	4.53E-05
CD9	2.53E-05
PLEKHG6	2.03E-05
TNFRSF1A	1.67E-05
RN7SL391P	1.67E-05
SCNN1A	2.19E-05
LTBR	2.19E-05
CD27	1.45E-05
TAPBPL	1.45E-05
VAMP1	2.19E-05
MRPL51	2.19E-05
NCAPD2	2.19E-05
SCARNA10	2.19E-05
GAPDH	2.19E-05
IFFO1	2.19E-05
NOP2	2.19E-05
CHD4	1.85E-05
SCARNA11|ENSG00000251898.1	1.85E-05
LPAR5	1.85E-05
ACRBP	1.85E-05
ING4	1.85E-05
ZNF384	1.85E-05
PIANP	2.28E-05
COPS7A	2.28E-05
MLF2	1.51E-05
PTMS	1.51E-05
LAG3	1.51E-05
RN7SL380P	1.51E-05
CD4	1.51E-05
GPR162	1.51E-05
LEPREL2	1.51E-05
GNB3	2.28E-05
CDCA3	2.28E-05
USP5	1.51E-05
SPSB2	1.90E-05
TPI1	1.90E-05
LRRC23	1.90E-05
RPL13P5	1.90E-05
DSTNP2	1.55E-05

ENO2	1.55E-05
ATN1	1.55E-05
C12orf57	1.55E-05
PTPN6	1.55E-05
MIR141	1.55E-05
MIR200C	1.55E-05
PHB2	1.22E-05
SCARNA12	1.55E-05
EMG1	1.22E-05
LPCAT3	1.22E-05
C1S	1.84E-05
C1R	2.24E-05
C1RL	2.24E-05
RBP5	2.24E-05
CLSTN3	2.72E-05
PEX5	2.72E-05
ACSM4	4.05E-05
CD163L1	4.86E-05
CD163	3.35E-05
APOBEC1	2.23E-05
GDF3	2.23E-05
DPPA3	2.23E-05
CLEC4C	0.000115
NANOGNB	2.76E-05
NANOG	2.76E-05
NANOGP1	2.76E-05
SLC2A14	2.76E-05
SLC2A3	2.76E-05
FOXJ2	2.76E-05
C3AR1	4.12E-05
NECAP1	4.12E-05
CLEC4A	2.76E-05
ZNF705A	0.000143
FAM66C	0.000143
FAM86FP	0.000143
FAM90A1	0.000143
LINC00937	0.000143
CLEC6A	0.000112
CLEC4D	0.000112
CLEC4E	0.000112
SCARNA11|ENSG00000252727.1	0.000138
AICDA	8.94E-05
MFAP5	8.94E-05
RIMKLB	0.000172
A2ML1	0.000128
PHC1	0.000128
M6PR	0.000128
KLRG1	0.000106
LINC00612	0.000235
A2M	0.000235
PZP	0.000557
A2MP1	0.000509
LINC00987	0.000509
SNORA75|ENSG00000212432.1	0.000399
SNORA75|ENSG00000212440.1	0.000399
KLRB1	0.000287
CLEC2D	0.000287
CLECL1	0.000399

CD69	0.000399
KLRF1	0.000589
CLEC2B	0.001062
KLRF2	0.000947
CLEC2A	0.000947
CLEC12A	0.000947
CLEC1B	0.000947
CLEC12B	0.000947
CLEC9A	0.000947
CLEC1A	0.000947
RN7SKP161	0.000947
CLEC7A	0.001143
OLR1	0.000947
TMEM52B	0.000947
GABARAPL1	0.000947
KLRD1	0.000899
KLRK1	0.00108
KLRC4	0.00108
KLRC1	0.00108
KLRC2	0.00108
KLRC3	0.00108
EIF2S3L	0.001061
KLRAP1	0.000719
MAGOHB	0.000719
STYK1	0.000944
YBX3	0.00113
TAS2R7	0.001643
TAS2R8	0.001643
TAS2R9	0.001643
PRR4	0.00113
TAS2R10	0.001643
PRH1	0.001365
TAS2R13	0.001643
PRH2	0.001643
TAS2R14	0.00113
TAS2R50	0.00113
TAS2R20	0.00113
TAS2R19	0.00113
TAS2R30	0.00113
TAS2R31	0.00113
TAS2R42	0.00113
TAS2R43	0.00113
TAS2R46	0.00113
TAS2R64P	0.00113
PRB3	0.00113
PRB4	0.00113
PRB1	0.00113
PRB2	0.00113
ETV6	0.00109
BCL2L14	0.001493
LRP6	0.00184
MANSC1	0.00157
LOH12CR2	0.00157
LOH12CR1	0.001818
DUSP16	0.001818
CREBL2	0.001818
GPR19	0.00159
CDKN1B	0.003544

APOLD1	0.003747
MIR613	0.003747
DDX47	0.002527
GPRC5A	0.002392
GPRC5D	0.002392
GSG1	0.002392
HEBP1	0.002392
HTR7P1	0.002392
KIAA1467	0.002392
MIR614	0.002392
SNORD88	0.002392
EMP1	0.003382
C12orf36	0.003382
RNA5SP353	0.003382
GRIN2B	0.004318
RN7SKP162	0.002436
RN7SL46P	0.001896
RN7SL676P	0.003179
RPL30P11	0.004077
ATF7IP	0.004077
PLBD1	0.004077
RN7SKP134	0.00828
GUCY2C	0.008642
H2AFJ	0.003751
HIST4H4	0.003751
WBP11	0.003751
C12orf60	0.001561
SMCO3	0.001498
ART4	0.001498
MGP	0.001561
ERP27	0.000963
ARHGDIB	0.000963
PDE6H	0.001066
RERG	0.002724
PTPRO	0.002039
EPS8	0.002445
STRAP	0.003846
DERA	0.005231
SLC15A5	0.007203
MGST1	0.008385
LMO3	0.006678
RERGL	0.000864
PIK3C2G	0.002119
PLCZ1	0.000456
CAPZA3	0.000456
PLEKHA5	0.001013
RN7SL459P	0.000445
RN7SL67P	0.002163
AEBP2	0.001819
PDE3A	0.002367
SLCO1C1	0.001048
SLCO1B3	0.000934
LST3	0.000934
SLCO1B7	0.000934
SLCO1B1	0.00174
SLCO1A2	0.001632
IAPP	0.001566
PYROXD1	0.001238

RECQL	0.001447
GOLT1B	0.002256
C12orf39	0.002256
GYS2	0.002538
LDHB	0.002216
KCNJ8	0.002549
ABCC9	0.002637
CMAS	0.006313
ST8SIA1	0.003967
C2CD5	0.002573
ETNK1	0.004906
SOX5	0.001065
MIR920	0.00191
LINC00477	0.001086
RN7SL38P	0.002098
BCAT1	0.002409
C12orf77	0.002409
LRMP	0.001738
CASC1	0.00183
LYRM5	0.003854
KRAS	0.003854
IFLTD1	0.009514
RN7SKP262	0.013629
RASSF8	0.010418
BHLHE41	0.008458
SSPN	0.006616
ITPR2	0.003759
RNA5SP354	0.005435
ASUN	0.001434
FGFR1OP2	0.001881
TM7SF3	0.002057
MED21	0.002057
C12orf71	0.002308
STK38L	0.004815
ARNTL2	0.004445
SMCO2	0.00459
PPFIBP1	0.005726
REP15	0.008628
MRPS35	0.008628
MANSC4	0.008628
KLHL42	0.008628
RN7SKP15	0.008628
PTHLH	0.005534
CCDC91	0.002121
RNA5SP355	0.002975
FAR2	0.002131
ERGIC2	0.000498
OVCH1	0.001295
TMTC1	0.000104
U3|ENSG00000253052.1	0.000183
RNA5SP356	0.000335
IPO8	0.000175
CAPRIN2	0.000175
LINC00941	0.000134
TSPAN11	0.000235
DDX11	0.000169
SNORA75|ENSG00000212533.1	0.000235
FAM60A	0.000366

snoU13|ENSG00000238661.1	0.000663
DENND5B	0.000663
snoU13|ENSG00000239033.1	0.000725
METTL20	0.000319
AMN1	0.000211
STMN1P1	0.000344
H3F3C	0.000567
SNORA25|ENSG00000252204.1	0.000505
KIAA1551	0.00042
BICD1	0.000738
FGD4	0.001253
DNM1L	0.003451
YARS2	0.003451
PKP2	0.004436
SNORD112|ENSG00000251863.1	0.006457
ALG10B	0.003753
ALG10	0.003753
CPNE8	0.003418
RNA5SP358	0.003753
RNA5SP359	0.003753
SYT10	0.003753
KIF21A	0.00283
ABCD2	0.001817
C12orf40	0.000942
SLC2A13	0.000942
SNORA22|ENSG00000199571.1	0.001258
LRRK2	0.00054
MUC19	0.000691
CNTN1	0.002082
PDZRN4	0.001744
RNA5SP360	0.000436
GXYLT1	0.000242
YAF2	0.000193
PPHLN1	0.000344
RN7SL10P	0.000193
ZCRB1	0.000134
SNORA74|ENSG00000252917.1	4.67E-05
PRICKLE1	0.00024
ADAMTS20	0.000197
PUS7L	0.000317
IRAK4	0.000127
TWF1	0.000127
TMEM117	0.000144
NELL2	0.000654
DBX2	0.000903
RNA5SP361	0.000903
PLEKHA8P1	0.000359
ANO6	0.000359
ARID2	0.001343
RN7SL246P	0.000833
SCAF11	0.001417
SLC38A1	0.000907
SLC38A2	0.000907
SLC38A4	0.001106
AMIGO2	0.001482
PCED1B	0.001482
MIR4698	0.001362
SNORA64|ENSG00000199566.1	0.000531

MIR4494	0.000531
RPAP3	0.000905
ENDOU	0.000761
RAPGEF3	0.000761
SLC48A1	0.000666
HDAC7	0.000578
VDR	0.000396
TMEM106C	0.000631
COL2A1	0.000631
SENP1	0.000283
PFKM	0.000283
ASB8	0.000303
C12orf68	0.000458
DKFZP779L1853	0.000458
OR10AD1	0.000458
H1FNT	0.000543
ZNF641	0.000543
ANP32D	0.000478
C12orf54	0.000605
OR8S1	0.000605
LALBA	0.000513
KANSL2	0.000541
SNORA34	0.000541
SNORA2A	0.000541
SNORA2B	0.000541
CCNT1	0.000421
LINC00935	0.000357
ADCY6	0.000424
MIR4701	0.000424
CACNB3	0.00029
DDX23	0.000346
RND1	0.000346
ARF3	0.000346
CCDC65	0.000346
FKBP11	0.000346
snoU13|ENSG00000238395.1	0.000554
WNT10B	0.000436
WNT1	0.000436
DDN	0.000436
PRKAG1	0.000436
KMT2D	0.000436
RHEBL1	0.000436
DHH	0.000436
LMBR1L	0.000436
TUBA1B	0.000552
TUBA1A	0.000552
TUBA1C	0.000552
PRPH	0.000344
C1QL4	0.000228
TROAP	0.000228
DNAJC22	0.000228
SPATS2	0.000228
KCNH3	0.000228
MCRS1	0.000228
PRPF40B	0.000324
FAM186B	0.000228
FMNL3	0.000271
TMBIM6	0.000415

NCKAP5L	0.000415
BCDIN3D	0.000415
FAIM2	0.000415
AQP2	0.000415
AQP5	0.000415
AQP6	0.000415
RACGAP1	0.000415
ASIC1	0.000152
SMARCD1	0.000152
COX14	0.000258
GPD1	0.000258
CERS5	0.000344
LIMA1	0.0004
MIR1293	0.0004
FAM186A	0.000337
LARP4	0.000337
DIP2B	0.000337
ATF1	0.00029
RN7SL519P	0.000201
TMPRSS12	0.000201
METTL7A	0.000201
HIGD1C	0.00025
SLC11A2	0.00025
U6|ENSG00000272028.1	0.00025
LETMD1	0.000283
CSRNP2	0.000329
TFCP2	0.000269
POU6F1	0.000325
DAZAP2	0.000508
SMAGP	0.000508
BIN2	0.000587
CELA1	0.00087
GALNT6	0.000767
SLC4A8	0.000767
SCN8A	0.000502
ACVRL1	0.000407
ANKRD33	0.000497
ACVR1B	0.000606
GRASP	0.001299
NR4A1	0.000816
C12orf44	0.000816
OR7E47P	0.000816
KRT80	0.000769
C12orf80	0.000769
LINC00592	0.000933
KRT7	0.001151
KRT121P	0.001151
KRT86	0.000933
KRT81	0.000933
KRT83	0.000933
KRT84	0.000933
KRT85	0.000933
KRT82	0.000933
KRT6B	0.000933
KRT6C	0.000933
KRT75	0.000933
KRT6A	0.000764
KRT5	0.000764

KRT71	0.000764
KRT74	0.000631
KRT72	0.000631
KRT73	0.000764
KRT2	0.000764
KRT1	0.000509
KRT77	0.000402
KRT76	0.00025
KRT3	0.00025
KRT4	0.00025
KRT79	0.00025
KRT78	0.00025
KRT8	0.00025
KRT18	0.00025
EIF4B	0.000212
IGFBP6	0.000212
SOAT2	0.000212
SPRYD3	0.000212
TENC1	0.000212
CSAD	0.000129
ZNF740	0.000129
ITGB7	0.000129
RARG	0.000129
MFSD5	0.000129
ESPL1	0.000157
PFDN5	0.000157
C12orf10	0.000157
AAAS	0.000157
SP7	0.000157
SP1	0.000183
AMHR2	0.000183
PCBP2	0.000183
PRR13	0.000183
MAP3K12	0.000157
TARBP2	0.000157
NPFF	0.000157
ATF7	0.000165
ATP5G2	0.000254
CALCOCO1	0.000254
SNORD81|ENSG00000223213.1	0.000415
RN7SKP289	0.000415
HOXC13	0.000646
HOXC12	0.000646
HOTAIR	0.000646
HOXC11	0.000476
HOXC10	0.000476
HOXC5	0.000476
HOXC6	0.000476
MIR196A2	0.000476
HOXC9	0.000476
HOXC8	0.000476
HOXC4	0.000476
MIR615	0.000476
SMUG1	0.000535
CBX5	0.000717
RN7SL390P	0.000717
HNRNPA1	0.000873
NFE2	0.000873

COPZ1	0.000873
MIR148B	0.000873
RN7SL744P	0.000873
GPR84	0.000873
ZNF385A	0.000873
ITGA5	0.000873
GTSF1	0.001012
NCKAP1L	0.000867
PDE1B	0.000726
PPP1R1A	0.000726
GLYCAM1	0.000718
LACRT	0.000579
DCD	0.000579
MUCL1	0.00195
TESPA1	0.00195
NEUROD4	0.002822
OR9K2	0.00181
OR10A7	0.00251
OR6C74	0.004379
OR6C6	0.002679
OR6C1	0.002963
OR6C3	0.002963
OR6C75	0.002963
OR6C65	0.002963
OR6C76	0.002963
OR6C2	0.002963
OR6C70	0.002099
OR6C68	0.001889
OR6C4	0.001889
OR2AP1	0.001889
OR10P1	0.002475
METTL7B	0.002475
ITGA7	0.004546
BLOC1S1	0.004546
RDH5	0.004546
CD63	0.004706
GDF11	0.003119
SARNP	0.002023
DNAJC14	0.002023
ORMDL2	0.002023
TMEM198B	0.002023
MMP19	0.001222
WIBG	0.001488
DGKA	0.00225
PMEL	0.00225
CDK2	0.00225
RAB5B	0.00225
SUOX	0.00225
IKZF4	0.003266
RPS26	0.003266
ERBB3	0.003832
PA2G4	0.003776
RPL41	0.003776
ESYT1	0.003776
ZC3H10	0.003776
MYL6B	0.002554
MYL6	0.002554
SMARCC2	0.002554

RN7SL770P	0.002554
RNF41	0.002058
NABP2	0.002058
SLC39A5	0.002058
ANKRD52	0.002058
COQ10A	0.002058
CS	0.002058
CNPY2	0.002921
PAN2	0.002921
IL23A	0.002921
STAT2	0.002921
APOF	0.002921
TIMELESS	0.000879
MIP	0.000879
SPRYD4	0.00051
GLS2	0.000775
RBMS2	0.000347
BAZ2A	0.000222
ATP5B	0.000442
SNORD59A	0.000442
PTGES3	0.000222
RN7SL809P	0.000222
NACA	0.000257
PRIM1	0.000257
HSD17B6	0.000231
SNORA48|ENSG00000212383.1	8.65E-05
SDR9C7	0.000133
RDH16	8.96E-05
GPR182	6.92E-05
HBCBP	6.92E-05
ZBTB39	6.92E-05
TAC3	6.92E-05
MYO1A	6.92E-05
TMEM194A	6.92E-05
NAB2	6.92E-05
STAT6	3.78E-05
LRP1	5.79E-05
MIR1228	5.79E-05
NXPH4	5.79E-05
SHMT2	6.59E-05
NDUFA4L2	5.60E-05
STAC3	5.60E-05
R3HDM2	1.37E-05
INHBC	1.02E-05
INHBE	1.02E-05
GLI1	1.02E-05
ARHGAP9	4.86E-06
MARS	4.86E-06
RN7SL312P	4.86E-06
DDIT3	4.86E-06
MBD6	4.86E-06
MIR616	4.86E-06
DCTN2	7.79E-06
KIF5A	3.17E-06
PIP4K2C	2.03E-06
DTX3	1.58E-06
ARHGEF25	1.58E-06
B4GALNT1	1.18E-06

SLC26A10	1.58E-06
snoU13|ENSG00000238436.1	1.42E-06
OS9	4.15E-06
AGAP2	6.69E-06
TSPAN31	6.69E-06
CDK4	4.39E-06
9-Mar	7.11E-06
CYP27B1	7.11E-06
METTL1	7.11E-06
METTL21B	7.11E-06
TSFM	7.11E-06
AVIL	7.70E-06
CTDSP2	1.53E-05
MIR26A2	1.53E-05
XRCC6BP1	5.01E-05
RN7SKP65	2.71E-05
LRIG3	7.17E-05
SLC16A7	2.42E-05
SNORA19|ENSG00000251822.1	1.73E-05
FAM19A2	6.61E-06
SNORD112|ENSG00000252883.1	3.75E-06
USP15	3.75E-06
MON2	1.68E-06
C12orf61	2.78E-06
MIRLET7I	2.78E-06
PPM1H	2.08E-05
snoU13|ENSG00000238475.1	3.07E-06
LDHAL6CP	1.52E-05
AVPR1A	1.61E-05
DPY19L2	8.16E-06
TMEM5	6.79E-06
SRGAP1	8.43E-06
C12orf66	5.41E-06
RPS11P6	5.74E-06
C12orf56	5.74E-06
snoU13|ENSG00000238440.1	5.41E-06
XPOT	5.41E-06
TBK1	5.41E-06
SNORD83	7.94E-06
RASSF3	6.27E-06
MIR548C	4.13E-06
GNS	6.27E-06
snoU13|ENSG00000238592.1	6.27E-06
TBC1D30	6.99E-06
RNU6ATAC42P	1.60E-05
WIF1	1.05E-05
LEMD3	7.63E-06
MSRB3	8.82E-06
RPSAP52	3.80E-06
HMGA2	1.35E-06
RNA5SP362	3.29E-06
LLPH	4.54E-06
TMBIM4	4.54E-06
IRAK3	4.54E-06
RN7SKP166	4.21E-06
HELB	4.21E-06
snoU13|ENSG00000238528.1	4.21E-06
GRIP1	5.42E-06

CAND1	2.14E-05
DYRK2	7.17E-06
IFNG	4.29E-06
IL26	7.57E-06
IL22	7.57E-06
MDM1	4.67E-06
RAP1B	4.42E-06
SNORA70G	5.33E-06
NUP107	4.99E-06
SLC35E3	1.10E-05
MDM2	1.07E-05
CPM	1.34E-05
CPSF6	1.05E-05
LYZ	7.09E-06
YEATS4	7.09E-06
RN7SL804P	7.09E-06
FRS2	4.36E-06
CCT2	2.50E-06
LRRC10	2.50E-06
BEST3	2.19E-06
RAB3IP	2.27E-06
MYRFL	6.78E-06
CNOT2	1.75E-05
KCNMB4	1.61E-05
PTPRB	1.61E-05
PTPRR	2.79E-05
TSPAN8	3.55E-06
LGR5	9.08E-06
ZFC3H1	1.46E-05
SNORA17|ENSG00000212461.1	2.37E-05
THAP2	2.62E-05
TMEM19	1.68E-05
RAB21	1.26E-05
TBC1D15	3.81E-05
TPH2	5.49E-05
TRHDE	5.13E-05
U8|ENSG00000201809.1	8.90E-06
ATXN7L3B	4.19E-06
KCNC2	8.01E-06
CAPS2	4.53E-06
GLIPR1L1	4.53E-06
GLIPR1L2	4.53E-06
GLIPR1	5.64E-06
KRR1	1.47E-05
SNORA70|ENSG00000251893.2	3.43E-06
RN7SL734P	1.33E-05
PHLDA1	2.11E-05
NAP1L1	2.11E-05
RN7SKP172	4.90E-05
BBS10	4.90E-05
OSBPL8	3.42E-05
ZDHHC17	4.57E-05
CSRP2	4.57E-05
E2F7	2.12E-05
snoU13|ENSG00000238769.1	2.12E-05
NAV3	8.06E-06
SYT1	4.88E-05
MIR1252	1.17E-05

RN7SL696P	1.90E-05
PAWR	3.27E-05
PPP1R12A	1.11E-05
RNA5SP363	1.80E-05
OTOGL	1.80E-05
RN7SKP261	1.04E-05
PTPRQ	7.78E-06
MYF6	2.92E-06
MYF5	2.92E-06
LIN7A	5.06E-06
MIR617	3.83E-06
ACSS3	5.06E-06
MIR618	5.06E-06
MIR4699	6.82E-06
PPFIA2	3.02E-06
CCDC59	6.18E-06
METTL25	9.23E-06
TMTC2	2.63E-06
SNORA3|ENSG00000221148.1	6.85E-06
SLC6A15	2.55E-05
TSPAN19	1.11E-05
LRRIQ1	1.11E-05
ALX1	1.46E-05
RASSF9	3.25E-05
NTS	3.25E-05
MGAT4C	0.0001
MKRN9P	0.0001
C12orf50	0.0001
C12orf29	0.0001
CEP290	0.000133
RNA5SP364	0.000133
TMTC3	6.28E-05
KITLG	0.000103
DUSP6	6.63E-05
POC1B	6.63E-05
GALNT4	5.47E-05
ATP2B1	5.47E-05
LINC00936	4.37E-05
RNA5SP365	4.37E-05
CCER1	8.38E-05
LINC00615	8.38E-05
EPYC	7.05E-05
KERA	7.05E-05
LUM	7.05E-05
DCN	7.05E-05
C12orf79	3.89E-05
BTG1	7.05E-05
CLLU1OS	8.93E-05
CLLU1	8.93E-05
snoU13|ENSG00000238865.1	8.93E-05
C12orf74	8.93E-05
PLEKHG7	8.93E-05
EEA1	8.93E-05
SNORD74|ENSG00000201502.1	0.000141
snoU13|ENSG00000238361.1	0.000141
snoU13|ENSG00000239073.1	0.000141
NUDT4	0.000211
UBE2N	0.000211

MRPL42	0.000211
RN7SL737P	0.000211
SOCS2	0.000211
CRADD	0.000211
RN7SL630P	0.000211
RN7SKP263	0.000166
PLXNC1	0.000166
CCDC41	0.000166
RN7SL330P	0.000102
RN7SL483P	0.000166
MIR5700	0.000214
TMCC3	0.000214
KRT19P2	0.000429
MIR492	0.000429
NDUFA12	0.000221
NR2C1	0.000188
FGD6	9.49E-05
VEZT	9.80E-05
MIR331	0.000148
MIR3685	0.000148
METAP2	0.000112
USP44	9.80E-05
PGAM1P5	7.22E-05
NTN4	7.22E-05
SNRPF	0.000112
CCDC38	0.000112
AMDHD1	0.000112
HAL	7.22E-05
LTA4H	7.22E-05
RN7SL88P	0.000123
ELK3	7.22E-05
CDK17	8.31E-05
RN7SKP11	8.31E-05
C12orf55	0.000128
C12orf63	0.000159
NEDD1	0.000159
RMST	0.000422
MIR1251	0.000422
MIR135A2	0.000222
MIR4495	0.000222
MIR4303	0.000222
SLC9A7P1	0.000173
TMPO	0.00013
RN7SL179P	0.000173
SLC25A3	9.97E-05
SNORA53	9.97E-05
IKBIP	9.08E-05
APAF1	9.08E-05
ANKS1B	9.18E-05
RNA5SP366	0.000112
FAM71C	8.36E-05
UHRF1BP1L	0.000167
GOLGA2B	0.000167
RN7SL176P	0.000167
ACTR6	0.000167
DEPDC4	0.000167
SCYL2	0.000167
SLC17A8	0.000167

snoU13|ENSG00000238748.1	0.000222
NR1H4	0.000222
GAS2L3	0.000222
ANO4	0.000211
SLC5A8	0.000277
UTP20	0.000277
snoU13|ENSG00000238800.1	0.000277
ARL1	0.000277
RNA5SP367	0.000277
SPIC	0.000277
MYBPC1	0.000356
CHPT1	0.00027
SYCP3	0.00027
GNPTAB	0.00027
RNA5SP368	0.00027
snoU13|ENSG00000238940.1	0.00027
RNA5SP369	0.00027
DRAM1	0.000181
CCDC53	0.00027
NUP37	0.00027
PARPBP	0.00027
PMCH	0.00027
RN7SL793P	0.00027
IGF1	0.00027
LINC00485	0.000329
PAH	0.00027
ASCL1	0.00027
C12orf42	0.000209
STAB2	0.000128
U8|ENSG00000212594.1	0.000209
snoU13|ENSG00000238914.1	0.000178
NT5DC3	0.000169
HSP90B1	0.000169
MIR3652	0.000169
C12orf73	0.000169
TDG	0.000169
GLT8D2	0.000169
HCFC2	0.000169
NFYB	0.000169
RNA5SP370	0.000169
TXNRD1	0.000209
EID3	0.000209
CHST11	0.000118
MIR3922	0.000118
SLC41A2	0.000125
C12orf45	0.000125
ALDH1L2	0.000125
KIAA1033	0.000125
APPL2	0.000125
C12orf75	0.000125
NUAK1	0.000125
CKAP4	0.000125
TCP11L2	0.000125
POLR3B	0.000162
RFX4	0.000201
RIC8B	0.000207
C12orf23	0.000207
MTERFD3	0.000207

CRY1	0.000207
BTBD11	0.000329
SNORD74|ENSG00000200897.1	0.000261
RNA5SP371	0.000261
PWP1	0.000308
PRDM4	0.000408
ASCL4	0.000408
WSCD2	0.000507
CMKLR1	0.000312
FICD	0.000357
SART3	0.000357
ISCU	0.00047
TMEM119	0.00047
SELPLG	0.00047
CORO1C	0.00047
SNORA40|ENSG00000264043.2	0.00047
SSH1	0.00047
MIR619	0.00047
DAO	0.00047
SVOP	0.00047
USP30	0.00047
RNA5SP372	0.00047
ALKBH2	0.00047
UNG	0.00047
ACACB	0.00047
FOXN4	0.00047
MYO1H	0.00047
KCTD10	0.000389
UBE3B	0.000258
MMAB	0.000389
MVK	0.000389
RN7SKP250	0.000389
FAM222A	0.000389
TRPV4	0.000442
MIR4497	0.000468
GLTP	0.000468
RN7SL441P	0.000468
TCHP	0.000468
GIT2	0.000468
ANKRD13A	0.000468
C12orf76	0.000468
IFT81	0.000726
ATP2A2	0.000593
RN7SL769P	0.000527
ANAPC7	0.000527
ARPC3	0.000527
GPN3	0.000527
FAM216A	0.000672
VPS29	0.000672
SNORD50|ENSG00000202335.1	0.000672
RAD9B	0.000672
PPTC7	0.000707
TCTN1	0.000829
HVCN1	0.000829
RN7SL387P	0.000829
PPP1CC	0.000829
CCDC63	0.000829
MYL2	0.000829

CUX2	0.000672
RNA5SP373	0.000672
FAM109A	0.000672
SH2B3	0.000573
ATXN2	0.000546
U7|ENSG00000272215.1	0.000546
BRAP	0.000672
ACAD10	0.000546
ALDH2	0.000546
MAPKAPK5	0.000546
ADAM1A	0.000546
TMEM116	0.000546
ERP29	0.000546
NAA25	0.000361
MIR3657	0.000546
TRAFD1	0.000778
HECTD4	0.000778
RN7SKP71	0.000778
PTPN11	0.001037
RPL6	0.000901
RPH3A	0.00072
OAS1	0.00072
OAS3	0.00072
OAS2	0.00072
DTX1	0.000901
RASAL1	0.000901
CCDC42B	0.000901
DDX54	0.000901
C12orf52	0.000901
IQCD	0.000901
TPCN1	0.000979
SLC24A6	0.000979
PLBD2	0.000757
SDS	0.000757
SDSL	0.000757
LHX5	0.000757
RBM19	0.001158
TBX5	0.001464
RN7SKP216	0.001157
TBX3	0.000912
SNORA27|ENSG00000252459.1	0.000912
RN7SL865P	0.001236
SNORD56|ENSG00000200112.1	0.001236
MED13L	0.000839
MIR620	0.000839
LINC00173	0.000839
MAP1LC3B2	0.000658
C12orf49	0.000658
RNFT2	0.000658
HRK	0.000658
FBXW8	0.000658
TESC	0.000658
FBXO21	0.000999
NOS1	0.000999
KSR2	0.000713
RFC5	0.000555
WSB2	0.000555
VSIG10	0.000555

PEBP1	0.000555
TAOK3	0.000555
SUDS3	0.000658
RNA5SP374	0.000845
SNORA38|ENSG00000201042.1	0.000845
SRRM4	0.000657
RN7SL508P	0.000657
HSPB8	0.000792
CCDC60	0.000311
TMEM233	0.000311
RN7SKP197	0.000311
PRKAB1	0.000311
CIT	0.000369
MIR1178	0.000369
CCDC64	0.000557
snoU13|ENSG00000272464.1	0.000557
RAB35	0.000729
GCN1L1	0.000369
MIR4498	0.000729
RPLP0	0.000369
PXN	0.000306
SIRT4	0.000306
PLA2G1B	0.000393
MSI1	0.000393
RPS27P25	0.000393
COX6A1	0.000604
GATC	0.000754
TRIAP1	0.000604
SRSF9	0.000754
DYNLL1	0.000754
COQ5	0.000604
RNF10	0.000342
POP5	0.000261
CABP1	0.000333
MLEC	0.000333
UNC119B	0.000333
MIR4700	0.000333
ACADS	0.000333
SPPL3	0.000333
HNF1A	0.000349
C12orf43	0.000349
OASL	0.000349
SNORA70|ENSG00000201945.1	0.000289
P2RX7	0.000349
P2RX4	0.000349
CAMKK2	0.000349
ANAPC5	0.000289
RNF34	0.000357
KDM2B	0.000357
ORAI1	0.000357
MORN3	0.000357
TMEM120B	0.000357
RHOF	0.000357
SETD1B	0.000357
HPD	0.000357
PSMD9	0.000357
WDR66	0.000357
BCL7A	0.000289

MLXIP	0.000277
LRRC43	0.000277
IL31	0.000277
B3GNT4	0.000277
DIABLO	0.000277
VPS33A	0.00035
CLIP1	0.000138
ZCCHC8	0.000169
SNORA9|ENSG00000252192.1	0.000169
RSRC2	0.000329
KNTC1	0.000329
HCAR1	0.000473
HCAR2	0.000559
HCAR3	0.000559
DENR	0.000526
CCDC62	0.000526
HIP1R	0.000526
VPS37B	0.000526
ABCB9	0.000414
ARL6IP4	0.000414
OGFOD2	0.000414
PITPNM2	0.000414
MIR4304	0.000414
RN7SL133P	0.000414
MPHOSPH9	0.000414
C12orf65	0.000414
CDK2AP1	0.000414
RNA5SP375	0.000414
SBNO1	0.000671
SETD8	0.000671
RILPL2	0.000522
SNRNP35	0.000522
RILPL1	0.000522
MIR3908	0.000522
TMED2	0.000522
DDX55	0.000522
SNORA9|ENSG00000206897.1	0.000522
EIF2B1	0.000522
ATP6V0A2	0.000522
GTF2H3	0.000522
TCTN2	0.000522
DNAH10	0.000614
CCDC92	0.000975
DNAH10OS	0.000614
ZNF664	0.000993
FAM101A	0.001445
NCOR2	0.000632
SCARB1	0.00125
UBC	0.001853
MIR5188	0.001853
RPL22P19	0.001853
DHX37	0.001853
BRI3BP	0.001238
AACS	0.001915
TMEM132B	0.001423
LINC00939	0.001177
LINC00943	0.001108
LINC00944	0.001102

LINC00507	0.001763
LINC00508	0.001763
MIR4419B	0.002225
MIR3612	0.002225
TMEM132C	0.0035
snoU13|ENSG00000238895.1	0.002225
SLC15A4	0.006364
GLT1D1	0.010684
TMEM132D	0.010891
FZD10	0.012458
PIWIL1	0.012458
RIMBP2	0.011827
RN7SL534P	0.012458
STX2	0.010234
snoU13|ENSG00000238822.1	0.010234
RAN	0.006844
GPR133	0.006844
RNA5SP376	0.012351
RNA5SP377	0.013188
SFSWAP	0.01409
RNA5SP378	0.017377
MMP17	0.017377
ULK1	0.011968
PUS1	0.011968
EP400	0.011968
SNORA49	0.014538
EP400NL	0.01217
DDX51	0.01217
GALNT9	0.01217
NOC4L	0.01217
MUC8	0.015342
FBRSL1	0.012177
ANHX	0.012177
ANKLE2	0.012177
CHFR	0.012177
GOLGA3	0.012177
LRCOL1	0.012177
P2RX2	0.012177
PGAM5	0.012177
POLE	0.012177
PXMP2	0.012177
RNA5SP379	0.012177
RNU4ATAC12P	0.012177
ZNF10	0.012177
ZNF140	0.012177
ZNF268	0.012177
ZNF26	0.012177
ZNF605	0.012177
ZNF84	0.012177
ZNF891	0.012177
LINC00349	0.029545
LINC00387	0.029545
LINC00388	0.029545
PHF2P2	0.026752
LINC00442	0.03776
RNA5SP24	0.03776
TUBA3C	0.026804
LINC00421	0.018236

TPTE2	0.012854
LINC00350	0.01991
MPHOSPH8	0.01991
PSPC1	0.01991
RN7SL166P	0.01991
ZMYM5	0.01991
snoU13|ENSG00000238665.1	0.01991
ZMYM2	0.01345
CRYL1	0.043335
GJB6	0.043335
MIR4499	0.043335
SGCG	0.030075
SACS	0.044747
GPR12	0.035585
USP12	0.02334
FLT1	0.019714
POMP	0.039859
SLC46A3	0.044057
RXFP2	0.048831
EEF1DP3	0.039224
FRY	0.036448
ZAR1L	0.041938
BRCA2	0.036448
N4BP2L1	0.025965
SNORA16|ENSG00000212293.1	0.025965
N4BP2L2	0.011794
PDS5B	0.007124
RNY1P4	0.011313
LINC00423	0.016906
KL	0.016906
STARD13	0.0176
RFC3	0.014788
SNORA25|ENSG00000199196.1	0.019305
LINC00457	0.005251
NBEA	0.016492
MAB21L1	0.027577
LINC00445	0.014683
DCLK1	0.014683
SOHLH2	0.023486
CCDC169	0.023486
SPG20	0.026693
SPG20OS	0.026693
CCNA1	0.026693
SERTM1	0.026693
ARL2BPP3	0.036154
RFXAP	0.030904
SMAD9	0.023757
ALG5	0.024057
EXOSC8	0.024057
SUPT20H	0.024057
CSNK1A1L	0.024057
RN7SKP1	0.010932
POSTN	0.014683
TRPC4	0.014683
RNA5SP26	0.014683
LINC00571	0.014683
UFM1	0.015244
LINC00366	0.026341

FREM2	0.03522
STOML3	0.035026
PROSER1	0.035026
NHLRC3	0.035026
snoU13|ENSG00000238408.1	0.040069
COG6	0.026408
RNY4P14	0.036319
SNORD116|ENSG00000212553.1	0.02024
SLC25A15	0.04694
TPTE2P5	0.04694
SUGT1P3	0.04694
ELF1	0.04694
WBP4	0.046737
MIR3168	0.046737
RN7SL597P	0.046737
KBTBD6	0.046737
snoU13|ENSG00000238651.1	0.046737
KBTBD7	0.046737
MTRF1	0.046737
NAA16	0.023411
RGCC	0.026939
VWA8	0.025672
MIR5006	0.023656
RN7SL515P	0.040993
DNAJC15	0.042196
ENOX1	0.033906
EPSTI1	0.042196
LINC00400	0.042196
CCDC122	0.042196
NUFIP1	0.030507
KIAA1704	0.030507
RN7SL49P	0.030507
RN7SKP3	0.030507
GTF2F2	0.030507
7SK|ENSG00000271818.1	0.030507
KCTD4	0.030507
LRRC63	0.04635
snoU13|ENSG00000238483.1	0.04635
RN7SKP5	0.04635
LINC00563	0.04635
KIAA0226L	0.047479
LRCH1	0.040105
ESD	0.040105
HTR2A	0.040105
LINC00444	0.036994
LINC00562	0.036994
SUCLA2	0.036994
NUDT15	0.049234
MED4	0.043892
LPAR6	0.036557
RCBTB2	0.027591
LINC00462	0.017769
CYSLTR2	0.032404
FNDC3A	0.032484
RNY3P2	0.041756
MLNR	0.029728
CDADC1	0.029728
CAB39L	0.030234

SETDB2	0.026663
PHF11	0.026663
RCBTB1	0.037124
ARL11	0.021329
EBPL	0.021329
KPNA3	0.021329
RNY4P30	0.021329
RNY4P9	0.021329
SPRYD7	0.027638
DLEU1	0.027638
DLEU2	0.027638
DLEU7	0.027638
GUCY1B2	0.019329
KCNRG	0.027638
MIR3613	0.027638
RNA5SP28	0.027638
RNA5SP29	0.027638
RNASEH2B	0.027638
TRIM13	0.027638
LINC00371	0.021316
FAM124A	0.022846
SERPINE3	0.022846
MIR5693	0.022846
INTS6	0.022846
RPS4XP16	0.022846
RN7SL320P	0.022846
MIR4703	0.022846
RNY1P6	0.022846
WDFY2	0.025322
RN7SL413P	0.022846
DHRS12	0.025322
CCDC70	0.023667
ATP7B	0.023667
ALG11	0.016673
UTP14C	0.016673
NEK5	0.01217
NEK3	0.016673
THSD1	0.016245
RNY4P24	0.016245
VPS36	0.016245
CKAP2	0.016245
LINC00345	0.010499
HNRNPA1L2	0.0089
SUGT1	0.012709
LECT1	0.012709
MIR759	0.023022
PCDH8	0.034085
OLFM4	0.037825
RN7SL618P	0.027665
LINC00558	0.020678
LINC00458	0.028808
MIR5007	0.037825
snoU13|ENSG00000238455.1	0.029816
RN7SKP6	0.029816
PRR20A	0.019583
PRR20B	0.019583
PRR20C	0.019583
PRR20D	0.019583

PRR20E	0.019583
PCDH17	0.022617
RNA5SP30	0.022617
RNY4P29	0.041863
DIAPH3	0.01105
RN7SL375P	0.009356
RNY4P28	0.016748
LINC00434	0.010718
TDRD3	0.009041
RNA5SP31	0.008144
LINC00378	0.008144
RNY3P5	0.005131
RNY4P31	0.005131
MIR3169	0.015257
PCDH20	0.015288
LINC00358	0.012474
LINC00459	0.008978
LINC00448	0.002894
LINC00395	0.008438
LINC00355	0.008438
MIR548X2	0.004902
MIR4704	0.004902
PCDH9	0.004443
LINC00364	0.004028
RN7SL761P	0.009146
KLHL1	0.00851
RNY3P10	0.011556
ATXN8OS	0.005586
LINC00348	0.014497
DACH1	0.015876
RNA5SP32	0.018159
SNORD37|ENSG00000212377.1	0.018934
SNORA68|ENSG00000251715.1	0.018934
SNORA9|ENSG00000199282.1	0.018934
MZT1	0.018934
BORA	0.016446
DIS3	0.016446
PIBF1	0.02044
KLF5	0.011003
RNY1P8	0.010476
LINC00393	0.014095
LINC00392	0.014018
KLF12	0.018572
LINC00402	0.016288
RNY1P5	0.016288
LINC00381	0.016288
LINC00347	0.016288
TBC1D4	0.019382
COMMD6	0.019907
UCHL3	0.019907
LMO7	0.032603
C13orf45	0.016638
LINC00561	0.016638
RN7SL571P	0.013614
KCTD12	0.00763
IRG1	0.00763
CLN5	0.008287
FBXL3	0.008287

MYCBP2	0.013167
SCEL	0.014275
RNY3P7	0.014275
MIR3665	0.012265
SLAIN1	0.012265
EDNRB	0.013341
RN7SL810P	0.008185
LINC00446	0.008185
RNY3P3	0.008185
POU4F1	0.009101
RNF219	0.006437
LINC00331	0.008596
RBM26	0.001192
RNA5SP33	0.001045
NDFIP2	0.000926
SPRY2	0.000893
LINC00564	0.000973
PTMAP5	0.001416
SLITRK1	0.000668
LINC00333	0.000721
LINC00351	0.001897
SLITRK6	0.001461
MIR4500HG	0.005687
MIR4500	0.005687
SLITRK5	0.005687
LINC00433	0.012103
LINC00560	0.012103
LINC00440	0.014335
LINC00353	0.014335
SNORD38|ENSG00000200733.1	0.012053
LINC00559	0.009039
RNA5SP34	0.009039
MIR622	0.009039
LINC00410	0.007292
LINC00379	0.004611
MIR17HG	0.002073
GPC5	0.006296
RNU4ATAC3P	0.021092
MIR548AS	0.009412
GPC6	0.001171
SNORD22	0.009285
RNA5SP35	0.01087
DCT	0.007962
TGDS	0.010737
GPR180	0.010737
RNA5SP36	0.015751
RN7SL585P	0.012082
LINC00391	0.012082
SOX21	0.012082
LINC00557	0.019482
ABCC4	0.043017
snoU13|ENSG00000238463.1	0.025163
RNY3P8	0.025163
RNY4P27	0.025163
CLDN10	0.013049
DZIP1	0.008403
DNAJC3	0.008403
snR65|ENSG00000251901.1	0.008403

UGGT2	0.013049
HS6ST3	0.00796
RN7SL164P	0.014207
LINC00359	0.005756
RN7SKP7	0.005756
SNORD112|ENSG00000252154.1	0.005756
OXGR1	0.005756
snoU13|ENSG00000238522.1	0.005756
MBNL2	0.003701
RNA5SP37	0.002473
RAP2A	0.003202
snoU13|ENSG00000238407.1	0.004081
IPO5	0.005214
FARP1	0.018771
RNF113B	0.012509
RN7SKP8	0.012509
MIR3170	0.012509
STK24	0.015297
RN7SL60P	0.011081
SLC15A1	0.008308
DOCK9	0.006073
UBAC2	0.009232
RN7SKP9	0.009232
GPR18	0.009232
GPR183	0.009232
MIR623	0.013871
LINC00449	0.013871
TM9SF2	0.017582
RNY3P6	0.017133
CLYBL	0.015638
MIR4306	0.017582
SNORA25|ENSG00000201245.1	0.015638
snoU13|ENSG00000238305.1	0.015638
ZIC5	0.01061
ZIC2	0.01061
LINC00554	0.01061
PCCA	0.028959
GGACT	0.017852
TMTC4	0.017852
LINC00411	0.022308
NALCN	0.022308
ITGBL1	0.017819
FGF14	0.006175
MIR2681	0.038984
RNY1P2	0.038984
MIR4705	0.038984
LINC00555	0.016658
TPP2	0.01422
METTL21C	0.01422
snoU13|ENSG00000238869.1	0.00578
CCDC168	0.00578
LINC00283	0.008457
TEX30	0.008457
KDELC1	0.008457
BIVM	0.008457
RNY5P8	0.008457
ERCC5	0.008457
METTL21EP	0.008457

SLC10A2	0.008457
DAOA	0.027388
LINC00343	0.027388
LINC00344	0.027388
SNORA25|ENSG00000252550.1	0.03213
RNA5SP38	0.028124
LINC00460	0.03213
EFNB2	0.035986
ARGLU1	0.035986
LINC00551	0.035986
LINC00443	0.035986
FAM155A	0.011957
SNORD31	0.019679
MIR1267	0.014632
LIG4	0.010244
ABHD13	0.010244
TNFSF13B	0.006524
RNA5SP39	0.010244
MYO16	0.013164
LINC00676	0.027521
IRS2	0.027521
RN7SKP10	0.027521
RN7SL783P	0.032488
COL4A1	0.026807
COL4A2	0.014632
snoU13|ENSG00000238629.1	0.021097
RAB20	0.025197
CARKD	0.03558
CARS2	0.03558
ING1	0.03558
LINC00567	0.03558
LINC00346	0.020409
ANKRD10	0.014587
ARHGEF7	0.013973
TEX29	0.020415
LINC00354	0.045338
SNORD44	0.041972
SOX1	0.049959
LINC00403	0.049959
LINC00404	0.049959
SPACA7	0.026993
TUBGCP3	0.024531
C13orf35	0.014986
ATP11A	0.007894
MCF2L	0.011898
F7	0.014408
F10	0.014408
PROZ	0.014408
PCID2	0.014408
CUL4A	0.014408
LAMP1	0.014408
GRTP1	0.014408
ADPRHL1	0.007272
DCUN1D2	0.007272
TMCO3	0.005875
TFDP1	0.01092
ATP4B	0.016581
GAS6	0.017459

GRK1	0.017459
LINC00454	0.017459
TMEM255B	0.017459
LINC00453	0.020839
LINC00452	0.020839
LINC00565	0.020839
RASA3	0.030665
CDC16	0.026287
CHAMP1	0.026287
MIR4502	0.026287
MIR548AR	0.026287
UPF3A	0.026287
OR11G2	0.046701
OR11H6	0.014917
OR11H7	0.014917
OR11H4	0.014917
TTC5	0.011556
CCNB1IP1	0.014865
SNORA79|ENSG00000222489.1	0.014865
SNORD126	0.014865
RPPH1	0.014865
PARP2	0.022411
TEP1	0.027322
RNA5SP382	0.022411
KLHL33	0.022411
OSGEP	0.025832
APEX1	0.025832
TMEM55B	0.025832
PNP	0.025832
RNASE10	0.018122
RNASE9	0.019415
RNASE11	0.018122
RNASE12	0.018122
OR6S1	0.033481
ANG	0.018867
RNASE4	0.018867
EDDM3A	0.018867
EDDM3B	0.018867
RNASE6	0.018867
RNASE1	0.018867
RN7SL189P	0.018867
RNASE2	0.018867
RNASE3	0.018867
METTL17	0.02679
SLC39A2	0.02679
TPPP2	0.035069
RNASE13	0.035069
RNASE7	0.035069
OR5AU1	0.030577
LINC00641	0.034303
HNRNPC	0.034303
RPGRIP1	0.035958
SUPT16H	0.033034
CHD8	0.043132
SNORD9	0.024381
SNORD8	0.024381
RN7SL650P	0.024381
RAB2B	0.034912

TOX4	0.034912
METTL3	0.034912
SALL2	0.034912
OR10G3	0.022382
OR10G2	0.024271
PSMB5	0.046119
PSMB11	0.036458
CDH24	0.036458
ACIN1	0.036458
C14orf119	0.036458
CEBPE	0.042023
SLC7A8	0.042023
DHRS2	0.040845
RN7SKP205	0.038946
LINC00596	0.038946
DHRS4	0.036556
DHRS4L2	0.040386
DHRS4L1	0.038946
LRRC16B	0.038946
CPNE6	0.038946
NRL	0.038946
PCK2	0.038946
DCAF11	0.038946
FITM1	0.024425
EMC9	0.024425
PSME1	0.024425
PSME2	0.024425
RNF31	0.024425
IRF9	0.024425
RNA5SP383	0.024425
REC8	0.020514
IPO4	0.020514
TM9SF1	0.013227
CHMP4A	0.013227
TSSK4	0.013227
MDP1	0.013227
NEDD8	0.013227
GMPR2	0.013227
TINF2	0.013227
TGM1	0.010984
RABGGTA	0.020993
DHRS1	0.016846
NOP9	0.016846
CIDEB	0.028307
LTB4R2	0.028307
LTB4R	0.028307
ADCY4	0.022524
RIPK3	0.022524
NFATC4	0.022524
NYNRIN	0.022524
CBLN3	0.022524
KHNYN	0.024088
SDR39U1	0.024088
CMA1	0.024088
CTSG	0.021216
GZMH	0.021216
GZMB	0.009591
STXBP6	0.016679

SNORD37|ENSG00000212270.1	0.007487
NOVA1	0.025773
MIR4307	0.018858
LINC00645	0.01327
BNIP3P1	0.001919
C14orf23	0.001458
FOXG1	0.001458
PRKD1	0.009796
G2E3	0.03196
SCFD1	0.047809
MIR624	0.048616
HECTD1	0.04769
HEATR5A	0.043315
DTD2	0.04022
GPR33	0.04022
NUBPL	0.045582
EAPP	0.045407
CFL2	0.039652
BAZ1A	0.030031
SRP54	0.02082
FAM177A1	0.02082
PPP2R3C	0.017038
KIAA0391	0.014509
PSMA6	0.008494
NFKBIA	0.008246
INSM2	0.013152
RALGAPA1	0.010758
SNORA31|ENSG00000253059.1	0.01698
snoU13|ENSG00000238718.1	0.01698
BRMS1L	0.016121
LINC00609	0.01045
PTCSC3	0.01045
RN7SKP21	0.005356
MBIP	0.00681
DPPA3P2	0.007244
SFTA3	0.001544
RN7SKP257	0.003156
PAX9	0.002953
SLC25A21	0.001467
MIR4503	0.002477
MIPOL1	0.00249
FOXA1	0.004484
TTC6	0.002688
SNORA42|ENSG00000200385.1	0.010772
LINC00517	0.00799
SSTR1	0.0096
CLEC14A	0.0096
LINC00639	0.00658
SEC23A	0.014194
GEMIN2	0.012442
TRAPPC6B	0.016139
PNN	0.013995
MIA2	0.0195
CTAGE5	0.0195
FBXO33	0.028403
SNORA31|ENSG00000251858.1	0.031102
LRFN5	0.023138
PTGDR	0.039124

PTGER2	0.027166
TXNDC16	0.010877
GPR137C	0.013234
ERO1L	0.013234
PSMC6	0.013234
STYX	0.013234
GNPNAT1	0.013234
RN7SL588P	0.013234
FERMT2	0.013234
DDHD1	0.010495
MIR5580	0.001865
BMP4	0.001865
CDKN3	0.001032
CNIH	0.001012
GMFB	0.001012
CGRRF1	0.001012
SAMD4A	0.001012
GCH1	0.000884
RNU6ATAC9P	0.000884
MIR4308	0.000884
WDHD1	0.001272
SOCS4	0.001272
MAPK1IP1L	0.001272
LGALS3	0.001258
DLGAP5	0.001258
FBXO34	0.000909
ATG14	0.00153
TBPL2	0.002125
KTN1	0.000499
LINC00520	0.000698
RPL13AP3	0.000698
PELI2	0.000989
TMEM260	0.00163
OTX2	0.00041
RN7SL461P	0.00041
EXOC5	0.000293
AP5M1	0.000293
NAA30	0.000185
C14orf105	0.000185
SLC35F4	0.000185
RN7SKP99	0.00034
C14orf37	5.40E-05
RN7SL598P	0.000186
ACTR10	0.000186
PSMA3	0.000101
ARID4A	8.70E-05
TOMM20L	0.000126
TIMM9	0.000126
KIAA0586	0.000108
DACT1	0.000108
DAAM1	0.000193
GPR135	0.001486
L3HYPDH	0.001486
JKAMP	0.001486
CCDC175	0.001486
RTN1	0.000754
MIR5586	0.000867
LRRC9	0.008426

PCNXL4	0.010919
DHRS7	0.010919
PPM1A	0.008734
C14orf39	0.009205
SIX6	0.013126
U3|ENSG00000253014.1	0.011336
SIX1	0.016815
SIX4	0.013126
MNAT1	0.008413
TRMT5	0.005817
SLC38A6	0.00505
PRKCH	0.001427
SNORD112|ENSG00000252380.1	0.002672
TMEM30B	0.002672
HIF1A	0.000991
SNAPC1	0.000991
SYT16	0.001634
LINC00643	0.001429
LINC00644	0.001429
KCNH5	0.001247
RHOJ	0.002247
GPHB5	0.001081
PPP2R5E	0.001081
RN7SL540P	0.001081
SCARNA20|ENSG00000252800.1	0.001081
WDR89	0.001081
U3|ENSG00000200693.1	0.001081
SGPP1	0.000764
SYNE2	0.006138
ESR2	0.003539
MIR548H1	0.004116
MTHFD1	0.005436
ZBTB25	0.01059
AKAP5	0.00573
ZBTB1	0.01059
HSPA2	0.01019
PPP1R36	0.012332
PLEKHG3	0.01233
SPTB	0.005263
CHURC1	0.003361
GPX2	0.003361
RAB15	0.003361
FNTB	0.003296
MAX	0.003296
MIR4706	0.003296
MIR4708	0.009001
FUT8	0.007676
MIR625	0.009001
LINC00238	0.002744
GPHN	0.002504
FAM71D	0.002504
MPP5	0.002266
ATP6V1D	0.002266
EIF2S1	0.002266
PLEK2	0.002266
MIR5694	0.002266
TMEM229B	0.001598
PLEKHH1	0.001216

PIGH	0.001216
ARG2	0.001216
VTI1B	0.001216
RDH11	0.001216
RDH12	0.001216
RN7SL369P	0.001216
ZFYVE26	0.000885
U3|ENSG00000252792.1	0.001216
RN7SL213P	0.001216
RAD51B	0.003176
RN7SL706P	0.00808
RN7SL108P	0.008546
ZFP36L1	0.001999
RN7SL224P	0.001999
ACTN1	0.002378
DCAF5	0.001757
EXD2	0.001757
GALNT16	0.00251
ERH	0.00251
SLC39A9	0.00251
PLEKHD1	0.00251
CCDC177|ENSG00000255994.1	0.00251
CCDC177|ENSG00000267909.1	0.00251
KIAA0247	0.00294
SRSF5	0.00294
SLC10A1	0.004204
SNORA11|ENSG00000221060.1	0.004204
SMOC1	0.007662
SLC8A3	0.004204
ADAM21P1	0.00288
COX16	0.00288
SYNJ2BP	0.00288
ADAM21	0.00288
ADAM20P1	0.00288
ADAM20	0.00288
MED6	0.003978
RN7SL77P	0.00288
TTC9	0.00288
MAP3K9	0.00288
PCNX	0.002352
SIPA1L1	0.001295
SNORD56B	0.001517
RN7SL683P	0.002135
RGS6	0.015724
DPF3	0.012556
snoU13|ENSG00000238972.1	0.013713
DCAF4	0.013713
ZFYVE1	0.013713
RN7SL586P	0.018902
RBM25	0.023018
PSEN1	0.028374
PAPLN	0.020644
NUMB	0.023521
HEATR4	0.01717
C14orf169	0.01717
ACOT1	0.01717
ACOT2	0.01717
ACOT4	0.01717

ACOT6	0.01717
DNAL1	0.01717
PNMA1	0.01717
ELMSAN1	0.014106
MIR4505	0.017264
snoU13|ENSG00000238330.1	0.014106
PTGR2	0.014106
ZNF410	0.014106
FAM161B	0.014106
COQ6	0.014106
ENTPD5	0.011256
CCDC176	0.014106
ALDH6A1	0.014106
LIN52	0.014106
RN7SL530P	0.017264
VSX2	0.017264
ABCD4	0.018308
VRTN	0.018308
SYNDIG1L	0.018308
NPC2	0.015822
MIR4709	0.021785
ISCA2	0.015822
LTBP2	0.012633
AREL1	0.007619
FCF1	0.006283
SNORA7|ENSG00000222604.1	0.006283
YLPM1	0.007353
PROX2	0.007353
DLST	0.007353
RPS6KL1	0.007353
PGF	0.007353
EIF2B2	0.008143
MLH3	0.008143
ACYP1	0.008143
ZC2HC1C	0.008143
NEK9	0.008143
TMED10	0.008143
RNU4ATAC14P	0.008143
FOS	0.011748
JDP2	0.002293
BATF	0.002293
FLVCR2	0.001821
RNA5SP387	0.002293
TTLL5	0.002723
C14orf1	0.001821
IFT43	0.002293
TGFB3	0.002812
GPATCH2L	0.002259
ESRRB	0.002463
RN7SL747P	0.002607
VASH1	0.001861
ANGEL1	0.002705
C14orf166B	0.002705
RN7SKP17	0.002705
RN7SL356P	0.002186
IRF2BPL	0.002151
KIAA1737	0.003134
TMEM63C	0.003866

ZDHHC22	0.003866
SNORA32|ENSG00000201384.1	0.004537
MIR1260A	0.005065
NGB	0.005065
POMT2	0.005065
GSTZ1	0.005065
TMED8	0.005065
RN7SL137P	0.005065
SAMD15	0.013423
NOXRED1	0.013423
FKSG61	0.013423
VIPAS39	0.013423
AHSA1	0.013423
SNORA46|ENSG00000212371.1	0.013423
ISM2	0.010515
SPTLC2	0.010515
RN7SL587P	0.010515
ALKBH1	0.02045
SLIRP	0.02045
SNW1	0.02045
C14orf178	0.02045
ADCK1	0.036232
RNA5SP388	0.033976
NRXN3	0.04021
DIO2	0.047213
CEP128	0.019168
TSHR	0.030805
GTF2A1	0.030805
SNORA79|ENSG00000221303.1	0.030805
STON2	0.020673
SEL1L	0.025169
RNU6ATAC28P	0.034512
RNU3P3	0.041801
LINC00911	0.035238
FLRT2	0.026411
TTC8	0.041485
FOXN3	0.030211
RN7SKP107	0.048472
EFCAB11	0.034701
TDP1	0.02702
KCNK13	0.032578
PSMC1	0.043851
NRDE2	0.043851
CALM1	0.043851
LINC00642	0.0384
TTC7B	0.029553
RPS6KA5	0.029553
C14orf159	0.029553
SNORA11B	0.029553
RN7SL506P	0.029553
GPR68	0.029357
CCDC88C	0.024295
SMEK1	0.03258
CATSPERB	0.03258
U3|ENSG00000200042.1	0.038557
TC2N	0.038557
FBLN5	0.038557
TRIP11	0.038557

ATXN3	0.038557
NDUFB1	0.038557
CPSF2	0.029262
SLC24A4	0.027494
RIN3	0.029458
LGMN	0.029458
GOLGA5	0.029458
CHGA	0.029458
ITPK1	0.021378
MOAP1	0.029458
TMEM251	0.029458
C14orf142	0.029458
UBR7	0.029458
BTBD7	0.01362
UNC79	0.017319
COX8C	0.017319
PRIMA1	0.040883
FAM181A	0.040883
ASB2	0.040883
MIR4506	0.040883
LINC00521	0.040883
OTUB2	0.033007
DDX24	0.033007
IFI27L1	0.033007
IFI27	0.033007
IFI27L2	0.033007
PPP4R4	0.043575
SERPINA6	0.038548
GSC	0.047102
DICER1	0.047102
MIR3173	0.047102
CLMN	0.047102
LINC00341	0.047102
SYNE3	0.0377
SNHG10	0.0377
SCARNA13	0.0377
GLRX5	0.0377
TCL6	0.049014
TCL1B	0.0377
TCL1A	0.0377
LINC00617	0.0377
C14orf132	0.0377
BDKRB2	0.0377
BDKRB1	0.0377
DKFZP434O1614	0.0377
ATG2B	0.040963
snoU13|ENSG00000238776.1	0.0377
GSKIP	0.040963
AK7	0.040963
PAPOLA	0.04288
RN7SKP108	0.04288
TNFAIP8L3	0.0279
CYP19A1	0.041584
MIR4713	0.041584
GLDN	0.041584
DMXL2	0.041584
SCG3	0.034605
LYSMD2	0.034605

TMOD2	0.023356
TMOD3	0.023
U6|ENSG00000272337.1	0.023
LEO1	0.023
MAPK6	0.023
BCL2L10	0.023
GNB5	0.023
MYO5C	0.028253
MIR1266	0.033466
MYO5A	0.034567
RSL24D1	0.037943
RAB27A	0.049649
PIGB	0.049649
CCPG1	0.049649
MIR628	0.049649
C15orf65	0.049649
DYX1C1	0.049649
PYGO1	0.049649
PRTG	0.049649
TEX9	0.039268
MNS1	0.039268
ZNF280D	0.038581
TCF12	0.037888
CGNL1	0.049412
snoU13|ENSG00000239100.1	0.045546
MYO1E	0.019125
LDHAL6B	0.042354
FAM81A	0.026941
snoU13|ENSG00000238767.1	0.026941
RNA5SP396	0.042045
GCNT3	0.042045
GTF2A2	0.042045
TLN2	0.047646
TPM1	0.032579
LACTB	0.032579
RPS27L	0.032579
RAB8B	0.01433
APH1B	0.01433
CA12	0.01433
USP3	0.042945
HERC1	0.038374
MIR422A	0.038374
DAPK2	0.037757
FAM96A	0.039517
SNX1	0.039517
SNX22	0.039517
PPIB	0.039517
OAZ2	0.040298
RBPMS2	0.040298
MIR1272	0.040298
PIF1	0.040298
PLEKHO2	0.040298
RN7SL348P	0.040298
ANKDD1A	0.027076
SPG21	0.019133
MTFMT	0.02823
SLC51B	0.02823
RASL12	0.02823

KBTBD13	0.02823
UBAP1L	0.02823
PDCD7	0.030376
CLPX	0.030376
CILP	0.030376
PARP16	0.042039
SNORA24|ENSG00000206903.1	0.030376
IGDCC3	0.047473
IGDCC4	0.048034
DPP8	0.048229
PTPLAD1	0.035036
snoU13|ENSG00000238715.1	0.034583
VWA9	0.034583
SLC24A1	0.034583
DENND4A	0.047622
snoU13|ENSG00000238311.1	0.034583
MIR4511	0.035378
RAB11A	0.047622
MEGF11	0.047572
MIR4311	0.047622
DIS3L	0.047572
TIPIN	0.047572
SCARNA14	0.047572
MAP2K1	0.047572
SNAPC5	0.036625
MIR4512	0.036625
RPL4	0.036625
SNORD18C	0.036625
SNORD16	0.036625
SNORD18A	0.036625
SNORD18B	0.036625
ZWILCH	0.036625
LCTL	0.036625
SMAD6	0.035092
SMAD3	0.020967
AAGAB	0.020967
IQCH	0.021523
C15orf61	0.022569
MAP2K5	0.015211
SKOR1	0.01673
PIAS1	0.015383
CALML4	0.014881
CLN6	0.014881
FEM1B	0.014881
ITGA11	0.015383
CORO2B	0.015383
ANP32A	0.010454
MIR4312	0.010454
SPESP1	0.015992
NOX5	0.014004
LINC00277	0.019225
GLCE	0.012299
RN7SL438P	0.012299
PAQR5	0.012299
SNORA77|ENSG00000221376.1	0.012299
RNA5SP398	0.012813
KIF23	0.012813
RPLP1	0.012813

U3|ENSG00000207119.1	0.012813
LINC00593	0.024325
TLE3	0.017414
MIR629	0.024325
UACA	0.010499
RPL29P30	0.0054
LARP6	0.0054
LRRC49	0.0054
THAP10	0.0054
THSD4	0.009217
CT62	0.010188
NR2E3	0.00762
MYO9A	0.031509
RNA5SP399	0.00762
SENP8	0.011753
GRAMD2	0.011753
PKM	0.011753
PARP6	0.011753
CELF6	0.011753
HEXA	0.011753
TMEM202	0.015936
ARIH1	0.015936
MIR630	0.015936
GOLGA6B	0.015936
RN7SL485P	0.015936
RN7SL853P	0.015936
HIGD2B	0.015936
BBS4	0.019304
ADPGK	0.019304
NEO1	0.019304
HCN4	0.019304
C15orf60	0.019304
NPTN	0.019304
CD276	0.019304
C15orf59	0.019304
TBC1D21	0.020294
LOXL1	0.020294
STOML1	0.020294
PML	0.031093
GOLGA6A	0.04163
ISLR2	0.04163
RN7SL429P	0.04163
ISLR	0.04163
STRA6	0.04163
CCDC33	0.04163
CYP11A1	0.04163
SNX33	0.035158
CSPG4	0.035158
ODF3L1	0.035158
DNM1P35	0.035158
MIR4313	0.035158
RN7SL319P	0.035158
SNORD112|ENSG00000252372.1	0.035158
UBE2Q2	0.016475
RN7SL510P	0.016475
FBXO22	0.016475
NRG4	0.016475
C15orf27	0.017261

ETFA	0.017261
ISL2	0.017261
SCAPER	0.017261
MIR3713	0.017261
RN7SKP217	0.016475
RCN2	0.01196
RN7SL278P	0.01196
PSTPIP1	0.024163
TSPAN3	0.038733
PEAK1	0.035155
HMG20A	0.047611
MTHFS	0.044479
ST20	0.044479
C15ORF37	0.044479
C15orf37	0.044479
BCL2A1	0.044479
ZFAND6	0.044479
SNORD112|ENSG00000251881.1	0.044479
FAH	0.044479
LINC00927	0.044479
ARNT2	0.044479
ABHD17C	0.045131
KIAA1199	0.033263
MIR549	0.029495
MESDC2	0.029829
MIR4514	0.029829
MESDC1	0.029829
C15orf26	0.029829
IL16	0.03131
STARD5	0.03131
TMC3	0.03131
MEX3B	0.021192
EFTUD1	0.015218
FAM154B	0.022016
CSPG4P8	0.02398
GOLGA6L10|ENSG00000205281.6	0.02398
GOLGA6L10|ENSG00000254374.2	0.02398
GOLGA6L9|ENSG00000196648.6	0.02398
GOLGA6L9|ENSG00000197978.8	0.02398
RN7SL256P	0.02398
RN7SL410P	0.02398
RN7SL61P	0.02398
RPS17	0.02398
UBE2Q2P2	0.02398
UBE2Q2P3	0.02398
CPEB1	0.02398
RPS17L	0.02398
AP3B2	0.023261
SCARNA15|ENSG00000252690.2	0.01712
FSD2	0.01712
WHAMM	0.023261
HOMER2	0.035606
C15orf40	0.035606
FAM103A1	0.035606
BTBD1	0.035436
MIR4515	0.035436
TM6SF1	0.035436
HDGFRP3	0.035436

BNC1	0.035436
ABHD2	0.030277
RLBP1	0.040513
FANCI	0.030277
POLG	0.044184
LINC00925	0.040513
RHCG	0.040942
LINC00928	0.030382
TICRR	0.030382
KIF7	0.030382
PLIN1	0.030382
PEX11A	0.030382
WDR93	0.030382
MESP1	0.030382
MESP2	0.030382
ANPEP	0.027816
AP3S2	0.019878
MIR5094	0.027816
MIR5009	0.027816
C15orf38	0.019878
ZNF710	0.025458
MIR3174	0.026441
IDH2	0.035398
CIB1	0.033284
GDPGP1	0.033284
TTLL13	0.033284
NGRN	0.033284
RN7SL736P	0.033284
GABARAPL3	0.032856
IQGAP1	0.032856
ZNF774	0.032856
CRTC3	0.020957
BLM	0.020238
SNORD18|ENSG00000200677.1	0.019259
RN7SL363P	0.020238
FURIN	0.027978
FES	0.027978
MAN2A2	0.029059
HDDC3	0.040563
UNC45A	0.040563
RCCD1	0.040563
PRC1	0.03045
VPS33B	0.03045
SV2B	0.03045
snoU13|ENSG00000238981.1	0.026768
SLCO3A1	0.01773
ST8SIA2	0.008535
snoU109|ENSG00000239197.1	0.011602
C15orf32	0.008535
LINC00930	0.011654
FAM174B	0.006559
RN7SL599P	0.011111
CHD2	0.003857
MIR3175	0.003857
RGMA	0.005733
MCTP2	0.006647
LINC00924	0.014716
NR2F2	0.013804

MIR1469	0.013804
RN7SKP254	0.014503
SPATA8	0.040185
RN7SKP181	0.040185
RNA5SP401	0.040185
LINC00923	0.043538
ARRDC4	0.043652
FAM169B	0.037991
CERS3	0.040012
LINS	0.040012
ARHGDIG	5.15E-06
AXIN1	5.15E-06
C16orf11	5.15E-06
CAPN15	5.15E-06
DDX11L10	5.15E-06
DECR2	5.15E-06
HBA1	5.15E-06
HBA2	5.15E-06
HBM	5.15E-06
HBQ1	5.15E-06
HBZ	5.15E-06
ITFG3	5.15E-06
LUC7L	5.15E-06
MIR3176	5.15E-06
MIR5587	5.15E-06
MPG	5.15E-06
MRPL28	5.15E-06
NHLRC4	5.15E-06
NME4	5.15E-06
NPRL3	5.15E-06
PDIA2	5.15E-06
PIGQ	5.15E-06
POLR3K	5.15E-06
RAB11FIP3	5.15E-06
RAB40C	2.86E-06
RGS11	5.15E-06
RHBDF1	5.15E-06
SNRNP25	5.15E-06
TMEM8A	5.15E-06
WASH4P	5.15E-06
WASIR2	5.15E-06
C16orf13	2.86E-06
FAM195A	2.86E-06
WDR90	2.86E-06
WFIKKN1	2.86E-06
RHOT2	2.86E-06
JMJD8	2.86E-06
RHBDL1	2.86E-06
STUB1	2.86E-06
FBXL16	4.16E-06
WDR24	4.16E-06
METRN	4.16E-06
CCDC78	4.16E-06
FAM173A	4.16E-06
HAGHL	4.16E-06
NARFL	4.16E-06
CHTF18	2.85E-06
GNG13	2.85E-06

MIR662	2.85E-06
MSLNL	2.85E-06
MSLN	2.85E-06
PRR25	2.85E-06
RPUSD1	2.85E-06
LMF1	1.95E-06
SOX8	4.82E-06
SSTR5	4.82E-06
C1QTNF8	4.82E-06
CACNA1H	4.82E-06
PRSS29P	4.82E-06
TPSAB1	4.82E-06
TPSB2	4.82E-06
TPSD1	4.82E-06
TPSG1	4.82E-06
UBE2I	7.12E-06
BAIAP3	6.05E-06
GNPTG	6.05E-06
TSR3	6.05E-06
UNKL	9.06E-06
C16orf91	1.61E-05
CCDC154	1.61E-05
CLCN7	1.61E-05
PTX4	1.70E-05
TELO2	1.70E-05
IFT140	1.70E-05
TMEM204	1.70E-05
CRAMP1L	2.97E-05
HN1L	3.17E-05
MAPK8IP3	3.17E-05
MIR3177	3.17E-05
EME2	1.81E-05
MRPS34	1.81E-05
NME3	1.81E-05
NUBP2	3.17E-05
SPSB3	3.17E-05
HAGH	4.92E-05
IGFALS	4.92E-05
FAHD1	4.92E-05
MEIOB	3.32E-05
RN7SL367P	1.29E-05
HS3ST6	1.29E-05
MSRB1	4.92E-05
RPL3L	4.92E-05
NDUFB10	4.92E-05
RNF151	4.92E-05
RPS2	4.92E-05
SNHG9	4.92E-05
SNORA10	4.92E-05
SNORA64|ENSG00000207405.1	4.92E-05
TBL3	4.92E-05
NOXO1	4.92E-05
GFER	4.92E-05
SYNGR3	4.92E-05
NPW	4.92E-05
ZNF598	4.92E-05
RN7SL219P	4.92E-05
SLC9A3R2	2.71E-05

NTHL1	2.71E-05
TSC2	2.71E-05
MIR1225	2.71E-05
PKD1	2.71E-05
CASKIN1	2.71E-05
MIR4516	2.71E-05
RAB26	2.71E-05
SNORD60|ENSG00000206630.1	2.71E-05
TRAF7	2.71E-05
MLST8	2.71E-05
BRICD5	2.71E-05
PGP	2.71E-05
DNASE1L2	2.71E-05
E4F1	2.71E-05
ECI1	2.71E-05
RNPS1	2.71E-05
MIR940	2.83E-05
MIR3677	2.83E-05
ABCA3	2.83E-05
MIR4717	2.83E-05
ABCA17P	2.83E-05
CCNF	6.56E-05
C16orf59	6.56E-05
NTN3	6.56E-05
TBC1D24	6.56E-05
ATP6C	6.56E-05
ATP6V0C	6.56E-05
AMDHD2	6.56E-05
CEMP1	6.56E-05
MIR3178	6.56E-05
PDPK1	6.56E-05
KCTD5	6.56E-05
PDPK2	6.56E-05
PRSS27	6.56E-05
SRRM2	6.56E-05
TCEB2	6.56E-05
PRSS33	6.56E-05
PRSS41	9.58E-05
SNORA3|ENSG00000221719.1	9.58E-05
PRSS21	9.58E-05
ZG16B	9.58E-05
PRSS30P	9.58E-05
PRSS22	9.58E-05
FLYWCH2	9.58E-05
FLYWCH1	9.58E-05
KREMEN2	9.28E-05
PAQR4	9.28E-05
PKMYT1	9.28E-05
LINC00514	9.28E-05
CLDN6	9.28E-05
CLDN9	9.28E-05
CCDC64B	9.28E-05
HCFC1R1	9.28E-05
THOC6	9.28E-05
TNFRSF12A	9.28E-05
MMP25	9.28E-05
IL32	9.28E-05
ZSCAN10	9.28E-05

ZNF205	9.28E-05
ZNF213	9.28E-05
CASP16	9.28E-05
OR1F1	9.28E-05
ZNF200	9.28E-05
MEFV	9.28E-05
ZNF263	8.64E-05
TIGD7	0.000134
ZNF75A	0.000134
OR2C1	0.000134
MTRNR2L4	0.000134
ZSCAN32	0.000134
ZNF174	0.000134
ZNF597	0.000134
NAA60	0.000232
C16orf90	0.000232
CLUAP1	0.000232
NLRC3	0.000232
SLX4	0.000232
DNASE1	8.64E-05
TRAP1	0.000193
CREBBP	0.000259
ADCY9	0.000195
SRL	0.000375
TFAP4	0.000303
GLIS2	0.000466
PAM16	0.000466
CORO7	0.000483
VASN	0.000466
DNAJA3	0.000483
NMRAL1	0.000573
HMOX2	0.000525
CDIP1	0.000525
C16orf96	0.000525
UBALD1	0.000336
MGRN1	0.000336
RN7SL850P	0.000336
NUDT16L1	0.000336
ANKS3	0.000336
C16orf71	0.000284
ZNF500	0.000284
12-Sep	0.000284
SMIM22	0.000284
GLYR1	0.000826
ROGDI	0.000284
UBN1	0.000826
PPL	0.000826
SEC14L5	0.000699
NAGPA	0.000699
ALG1	0.000699
C16orf89	0.000699
FAM86A	0.000699
RBFOX1	0.00282
SNORA40|ENSG00000252138.1	0.006677
TMEM114	0.000719
METTL22	0.000341
ABAT	0.000337
RN7SL743P	0.000337

TMEM186	0.000337
PMM2	0.000337
C16orf72	0.000337
CARHSP1	0.000337
USP7	0.000337
RNA5SP403	0.000499
RNA5SP404	0.000499
GRIN2A	0.000668
RN7SL493P	0.000135
ATF7IP2	0.000423
RN7SL99P	0.000273
EMP2	0.000786
TEKT5	0.000815
NUBP1	0.001752
TVP23A	0.001529
CIITA	0.000644
DEXI	0.000644
CLEC16A	0.000656
RMI2	0.000734
SOCS1	0.000734
TNP2	0.000734
PRM3	0.000734
SNORA48|ENSG00000212228.1	0.000734
PRM2	0.000734
PRM1	0.000734
snoU13|ENSG00000238409.1	0.000527
MIR548H2	0.000527
snoU13|ENSG00000272310.1	0.00093
RN7SL522P	0.000582
LITAF	0.00026
SNN	0.000531
TXNDC11	0.000544
ZC3H7A	0.000421
BCAR4	0.000421
RSL1D1	0.000904
GSPT1	0.000904
TNFRSF17	0.00134
SNX29	0.001235
ACA64|ENSG00000238685.1	0.001291
CPPED1	0.000552
MIR4718	0.000828
SNORA27|ENSG00000199474.1	0.001138
SHISA9	0.003276
ERCC4	0.001396
MKL2	0.000879
MIR193B	0.000879
MIR365A	0.000879
PARN	0.001326
RN7SL274P	0.001481
BFAR	0.001354
ABCC1	0.001414
ABCC6P1	0.001414
ABCC6	0.001414
ARL6IP1	0.00155
C16orf45	0.001414
FOPNL	0.001414
KIAA0430	0.001414
MIR484	0.001414

MPV17L	0.001414
MYH11	0.001414
NDE1	0.001414
NOMO1	0.001414
NOMO2	0.001414
NOMO3	0.001414
NPIPA1	0.001414
NPIPA2	0.001414
NPIPA3	0.001414
NPIPA5	0.001414
NPIPA7	0.001414
NPIPA8	0.001414
NPIPP1	0.001414
NTAN1	0.001414
PDXDC1	0.001414
PKD1P5	0.001414
PKD1P6	0.001414
PLA2G10	0.001414
RN7SL90P	0.001414
RPS15A	0.001414
RRN3	0.001414
XYLT1	0.001414
SMG1	0.004876
snoU13|ENSG00000238329.1	0.004703
TMC7	0.004444
COQ7	0.004444
ITPRIPL2	0.004444
SYT17	0.003006
CLEC19A	0.003006
TMC5	0.002298
GDE1	0.002504
CCP110	0.002504
C16orf62	0.002504
KNOP1	0.002284
IQCK	0.002091
GPRC5B	0.002423
GPR139	0.003414
GP2	0.005394
UMOD	0.005394
PDILT	0.005394
ACSM5	0.005394
ACSM2A	0.005394
ACSM2B	0.005394
ACSM1	0.005394
ACSM3	0.003657
THUMPD1	0.007734
ERI2	0.005303
DCUN1D3	0.005303
LYRM1	0.005303
DNAH3	0.005184
TMEM159	0.005184
ZP2	0.005184
ANKS4B	0.005184
CRYM	0.005184
C16orf52	0.006349
CDR2	0.006349
EEF2K	0.006349
IGSF6	0.006349

METTL9	0.006349
NPIPB3	0.006349
NPIPB4	0.006349
NPIPB5	0.006349
OTOA	0.006349
PDZD9	0.006349
POLR3E	0.006349
RN7SL245P	0.006349
RRN3P1	0.006349
RRN3P3	0.006349
SCARNA6|ENSG00000252798.1	0.006349
SDR42E2	0.006349
SMG1P1	0.006349
UQCRC2	0.006349
VWA3A	0.006349
snoU13|ENSG00000238712.1	0.006349
snoU13|ENSG00000238954.1	0.006349
snoU13|ENSG00000239172.1	0.006349
HS3ST2	0.004989
USP31	0.003785
SCNN1G	0.003785
SCNN1B	0.003785
COG7	0.003785
RN7SKP23	0.003785
SNORA75|ENSG00000212593.1	0.003785
GGA2	0.003785
EARS2	0.003785
UBFD1	0.004188
NDUFAB1	0.004087
PALB2	0.004087
DCTN5	0.004087
PLK1	0.003738
ERN2	0.002779
CHP2	0.002779
PRKCB	0.004729
CACNG3	0.005533
SNORA1|ENSG00000201541.1	0.005533
RBBP6	0.00417
TNRC6A	0.00417
SLC5A11	0.003777
ARHGAP17	0.003777
LCMT1	0.003843
RN7SL557P	0.002898
AQP8	0.002898
ZKSCAN2	0.002898
HS3ST4	0.000397
MIR548W	0.0004
RNA5SP405	0.0004
C16orf82	0.001596
KDM8	0.001596
NSMCE1	0.002053
IL4R	0.000915
IL21R	0.001416
GTF3C1	0.00141
KIAA0556	0.00136
GSG1L	0.002063
XPO6	0.002723
SNORA25|ENSG00000200652.1	0.002723

SBK1	0.003633
NPIPB6	0.003055
CLN3	0.003055
EIF3CL	0.003055
NPIPB7	0.003055
snoU13|ENSG00000238703.1	0.003055
APOBR	0.003055
IL27	0.003055
NUPR1	0.002133
CCDC101	0.002133
SULT1A2	0.002487
SULT1A1	0.002922
ATP2A1	0.002922
ATXN2L	0.002922
CD19	0.002922
EIF3C	0.002922
LAT	0.002922
MIR4517	0.002922
MIR4721	0.002922
NFATC2IP	0.002922
NPIPB8	0.002922
NPIPB9	0.002922
RABEP2	0.002922
RRN3P2	0.003804
SH2B1	0.002922
SNORA43|ENSG00000252461.1	0.002922
SPNS1	0.002922
TUFM	0.002922
snoU13|ENSG00000238352.1	0.002922
snoU13|ENSG00000238684.1	0.002922
snoU13|ENSG00000238699.1	0.002922
SNX29P2	0.006395
NPIPB11	0.006112
ALDOA	0.017711
ASPHD1	0.017711
BOLA2B	0.017711
BOLA2	0.017711
C16orf54	0.017711
C16orf92	0.017711
CDIPT	0.017711
CORO1A	0.017711
DOC2A	0.017711
FAM57B	0.017711
GDPD3	0.017711
HIRIP3	0.017711
INO80E	0.017711
KCTD13	0.017711
KIF22	0.017711
MAPK3	0.017711
MAZ	0.017711
MVP	0.017711
PAGR1	0.017711
PPP4C	0.017711
PRRT2	0.017711
QPRT	0.017711
RN7SKP127	0.017711
SEZ6L2	0.017711
SLX1A	0.017711

SLX1B	0.017711
SPN	0.017711
SULT1A3	0.017711
SULT1A4	0.017711
TAOK2	0.017711
TBX6	0.017711
TMEM219	0.017711
YPEL3	0.017711
ZG16	0.017711
snoU13|ENSG00000238639.1	0.017711
snoU13|ENSG00000239114.1	0.017711
snoU13|ENSG00000239193.1	0.017711
CD2BP2	0.031705
TBC1D10B	0.031705
MYLPF	0.031705
ZNF48	0.031705
ZNF771	0.037664
DCTPP1	0.037664
SNORA42|ENSG00000199787.1	0.037664
SEPHS2	0.037664
ZNF764	0.03116
ZNF688	0.03116
ZNF785	0.028299
ZNF689	0.024866
FBRS	0.024866
PRR14	0.024866
SRCAP	0.032943
SNORA30|ENSG00000206755.1	0.032943
CTF1	0.04418
STX4	0.029815
ZNF668	0.022504
PRSS53	0.025701
ZNF646	0.025701
VKORC1	0.025701
BCKDK	0.021344
KAT8	0.021344
PRSS8	0.021344
PRSS36	0.021344
FUS	0.015027
C16orf98	0.015027
PYCARD	0.015027
PYDC1	0.015027
TRIM72	0.015027
ITGAM	0.017659
ITGAX	0.01313
ITGAD	0.024851
COX6A2	0.024838
ZNF843	0.024838
ARMC5	0.024838
TGFB1I1	0.024838
SLC5A2	0.016384
C16orf58	0.016384
AHSP	0.016384
CLUHP3	0.010677
ZNF720	0.012426
ANKRD26P1	0.014411
ARHGAP23P1	0.022364
HERC2P4	0.022364

HERC2P5	0.022364
HERC2P8	0.022364
LINC00273	0.022364
RNA5SP406	0.022364
RNA5SP407	0.022364
RNA5SP408	0.022364
RNA5SP409	0.022364
RNA5SP410	0.022364
RNA5SP413	0.022364
RNA5SP415	0.022364
RNA5SP416	0.022364
RNA5SP417	0.022364
RNA5SP418	0.022364
RNA5SP419	0.022364
RNA5SP420	0.022364
RNA5SP421	0.022364
RNA5SP422	0.022364
RNA5SP423	0.022364
SLC6A10P	0.022364
TP53TG3B	0.022364
TP53TG3C	0.022364
TP53TG3D	0.022364
TP53TG3	0.022364
ZNF267	0.022364
SHCBP1	0.013128
VPS35	0.012636
ORC6	0.012636
MYLK3	0.011962
C16orf87	0.009863
GPT2	0.009863
DNAJA2	0.009863
NETO2	0.018647
snoU13|ENSG00000238834.1	0.011962
ITFG1	0.035156
RNA5SP424	0.024062
PHKB	0.024062
RNA5SP425	0.024062
ABCC12	0.029114
ABCC11	0.016026
LONP2	0.02083
SIAH1	0.019316
RN7SL54P	0.024357
snoU13|ENSG00000239013.1	0.017987
N4BP1	0.013258
snoU13|ENSG00000239038.1	0.013258
CBLN1	0.010498
C16orf78	0.016035
ZNF423	0.016669
CNEP1R1	0.007012
HEATR3	0.007012
RNY4P3	0.007012
SNORA70|ENSG00000252526.1	0.00969
PAPD5	0.007012
ADCY7	0.007012
BRD7	0.00864
snoU13|ENSG00000238544.1	0.00864
SNORD112|ENSG00000252077.1	0.00864
NKD1	0.011727

SNX20	0.011727
NOD2	0.008448
CYLD	0.006271
RNA5SP426	0.004464
SALL1	0.004342
HNRNPA1P48	0.010457
RN7SKP142	0.010457
C16orf97	0.010457
LINC00919	0.013984
TOX3	0.017619
CASC16	0.017619
CHD9	0.014279
RNA5SP427	0.012256
snoU13|ENSG00000238645.1	0.012256
RBL2	0.009892
AKTIP	0.009892
RPGRIP1L	0.009892
FTO	0.011645
IRX3	0.00617
CRNDE	0.001969
IRX5	0.001969
RN7SL841P	0.003425
IRX6	0.003425
MMP2	0.00194
LPCAT2	0.001401
CAPNS2	0.001455
SLC6A2	0.001995
CES1P1	0.006187
CES1	0.006187
CES5A	0.000982
GNAO1	0.003646
MIR3935	0.002639
AMFR	0.003646
NUDT21	0.016528
OGFOD1	0.016528
BBS2	0.016528
MT4	0.017814
MT3	0.017814
MT2A	0.017814
MT1L	0.017814
MT1E	0.017508
MT1M	0.017508
MT1JP	0.017508
MT1A	0.017508
MT1DP	0.017508
MT1B	0.017508
MT1F	0.017508
MT1G	0.017508
MT1H	0.017508
MT1X	0.017508
NUP93	0.017123
SLC12A3	0.017123
HERPUD1	0.02176
CETP	0.02176
CPNE2	0.02176
FAM192A	0.02176
RSPRY1	0.028009
ARL2BP	0.027133

PLLP	0.0261
CCL22	0.0261
CX3CL1	0.0261
CCL17	0.0261
CIAPIN1	0.0261
COQ9	0.0261
POLR2C	0.0261
DOK4	0.0261
CCDC102A	0.019356
GPR114	0.019356
GPR56	0.023965
GPR97	0.025221
CCDC135	0.025221
KATNB1	0.033859
KIFC3	0.044454
CNGB1	0.03355
TEPP	0.034713
USB1	0.02147
ZNF319	0.027891
MMP15	0.02147
C16orf80	0.02147
CSNK2A2	0.027891
RN7SL645P	0.027891
CCDC113	0.027891
PRSS54	0.027891
GINS3	0.027891
NDRG4	0.035974
SETD6	0.045615
CNOT1	0.035974
SNORA46|ENSG00000207493.1	0.045615
SNORA50|ENSG00000206952.2	0.045615
snoU13|ENSG00000239121.1	0.035974
SLC38A7	0.037819
CDH8	0.048478
RN7SKP76	0.030736
snoU13|ENSG00000238507.1	0.010023
CTRL	0.033406
PSMB10	0.033406
LCAT	0.033406
SLC12A4	0.033406
DPEP3	0.033406
DPEP2	0.033406
DUS2L	0.033406
DDX28	0.033406
NFATC3	0.042984
SNORA48|ENSG00000212445.1	0.042219
ESRP2	0.042219
PLA2G15	0.042219
SLC7A6	0.042219
SLC7A6OS	0.042219
PRMT7	0.042219
snoU13|ENSG00000238343.1	0.042219
CDH1	0.033502
RNA5SP429	0.042516
TANGO6	0.026582
RPS2P45	0.033911
HAS3	0.026582
CHTF8	0.026582

CIRH1A	0.026834
SNTB2	0.026834
VPS4A	0.033393
COG8	0.043353
PDF	0.043353
NIP7	0.043353
TMED6	0.043353
TERF2	0.043353
AP1G1	0.038403
ATXN1L	0.038403
ZNF821	0.038403
IST1	0.038403
PKD1L3	0.048431
DHODH	0.048431
TXNL4B	0.048431
HPR	0.048431
HP	0.048431
ZFHX3	0.044323
HCCAT5	0.043086
C16orf47	0.043086
CNTNAP4	0.039592
RN7SKP233	0.039592
SNORD33|ENSG00000252022.1	0.039592
DYNLRB2	0.047567
CMC2	0.01631
CENPN	0.02981
ATMIN	0.02981
C16orf46	0.02981
GCSH	0.02981
PKD1L2	0.02981
BCMO1	0.034615
GAN	0.02981
MIR4720	0.026981
CMIP	0.021973
PLCG2	0.039999
7SK|ENSG00000260682.2	0.026535
SDR42E1	0.026535
HSD17B2	0.022252
MPHOSPH6	0.022252
RN7SKP190	0.022252
snoU13|ENSG00000238321.1	0.032467
CDH13	0.032074
RN7SL134P	0.030992
MIR3182	0.042654
LINC00917	0.047571
SLC7A5	0.041649
CA5A	0.041649
BANP	0.035933
ZNF469	0.041437
YWHAE	0.046178
CRK	0.035029
MYO1C	0.047212
INPP5K	0.047212
PITPNA	0.047212
SLC43A2	0.043975
RN7SL105P	0.043975
snoU13|ENSG00000238946.1	0.047212
SMYD4	0.046908

RPA1	0.034834
SMG6	0.037756
RN7SL624P	0.037756
SRR	0.037756
SNORD91A	0.037756
SNORD91B	0.037756
TSR1	0.037756
SGSM2	0.037756
MNT	0.043483
METTL16	0.02631
RN7SL33P	0.043763
PAFAH1B1	0.043763
snoU13|ENSG00000239024.1	0.043763
RN7SL608P	0.043763
CLUH	0.043763
MIR1253	0.026455
RN7SL605P	0.026455
RAP1GAP2	0.026455
OR1D2	0.026455
OR1D5	0.026455
OR1G1	0.029168
OR1A2	0.029168
OR1A1	0.029168
OR1D4	0.029168
OR3A2	0.029168
OR3A1	0.029168
OR3A4P	0.036471
OR1E1	0.047221
OR3A3	0.047221
OR1E2	0.047221
SPATA22	0.047221
ASPA	0.047221
TRPV3	0.047221
SHPK	0.035574
TRPV1	0.035574
CTNS	0.035574
EMC6	0.035574
P2RX5	0.035574
TAX1BP3	0.035574
ITGAE	0.027097
GSG2	0.025845
C17orf85	0.027097
CAMKK1	0.027097
P2RX1	0.037565
ATP2A3	0.025772
ZZEF1	0.017805
RNA5SP434	0.017805
snoU13|ENSG00000238807.1	0.017805
CYB5D2	0.027097
ANKFY1	0.027726
UBE2G1	0.027726
RN7SL774P	0.027726
SPNS3	0.042554
SPNS2	0.027097
MYBBP1A	0.018566
GGT6	0.018566
SMTNL2	0.018566
ALOX15	0.018566

PELP1	0.018566
ARRB2	0.018566
MED11	0.018924
CXCL16	0.018924
ZMYND15	0.018924
GLTPD2	0.018924
TM4SF5	0.018924
VMO1	0.018924
PLD2	0.028109
PSMB6	0.028109
MINK1	0.027358
RN7SL171P	0.027358
RN7SL784P	0.027358
CHRNE	0.027358
C17orf107	0.027358
GP1BA	0.027358
SLC25A11	0.027358
RNF167	0.027358
ENO3	0.027358
PFN1	0.027358
SPAG7	0.018987
CAMTA2	0.018987
INCA1	0.027358
KIF1C	0.027358
SLC52A1	0.028109
ZFP3	0.028109
USP6	0.028109
ZNF232	0.028109
ZNF594	0.028109
SCIMP	0.040788
RABEP1	0.039304
NUP88	0.039304
RPAIN	0.037711
C1QBP	0.037711
DHX33	0.037711
DERL2	0.037711
MIS12	0.037711
NLRP1	0.036111
WSCD1	0.044584
AIPL1	0.03427
FAM64A	0.03427
ACKR6	0.046209
KIAA0753	0.024475
RNA5SP435	0.033737
MED31	0.016676
TXNDC17	0.016676
C17orf100	0.016676
MIR4520B	0.016676
SLC13A5	0.016676
XAF1	0.022074
FBXO39	0.022074
TEKT1	0.022074
ALOX12P2	0.015245
ALOX12	0.015245
C17orf49	0.022611
MIR497HG	0.022611
RNASEK	0.022611
BCL6B	0.022611

SLC16A11	0.022611
SLC16A13	0.022611
CLEC10A	0.022074
ASGR2	0.022074
ASGR1	0.021309
DLG4	0.036142
ACADVL	0.036142
DVL2	0.036142
MIR324	0.036142
PHF23	0.036142
GABARAP	0.036142
CTDNEP1	0.036142
ELP5	0.036142
CLDN7	0.036142
SLC2A4	0.036142
YBX2	0.036142
EIF5A	0.021309
GPS2	0.021309
NEURL4	0.021309
ACAP1	0.021309
KCTD11	0.021309
TMEM95	0.021309
TNK1	0.021309
PLSCR3	0.021309
NLGN2	0.021309
TMEM256	0.021309
SPEM1	0.021309
C17orf74	0.021309
FGF11	0.021309
TMEM102	0.021309
CHRNB1	0.022134
ZBTB4	0.022134
SLC35G6	0.022134
POLR2A	0.022611
TNFSF12	0.022074
SENP3	0.022074
TNFSF13	0.022074
EIF4A1	0.022074
SNORA48|ENSG00000209582.1	0.022074
SNORA67|ENSG00000264772.2	0.022074
SNORD10	0.022074
CD68	0.022074
FXR2	0.022074
MPDU1	0.022074
SOX15	0.022074
SHBG	0.022074
snoU13|ENSG00000251860.1	0.022074
SAT2	0.022074
ATP1B2	0.022074
TP53	0.022074
WRAP53	0.022546
EFNB3	0.022546
DNAH2	0.022546
RPL29P2	0.022546
KDM6B	0.032483
CYB5D1	0.032483
LSMD1	0.032483
TMEM88	0.032483

CHD3	0.032483
SCARNA21|ENSG00000252835.1	0.032483
KCNAB3	0.032483
CNTROB	0.032483
TRAPPC1	0.032483
GUCY2D	0.032483
snoU13|ENSG00000238676.1	0.032483
ALOX15B	0.032483
ALOX12B	0.032483
MIR4314	0.032483
ALOXE3	0.032483
HES7	0.022546
PER1	0.022264
VAMP2	0.022264
SNORD118	0.022264
TMEM107	0.022264
C17orf59	0.022264
MIR3676	0.022264
AURKB	0.021461
LINC00324	0.02432
CTC1	0.02432
PFAS	0.017582
SLC25A35	0.017582
RANGRF	0.017582
ARHGEF15	0.025714
SNORA69|ENSG00000212206.1	0.025714
ODF4	0.026232
KRBA2	0.026232
RPL26	0.026232
RNF222	0.043051
NDEL1	0.044382
MYH10	0.044825
RN7SL129P	0.043051
PIK3R6	0.039673
PIK3R5	0.033259
MYOCD	0.048775
BCAS3	0.030481
RNA5SP444	0.049744
RN7SL735P	0.045264
CANT1	0.037589
C1QTNF1	0.032837
ENGASE	0.04538
RBFOX3	0.045629
TBC1D16	0.038454
CCDC40	0.04205
GAA	0.033543
EIF4A3	0.030751
CARD14	0.030751
SGSH	0.04205
SLC26A11	0.04205
RNF213	0.04205
CHMP6	0.034412
BAIAP2	0.02254
AATK	0.02254
MIR1250	0.02254
MIR338	0.02254
MIR657	0.02254
AZI1	0.02678

ENTHD2	0.02678
C17orf89	0.029027
SLC38A10	0.029027
LINC00482	0.029027
TMEM105	0.029027
MIR4740	0.030055
MIR3186	0.03189
ACTG1	0.030951
FSCN2	0.030951
C17orf70	0.030951
NPLOC4	0.027579
TSPAN10	0.027826
PDE6G	0.027826
OXLD1	0.027826
CCDC137	0.027826
ARL16	0.027826
HGS	0.019353
MRPL12	0.019353
SLC25A10	0.019353
FAM195B	0.013365
GCGR	0.013365
P4HB	0.013365
PPP1R27	0.013365
ARHGDIA	0.014549
ALYREF	0.01442
ANAPC11	0.014496
NPB	0.014496
PCYT2	0.014496
SIRT7	0.014496
MAFG	0.014496
MYADML2	0.014496
PYCR1	0.014496
NOTUM	0.015561
ASPSCR1	0.010366
STRA13	0.011003
DCXR	0.011003
LRRC45	0.011003
RAC3	0.011003
RFNG	0.01517
GPS1	0.01517
DUS1L	0.01517
FASN	0.014465
CCDC57	0.010876
CSNK1D	0.014465
SLC16A3	0.014465
snoU13|ENSG00000238947.1	0.014465
CD7	0.015858
SECTM1	0.015858
TEX19	0.015858
UTS2R	0.015727
OGFOD3	0.015727
HEXDC	0.015727
C17orf62	0.031151
NARF	0.031151
FOXK2	0.031151
snoU13|ENSG00000238403.1	0.031566
RAB40B	0.035991
MIR4525	0.037228

FN3KRP	0.034952
FN3K	0.034952
TBCD	0.017676
ZNF750	0.049515
B3GNTL1	0.017676
METRNL	0.017676
DLGAP1	0.022601
RAB12	0.044248
SOGA2	0.046627
VAPA	0.04327
APCDD1	0.042392
NAPG	0.042392
PIEZO2	0.045813
NPIPB1P	0.040369
GNAL	0.031311
CHMP1B	0.031311
MPPE1	0.031255
CIDEA	0.01158
TUBB6	0.01158
AFG3L2	0.022938
SLMO1	0.022938
SPIRE1	0.021181
snoU13|ENSG00000238309.1	0.012159
CEP76	0.003446
PSMG2	0.006844
PTPN2	0.017205
SEH1L	0.012221
CEP192	0.012499
LDLRAD4	0.021352
MIR5190	0.01231
MIR4526	0.042761
FAM210A	0.042761
RN7SL362P	0.031908
RNMT	0.031908
MC2R	0.024057
ZNF519	0.017288
ANKRD20A5P	0.014062
CYP4F35P	0.014062
CXADRP3	0.010663
POTEC	0.010663
ANKRD30B	0.005954
RN7SL662P	0.005954
ROCK1	0.005954
GREB1L	0.001231
SNORD23|ENSG00000221139.1	0.001014
ESCO1	0.002122
SNRPD1	0.002675
ABHD3	0.003955
MIR320C1	0.003955
MIB1	0.003954
SNORA81|ENSG00000252677.1	0.003955
RN7SL233P	0.003642
SNORA73|ENSG00000199977.1	0.003642
RNA5SP451	0.003572
RNU6ATAC20P	0.001495
GATA6	0.001495
snoU13|ENSG00000238907.1	0.001063
CTAGE1	0.001699

RBBP8	0.000205
snoU13|ENSG00000238537.1	0.00023
MIR4741	0.000359
RN7SL745P	0.000364
CABLES1	0.000626
TMEM241	0.001224
RIOK3	0.000747
C18orf8	0.00057
NPC1	0.00057
ANKRD29	0.000684
LAMA3	0.000437
TTC39C	0.000132
CABYR	0.000112
OSBPL1A	7.12E-05
RNA5SP452	6.35E-05
RN7SL247P	1.83E-05
MIR320C2	2.11E-05
IMPACT	3.43E-05
HRH4	3.07E-05
ZNF521	6.53E-05
RN7SL97P	0.00203
SS18	0.001478
PSMA8	0.002265
TAF4B	0.001504
U3|ENSG00000252921.1	0.001478
KCTD1	0.000685
U3|ENSG00000265369.2	0.000832
AQP4	0.00092
CHST9	0.002151
CDH2	0.000772
DSC3	4.20E-05
DSC2	3.07E-05
snoU13|ENSG00000238376.1	4.45E-05
DSC1	3.07E-05
DSG1	0.000172
DSG4	0.000172
DSG3	0.000172
DSG2	0.000271
TTR	0.000271
B4GALT6	0.000271
RN7SKP44	0.000398
LRRC37A7P	0.000398
SLC25A52	0.000462
TRAPPC8	0.000671
RNF125	0.000671
RNF138	0.000951
snoU13|ENSG00000238982.1	0.001743
GAREM	0.000588
RNA5SP453	0.000744
MEP1B	0.000458
WBP11P1	0.001167
KLHL14	0.000638
CCDC178	0.000103
ASXL3	0.000418
NOL4	0.002475
DTNA	0.002264
MAPRE2	0.001319
ZNF397	0.00177

ZSCAN30	0.00177
ZNF271	0.003004
ZNF24	0.001967
ZNF396	0.000857
INO80C	0.001255
GALNT1	0.001584
MIR187	0.000857
MIR3929	0.000569
C18orf21	0.000569
RPRD1A	0.000879
SLC39A6	0.002024
ELP2	0.002024
MOCOS	0.002024
FHOD3	0.000249
SNORD112|ENSG00000252078.1	0.000287
TPGS2	0.000144
KIAA1328	0.000135
CELF4	0.001188
MIR4318	0.000408
RN7SKP182	1.37E-05
LINC00669	6.19E-05
PIK3C3	1.89E-05
LINC00907	1.69E-05
RNA5SP454	1.89E-05
RIT2	1.69E-05
SYT4	1.69E-05
RNA5SP455	3.11E-05
SETBP1	2.32E-05
MIR4319	2.32E-05
SLC14A2	5.75E-06
SLC14A1	8.92E-06
SIGLEC15	8.92E-06
EPG5	8.92E-06
PSTPIP2	8.92E-06
RN7SKP26	8.92E-06
ATP5A1	8.92E-06
HAUS1	8.92E-06
C18orf25	8.92E-06
RNF165	7.08E-06
LOXHD1	3.80E-06
ST8SIA5	6.70E-06
PIAS2	2.88E-06
KATNAL2	1.75E-06
TCEB3B	2.88E-06
TCEB3CL2	2.88E-06
TCEB3CL	2.88E-06
TCEB3C	2.88E-06
HDHD2	1.75E-06
IER3IP1	1.75E-06
SKOR2	3.23E-06
MIR4527	3.18E-06
SMAD2	9.24E-06
ZBTB7C	3.20E-06
RNA5SP456	4.70E-06
CTIF	4.52E-06
MIR4743	5.21E-06
SMAD7	1.60E-05
DYM	3.45E-05

MIR4744	2.53E-05
C18orf32	1.21E-05
MIR1539	9.27E-06
RPL17	9.27E-06
SNORD58C	9.27E-06
SNORD58A	9.27E-06
SNORD58B	9.27E-06
LIPG	9.27E-06
ACAA2	6.01E-06
SCARNA17|ENSG00000251992.1	6.01E-06
SCARNA17|ENSG00000267322.1	6.01E-06
SCARNA18|ENSG00000252139.1	6.01E-06
MYO5B	1.87E-06
RNA5SP457	3.61E-06
RN7SL310P	4.19E-06
CCDC11	2.63E-06
MBD1	1.09E-06
CXXC1	1.09E-06
RNA5SP458	1.09E-06
SKA1	1.09E-06
MAPK4	2.42E-06
MRO	3.57E-06
ME2	3.96E-06
ELAC1	3.96E-06
SMAD4	4.68E-06
RN7SL695P	2.42E-06
MEX3C	7.91E-07
snoU13|ENSG00000238885.1	6.70E-07
DCC	1.06E-06
MBD2	2.36E-06
SNORA37	2.36E-06
POLI	2.36E-06
STARD6	2.36E-06
C18orf54	2.36E-06
DYNAP	2.36E-06
RAB27B	1.24E-06
CCDC68	1.24E-06
RNA5SP459	1.24E-06
TCF4	6.46E-07
MIR4529	6.46E-07
SNORA73|ENSG00000201816.1	1.65E-06
TXNL1	2.37E-06
WDR7	4.25E-06
U3|ENSG00000212539.1	3.71E-06
BOD1L2	6.72E-06
ST8SIA3	8.51E-07
ONECUT2	1.20E-06
FECH	1.20E-06
NARS	1.20E-06
ATP8B1	5.38E-07
NEDD4L	3.21E-06
MIR122	2.81E-06
ALPK2	2.81E-06
SNORD28|ENSG00000252284.1	2.81E-06
MALT1	2.81E-06
RN7SL112P	1.48E-06
U8|ENSG00000199713.1	1.48E-06
ZNF532	1.48E-06

OACYLP	2.81E-06
SEC11C	7.75E-06
GRP	6.63E-06
RAX	6.63E-06
CPLX4	1.24E-05
LMAN1	1.24E-05
CCBE1	2.20E-05
PMAIP1	2.17E-05
RN7SL342P	1.88E-05
MC4R	1.69E-05
CDH20	9.27E-06
RNF152	4.45E-06
PIGN	2.00E-05
KIAA1468	3.56E-05
TNFRSF11A	2.94E-05
ZCCHC2	1.13E-05
RN7SL705P	8.18E-06
PHLPP1	1.09E-05
BCL2	8.55E-06
snoU13|ENSG00000238988.1	1.32E-05
KDSR	1.32E-05
VPS4B	1.32E-05
SERPINB5	1.32E-05
SERPINB12	7.06E-06
SERPINB13	7.06E-06
SERPINB4	7.06E-06
SERPINB11	7.06E-06
SERPINB3	7.06E-06
SERPINB7	8.55E-06
SERPINB2	8.52E-06
SERPINB10	8.52E-06
HMSD	8.52E-06
SERPINB8	8.52E-06
LINC00305	2.18E-05
CDH7	5.93E-05
CDH19	1.20E-05
MIR5011	1.51E-05
DSEL	6.52E-06
TMX3	1.31E-05
CCDC102B	1.31E-05
DOK6	1.14E-05
CD226	9.62E-06
RTTN	1.32E-05
SOCS6	2.95E-06
RN7SL795P	7.86E-06
GTSCR1	7.86E-06
CBLN2	4.85E-06
MIR548AV	4.85E-06
NETO1	4.85E-06
RNA5SP460	4.85E-06
RN7SL401P	5.63E-06
FBXO15	1.90E-06
TIMM21	2.24E-06
RN7SL551P	2.24E-06
CYB5A	2.24E-06
C18orf63	2.67E-06
FAM69C	2.67E-06
CNDP2	1.42E-06

CNDP1	2.77E-06
LINC00909	2.67E-06
ZNF407	2.03E-06
ZADH2	1.54E-06
TSHZ1	1.53E-06
SMIM21	4.39E-06
ZNF516	6.31E-06
LINC00908	6.31E-06
LINC00683	6.31E-06
ZNF236	3.12E-06
MBP	8.28E-07
GALR1	8.28E-07
SNORA25|ENSG00000199392.1	5.83E-07
RNA5SP461	8.55E-07
SALL3	8.67E-07
ATP9B	4.89E-07
ADNP2	4.89E-07
CTDP1	4.89E-07
HSBP1L1	4.89E-07
KCNG2	4.89E-07
NFATC1	4.89E-07
PARD6G	4.89E-07
PQLC1	4.89E-07
RBFADN	4.89E-07
RBFA	4.89E-07
TXNL4A	4.89E-07
FAM138F	4.73E-05
OR4F17	4.73E-05
PPAP2C	4.73E-05
WASH5P	4.73E-05
MIER2	4.73E-05
THEG	4.73E-05
C2CD4C	2.98E-05
SHC2	2.47E-05
RNA5SP462	2.47E-05
ODF3L2	1.85E-05
MADCAM1	1.85E-05
TPGS1	1.85E-05
CDC34	1.85E-05
GZMM	1.85E-05
BSG	1.85E-05
HCN2	1.85E-05
POLRMT	1.85E-05
FGF22	1.85E-05
RNF126	1.85E-05
FSTL3	1.16E-05
PRSS57	8.67E-06
PALM	1.34E-05
MISP	2.67E-05
PTBP1	1.67E-05
MIR4745	1.67E-05
AZU1	1.06E-05
LPPR3	1.67E-05
MIR3187	1.67E-05
ARID3A	8.54E-06
CFD	8.54E-06
ELANE	8.54E-06
KISS1R	8.54E-06

MED16	8.54E-06
PRTN3	8.54E-06
R3HDM4	8.54E-06
WDR18	6.29E-06
GRIN3B	6.29E-06
TMEM259	6.29E-06
CNN2	6.29E-06
ABCA7	6.29E-06
HMHA1	6.14E-06
POLR2E	6.14E-06
GPX4	8.66E-06
SBNO2	6.11E-06
STK11	9.04E-06
ATP5D	2.03E-05
C19orf26	2.03E-05
CIRBP	2.03E-05
MIDN	2.03E-05
C19orf24	1.68E-05
EFNA2	1.48E-05
MUM1	1.48E-05
NDUFS7	8.22E-06
GAMT	8.22E-06
DAZAP1	8.22E-06
RPS15	8.22E-06
APC2	8.22E-06
C19orf25	8.22E-06
PCSK4	8.22E-06
REEP6	8.22E-06
ADAMTSL5	7.09E-06
PLK5	1.06E-05
MEX3D	7.91E-06
MBD3	8.53E-06
RN7SL477P	7.91E-06
UQCR11	8.53E-06
TCF3	1.31E-05
ATP8B3	1.22E-05
KLF16	1.22E-05
MIR1909	1.22E-05
ONECUT3	1.22E-05
REXO1	1.22E-05
ABHD17A	1.75E-05
ADAT3	1.75E-05
SCAMP4	1.75E-05
CSNK1G2	1.10E-05
BTBD2	1.08E-05
RN7SL226P	1.08E-05
MKNK2	2.76E-05
MOB3A	1.86E-05
AP3D1	3.17E-05
IZUMO4	3.17E-05
DOT1L	2.49E-05
MIR1227	2.49E-05
PLEKHJ1	2.49E-05
SF3A2	2.49E-05
AMH	2.49E-05
JSRP1	2.49E-05
MIR4321	2.49E-05
C19orf35	2.49E-05

OAZ1	2.49E-05
LINGO3	2.53E-05
LSM7	2.53E-05
SPPL2B	2.53E-05
TMPRSS9	2.53E-05
LMNB2	3.04E-05
TIMM13	2.53E-05
GADD45B	3.04E-05
GNG7	4.63E-05
DIRAS1	6.78E-05
SLC39A3	6.78E-05
SGTA	6.78E-05
THOP1	6.78E-05
ZNF554	4.63E-05
ZNF555	4.63E-05
ZNF556	4.63E-05
ZNF57	4.63E-05
ZNF77	4.63E-05
TLE6	6.09E-05
TLE2	6.09E-05
AES	6.25E-05
GNA11	7.53E-05
GNA15	6.25E-05
S1PR4	6.25E-05
NCLN	4.24E-05
CELF5	4.24E-05
NFIC	4.24E-05
C19orf77	3.04E-05
DOHH	2.54E-05
RN7SL866P	2.54E-05
FZR1	2.54E-05
SNORD38|ENSG00000252408.1	2.54E-05
C19orf71	2.54E-05
MFSD12	2.54E-05
GIPC3	2.54E-05
HMG20B	2.54E-05
TBXA2R	2.54E-05
CACTIN	3.35E-05
PIP5K1C	3.35E-05
TJP3	3.35E-05
APBA3	3.35E-05
MRPL54	3.35E-05
RAX2	3.35E-05
MATK	3.35E-05
ZFR2	3.35E-05
ATCAY	3.35E-05
RN7SL202P	3.35E-05
NMRK2	3.35E-05
DAPK3	3.35E-05
MIR637	3.35E-05
EEF2	3.35E-05
SNORD37|ENSG00000206775.1	3.35E-05
PIAS4	3.35E-05
ZBTB7A	3.35E-05
MAP2K2	3.35E-05
CREB3L3	5.39E-05
SIRT6	5.39E-05
ANKRD24	5.39E-05

RN7SL84P	5.39E-05
EBI3	5.39E-05
CCDC94	6.66E-05
SHD	4.24E-05
TMIGD2	2.03E-05
FSD1	2.03E-05
STAP2	2.03E-05
MPND	2.03E-05
SH3GL1	2.07E-05
CHAF1A	2.14E-05
C19orf10	2.14E-05
HDGFRP2	2.14E-05
LRG1	2.14E-05
MIR4746	2.14E-05
PLIN4	2.14E-05
PLIN5	2.14E-05
RN7SL121P	2.14E-05
RN7SL528P	2.14E-05
SEMA6B	2.14E-05
TNFAIP8L1	2.14E-05
UBXN6	2.14E-05
DPP9	2.18E-05
FEM1A	5.35E-05
TICAM1	5.35E-05
PLIN3	5.35E-05
ARRDC5	5.35E-05
UHRF1	5.35E-05
MIR4747	8.21E-05
KDM4B	3.35E-05
PTPRS	3.35E-05
RN7SL626P	1.79E-05
ZNRF4	1.79E-05
TINCR	1.79E-05
SAFB2	1.66E-05
SAFB	1.66E-05
RPL36	1.14E-05
C19orf70	1.14E-05
HSD11B1L	1.14E-05
LONP1	1.14E-05
CATSPERD	1.20E-05
DUS3L	2.95E-05
PRR22	2.95E-05
NRTN	2.95E-05
FUT3	1.17E-05
FUT5	1.17E-05
FUT6	1.17E-05
NDUFA11	1.79E-05
CAPS	1.79E-05
VMAC	1.79E-05
RANBP3	2.72E-05
RFX2	1.33E-05
ACSBG2	6.68E-06
MLLT1	1.49E-05
ACER1	1.90E-05
CLPP	1.67E-05
ALKBH7	1.67E-05
GTF2F1	1.58E-05
PSPN	1.67E-05

KHSRP	1.76E-05
MIR3940	1.76E-05
SLC25A41	1.76E-05
SLC25A23	1.76E-05
CRB3	1.76E-05
DENND1C	1.76E-05
TUBB4A	1.04E-05
TNFSF9	1.04E-05
CD70	1.04E-05
TNFSF14	1.04E-05
C3	1.04E-05
GPR108	1.04E-05
TRIP10	1.04E-05
SH2D3A	1.04E-05
VAV1	1.04E-05
EMR1	1.17E-05
EMR4P	1.17E-05
MBD3L2	1.17E-05
MBD3L3	1.17E-05
MBD3L4	1.17E-05
MBD3L5	1.17E-05
ZNF557	1.17E-05
INSR	1.17E-05
ARHGEF18	1.17E-05
PEX11G	1.79E-05
C19orf45	1.20E-05
ZNF358	1.20E-05
MCOLN1	1.20E-05
PNPLA6	1.20E-05
CAMSAP3	1.20E-05
XAB2	1.20E-05
PCP2	1.20E-05
PET100	1.20E-05
STXBP2	1.20E-05
RETN	1.20E-05
C19orf59	1.20E-05
TRAPPC5	1.20E-05
FCER2	1.20E-05
CLEC4G	7.74E-06
CD209	7.74E-06
CLEC4M	7.74E-06
EVI5L	4.54E-06
LRRC8E	4.54E-06
RNA5SP463	4.54E-06
MAP2K7	4.54E-06
RN7SL115P	4.54E-06
TGFBR3L	4.54E-06
SNAPC2	4.54E-06
CTXN1	4.54E-06
TIMM44	4.54E-06
ELAVL1	4.54E-06
CCL25	4.54E-06
FBN3	4.54E-06
CERS4	8.98E-06
CD320	1.24E-05
NDUFA7	1.24E-05
RPS28	1.24E-05
KANK3	1.24E-05

ANGPTL4	1.24E-05
MIR4999	1.24E-05
RAB11B	9.06E-06
2-Mar	9.06E-06
HNRNPM	9.15E-06
PRAM1	9.15E-06
ZNF414	9.15E-06
MYO1F	9.15E-06
ADAMTS10	9.15E-06
ACTL9	1.43E-05
OR2Z1	1.43E-05
ZNF558	8.18E-06
MBD3L1	8.18E-06
MUC16	1.63E-05
OR1M1	1.07E-05
OR7G2	1.07E-05
OR7G1	1.07E-05
OR7G3	1.07E-05
ZNF317	1.07E-05
OR7D2	1.14E-05
OR7D4	1.14E-05
OR7E24	1.14E-05
OR7E19P	1.14E-05
ZNF699	1.14E-05
ZNF177	1.16E-05
ZNF559	1.14E-05
ZNF266	1.16E-05
ZNF560	1.13E-05
ZNF426	1.13E-05
ZNF121	1.86E-05
ZNF561	1.86E-05
C19orf82	1.86E-05
ZNF562	1.86E-05
ZNF812	1.86E-05
ZNF846	1.86E-05
FBXL12	2.98E-05
SNORA70|ENSG00000200237.1	2.98E-05
RN7SL94P	2.98E-05
UBL5	2.98E-05
PIN1	2.98E-05
OLFM2	2.20E-05
COL5A3	6.49E-06
RDH8	3.82E-06
C3P1	3.82E-06
MIR5589	3.82E-06
C19orf66	3.82E-06
ANGPTL6	5.80E-06
PPAN	5.80E-06
SNORD105	5.80E-06
P2RY11	6.32E-06
SNORD105B	6.32E-06
EIF3G	6.32E-06
DNMT1	8.32E-06
S1PR2	8.32E-06
MIR4322	8.32E-06
MRPL4	1.09E-05
ICAM1	6.93E-06
ICAM4	7.75E-06

ICAM5	7.75E-06
ZGLP1	7.75E-06
FDX1L	7.75E-06
RAVER1	7.75E-06
ICAM3	7.75E-06
TYK2	1.09E-05
CDC37	1.07E-05
MIR1181	1.07E-05
PDE4A	9.04E-06
KEAP1	1.74E-05
S1PR5	1.42E-05
ATG4D	9.04E-06
MIR1238	9.04E-06
KRI1	9.04E-06
AP1M2	1.12E-05
CDKN2D	1.12E-05
SLC44A2	9.17E-06
ILF3	1.12E-05
QTRT1	1.12E-05
DNM2	1.12E-05
MIR638	1.12E-05
MIR4748	1.12E-05
MIR199A1	1.12E-05
C19orf38	1.12E-05
TMED1	1.12E-05
CARM1	6.54E-06
YIPF2	6.54E-06
C19orf52	6.54E-06
SMARCA4	1.37E-05
RN7SL192P	1.45E-05
LDLR	2.47E-05
SPC24	1.86E-05
KANK2	9.75E-06
DOCK6	9.75E-06
RN7SL298P	9.75E-06
C19orf80	9.75E-06
TSPAN16	1.26E-05
RAB3D	1.26E-05
CCDC159	1.26E-05
TMEM205	1.26E-05
DKFZP761J1410	1.26E-05
EPOR	1.18E-05
SWSAP1	1.26E-05
RGL3	1.18E-05
CCDC151	7.57E-06
PRKCSH	7.57E-06
snoU13|ENSG00000238349.1	7.57E-06
ELAVL3	7.57E-06
RN7SL669P	7.57E-06
ZNF653	7.57E-06
ECSIT	7.57E-06
RN7SL833P	7.57E-06
CNN1	6.55E-06
ELOF1	6.55E-06
ZNF627	6.55E-06
ACP5	6.55E-06
ZNF833P	7.57E-06
ZNF823	7.57E-06

ZNF441	1.08E-05
ZNF491	4.57E-06
ZNF439	3.08E-06
ZNF440	3.08E-06
ZNF69	3.08E-06
ZNF700	1.73E-06
ZNF763	1.50E-06
RNA5SP464	2.09E-06
ZNF433	2.09E-06
RNA5SP465	2.09E-06
ZNF878	2.09E-06
RNA5SP466	2.09E-06
ZNF844	2.09E-06
ZNF788	2.09E-06
ZNF20	2.09E-06
RNA5SP467	2.09E-06
ZNF625	2.09E-06
ZNF136	2.09E-06
ZNF44	1.22E-06
ZNF563	1.99E-06
ZNF442	1.99E-06
ZNF443	1.99E-06
ZNF799	1.99E-06
ZNF709	1.99E-06
ZNF564	1.99E-06
ZNF490	3.24E-06
ZNF791	3.24E-06
MAN2B1	3.24E-06
WDR83OS	3.24E-06
WDR83	3.24E-06
DHPS	3.24E-06
FBXW9	3.90E-06
TNPO2	3.90E-06
SNORD41|ENSG00000209702.1	3.90E-06
C19orf43	3.90E-06
ASNA1	4.35E-06
BEST2	4.35E-06
HOOK2	2.65E-06
MIR5684	4.35E-06
JUNB	4.35E-06
PRDX2	4.35E-06
RNASEH2A	4.35E-06
RTBDN	2.65E-06
MAST1	2.44E-06
DNASE2	2.44E-06
KLF1	2.44E-06
GCDH	2.44E-06
SYCE2	2.38E-06
FARSA	2.38E-06
MIR5695	2.38E-06
CALR	1.51E-06
RAD23A	9.25E-07
GADD45GIP1	9.25E-07
DAND5	9.25E-07
NFIX	5.24E-07
LYL1	5.24E-07
TRMT1	5.24E-07
NACC1	9.25E-07

STX10	9.25E-07
IER2	9.25E-07
CACNA1A	1.30E-06
CCDC130	7.93E-07
MRI1	7.69E-07
C19orf53	7.69E-07
ZSWIM4	7.69E-07
RN7SL619P	7.69E-07
MIR27A	7.69E-07
MIR23A	7.69E-07
NANOS3	7.69E-07
MIR181C	7.69E-07
MIR181D	7.69E-07
C19orf57	7.93E-07
CC2D1A	1.30E-06
PODNL1	2.20E-06
DCAF15	2.20E-06
RFX1	2.27E-06
IL27RA	1.25E-06
RLN3	1.25E-06
PALM3	2.54E-06
C19orf67	4.35E-06
PRKACA	4.35E-06
SAMD1	4.35E-06
ASF1B	4.35E-06
LPHN1	1.99E-06
RN7SL231P	2.32E-06
CD97	1.73E-06
DDX39A	1.73E-06
PKN1	1.73E-06
GIPC1	2.66E-06
PTGER1	2.79E-06
DNAJB1	2.66E-06
MIR639	2.66E-06
TECR	2.66E-06
NDUFB7	2.66E-06
CLEC17A	4.44E-06
RN7SL337P	4.44E-06
RN7SL842P	4.44E-06
EMR3	7.30E-06
ZNF333	1.20E-05
EMR2	1.14E-05
OR7A5	1.96E-05
OR7C1	1.96E-05
OR7A10	1.96E-05
OR7A17	1.77E-05
OR7C2	1.96E-05
SLC1A6	1.14E-05
CCDC105	1.46E-05
CASP14	8.88E-06
OR1I1	5.43E-06
SYDE1	8.88E-06
ILVBL	8.88E-06
NOTCH3	1.50E-05
EPHX3	1.50E-05
BRD4	1.39E-05
AKAP8	1.33E-05
AKAP8L	1.33E-05

WIZ	2.13E-05
MIR1470	2.13E-05
RASAL3	2.13E-05
PGLYRP2	1.87E-05
CYP4F22	1.87E-05
CYP4F23P	2.02E-05
CYP4F8	1.94E-05
CYP4F3	1.94E-05
CYP4F12	1.23E-05
OR10H2	1.23E-05
OR10H3	1.23E-05
CYP4F24P	9.43E-06
OR10H5	1.23E-05
OR10H1	1.23E-05
UCA1	1.23E-05
CYP4F2	1.23E-05
CYP4F11	1.23E-05
OR10H4	7.93E-06
LINC00661	1.23E-05
LINC00905	1.23E-05
TPM4	1.49E-05
RAB8A	1.49E-05
HSH2D	1.49E-05
CIB3	1.49E-05
FAM32A	1.49E-05
AP1M1	1.37E-05
KLF2	1.49E-05
EPS15L1	8.67E-06
RN7SL844P	1.43E-05
CALR3	1.43E-05
C19orf44	1.43E-05
CHERP	8.91E-06
RN7SL146P	8.91E-06
SLC35E1	2.14E-05
MED26	2.14E-05
SMIM7	2.12E-05
TMEM38A	2.12E-05
NWD1	1.35E-05
SIN3B	1.35E-05
F2RL3	1.35E-05
CPAMD8	1.35E-05
RN7SL835P	1.35E-05
RN7SL823P	1.35E-05
HAUS8	1.35E-05
MYO9B	1.42E-05
OCEL1	1.42E-05
USE1	1.42E-05
NR2F6	1.42E-05
USHBP1	1.92E-05
BABAM1	1.94E-05
ANKLE1	1.94E-05
ABHD8	1.94E-05
MRPL34	1.94E-05
DDA1	1.94E-05
ANO8	1.94E-05
GTPBP3	1.94E-05
PLVAP	1.94E-05
BST2	1.94E-05

MVB12A	1.94E-05
TMEM221	1.64E-05
NXNL1	1.64E-05
SLC27A1	1.64E-05
PGLS	1.94E-05
FAM129C	1.41E-05
COLGALT1	1.41E-05
UNC13A	1.66E-05
MAP1S	1.63E-05
FCHO1	1.67E-05
B3GNT3	1.06E-05
INSL3	1.13E-05
JAK3	1.13E-05
RPL18A	1.13E-05
SNORA68|ENSG00000207166.1	1.13E-05
SLC5A5	1.13E-05
CCDC124	1.13E-05
RNA5SP468	1.07E-05
ARRDC2	1.07E-05
IL12RB1	4.55E-06
MAST3	4.55E-06
PIK3R2	2.87E-06
IFI30	4.69E-06
MPV17L2	4.69E-06
RAB3A	4.69E-06
PDE4C	4.69E-06
KIAA1683	3.00E-06
JUND	3.00E-06
MIR3188	3.00E-06
LSM4	3.00E-06
RN7SL513P	3.00E-06
PGPEP1	4.78E-06
GDF15	3.01E-06
MIR3189	3.01E-06
LRRC25	3.01E-06
SSBP4	3.01E-06
ELL	3.01E-06
ISYNA1	3.01E-06
FKBP8	2.70E-06
KXD1	2.70E-06
CRLF1	4.21E-06
UBA52	4.22E-06
C19orf60	4.21E-06
TMEM59L	4.26E-06
RN7SL155P	4.26E-06
KLHL26	4.26E-06
CRTC1	4.26E-06
COMP	4.92E-06
UPF1	4.92E-06
CERS1	3.12E-06
GDF1	3.12E-06
COPE	3.12E-06
DDX49	3.12E-06
HOMER3	3.11E-06
RN7SL70P	3.11E-06
SUGP2	4.75E-06
ARMC6	7.43E-06
SLC25A42	1.08E-05

TMEM161A	1.08E-05
MEF2B	1.08E-05
MEF2BNB	1.08E-05
RFXANK	1.08E-05
NR2C2AP	1.08E-05
NCAN	6.95E-06
HAPLN4	6.95E-06
TM6SF2	6.95E-06
SUGP1	6.95E-06
MAU2	6.84E-06
GATAD2A	5.57E-06
MIR640	5.34E-06
NDUFA13	5.38E-06
TSSK6	5.38E-06
YJEFN3	5.38E-06
CILP2	5.38E-06
PBX4	1.20E-05
LPAR2	8.06E-06
GMIP	8.06E-06
ATP13A1	8.47E-06
ZNF101	1.08E-05
LINC00663	1.08E-05
ZNF14	1.08E-05
ZNF506	1.05E-05
ZNF253	2.16E-05
ZNF430	2.16E-05
ZNF486	2.16E-05
ZNF626	2.16E-05
ZNF66	2.16E-05
ZNF682	2.16E-05
ZNF737	2.16E-05
ZNF826P	2.16E-05
ZNF85	2.16E-05
ZNF90	2.16E-05
ZNF93	2.16E-05
ZNF714	1.06E-05
RNA5SP469	1.06E-05
ZNF431	7.19E-06
ZNF708	6.44E-06
ZNF738	7.19E-06
ZNF493	7.19E-06
LINC00664	5.00E-06
ZNF429	3.14E-06
ZNF100	1.90E-06
ZNF43	3.09E-06
ZNF208	8.32E-07
ZNF257	9.49E-07
ZNF676	9.49E-07
ZNF729	9.49E-07
ZNF98	9.67E-07
RN7SL860P	9.08E-07
ZNF492	1.20E-06
ZNF99	1.36E-06
ZNF728	1.44E-06
ZNF730	4.87E-07
ZNF724P	2.70E-07
ZNF91	5.88E-07
ZNF675	1.35E-06

ZNF681	1.02E-06
RPSAP58	1.29E-06
LINC00662	2.02E-06
ZNF254	1.35E-06
ZNF726	1.35E-06
snoU13|ENSG00000238514.1	0.000105
LINC00906	0.000108
RNA5SP470	7.22E-05
UQCRFS1	2.12E-05
RN7SL340P	2.12E-05
VSTM2B	2.09E-05
POP4	2.82E-05
PLEKHF1	2.82E-05
C19orf12	2.82E-05
CCNE1	9.10E-06
URI1	3.70E-05
ZNF536	9.44E-05
TSHZ3	7.42E-05
THEG5	6.64E-05
RNA5SP471	3.93E-05
RNA5SP472	0.00014
ZNF507	0.000179
DPY19L3	0.000266
PDCD5	0.000163
ANKRD27	0.000266
SNORA68|ENSG00000201388.1	0.000163
RN7SL789P	0.000163
RGS9BP	0.000266
NUDT19	0.000266
TDRD12	0.000266
SLC7A9	0.000199
RN7SKP22	0.000199
CEP89	0.000319
C19orf40	0.000319
RHPN2	0.000319
GPATCH1	0.000513
WDR88	0.000513
LRP3	0.000513
SLC7A10	0.000246
CEBPA	0.000183
CEBPG	0.000155
PEPD	0.000188
CHST8	0.000134
KCTD15	0.00028
RN7SL150P	0.000143
LSM14A	6.68E-05
KIAA0355	5.46E-05
GPI	7.29E-05
PDCD2L	7.53E-05
RN7SL154P	7.53E-05
UBA2	8.10E-05
WTIP	9.38E-05
SCGB1B2P	9.38E-05
SCGB2B2	0.000124
SNORD111|ENSG00000252230.1	9.74E-05
ZNF302	0.000124
ZNF181	0.000115
ZNF599	0.000115

LINC00904	0.000211
ZNF30	0.000143
ZNF792	0.000143
GRAMD1A	0.000162
SCN1B	0.000182
HPN	0.000182
FXYD3	0.000147
LGI4	0.000147
FXYD1	0.000147
FXYD7	0.000147
FXYD5	0.000147
FAM187B	0.000239
LSR	0.000169
USF2	0.000147
HAMP	0.000147
MAG	7.37E-05
CD22	7.37E-05
MIR5196	7.37E-05
FFAR1	7.37E-05
FFAR3	9.50E-05
GPR42	9.50E-05
RN7SL491P	9.50E-05
FFAR2	8.10E-05
KRTDAP	9.95E-05
DMKN	0.000108
SBSN	0.000108
GAPDHS	0.000108
TMEM147	0.000108
ATP4A	0.000108
HAUS5	0.000108
RBM42	0.000108
ETV2	0.000108
COX6B1	0.000108
RN7SL765P	0.000108
UPK1A	0.000108
ZBTB32	0.000108
KMT2B	0.000108
WBP7	0.000108
IGFLR1	9.95E-05
LIN37	9.95E-05
PSENEN	9.95E-05
U2AF1L4	9.95E-05
C19orf55	9.95E-05
HSPB6	9.95E-05
ARHGAP33	4.36E-05
PRODH2	4.36E-05
NPHS1	2.44E-05
KIRREL2	2.18E-05
APLP1	2.18E-05
RN7SL402P	2.93E-05
NFKBID	2.93E-05
HCST	4.75E-05
TYROBP	4.75E-05
LRFN3	7.60E-05
SDHAF1	8.44E-05
SYNE4	8.44E-05
ALKBH6	8.44E-05
CLIP3	8.44E-05

THAP8	8.44E-05
WDR62	8.44E-05
OVOL3	8.44E-05
POLR2I	8.44E-05
TBCB	8.44E-05
CAPNS1	7.41E-05
COX7A1	7.41E-05
ZNF565	7.41E-05
RN7SL287P	7.41E-05
ZNF146	7.41E-05
LINC00665	9.27E-05
ZFP14	9.27E-05
ZFP82	8.54E-05
ZNF566	6.19E-05
ZNF260	7.66E-05
ZNF529	8.60E-05
ZNF382	6.69E-05
ZNF461	4.90E-05
ZNF567	3.45E-05
ZNF850	6.69E-05
ZNF790	7.06E-05
ZNF345	8.08E-05
ZNF829	8.08E-05
ZNF568	8.08E-05
ZNF420	6.98E-05
ZNF585A	7.60E-05
ZNF585B	9.64E-05
ZNF383	9.62E-05
HKR1	5.43E-05
ZNF527	7.10E-05
ZNF569	7.10E-05
ZNF570	7.10E-05
ZNF793	9.27E-05
ZNF540	4.79E-05
ZNF571	4.79E-05
ZFP30	4.45E-05
ZNF781	4.45E-05
ZNF607	2.75E-05
ZNF573	2.75E-05
WDR87	3.56E-05
SIPA1L3	3.57E-05
DPF1	5.21E-05
RN7SL663P	3.19E-05
SPINT2	5.21E-05
PPP1R14A	5.21E-05
C19orf33	3.31E-05
YIF1B	3.31E-05
KCNK6	3.31E-05
CATSPERG	5.34E-05
snoU13|ENSG00000238838.1	5.34E-05
PSMD8	5.34E-05
GGN	5.34E-05
SPRED3	5.34E-05
FAM98C	5.34E-05
RASGRP4	5.34E-05
RYR1	4.80E-05
MAP4K1	6.73E-05
EIF3K	6.73E-05

ACTN4	6.73E-05
CAPN12	5.41E-05
LGALS7	5.41E-05
LGALS7B	6.44E-05
LGALS4	6.44E-05
ECH1	6.44E-05
HNRNPL	5.41E-05
RINL	5.41E-05
SIRT2	5.41E-05
NFKBIB	5.41E-05
SARS2	5.41E-05
MRPS12	5.41E-05
FBXO17	5.41E-05
FBXO27	3.77E-05
PAPL	2.37E-05
snoU13|ENSG00000251709.1	2.91E-05
PAK4	1.98E-05
NCCRP1	3.88E-05
SYCN	3.88E-05
IFNL3	3.88E-05
IFNL4	3.88E-05
IFNL2	5.70E-05
IFNL1	4.49E-05
LRFN1	4.49E-05
GMFG	5.34E-05
SAMD4B	5.34E-05
RN7SL566P	5.34E-05
PAF1	5.34E-05
MED29	5.34E-05
MIR4530	5.34E-05
PLEKHG2	5.34E-05
ZFP36	5.34E-05
RPS16	2.69E-05
SUPT5H	2.69E-05
DLL3	2.96E-05
TIMM50	2.96E-05
SELV	3.20E-05
EID2B	3.20E-05
EID2	3.20E-05
LGALS13	3.20E-05
LGALS16	3.20E-05
LGALS17A	4.13E-05
LGALS14	3.74E-05
CLC	3.02E-05
LEUTX	2.03E-05
DYRK1B	2.45E-05
FBL	2.45E-05
FCGBP	2.25E-05
PSMC4	2.25E-05
ZNF546	2.25E-05
ZNF780B	4.48E-05
ZNF780A	4.48E-05
MAP3K10	4.74E-05
TTC9B	4.74E-05
CNTD2	4.74E-05
AKT2	4.74E-05
MIR641	4.74E-05
C19orf47	3.56E-05

PLD3	9.84E-05
HIPK4	0.000104
PRX	0.000104
SERTAD1	5.56E-05
SERTAD3	6.70E-05
BLVRB	6.70E-05
SPTBN4	6.70E-05
LTBP4	4.64E-05
RN7SL758P	4.64E-05
SHKBP1	4.64E-05
NUMBL	7.09E-05
ADCK4	7.09E-05
ITPKC	7.09E-05
C19orf54	7.09E-05
SNRPA	7.09E-05
MIA	7.48E-05
RAB4B	7.48E-05
EGLN2	8.60E-05
CYP2A6	7.91E-05
CYP2A7	7.91E-05
CYP2G1P	7.93E-05
CYP2B7P1	8.45E-05
CYP2B6	7.93E-05
CYP2A13	2.11E-05
CYP2F1	2.11E-05
CYP2S1	3.95E-05
RN7SL718P	3.95E-05
AXL	3.95E-05
HNRNPUL1	2.54E-05
RN7SL34P	2.54E-05
TGFB1	2.77E-05
CCDC97	2.63E-05
B9D2	2.37E-05
BCKDHA	2.37E-05
TMEM91	2.37E-05
EXOSC5	2.37E-05
B3GNT8	1.64E-05
ATP5SL	1.64E-05
C19orf69	1.64E-05
CEACAM21	1.01E-05
CEACAMP3	1.15E-05
CEACAM4	1.31E-05
CEACAM7	2.15E-05
CEACAM5	2.15E-05
CEA	1.54E-05
CEACAM3	1.54E-05
CEACAM6	1.54E-05
LYPD4	1.60E-05
DMRTC2	1.60E-05
RPS19	1.60E-05
CD79A	9.34E-06
ARHGEF1	9.34E-06
RABAC1	9.34E-06
ATP1A3	1.37E-05
GRIK5	1.39E-05
ZNF574	2.51E-05
POU2F2	3.00E-05
MIR4323	1.96E-05

SNORD112|ENSG00000252356.1	1.96E-05
DEDD2	3.00E-05
ZNF526	3.00E-05
GSK3A	2.51E-05
ERF	1.96E-05
CIC	1.96E-05
PAFAH1B3	2.37E-05
PRR19	2.37E-05
TMEM145	2.37E-05
MEGF8	2.37E-05
CNFN	1.67E-05
LIPE	1.01E-05
CXCL17	1.01E-05
CEACAM1	1.24E-05
CEACAM8	1.45E-05
CD177	1.33E-05
CEACAMP10	1.33E-05
PSG10P	1.33E-05
PSG11	1.33E-05
PSG1	1.33E-05
PSG2	1.33E-05
PSG3	1.33E-05
PSG4	1.33E-05
PSG5	1.33E-05
PSG6	1.33E-05
PSG7	1.33E-05
PSG8	1.33E-05
PSG9	1.33E-05
TEX101	1.33E-05
LYPD3	2.83E-06
PHLDB3	2.38E-06
ETHE1	2.06E-06
ZNF575	3.11E-06
XRCC1	3.11E-06
PINLYP	2.46E-06
IRGQ	2.46E-06
SRRM5	2.46E-06
ZNF576	2.46E-06
ZNF428	2.46E-06
CADM4	2.13E-06
PLAUR	2.13E-06
RN7SL368P	2.13E-06
IRGC	2.46E-06
SMG9	2.46E-06
KCNN4	2.13E-06
LYPD5	1.40E-06
ZNF283	1.64E-06
ZNF404	1.40E-06
ZNF45	1.04E-06
ZNF221	5.73E-07
ZNF155	5.73E-07
ZNF230	3.70E-07
ZNF222	3.70E-07
ZNF223	4.30E-07
ZNF284	4.30E-07
RN7SL53P	4.30E-07
ZNF224	4.30E-07
ZNF225	4.30E-07

ZNF234	4.30E-07
ZNF226	4.30E-07
ZNF227	4.30E-07
ZNF235	4.30E-07
ZNF233	4.30E-07
ZNF112	6.52E-07
ZNF285	7.82E-07
ZNF229	7.82E-07
ZNF180	9.24E-07
CEACAM20	1.20E-06
CEACAM22P	4.04E-06
IGSF23	4.56E-06
PVR	2.61E-06
CEACAM19	2.61E-06
CEACAM16	2.37E-06
snoZ6|ENSG00000252200.1	2.55E-06
BCL3	2.48E-06
SNORA70|ENSG00000253027.1	2.48E-06
CBLC	2.27E-06
BCAM	3.85E-06
PVRL2	2.48E-06
TOMM40	1.20E-06
APOE	1.20E-06
APOC1	1.20E-06
APOC1P1	1.20E-06
APOC4	9.07E-07
APOC2	9.07E-07
CLPTM1	9.95E-07
RELB	1.32E-06
CLASRP	7.46E-07
ZNF296	6.94E-07
GEMIN7	6.94E-07
MARK4	1.41E-07
PPP1R37	4.43E-07
NKPD1	3.58E-07
TRAPPC6A	1.42E-07
BLOC1S3	1.12E-07
EXOC3L2	5.71E-08
CKM	3.32E-08
CD3EAP	3.32E-08
ERCC1	1.86E-08
ERCC2	3.32E-08
KLC3	3.32E-08
PPP1R13L	3.32E-08
FOSB	2.05E-08
RTN2	2.05E-08
PPM1N	2.05E-08
VASP	3.09E-08
OPA3	4.08E-08
GPR4	4.08E-08
EML2	4.84E-08
MIR330	4.84E-08
RN7SL836P	4.84E-08
GIPR	7.13E-08
MIR642A	7.13E-08
SNRPD2	7.13E-08
QPCTL	7.13E-08
FBXO46	1.28E-07

DMPK	3.38E-07
SIX5	3.38E-07
DMWD	3.38E-07
RSPH6A	3.38E-07
SYMPK	2.78E-07
FOXA3	3.38E-07
IRF2BP1	3.38E-07
MYPOP	3.38E-07
NANOS2	2.42E-07
NOVA2	1.97E-07
CCDC61	4.26E-07
MIR769	4.26E-07
PGLYRP1	4.26E-07
IGFL4	6.09E-07
IGFL3	7.24E-07
IGFL2	7.24E-07
IGFL1	7.24E-07
HIF3A	1.45E-06
PPP5C	1.45E-06
CCDC8	1.63E-06
PNMAL1	1.68E-06
PNMAL2	1.68E-06
PPP5D1	1.12E-06
CALM3	8.41E-07
PTGIR	8.41E-07
GNG8	8.41E-07
DACT3	3.93E-07
PRKD2	3.03E-07
RN7SL364P	4.65E-07
MIR320E	3.03E-07
STRN4	3.03E-07
FKRP	5.44E-07
SLC1A5	5.44E-07
AP2S1	1.28E-06
ARHGAP35	9.82E-07
snoU13|ENSG00000252071.1	5.92E-07
NPAS1	5.92E-07
TMEM160	5.92E-07
ZC3H4	5.92E-07
RN7SL533P	5.92E-07
SAE1	5.92E-07
BBC3	3.31E-07
MIR3191	3.31E-07
CCDC9	2.19E-07
PRR24	2.19E-07
C5AR1	2.19E-07
C5AR2	3.81E-07
DHX34	4.87E-07
MEIS3	3.31E-07
SLC8A2	3.31E-07
KPTN	3.31E-07
NAPA	3.31E-07
ZNF541	3.31E-07
RN7SL322P	2.35E-07
GLTSCR1	4.42E-07
EHD2	8.02E-07
GLTSCR2	8.02E-07
SNORD23|ENSG00000221803.1	6.80E-07

SEPW1	6.80E-07
TPRX1	6.80E-07
CRX	6.80E-07
TPRX2P	6.80E-07
SULT2A1	6.80E-07
BSPH1	9.80E-07
ELSPBP1	2.57E-06
CABP5	1.53E-06
PLA2G4C	1.53E-06
LIG1	3.91E-07
C19orf68	3.91E-07
ZNF114	4.45E-07
CARD8	4.69E-07
CCDC114	4.69E-07
EMP3	4.69E-07
TMEM143	4.69E-07
SYNGR4	4.69E-07
KDELR1	4.69E-07
GRIN2D	4.69E-07
GRWD1	4.69E-07
KCNJ14	4.69E-07
CYTH2	4.69E-07
LMTK3	2.60E-07
SULT2B1	2.60E-07
FAM83E	2.60E-07
SPACA4	2.60E-07
RPL18	2.60E-07
SPHK2	2.60E-07
DBP	4.69E-07
CA11	4.69E-07
SEC1P	4.69E-07
NTN5	4.69E-07
FUT2	3.07E-07
RN7SL345P	3.07E-07
MAMSTR	3.07E-07
RASIP1	3.07E-07
IZUMO1	3.07E-07
FUT1	3.07E-07
FGF21	3.07E-07
BCAT2	3.07E-07
HSD17B14	3.07E-07
PLEKHA4	3.07E-07
PPP1R15A	3.07E-07
TULP2	3.07E-07
NUCB1	3.07E-07
DHDH	2.05E-07
BAX	1.78E-07
FTL	1.78E-07
GYS1	1.78E-07
RUVBL2	2.73E-07
LHB	3.07E-07
CGB2	3.07E-07
CGB	3.07E-07
CGB1	2.65E-07
CGB5	2.65E-07
CGB7	2.65E-07
CGB8	2.65E-07
NTF4	2.65E-07

KCNA7	2.65E-07
RN7SL708P	2.65E-07
SNRNP70	2.65E-07
LIN7B	2.65E-07
C19orf73	2.65E-07
PPFIA3	2.65E-07
HRC	2.65E-07
TRPM4	2.65E-07
SLC6A16	2.65E-07
MIR4324	2.65E-07
CD37	2.65E-07
TEAD2	2.65E-07
DKKL1	2.65E-07
CCDC155	2.65E-07
PTH2	2.73E-07
SLC17A7	2.73E-07
PIH1D1	2.73E-07
ALDH16A1	2.73E-07
FLT3LG	2.73E-07
RPL13A	2.73E-07
SNORD32A	2.73E-07
SNORD33|ENSG00000199631.1	2.73E-07
SNORD34	2.73E-07
SNORD35A	2.73E-07
RPS11	2.73E-07
SNORD35B	2.73E-07
FCGRT	2.73E-07
MIR150	2.73E-07
RCN3	2.73E-07
NOSIP	2.73E-07
PRRG2	4.87E-07
PRR12	4.87E-07
RRAS	5.85E-07
SCAF1	5.85E-07
ADM5	5.85E-07
BCL2L12	5.85E-07
CPT1C	5.85E-07
IRF3	5.85E-07
MIR5088	5.85E-07
PRMT1	5.85E-07
TSKS	5.53E-07
AP2A1	5.53E-07
FUZ	7.68E-07
MED25	7.68E-07
PTOV1	7.68E-07
MIR4749	7.68E-07
PNKP	9.34E-07
AKT1S1	8.26E-07
ATF5	5.48E-07
IL4I1	5.48E-07
MIR4750	5.48E-07
MIR4751	5.48E-07
NUP62	5.48E-07
SIGLEC11	5.48E-07
SIGLEC16	5.48E-07
TBC1D17	5.48E-07
U3|ENSG00000221125.1	5.48E-07
VRK3	5.48E-07

ZNF473	5.48E-07
IZUMO2	8.45E-07
MYH14	4.96E-07
KCNC3	7.34E-07
NAPSB	7.34E-07
NR1H2	7.34E-07
NAPSA	7.34E-07
POLD1	7.34E-07
RN7SL324P	7.34E-07
SPIB	7.34E-07
MYBPC2	7.34E-07
FAM71E1	7.34E-07
EMC10	7.34E-07
JOSD2	6.19E-07
ASPDH	6.19E-07
LRRC4B	8.42E-07
SYT3	3.93E-07
C19orf81	3.05E-07
SHANK1	3.67E-07
CLEC11A	3.67E-07
GPR32	3.67E-07
ACPT	4.71E-07
C19orf48	4.71E-07
SNORD88A	4.71E-07
SNORD88B	4.71E-07
SNORD88C	4.71E-07
KLK1	4.71E-07
KLK15	5.60E-07
KLK2	5.60E-07
KLK3	5.60E-07
KLK4	5.60E-07
KLKP1	5.60E-07
KLK5	3.03E-07
KLK6	5.60E-07
KLK7	5.60E-07
KLK8	5.60E-07
KLK9	5.60E-07
KLK10	5.60E-07
KLK11	5.60E-07
KLK12	5.60E-07
KLK13	5.60E-07
KLK14	5.60E-07
CTU1	5.60E-07
SIGLEC7	5.60E-07
SIGLEC9	5.60E-07
SIGLEC17P	5.60E-07
SIGLEC22P	5.60E-07
CD33	3.63E-07
SIGLECL1	4.61E-07
IGLON5	4.34E-07
VSIG10L	4.34E-07
ETFB	4.34E-07
CLDND2	6.73E-07
NKG7	6.73E-07
LIM2	6.73E-07
SIGLEC10	6.73E-07
SIGLEC8	5.19E-07
CEACAM18	5.19E-07

SIGLEC12	5.19E-07
SIGLEC6	5.19E-07
ZNF175	5.19E-07
SIGLEC5	5.19E-07
SIGLEC14	2.67E-07
snoU13|ENSG00000238486.1	2.67E-07
LINC00085	2.67E-07
MIR125A	2.67E-07
MIR99B	2.67E-07
MIRLET7E	2.67E-07
HAS1	5.19E-07
FPR1	1.18E-06
FPR2	1.18E-06
FPR3	1.18E-06
HCCAT3	1.18E-06
ZNF350	1.18E-06
ZNF432	1.18E-06
ZNF577	1.18E-06
ZNF613	1.18E-06
ZNF614	1.18E-06
ZNF615	1.18E-06
ZNF616	1.18E-06
ZNF649	1.18E-06
ZNF841	1.18E-06
snoU13|ENSG00000238630.1	1.09E-06
ZNF836	1.42E-06
PPP2R1A	1.42E-06
ZNF766	7.44E-07
MIR643	7.44E-07
ZNF480	7.44E-07
ZNF610	8.23E-07
ZNF528	1.47E-06
ZNF534	1.47E-06
ZNF578	1.47E-06
ZNF880	1.47E-06
ZNF808	1.47E-06
ZNF701	1.14E-06
ZNF137P	1.14E-06
ZNF83	2.60E-06
ZNF28	1.40E-06
ZNF320	1.40E-06
ZNF321P	1.40E-06
ZNF468	1.40E-06
ZNF600	1.40E-06
ZNF611	1.40E-06
ZNF702P	7.91E-07
ZNF816	1.40E-06
ZNF888	1.40E-06
ZNF160	1.45E-06
ZNF347	1.45E-06
ZNF415	1.45E-06
ZNF665	1.45E-06
ZNF677	2.65E-06
VN1R2	2.65E-06
VN1R4	2.65E-06
FAM90A27P	1.78E-06
BIRC8	1.78E-06
FAM90A28P	1.20E-06

ZNF845	1.78E-06
ZNF525	1.78E-06
ZNF765	3.14E-06
TPM3P9	3.14E-06
ZNF761	3.14E-06
ZNF813	3.14E-06
ZNF331	1.07E-06
DPRX	1.58E-06
MIR1323	1.58E-06
MIR371A	1.58E-06
MIR371B	1.58E-06
MIR372	1.58E-06
MIR373	1.58E-06
MIR498	1.58E-06
MIR516A1	1.58E-06
MIR516A2	1.58E-06
MIR516B1	1.58E-06
MIR516B2	1.58E-06
MIR517A	1.58E-06
MIR517B	1.58E-06
MIR517C	1.58E-06
MIR518A1	1.58E-06
MIR518A2	1.58E-06
MIR518B	1.58E-06
MIR518C	1.58E-06
MIR518D	1.58E-06
MIR518E	1.58E-06
MIR518F	1.58E-06
MIR519A1	1.58E-06
MIR519A2	1.58E-06
MIR519B	1.58E-06
MIR519C	1.58E-06
MIR519D	1.58E-06
MIR519E	1.58E-06
MIR520A	1.58E-06
MIR520B	1.58E-06
MIR520C	1.58E-06
MIR520D	1.58E-06
MIR520E	1.58E-06
MIR520F	1.58E-06
MIR520G	1.58E-06
MIR520H	1.58E-06
MIR522	1.58E-06
MIR523	1.58E-06
MIR524	1.58E-06
MIR525	1.58E-06
MIR526A1	1.58E-06
MIR526A2	1.58E-06
MIR526B	1.58E-06
MIR527	1.58E-06
NLRP12	1.58E-06
RN7SL317P	1.58E-06
MYADM	6.82E-07
PRKCG	3.87E-07
CACNG7	3.50E-07
CACNG8	3.50E-07
CACNG6	4.38E-07
MIR935	3.50E-07

VSTM1	3.50E-07
TARM1	3.50E-07
OSCAR	3.50E-07
NDUFA3	3.50E-07
TFPT	3.50E-07
PRPF31	2.36E-07
CNOT3	2.67E-07
LENG1	2.67E-07
TMC4	2.08E-07
MBOAT7	2.08E-07
TSEN34	2.08E-07
RPS9	6.21E-07
LILRA3	7.64E-07
LILRA4	7.64E-07
LILRA5	7.64E-07
LILRA6	7.64E-07
LILRB2	7.64E-07
LILRB3	7.64E-07
LILRB5	7.64E-07
MIR4752	7.64E-07
LAIR1	9.77E-07
TTYH1	5.18E-07
LENG8	5.18E-07
CDC42EP5	5.18E-07
LENG9	5.18E-07
LAIR2	2.94E-07
KIR3DX1	2.45E-07
LILRA2	2.45E-07
LILRB1	2.92E-07
LILRA1	2.92E-07
LILRB4	2.45E-07
FCAR	2.45E-07
KIR2DL1	2.45E-07
KIR2DL3	2.45E-07
KIR2DL4	2.45E-07
KIR2DS4	2.45E-07
KIR3DL1	2.45E-07
KIR3DL2	2.45E-07
KIR3DL3	2.45E-07
NCR1	5.06E-07
NLRP7	5.06E-07
NLRP2	5.06E-07
GP6	5.06E-07
RDH13	4.52E-07
EPS8L1	4.52E-07
PPP1R12C	5.60E-07
TNNT1	3.88E-07
DNAAF3	3.88E-07
TNNI3	3.88E-07
snoU13|ENSG00000239137.1	3.88E-07
SYT5	3.88E-07
PTPRH	3.88E-07
TMEM86B	3.88E-07
PPP6R1	4.52E-07
HSPBP1	5.06E-07
BRSK1	5.06E-07
TMEM150B	5.06E-07
COX6B2	5.06E-07

SUV420H2	5.06E-07
FAM71E2	5.06E-07
IL11	5.06E-07
TMEM190	5.06E-07
TMEM238	5.06E-07
RPL28	5.06E-07
UBE2S	5.06E-07
SHISA7	5.06E-07
ISOC2	3.32E-07
ZNF628	4.15E-07
NAT14	4.15E-07
SSC5D	4.57E-07
SBK2	4.57E-07
SGK110	4.57E-07
ZNF579	4.57E-07
FIZ1	4.57E-07
ZNF524	4.57E-07
ZNF865	4.57E-07
ZNF784	4.57E-07
ZNF580	4.57E-07
ZNF581	4.57E-07
CCDC106	4.57E-07
U2AF2	5.65E-07
EPN1	1.04E-06
NLRP9	6.25E-07
RN7SKP109	5.13E-07
RFPL4AL1	5.13E-07
RFPL4A	5.13E-07
NLRP11	5.13E-07
NLRP4	5.13E-07
NLRP13	7.88E-07
NLRP5	1.12E-06
NLRP8	1.35E-06
ZNF787	1.12E-06
ZNF444	1.12E-06
GALP	2.20E-07
ZSCAN5B	2.20E-07
ZSCAN5A	2.20E-07
ZSCAN5C	2.20E-07
ZSCAN5D	2.20E-07
ZNF542	2.20E-07
ZNF582	2.20E-07
ZNF583	2.20E-07
ZNF667	1.42E-07
ZNF471	1.42E-07
ZFP28	1.42E-07
ZNF470	1.42E-07
ZNF71	1.42E-07
SMIM17	1.42E-07
ZNF835	3.11E-07
ZIM2	1.42E-07
PEG3	1.42E-07
MIMT1	1.42E-07
USP29	2.18E-07
DUXA	2.18E-07
U3|ENSG00000252683.1	2.18E-07
ZIM3	2.18E-07
ZNF264	1.35E-07

AURKC	1.35E-07
ZNF805	1.35E-07
ZNF460	2.35E-07
ZNF543	2.35E-07
ZNF304	2.35E-07
TRAPPC2P1	2.08E-07
ZNF547	1.31E-07
ZNF548	8.59E-08
ZNF17	8.59E-08
ZNF749	8.59E-08
VN1R1	1.06E-07
ZNF419	1.06E-07
ZNF772	1.06E-07
ZNF773	1.06E-07
ZNF549	5.77E-08
ZNF550	5.77E-08
ZNF416	5.77E-08
ZIK1	3.49E-08
ZNF530	2.33E-08
ZNF134	2.33E-08
ZNF211	2.33E-08
ZSCAN4	4.71E-08
ZNF551	4.71E-08
ZNF154	2.29E-08
ZNF671	2.53E-08
ZNF776	2.53E-08
ZNF586	2.53E-08
ZNF552	2.53E-08
ZNF587B	2.53E-08
ZNF814	2.53E-08
ZNF587	2.53E-08
ZNF417	4.71E-08
ZNF418	4.71E-08
ZNF256	2.53E-08
C19orf18	2.29E-08
ZNF606	2.29E-08
ZSCAN1	5.57E-08
ZNF135	5.57E-08
RN7SL526P	5.57E-08
ZSCAN18	5.57E-08
ZNF329	6.42E-08
ZNF274	6.42E-08
ZNF544	1.82E-07
ZNF8	1.82E-07
ZSCAN22	1.82E-07
A1BG	1.28E-07
ZNF497	1.82E-07
RNA5SP473	2.92E-07
CHMP2A	2.92E-07
MIR4754	2.92E-07
MZF1	2.92E-07
RN7SL525P	2.92E-07
RN7SL693P	2.92E-07
RPL23AP79	2.92E-07
RPS5	2.92E-07
SLC27A5	2.92E-07
TRIM28	2.92E-07
UBE2M	2.92E-07

ZBTB45	2.92E-07
ZNF132	2.92E-07
ZNF324B	2.92E-07
ZNF324	2.92E-07
ZNF446	2.92E-07
ZNF584	2.92E-07
ZNF837	2.92E-07
RASSF2	0.045425
SNORA31|ENSG00000252096.1	0.049483
snoU13|ENSG00000252058.1	0.049483
RN7SL514P	0.045425
RNA5SP474	0.045425
TMEM230	0.045425
PCNA	0.045425
SNORA26|ENSG00000212517.1	0.045425
CDS2	0.045425
PROKR2	0.040158
GPCPD1	0.029498
LINC00654	0.029498
LINC00658	0.029498
C20orf196	0.04925
LRRN4	0.04316
BMP2	0.02904
snoR26	0.023077
RN7SL547P	0.044804
HAO1	0.018901
TMX4	0.018901
PLCB1	0.025381
RNU105B	0.016271
PLCB4	0.031143
LAMP5	0.011014
PAK7	0.011262
ANKEF1	0.021395
SNAP25	0.02412
MKKS	0.02412
SLX4IP	0.033629
JAG1	0.033629
LINC00687	0.013303
RN7SKP111	0.013303
BTBD3	0.013303
SPTLC3	0.022485
ISM1	0.022815
TASP1	0.016234
ESF1	0.047049
SEL1L2	0.036923
MACROD2	0.039552
RN7SL864P	0.018958
FLRT3	0.017133
KIF16B	0.031685
SNRPB2	0.026374
OTOR	0.011559
U3|ENSG00000212165.1	0.005494
PCSK2	0.029175
BFSP1	0.049147
DSTN	0.037666
RRBP1	0.028454
BANF2	0.037742
RALGAPA2	0.027781

CST7	0.043068
APMAP	0.032903
ACSS1	0.032903
VSX1	0.032596
ENTPD6	0.032183
PYGB	0.02476
ABHD12	0.02476
GINS1	0.03273
MIR663A	0.026233
C20orf112	0.023614
C20orf203	0.031037
COMMD7	0.043669
DNMT3B	0.043669
MAPRE1	0.04258
ZNF341	0.032592
CHMP4B	0.048045
MIR4755	0.049278
EIF2S2	0.049278
ZHX3	0.037387
RN7SL615P	0.037387
LPIN3	0.033772
EMILIN3	0.033772
CHD6	0.028852
SNORA26|ENSG00000212224.1	0.04998
PTPRT	0.049576
RN7SKP100	0.044708
RN7SL666P	0.043403
SRSF6	0.014846
L3MBTL1	0.028775
SGK2	0.030376
IFT52	0.012938
MYBL2	0.012938
GTSF1L	0.010616
TOX2	0.007526
RN7SL443P	0.020064
JPH2	0.021173
OSER1	0.025551
GDAP1L1	0.037035
FITM2	0.045749
R3HDML	0.045749
HNF4A	0.045749
MIR3646	0.040938
C20orf62	0.040938
TTPAL	0.031533
SERINC3	0.032894
PKIG	0.037475
ADA	0.037475
WISP2	0.024901
KCNK15	0.031533
RIMS4	0.02918
RN7SL31P	0.018821
YWHAB	0.014244
PABPC1L	0.014244
TOMM34	0.014244
STK4	0.018179
KCNS1	0.018179
WFDC5	0.018179
WFDC12	0.018179

PI3	0.017023
SEMG1	0.017023
SEMG2	0.017023
SLPI	0.022676
MATN4	0.03185
RBPJL	0.03185
SDC4	0.028105
SYS1	0.029099
TP53TG5	0.029099
DBNDD2	0.028105
PIGT	0.028105
WFDC2	0.028105
SPINT3	0.029099
WFDC6	0.029099
EPPIN	0.039228
WFDC8	0.04768
TP53RK	0.04752
SLC2A10	0.038189
RN7SKP33	0.048158
EYA2	0.019877
ZMYND8	0.048087
NCOA3	0.033278
SULF2	0.012725
RNA5SP486	0.011451
SNORD36|ENSG00000252227.1	0.025231
LINC00494	0.035089
PREX1	0.011272
ARFGEF2	0.018751
CSE1L	0.014459
STAU1	0.014582
DDX27	0.02215
ZNFX1	0.020213
SNORD12B	0.020213
SNORD12C	0.020213
SNORD12	0.020213
ZFAS1	0.020213
KCNB1	0.040569
PTGIS	0.031953
B4GALT5	0.013561
RN7SL197P	0.013561
SLC9A8	0.01047
SPATA2	0.006291
RNF114	0.006248
snoU13|ENSG00000239157.1	0.008054
SNAI1	0.006248
LINC00651	0.006248
TMEM189	0.011616
UBE2V1	0.006248
CEBPB	0.011616
RN7SL636P	0.019802
PTPN1	0.019139
RN7SL672P	0.019139
FAM65C	0.025115
MIR645	0.019139
PARD6B	0.016745
BCAS4	0.024138
ADNP	0.008054
DPM1	0.005384

MOCS3	0.005384
KCNG1	0.006325
NFATC2	0.01852
MIR3194	0.012531
ATP9A	0.019885
SALL4	0.019885
RN7SL603P	0.035032
ZFP64	0.034713
TSHZ2	0.034056
ZNF217	0.028704
BCAS1	0.011444
MIR4756	0.006877
CYP24A1	0.010732
PFDN4	0.011271
DOK5	0.01018
RNU4ATAC7P	0.009751
CBLN4	0.014034
RNA5SP487	0.014763
MC3R	0.022234
FAM210B	0.022234
AURKA	0.021108
CSTF1	0.021108
CASS4	0.021108
RTFDC1	0.018872
snoU13|ENSG00000238294.1	0.018872
GCNT7	0.018843
FAM209A	0.018843
FAM209B	0.018965
U3|ENSG00000252536.1	0.020237
TFAP2C	0.020237
RN7SL170P	0.020991
BMP7	0.022823
MIR4325	0.022823
SPO11	0.022823
RAE1	0.024418
MTRNR2L3	0.027427
RBM38	0.024776
CTCFL	0.042815
PCK1	0.042815
ZBP1	0.049832
PMEPA1	0.039637
MIR4532	0.042023
C20orf85	0.027
ANKRD60	0.017732
PPP4R1L	0.015828
RAB22A	0.014856
VAPB	0.014377
APCDD1L	0.012983
MGC4294	0.012095
STX16	0.014499
NPEPL1	0.014499
MIR296	0.021235
MIR298	0.021235
GNAS	0.022758
NELFCD	0.018511
CTSZ	0.018511
TUBB1	0.018511
ATP5E	0.018511

SLMO2	0.018511
ZNF831	0.018511
EDN3	0.014956
PHACTR3	0.009625
SYCP2	0.006065
FAM217B	0.003605
PPP1R3D	0.003605
CDH26	0.002568
C20orf197	0.002568
MIR646	0.003766
MIR4533	0.006628
MIR548AG2	0.006752
CDH4	0.001964
MIR1257	0.00465
TAF4	0.005091
LSM14B	0.003788
PSMA7	0.003788
SS18L1	0.002701
MTG2	0.002744
HRH3	0.002715
OSBPL2	0.001873
ADRM1	0.001873
LAMA5	0.001429
MIR4758	0.001429
RPS21	0.001462
CABLES2	0.001977
RBBP8NL	0.001977
GATA5	0.002735
C20orf166	0.003541
MIR133A2	0.004464
SLCO4A1	0.014372
LINC00686	0.005932
NTSR1	0.004763
LINC00659	0.004602
MRGBP	0.004602
OGFR	0.004602
COL9A3	0.004602
TCFL5	0.003878
DIDO1	0.004348
GID8	0.004348
SLC17A9	0.004348
BHLHE23	0.004787
LINC00029	0.004787
HAR1A	0.004706
HAR1B	0.004706
YTHDF1	0.00509
BIRC7	0.006649
MIR3196	0.006649
NKAIN4	0.005813
ARFGAP1	0.00776
MIR4326	0.00776
COL20A1	0.00776
CHRNA4	0.00776
KCNQ2	0.023221
EEF1A2	0.023221
PPDPF	0.023221
C20orf195	0.023221
PTK6	0.023221

SRMS	0.023221
HELZ2	0.021501
GMEB2	0.019929
ABHD16B	0.019929
ARFRP1	0.019929
C20ORF135	0.019929
C20orf201	0.019929
DNAJC5	0.019929
LIME1	0.019929
LINC00176	0.019929
MIR1914	0.019929
MIR647	0.019929
MYT1	0.019929
NPBWR2	0.019929
OPRL1	0.019929
PCMTD2	0.019929
PRPF6	0.019929
RGS19	0.019929
RTEL1	0.019929
SAMD10	0.019929
SLC2A4RG	0.019929
SOX18	0.019929
STMN3	0.019929
TCEA2	0.019929
TNFRSF6B	0.019929
TPD52L2	0.019929
UCKL1	0.019929
ZBTB46	0.019929
ZGPAT	0.019929
ZNF512B	0.019929
7SK|ENSG00000232512.2	0.019924
RNA5SP489	0.020458
LINC00158	0.047125
snoU13|ENSG00000238314.1	0.028772
MIR155HG	0.037722
LINC00515	0.037722
MRPL39	0.037722
JAM2	0.010381
ATP5J	0.012989
GABPA	0.012989
APP	0.012989
CYYR1	0.023331
ADAMTS1	0.029316
ADAMTS5	0.021922
MIR4759	0.030202
LINC00113	0.048693
LINC00314	0.048172
LINC00161	0.008499
N6AMT1	0.001954
LTN1	0.002266
RWDD2B	0.001798
USP16	0.001798
CCT8	0.00192
MAP3K7CL	0.002512
U3|ENSG00000212479.1	0.004284
BACH1	0.016374
LINC00189	0.012771
snoU13|ENSG00000239171.1	0.004388

GRIK1	0.015735
MIR4327	0.041418
TIAM1	0.001077
SOD1	0.001823
SCAF4	0.00108
SNORA81|ENSG00000238390.1	0.001823
HUNK	0.002102
LINC00159	0.007293
MIS18A	0.007813
MRAP	0.009794
URB1	0.010389
RN7SL109P	0.010691
SNORA80	0.010691
C21orf119	0.010691
EVA1C	0.007986
SNORA33|ENSG00000252045.1	0.010442
RNA5SP490	0.010442
LINC00846	0.010442
TCP10L	0.010442
C21ORF59	0.028256
C21orf59	0.028256
SYNJ1	0.028256
PAXBP1	0.028256
C21orf49	0.049766
C21orf62	0.049766
SNORA70|ENSG00000207098.1	0.040946
IFNAR1	0.048594
GART	0.019154
SON	0.011179
DONSON	0.019154
CRYZL1	0.014672
ITSN1	0.019425
ATP5O	0.017948
LINC00649	0.014089
RN7SL740P	0.017948
MRPS6	0.013974
SLC5A3	0.013974
LINC00310	0.013974
KCNE1	0.010435
KCNE2	0.010435
RCAN1	0.00693
SMIM11	0.010435
SNORA11|ENSG00000221398.1	0.010435
CLIC6	0.0038
LINC00160	0.002779
RUNX1	0.021568
MIR802	0.010118
FKSG68	0.010068
SETD4	0.009966
CBR1	0.009966
CBR3	0.014444
DOPEY2	0.018597
RN7SL73P	0.014444
snoU13|ENSG00000238851.1	0.014444
MORC3	0.047406
CHAF1B	0.039621
CLDN14	0.041662
BACE2	0.045185

PKNOX1	0.049528
CBS	0.044065
LRRC3	0.044097
LRRC3DN	0.044097
TSPEAR	0.030325
C21orf90	0.044097
CCT8L2	0.001864
HSFY1P1	0.001864
KCNMB3P1	0.001864
OR11H1	0.001864
POTEH	0.001864
TPTEP1	0.001864
XKR3	0.001864
GAB4	0.001331
CECR7	0.00143
IL17RA	0.001864
CECR6	0.001864
CECR5	0.002395
CECR1	0.001823
CECR3	0.001099
CECR9	0.001823
RN7SL843P	0.001289
CECR2	0.003231
SLC25A18	0.001823
ATP6V1E1	0.001823
BCL2L13	0.001958
snoU13|ENSG00000251737.1	0.001958
BID	0.003306
LINC00528	0.00186
MICAL3	0.00172
MIR648	0.001316
PEX26	0.000124
TUBA8	0.000124
AIFM3	2.11E-05
ARVCF	2.11E-05
BCRP2	2.11E-05
C22orf29	2.11E-05
C22orf39	2.11E-05
CCDC116	2.11E-05
CDC45	2.11E-05
CLDN5	2.11E-05
CLTCL1	2.11E-05
COMT	2.11E-05
CRKL	2.11E-05
DGCR14	2.11E-05
DGCR2	2.11E-05
DGCR5	2.11E-05
DGCR6L	2.11E-05
DGCR6	2.11E-05
DGCR8	2.11E-05
FAM230A	2.11E-05
FAM230B	2.11E-05
FAM230C	2.11E-05
GGT2	2.11E-05
GGT3P	2.11E-05
GGTLC3	2.11E-05
GNB1L	2.11E-05
GP1BB	2.11E-05

GSC2	2.11E-05
HIC2	2.11E-05
HIRA	2.11E-05
KLHL22	2.11E-05
LZTR1	2.11E-05
MED15	2.11E-05
MIR1286	2.11E-05
MIR1306	2.11E-05
MIR130B	2.11E-05
MIR185	2.11E-05
MIR301B	2.11E-05
MIR3618	2.11E-05
MIR4761	2.11E-05
MIR649	2.11E-05
MRPL40	2.11E-05
P2RX6P	2.11E-05
P2RX6	2.11E-05
PI4KAP1	2.11E-05
PI4KAP2	2.11E-05
PI4KA	2.11E-05
POM121L4P	2.11E-05
POM121L7	2.11E-05
PPIL2	2.11E-05
PRODH	2.11E-05
RANBP1	2.11E-05
RIMBP3B	2.11E-05
RIMBP3C	2.11E-05
RIMBP3	2.11E-05
RN7SKP131	2.11E-05
RN7SKP221	2.11E-05
RN7SKP63	2.11E-05
RN7SL168P	2.11E-05
RN7SL280P	2.11E-05
RN7SL389P	2.11E-05
RN7SL812P	2.11E-05
RTN4R	2.11E-05
SCARF2	2.11E-05
SCARNA17|ENSG00000252020.1	2.11E-05
SCARNA17|ENSG00000252143.1	2.11E-05
SCARNA17|ENSG00000252571.1	2.11E-05
SCARNA18|ENSG00000252024.1	2.11E-05
SCARNA18|ENSG00000252314.1	2.11E-05
SCARNA18|ENSG00000252605.1	2.11E-05
SDF2L1	2.11E-05
5-Sep	2.11E-05
SERPIND1	2.11E-05
SLC25A1	2.11E-05
SLC7A4	2.11E-05
SMPD4P1	2.11E-05
SNAP29	2.11E-05
SNORA15|ENSG00000251940.1	2.11E-05
SNORA77|ENSG00000264346.1	2.11E-05
TANGO2	2.11E-05
TBX1	2.11E-05
THAP7	2.11E-05
TMEM191A	2.11E-05
TMEM191C	2.11E-05
TRMT2A	2.11E-05

TSSK2	2.11E-05
TUBA3FP	2.11E-05
TXNRD2	2.11E-05
UBE2L3	2.11E-05
UFD1L	2.11E-05
USP18	2.11E-05
USP41	2.11E-05
YDJC	2.11E-05
YPEL1	2.11E-05
ZDHHC8	2.11E-05
ZNF74	2.11E-05
snoU13|ENSG00000252402.1	2.11E-05
snoU13|ENSG00000252799.1	2.11E-05
snoU13|ENSG00000271796.1	2.11E-05
MAPK1	0.000347
RNA5SP493	0.000129
PPM1F	0.000414
BCR	0.000169
CES5AP1	0.000169
GGTLC2	0.000169
GNAZ	0.000169
IGLC1	0.000169
IGLC2	0.000169
IGLC3	0.000169
IGLC7	0.000169
IGLJ1	0.000169
IGLJ2	0.000169
IGLJ3	0.000169
IGLJ4	0.000169
IGLJ5	0.000169
IGLJ6	0.000169
IGLJ7	0.000169
IGLL5	0.000169
MIR650	0.000169
POM121L1P	0.000169
PRAMEF24P	0.000169
PRAME	0.000169
RAB36	0.000169
RN7SL263P	0.000169
RTDR1	0.000169
TOP3B	0.000169
VPREB1	0.000169
ZDHHC8P1	0.000169
ZNF280A	0.000169
ZNF280B	0.000169
snoU13|ENSG00000239066.1	0.000169
C22orf43	0.000876
GUSBP11	0.000876
IGLL1	0.000876
RGL4	0.001266
VPREB3	0.001744
ZNF70	0.001744
C22orf15	0.00164
CHCHD10	0.00164
MMP11	0.00164
SMARCB1	0.001744
DERL3	0.001744
SLC2A11	0.001392

RN7SL268P	0.001392
MIF	0.001392
CABIN1	0.000506
DDTL	0.001223
DDT	0.001223
GSTT1	0.001223
GSTT2B	0.001223
GSTT2	0.001223
GSTTP2	0.001223
SUSD2	0.000693
GGT5	0.000693
POM121L9P	0.000981
SPECC1L	0.000981
ADORA2A	0.000981
UPB1	0.000981
GUCD1	0.000723
SNRPD3	0.000723
FAM211B	0.000723
GGT1	0.000723
PIWIL3	0.000953
SGSM1	0.000911
SNORD56|ENSG00000199783.1	0.000911
TMEM211	0.000911
KIAA1671	0.00111
CRYBB3	0.001333
CRYBB2	0.001273
ADRBK2	0.000559
CRYBB2P1	0.000741
LRP5L	0.000741
RNA5SP494	0.000292
MYO18B	0.000296
RN7SKP169	0.000154
SEZ6L	8.19E-05
RNA5SP495	9.36E-05
ASPHD2	8.70E-05
HPS4	8.70E-05
SRRD	8.70E-05
TFIP11	8.70E-05
TPST2	8.70E-05
MIR548J	8.80E-05
CRYBB1	0.000126
CRYBA4	0.000126
MIAT	0.000126
MN1	0.000871
PITPNB	0.000603
TTC28	0.000529
RN7SL757P	0.00044
SNORD42|ENSG00000201209.1	0.00044
RN7SL162P	0.001631
CHEK2	0.001191
HSCB	0.001196
CCDC117	0.001196
XBP1	0.000995
ZNRF3	0.000735
C22orf31	0.001084
KREMEN1	0.000685
EMID1	0.000747
RHBDD3	0.000747

EWSR1	0.000747
GAS2L1	0.000747
RASL10A	0.000747
AP1B1	0.000292
SNORD125	0.00064
RFPL1S	0.000399
RFPL1	0.000399
NEFH	0.000417
THOC5	0.000417
NIPSNAP1	0.000574
NF2	0.000601
CABP7	0.000268
ZMAT5	0.000268
UQCR10	0.000386
ASCC2	0.000386
MTMR3	0.000386
HORMAD2	0.001118
LIF	0.001007
MGC20647	0.001007
OSM	0.001007
GATSL3	0.000734
TBC1D10A	0.000734
SF3A1	0.001033
CCDC157	0.001033
RNF215	0.000938
SEC14L2	0.000938
KIAA1658	0.001311
MTFP1	0.001311
SEC14L3	0.001311
SEC14L4	0.001503
SEC14L6	0.001845
GAL3ST1	0.001845
PES1	0.001463
TCN2	0.001245
SLC35E4	0.002321
DUSP18	0.002321
OSBP2	0.001646
MIR3200	0.003254
MORC2	0.001583
TUG1	0.001207
RN7SL633P	0.000526
SMTN	0.00061
INPP5J	0.000882
PLA2G3	0.000882
MIR3928	0.000882
RNF185	0.000882
LIMK2	0.000882
PIK3IP1	0.000882
RNA5SP496	0.000882
PATZ1	0.000882
DRG1	0.000454
EIF4ENIF1	0.000454
SFI1	0.000428
PISD	0.000392
PRR14L	0.000392
DEPDC5	0.000129
RN7SL20P	0.000178
C22orf24	0.000175

YWHAH	0.000175
snoU13|ENSG00000238910.1	0.000175
RN7SL305P	0.000183
SLC5A1	0.000237
AP1B1P1	0.000375
C22orf42	0.000375
RFPL2	0.000375
SLC5A4	0.000539
RFPL3	0.000539
RFPL3S	0.000539
RTCB	0.000539
BPIFC	0.000539
FBXO7	0.000539
SYN3	0.000516
RNA5SP497	0.000732
TIMP3	0.000479
LARGE	0.000106
MIR4764	0.000628
SNORA76|ENSG00000253007.2	0.00016
ISX	0.0004
HMGXB4	0.000649
TOM1	0.000649
MIR3909	0.000649
HMOX1	0.000591
MCM5	0.000636
RASD2	0.000636
APOL6	0.000617
MB	0.000617
APOL5	0.000617
RBFOX2	0.000361
APOL3	0.000374
APOL4	0.000465
APOL1	0.000658
APOL2	0.000658
MYH9	0.001756
RN7SL349P	0.000658
TXN2	0.000216
FOXRED2	0.000216
EIF3D	0.000339
CACNG2	0.000626
IFT27	0.001231
PVALB	0.002338
NCF4	0.002621
CSF2RB	0.002621
snoU13|ENSG00000239056.1	0.002621
TEX33	0.002701
TST	0.003753
MPST	0.003753
KCTD17	0.003753
RN7SKP214	0.003753
TMPRSS6	0.002739
IL2RB	0.002739
C1QTNF6	0.002739
SSTR3	0.002739
RAC2	0.002739
CYTH4	0.003759
ELFN2	0.00267
MFNG	0.001937

CARD10	0.001937
CDC42EP1	0.001409
LGALS2	0.001409
GGA1	0.000887
SH3BP1	0.000887
PDXP	0.000933
RN7SL385P	0.000933
LGALS1	0.000933
NOL12	0.000933
TRIOBP	0.000874
snoU13|ENSG00000238569.1	0.001034
GCAT	0.001034
H1F0	0.001034
ANKRD54	0.001034
GALR3	0.001034
MIR658	0.001034
EIF3L	0.000889
MIR659	0.001034
MICALL1	0.000889
C22orf23	0.000889
POLR2F	0.000889
SOX10	0.001042
MIR4534	0.001042
PICK1	0.000889
SLC16A8	0.000889
BAIAP2L2	0.000889
PLA2G6	0.000889
MAFF	0.001037
TMEM184B	0.001037
RN7SL704P	0.001037
CSNK1E	0.001097
KCNJ4	0.001251
KDELR3	0.001251
DDX17	0.001471
DMC1	0.001567
FAM227A	0.001567
CBY1	0.001567
TOMM22	0.001567
JOSD1	0.001531
GTPBP1	0.001531
SUN2	0.001584
DNAL4	0.001584
NPTXR	0.001584
CBX6	0.001584
APOBEC3A	0.002881
APOBEC3B	0.002881
APOBEC3C	0.002881
APOBEC3D	0.002881
APOBEC3F	0.002881
APOBEC3G	0.001968
APOBEC3H	0.001968
CBX7	0.002698
PDGFB	0.001968
RPL3	0.002881
SNORD83B	0.002881
SNORD83A	0.002881
SNORD43|ENSG00000263764.1	0.002881
SYNGR1	0.002881

TAB1	0.002881
MGAT3	0.002881
SMCR7L	0.002881
ATF4	0.001628
RPS19BP1	0.001628
CACNA1I	0.001917
ENTHD1	0.001422
RN7SKP210	0.001425
GRAP2	0.001032
FAM83F	0.00089
TNRC6B	0.000372
ADSL	0.002107
SGSM3	0.001791
MKL1	0.001198
MCHR1	0.0022
SLC25A17	0.000871
MIR4766	0.001093
ST13	0.001093
DNAJB7	0.001127
XPNPEP3	0.001093
snoU13|ENSG00000238887.1	0.001093
RBX1	0.001093
EP300	0.000698
MIR1281	0.000919
L3MBTL2	0.000898
CHADL	0.001494
RANGAP1	0.001494
ZC3H7B	0.0008
TEF	0.001041
TOB2	0.00126
PHF5A	0.001797
ACO2	0.003221
POLR3H	0.001093
CSDC2	0.001042
PMM1	0.001042
DESI1	0.001042
XRCC6	0.000608
NHP2L1	0.000624
C22orf46	0.000624
MEI1	0.000624
RNU6ATAC22P	0.000624
CCDC134	0.000925
SREBF2	0.001113
CENPM	0.001113
LINC00634	0.001113
MIR33A	0.001113
MIR378I	0.001113
3-Sep	0.001113
SHISA8	0.001113
TNFRSF13C	0.001113
WBP2NL	0.001867
NAGA	0.001319
FAM109B	0.001319
snoU13|ENSG00000238498.1	0.001319
SMDT1	0.001319
NDUFA6	0.001319
CYP2D6	0.001653
CYP2D7P1	0.001653

TCF20	0.001873
NFAM1	0.001873
POLDIP3	0.001824
RN7SKP80	0.001873
RRP7A	0.001873
RRP7B	0.001873
SERHL2	0.001873
SERHL	0.001873
RNU12	0.001824
CYB5R3	0.001827
ATP5L2	0.001824
A4GALT	0.002253
ARFGAP3	0.001107
PACSIN2	0.001078
TTLL1	0.000666
BIK	0.000669
MCAT	0.000669
TSPO	0.000669
TTLL12	0.000669
SCUBE1	0.000669
MPPED1	0.000639
EFCAB6	0.000439
SULT4A1	0.000439
PNPLA5	0.00052
PNPLA3	0.00052
SAMM50	0.00052
PARVB	0.00052
PARVG	0.000286
KIAA1644	0.000286
LDOC1L	0.000171
LINC00207	0.000165
LINC00229	0.000233
PRR5	0.000228
ARHGAP8	0.000159
PHF21B	0.000144
NUP50	0.00016
KIAA0930	0.00016
MIR1249	0.00016
UPK3A	0.00016
FAM118A	0.000139
SMC1B	9.66E-05
RIBC2	9.66E-05
FBLN1	4.23E-05
ATXN10	3.05E-05
MIR4762	0.000102
WNT7B	4.80E-05
LINC00899	7.20E-05
C22orf26	7.20E-05
FLJ27365	4.89E-05
MIR3619	7.20E-05
MIR4763	7.20E-05
MIRLET7A3	7.20E-05
MIRLET7B	7.20E-05
PPARA	4.80E-05
CDPF1	2.17E-05
PKDREJ	2.17E-05
TTC38	2.17E-05
GTSE1	1.39E-05

TRMU	9.12E-06
CELSR1	3.78E-06
GRAMD4	5.83E-06
CERK	8.64E-06
TBC1D22A	4.81E-06
LINC00898	7.97E-06
MIR3201	2.89E-05
FAM19A5	1.32E-05
MIR4535	8.92E-06
ACR	1.34E-05
ADM2	1.34E-05
ALG12	1.34E-05
ARSA	1.34E-05
BRD1	1.34E-05
C22orf34	1.34E-05
CHKB	1.34E-05
CPT1B	1.34E-05
CRELD2	1.34E-05
DENND6B	1.34E-05
HDAC10	1.34E-05
IL17REL	1.34E-05
KLHDC7B	1.34E-05
LMF2	1.34E-05
MAPK11	1.34E-05
MAPK12	1.34E-05
MAPK8IP2	1.34E-05
MIOX	1.34E-05
MIR3667	1.34E-05
MLC1	1.34E-05
MOV10L1	1.34E-05
NCAPH2	1.34E-05
ODF3B	1.34E-05
PANX2	1.34E-05
PIM3	1.34E-05
PLXNB2	1.34E-05
PPP6R2	1.34E-05
RABL2B	1.34E-05
RN7SKP252	1.34E-05
RN7SL500P	1.34E-05
SBF1	1.34E-05
SCO2	1.34E-05
SHANK3	1.34E-05
SYCE3	1.34E-05
TRABD	1.34E-05
TTLL8	1.34E-05
TUBGCP6	1.34E-05
TYMP	1.34E-05
ZBED4	1.34E-05
KLHL15	0.044579
ZFX	0.044579
PCYT1B	0.039943
POLA1	0.037876
SCARNA23	0.037876
ATP6AP2	0.019962
MED14	0.045712
CPXCR1	0.021581
TGIF2LX	0.021624
PABPC5	0.003919

PCDH11X	0.003515
SNORA25|ENSG00000252296.1	0.030667
PCDH19	0.035477
TNMD	0.026966
TSPAN6	0.026966
SRPX2	0.026966
SYTL4	0.026966
CSTF2	0.019436
SNORA9|ENSG00000202231.1	0.019436
NOX1	0.019436
ARL13A	0.019989
TMEM35	0.019989
TRMT2B	0.019989
XKRX	0.019989
CENPI	0.019989
DRP2	0.018328
TAF7L	0.014652
BTK	0.014652
TIMM8A	0.014652
RPL36A	0.014652
GLA	0.014652
HNRNPH2	0.014652
ARMCX4	0.014473
ARMCX1	0.014473
ARMCX6	0.014473
ARMCX3	0.014473
ARMCX2	0.013142
NXF5	0.005282
ZMAT1	0.005282
TCEAL2	0.005282
TCEAL6	0.005938
BEX5	0.005938
TCP11X3P	0.005938
NXF2	0.005938
TCP11X1	0.005938
NXF2B	0.008497
TCP11X2	0.008497
TMSB15A	0.006677
NXF4	0.006677
ARMCX5	0.006677
GPRASP1	0.006677
GPRASP2	0.006677
BHLHB9	0.006677
LINC00630	0.004539
RAB40AL	0.004539
BEX1	0.00338
NXF3	0.002303
BEX4	0.004421
TCEAL8	0.004421
TCEAL5	0.004421
BEX2	0.004421
TCEAL7	0.004421
WBP5	0.004421
NGFRAP1	0.004421
RAB40A	0.002303
TCEAL4	0.002303
TCEAL3	0.002303
TCEAL1	0.002303

MORF4L2	0.00541
GLRA4	0.00541
TMEM31	0.00541
PLP1	0.002303
RAB9B	0.00338
RNA5SP511	0.00338
TMSB15B	0.00338
H2BFM	0.00338
H2BFWT	0.00338
SLC25A53	0.00338
ZCCHC18	0.00338
FAM199X	0.00338
ESX1	0.00333
IL1RAPL2	0.008646
TEX13A	0.006785
NRK	0.008192
SERPINA7	0.005386
MUM1L1	0.006962
CXorf57	0.004143
MIR548AN	0.004143
RNF128	0.004143
TBC1D8B	0.004143
MORC4	0.004143
CLDN2	0.004143
RIPPLY1	0.004143
NUP62CL	0.004143
PIH1D3	0.004143
RBM41	0.004143
FRMPD3	0.004143
PRPS1	0.005149
TSC22D3	0.005149
NCBP2L	0.005149
MID2	0.005352
TEX13B	0.005352
VSIG1	0.005352
PSMD10	0.005149
ATG4A	0.005149
COL4A6	0.005149
COL4A5	0.010389
IRS4	0.010389
GUCY2F	0.008825
NXT2	0.005282
KCNE1L	0.005282
ACSL4	0.004967
TMEM164	0.005222
MIR652	0.005222
MIR3978	0.005222
AMMECR1	0.006538
SNORD96B	0.005222
GNG5P2	0.006538
RGAG1	0.006538
TDGF1P3	0.006538
CHRDL1	0.003628
PAK3	0.007723
CAPN6	0.006387
DCX	0.006387
LINC00890	0.006937
RN7SL661P	0.006937

ALG13	0.006937
RNA5SP512	0.006937
TRPC5	0.006937
TRPC5OS	0.006937
ZCCHC16	0.007226
LHFPL1	0.007226
AMOT	0.007226
MIR4329	0.007226
RN7SL266P	0.007226
snoU13|ENSG00000238811.1	0.007487
U3|ENSG00000201674.1	0.010871
RN7SL93P	0.010871
SNORD30	0.010871
HTR2C	0.010186
SNORA35|ENSG00000208839.1	0.010871
MIR764	0.010871
MIR1912	0.010871
MIR1298	0.010871
MIR1911	0.010871
MIR448	0.010871
IL13RA2	0.010186
LRCH2	0.010186
LUZP4	0.010186
RBMXL3	0.010186
SNORA35|ENSG00000271907.1	0.010186
SNORA64|ENSG00000252441.1	0.016077
PLS3	0.019021
RN7SL712P	0.015121
AGTR2	0.010847
SLC6A14	0.016257
CXorf61	0.016257
KLHL13	0.015077
WDR44	0.017152
MIR1277	0.017152
DOCK11	0.010934
IL13RA1	0.010934
SNORA35|ENSG00000239182.1	0.010934
ZCCHC12	0.010934
LONRF3	0.012193
KIAA1210	0.01178
PGRMC1	0.012193
RN7SL118P	0.008956
SLC25A43	0.008956
SLC25A5	0.008956
CXorf56	0.004705
UBE2A	0.004705
NKRF	0.004705
6-Sep	0.004705
MIR766	0.004705
SOWAHD	0.004705
RPL39	0.004705
SNORA69|ENSG00000206622.1	0.004705
UPF3B	0.008956
NDUFA1	0.008956
RNF113A	0.008956
AKAP14	0.012193
NKAP	0.016864
RHOXF2B	0.006543

RHOXF1	0.006543
NKAPP1	0.007128
RHOXF2	0.007128
ZBTB33	0.007128
TMEM255A	0.007128
ATP1B4	0.007128
LAMP2	0.006401
CUL4B	0.006401
snoU13|ENSG00000272179.1	0.006401
MCTS1	0.006401
C1GALT1C1	0.006401
CT47A10	0.003791
CT47A11	0.003791
CT47A12	0.003791
CT47A1	0.003791
CT47A2	0.003791
CT47A3	0.003791
CT47A4	0.003791
CT47A5	0.003791
CT47A6	0.003791
CT47A7	0.003791
CT47A8	0.003791
CT47A9	0.003791
CT47B1	0.003791
GLUD2	0.003791
MIR3672	0.008015
U3|ENSG00000212321.1	0.003749
GRIA3	0.007957
THOC2	0.007345
RN7SL29P	0.007345
XIAP	0.007345
STAG2	0.004335
snoU13|ENSG00000239058.1	0.005616
SNORA40|ENSG00000252693.1	0.005616
SH2D1A	0.003502
TENM1	0.013845
snoU13|ENSG00000238626.1	0.008058
DCAF12L2	0.000918
DCAF12L1	0.001846
CXorf64	0.000993
ACTRT1	0.002507
RNA5SP513	0.002702
SMARCA1	0.00194
OCRL	0.003068
APLN	0.003381
XPNPEP2	0.004066
SASH3	0.004066
ZDHHC9	0.004066
UTP14A	0.004066
BCORL1	0.004729
ELF4	0.004729
AIFM1	0.003026
RAB33A	0.004767
ZNF280C	0.004767
SLC25A14	0.007217
GPR119	0.007217
RBMX2	0.007217
FAM45B	0.007217

ENOX2	0.010466
ARHGAP36	0.010466
RN7SL191P	0.010466
IGSF1	0.008437
OR13H1	0.008437
RNA5SP514	0.006185
MST4	0.006185
FRMD7	0.006185
RAP2C	0.009101
MBNL3	0.014099
HS6ST2	0.005688
USP26	0.005267
SNORA8|ENSG00000207100.1	0.005267
TFDP3	0.002611
GPC4	0.002435
GPC3	0.003093
MIR106A	0.002787
MIR18B	0.002787
MIR19B2	0.002787
MIR20B	0.002787
MIR363	0.002787
MIR92A2	0.002787
CCDC160	0.003093
PHF6	0.006155
HPRT1	0.008618
MIR450A1	0.003468
MIR450A2	0.003468
MIR450B	0.003468
MIR503HG	0.003468
MIR542	0.003468
MIR503	0.003468
LINC00629	0.003468
PLAC1	0.003468
FAM122B	0.003468
FAM122C	0.003468
MOSPD1	0.003468
SMIM10	0.004957
FAM127C	0.004957
FAM127A	0.004957
FAM127B	0.002732
LINC00087	0.002732
LINC00633	0.002732
CT45A1	0.005567
CT45A2	0.005567
CT45A3	0.005567
CT45A4	0.005567
CT45A5	0.005567
CT45A6	0.005567
CXorf48	0.005567
DDX26B	0.005567
LINC00086	0.005567
RNA5SP515	0.005567
SAGE1	0.005567
ZNF449	0.005567
ZNF75D	0.005567
MMGT1	0.0043
SLC9A6	0.0043
snoU13|ENSG00000239080.1	0.004696

FHL1	0.004696
MAP7D3	0.004696
GPR112	0.00961
BRS3	0.0043
HTATSF1	0.004646
VGLL1	0.004646
MIR934	0.0043
LINC00892	0.0043
CD40LG	0.0043
ARHGEF6	0.004001
RBMX	0.004001
SNORD61|ENSG00000206979.1	0.004001
GPR101	0.00488
ZIC3	0.00965
RN7SL325P	0.00965
RN7SKP31	0.003347
FGF13	0.001556
MIR504	0.003347
F9	0.001671
MCF2	0.001688
ATP11C	0.001688
CXorf66	0.001688
MIR505	0.001688
RN7SL727P	0.001688
RNU6ATAC23P	0.001688
SNORA18|ENSG00000252719.1	0.001688
snoU13|ENSG00000238485.1	0.001688
SOX3	0.001177
LINC00632	0.001114
CDR1	0.001114
MIR320D2	0.001631
SPANXB1	0.001631
SPANXB2	0.001631
LDOC1	0.001114
MAGEC1	0.000672
MAGEC3	0.000672
SPANXA1	0.000672
SPANXA2	0.000672
SPANXC	0.000672
SPANXD	0.000672
MAGEC2	0.002817
RNA5SP516	0.00228
snoU13|ENSG00000239188.1	0.002817
SPANXN4	0.003505
RN7SKP81	0.00247
RN7SKP149	0.00247
SPANXN3	0.002817
SLITRK4	0.004103
SPANXN2	0.00235
UBE2NL	0.001027
RN7SKP189	0.000873
SPANXN1	0.000692
SLITRK2	0.001023
TMEM257	0.001023
MIR888	0.001408
MIR890	0.001408
MIR892A	0.001408
MIR892B	0.001408

MIR891B	0.001408
MIR891A	0.001408
RNA5SP517	0.001483
CXorf51A	0.000877
CXorf51B	0.000877
MIR513C	0.001262
MIR513B	0.001262
MIR513A1	0.001262
MIR513A2	0.001262
MIR506	0.001262
MIR507	0.001262
MIR508	0.001262
MIR514B	0.001262
MIR510	0.001262
MIR514A1	0.001262
MIR514A2	0.001262
MIR514A3	0.001262
FMR1	0.001509
FMR1NB	0.001509
AFF2	0.002119
RN7SKP267	0.002295
IDS	0.002855
LINC00893	0.002855
CXorf40A	0.002855
CXorf40B	0.002855
HSFX1	0.002855
HSFX2	0.002855
LINC00894	0.003039
MAGEA11	0.002855
MAGEA8	0.002855
MAGEA9B	0.002855
MAGEA9	0.002855
TMEM185A	0.002855
MIR2114	0.003466
MAMLD1	0.004985
MTM1	0.005367
MTMR1	0.005367
CD99L2	0.003338
U3|ENSG00000253009.1	0.003157
HMGB3	0.003998
MIR4330	0.00551
GPR50	0.00551
VMA21	0.006316
PASD1	0.005485
PRRG3	0.005485
FATE1	0.006316
CNGA2	0.006316
MAGEA4	0.004874
GABRE	0.004874
MIR224	0.004874
MIR452	0.004874
MAGEA10	0.007289
GABRA3	0.008721
MIR767	0.009813
GABRQ	0.014745
CETN2	0.012873
CSAG1	0.012873
CSAG2	0.012873

CSAG3	0.012873
CSAG4	0.012873
MAGEA12	0.012873
MAGEA1	0.012873
MAGEA2B	0.012873
MAGEA2	0.012873
MAGEA3	0.012873
MAGEA6	0.012873
NSDHL	0.012873
PNMA3	0.012873
PNMA5	0.012873
PNMA6A	0.012873
PNMA6B	0.012873
PNMA6C	0.012873
PNMA6D	0.012873
RN7SL190P	0.012873
RN7SL667P	0.012873
ZFP92	0.012873
ZNF185	0.012873
ZNF275	0.012873
snoU13|ENSG00000239037.1	0.012873
HAUS7	0.010932
TREX2	0.012804
BGN	0.010932
ATP2B3	0.012804
FAM58A	0.012873
DUSP9	0.012873
RN7SL687P	0.012873
PNCK	0.012873
SLC6A8	0.012873
BCAP31	0.012956
ABCD1	0.012956
PLXNB3	0.011694
IDH3G	0.011694
SRPK3	0.011694
SSR4	0.011694
PDZD4	0.011694
SNORD36|ENSG00000251846.1	0.011694
L1CAM	0.011694
LCA10	0.011694
AVPR2	0.011694
ARHGAP4	0.011694
NAA10	0.014555
HCFC1	0.014555
RENBP	0.014555
TMEM187	0.014555
IRAK1	0.014555
MECP2	0.011295
MIR718	0.014555
OPN1LW	0.008351
OPN1MW2	0.008351
OPN1MW	0.008351
TEX28P1	0.008351
TEX28P2	0.008351
TEX28	0.008351
TKTL1	0.008351
EMD	0.008314
FLNA	0.008314

RPL10	0.010723
SNORA70|ENSG00000207165.1	0.013749
DNASE1L1	0.013749
TAZ	0.014758
ATP6AP1	0.013502
FAM50A	0.013502
GDI1	0.013502
PLXNA3	0.011633
LAGE3	0.011633
UBL4A	0.011633
SLC10A3	0.011633
RN7SL697P	0.011633
RN7SL742P	0.011633
FAM3A	0.011633
G6PD	0.018155
IKBKG	0.024759
BRCC3	0.017597
CLIC2	0.017597
CMC4	0.017597
CTAG1A	0.017597
CTAG1B	0.017597
CTAG2	0.017597
DKC1	0.017597
F8A1	0.017597
F8A2	0.017597
F8A3	0.017597
F8	0.017597
FUNDC2	0.017597
GAB3	0.017597
H2AFB1	0.017597
H2AFB2	0.017597
H2AFB3	0.017597
MPP1	0.017597
MTCP1	0.017597
RAB39B	0.017597
SMIM9	0.017597
SNORA36A	0.017597
SNORA56	0.017597
TMLHE	0.017597
VBP1	0.017597
IL9R|ENSG00000124334.12	0.015463
SPRY3|ENSG00000168939.6	0.015463
VAMP7|ENSG00000124333.10	0.015463
WASH6P|ENSG00000182484.10	0.015463
WASIR1|ENSG00000185203.7	0.015463

Table S7: Differential sensitivity of 138 drugs in different score groups

Table S7			
	**p**	**cor**	**cor.p**
A.443654	0.000699	0.176515	5.82E-05
A.770041	0.022152	0.126908	0.003989
ABT.263	0.017991	0.105529	0.016801
ABT.888	0.494631	-0.00596	0.892873
AG.01469(	0.558367	-0.05155	0.243787
AICAR	8.57E-16	-0.42164	1.58E-23
AKT.inhib	0.002773	-0.13446	0.002274
AMG.706	3.22E-06	0.212125	1.25E-06
AP.24534	7.02E-18	-0.4431	4.41E-26
AS601245	9.08E-07	-0.27348	2.98E-10
ATRA	0.003275	0.137542	0.001793
AUY922	2.83E-15	-0.39645	9.36E-21
Axitinib	0.076909	-0.11442	0.009492
AZ628	8.39E-07	-0.29132	1.71E-11
AZD.0530	0.545228	0.054824	0.215111
AZD.2281	1.44E-12	-0.40735	6.30E-22
AZD6244	8.98E-20	-0.46675	4.08E-29
AZD6482	7.06E-25	-0.54188	1.73E-40
AZD7762	5.62E-13	-0.38831	6.57E-20
AZD8055	0.074941	-0.11031	0.012421
BAY.61.3(	0.000151	-0.21138	1.36E-06
Bexarotene	5.74E-06	-0.22019	4.73E-07
BI.2536	0.818372	0.024202	0.584443
BIBW2992	2.09E-05	0.250191	9.22E-09
Bicalutami	0.020507	-0.0992	0.024651
BI.D1870	0.011112	0.096944	0.028125
BIRB.0796	2.00E-14	0.397691	6.92E-21
Bleomycin	7.53E-06	-0.2152	8.65E-07
BMS.5097	1.52E-06	-0.24033	3.57E-08
BMS.5369	3.05E-13	-0.37092	3.54E-18
BMS.7081	0.001906	-0.16089	0.000253
BMS.7548	1.82E-30	-0.58364	3.69E-48
Bortezomil	4.51E-10	-0.35153	2.29E-16
Bosutinib	1.27E-12	-0.35344	1.54E-16
Bryostatin.	5.15E-05	-0.21922	5.32E-07
BX.795	4.79E-10	-0.33023	1.62E-14
Camptothe	3.27E-08	-0.32409	5.21E-14
CCT00709	0.000274	-0.18877	1.68E-05
CCT01815	8.83E-09	0.30332	2.23E-12
CEP.701	2.26E-12	-0.37966	4.91E-19
CGP.0829(	0.899269	-0.01547	0.726681
CGP.6047	1.09E-08	-0.34125	1.86E-15
CHIR.9902	8.59E-18	-0.42808	2.83E-24
CI.1040	1.51E-20	-0.49446	5.50E-33
Cisplatin	0.01045	-0.12859	0.003529
CMK	6.54E-12	-0.36577	1.10E-17
Cyclopami	0.000888	-0.18324	2.97E-05
Cytarabine	8.31E-07	-0.25755	3.23E-09
Dasatinib	1.63E-07	-0.27496	2.37E-10
DMOG	4.08E-32	-0.6075	4.57E-53
Docetaxel	0.653536	0.010397	0.814268
Doxorubici	0.890788	0.012605	0.775785
EHT.1864	0.906818	-0.03579	0.418523
Elesclomol	0.001891	0.148874	0.000718
Embelin	2.51E-10	-0.31367	3.56E-13
Epothilone	0.199762	0.078287	0.076468

Erlotinib	0.001744	-0.16124	0.000245
Etoposide	0.000515	-0.19199	1.19E-05
FH535	0.294308	0.083597	0.058475
FTI.277	7.10E-05	-0.20259	3.74E-06
GDC.0449	3.84E-06	-0.28885	2.57E-11
GDC0941	3.89E-08	-0.31882	1.39E-13
Gefitinib	0.299272	0.072101	0.102852
Gemcitabir	5.75E-09	-0.30606	1.38E-12
GNF.2	0.944198	-0.00281	0.949293
GSK26996	0.606153	-0.00804	0.855889
GSK.65039	0.254842	-0.0613	0.165679
GW.44175	8.88E-13	0.388661	6.05E-20
GW843682	0.002436	0.151741	0.000564
Imatinib	0.918156	0.00303	0.945419
IPA.3	1.01E-07	-0.27757	1.58E-10
JNJ.26854	1.14E-10	-0.34513	8.52E-16
JNK.9L	1.02E-12	-0.38582	1.18E-19
JNK.Inhibi	3.37E-05	0.209265	1.74E-06
JW.7.52.1	0.204609	0.067055	0.129325
KIN001.13	1.23E-05	-0.22826	1.73E-07
KU.55933	0.316509	0.032144	0.467557
Lapatinib	0.215661	0.076997	0.08146
Lenalidom	6.80E-05	-0.22927	1.52E-07
LFM.A13	1.28E-33	-0.64393	2.05E-61
Metformin	0.0028	0.127729	0.003758
Methotrexa	9.05E-09	-0.2822	7.58E-11
MG.132	0.024968	-0.11657	0.008224
Midostauri	3.35E-12	-0.33306	9.37E-15
Mitomycin	0.178505	0.071053	0.107963
MK.2206	0.142081	-0.09337	0.034503
MS.275	0.007264	-0.18502	2.48E-05
Nilotinib	1.35E-11	-0.37279	2.33E-18
NSC.87877	6.81E-06	0.25088	8.37E-09
NU.7441	1.20E-05	-0.27982	1.11E-10
Nutlin.3a	0.013001	-0.16104	0.00025
NVP.BEZ2	2.45E-05	-0.24617	1.61E-08
NVP.TAE6	2.32E-11	-0.34553	7.86E-16
Obatoclax.	0.007703	-0.17999	4.13E-05
OSI.906	4.43E-15	-0.4003	3.65E-21
PAC.1	0.001336	-0.13114	0.002922
Paclitaxel	0.412912	-0.04916	0.266376
Parthenolid	3.01E-12	-0.39657	9.08E-21
Pazopanib	4.69E-12	-0.3791	5.59E-19
PD.032590	1.98E-14	-0.40824	5.05E-22
PD.033299	2.05E-05	-0.23307	9.31E-08
PD.173074	0.849522	-0.06252	0.157385
PF.023410	1.51E-30	-0.62119	4.48E-56
PF.470867	5.02E-25	0.54675	2.51E-41
PF.562271	8.53E-16	-0.39514	1.29E-20
PHA.6657	6.47E-07	-0.25421	5.22E-09
PLX4720	1.52E-07	-0.29908	4.63E-12
Pyrimethar	0.432905	-0.04054	0.359541
QS11	0.576122	-0.03806	0.389694
Rapamycin	0.001271	-0.20161	4.17E-06
RDEA119	1.41E-13	-0.38844	6.37E-20
RO.3306	0.004771	0.094207	0.032902
Roscovitine	1.14E-06	-0.27525	2.27E-10
Salubrinal	1.46E-06	0.224061	2.93E-07

SB.216763	3.23E-16	-0.40918	3.97E-22
SB590885	0.26851	-0.08642	0.050425
Shikonin	1.65E-09	-0.3461	6.99E-16
SL.0101.1	0.00047	0.138575	0.001654
Sorafenib	1.80E-06	0.253814	5.53E-09
S.Trityl.L.c	0.037867	0.095254	0.030998
Sunitinib	4.65E-15	-0.43042	1.50E-24
Temsirolim	1.35E-08	-0.31129	5.46E-13
Thapsigarg	0.736668	0.048778	0.270129
Tipifarnib	0.000149	-0.20044	4.75E-06
TW.37	0.490125	-0.06081	0.169045
Vinblastine	0.016168	-0.17484	6.86E-05
Vinorelbine	0.040883	-0.10428	0.018145
Vorinostat	2.25E-06	-0.27058	4.66E-10
VX.680	0.466105	-0.04008	0.364988
VX.702	9.68E-18	-0.45687	8.07E-28
WH.4.023	0.017392	-0.12659	0.00408
WO200909	4.74E-09	-0.34271	1.39E-15
WZ.1.84	0.02282	-0.10994	0.01272
X17.AAG	0.389525	-0.02681	0.544631
X681640	3.51E-08	-0.28505	4.79E-11
XMD8.85	7.26E-19	-0.46687	3.93E-29
Z.LLNle.C	0.113002	-0.09226	0.036712
ZM.44743	0.60034	-0.01327	0.764354
